# Insights into 6S RNA in lactic acid bacteria (LAB)

**DOI:** 10.1186/s12863-021-00983-2

**Published:** 2021-09-03

**Authors:** Pablo Gabriel Cataldo, Paul Klemm, Marietta Thüring, Lucila Saavedra, Elvira Maria Hebert, Roland K. Hartmann, Marcus Lechner

**Affiliations:** 1 Centro de Referencia para Lactobacilos (CERELA-CONICET), Chacabuco 145, San Miguel de Tucumán, 4000 Argentina; 2 Philipps-Universität Marburg, Institut für Pharmazeutische Chemie, Marbacher Weg 6, Marburg, 35032 Germany; 3 Philipps-Universität Marburg, Center for Synthetic Microbiology (Synmikro), Hans-Meerwein-Straße 6, Marburg, 35043 Germany

**Keywords:** 6S RNA, SsrS, ncRNA, CcpA, cre site, Lactic acid bacteria, LAB

## Abstract

**Background:**

6S RNA is a regulator of cellular transcription that tunes the metabolism of cells. This small non-coding RNA is found in nearly all bacteria and among the most abundant transcripts. Lactic acid bacteria (LAB) constitute a group of microorganisms with strong biotechnological relevance, often exploited as starter cultures for industrial products through fermentation. Some strains are used as probiotics while others represent potential pathogens. Occasional reports of 6S RNA within this group already indicate striking metabolic implications. A conceivable idea is that LAB with 6S RNA defects may metabolize nutrients faster, as inferred from studies of *Echerichia coli*. This may accelerate fermentation processes with the potential to reduce production costs. Similarly, elevated levels of secondary metabolites might be produced. Evidence for this possibility comes from preliminary findings regarding the production of surfactin in *Bacillus subtilis*, which has functions similar to those of bacteriocins. The prerequisite for its potential biotechnological utility is a general characterization of 6S RNA in LAB.

**Results:**

We provide a genomic annotation of 6S RNA throughout the *Lactobacillales* order. It laid the foundation for a bioinformatic characterization of common 6S RNA features. This covers secondary structures, synteny, phylogeny, and product RNA start sites. The canonical 6S RNA structure is formed by a central bulge flanked by helical arms and a template site for product RNA synthesis. 6S RNA exhibits strong syntenic conservation. It is usually flanked by the replication-associated recombination protein A and the universal stress protein A. A catabolite responsive element was identified in over a third of all 6S RNA genes. It is known to modulate gene expression based on the available carbon sources. The presence of antisense transcripts could not be verified as a general trait of LAB 6S RNAs.

**Conclusions:**

Despite a large number of species and the heterogeneity of LAB, the stress regulator 6S RNA is well-conserved both from a structural as well as a syntenic perspective. This is the first approach to describe 6S RNAs and short 6S RNA-derived transcripts beyond a single species, spanning a large taxonomic group covering multiple families. It yields universal insights into this regulator and complements the findings derived from other bacterial model organisms.

**Supplementary Information:**

The online version contains supplementary material available at (10.1186/s12863-021-00983-2).

## Background

### Lactic acid bacteria

Lactic acid bacteria (LAB) constitute a genotypically, phenotypically, and phylogenetically diverse group of Gram-positive bacteria that belongs to the taxonomic order of the *Lactobacillales*. Shared metabolic characteristics and evolutionary relationships have been used as common markers for the identification, classification, typing, and phylogenetic analysis of LAB species [1]. During the last few decades, the analysis of 16S rRNA gene similarity was combined with the study of the carbohydrate fermentation profile to classify new bacterial isolates. The ongoing exploration of the *Lactobacillus* genus has led to frequent taxonomic rearrangements [2]. One reason is the presence of odd similarities and ambiguities in 16S rRNA gene sequence comparisons, resulting in a biased annotation of strains, species, and even LAB genera at short and long phylogenetic distances [3]. Currently, LAB are grouped into six families: *Aerococcaceae, Carnobacteriaceae, Enterococcaceae, Lactobacillaceae, Leuconostocaceae*, and *Streptococcaceae*. These groups share the ability to catabolize sugars for the efficient production of lactic acid [4]. LAB constitute the most competitive and technologically relevant group of microorganisms *G*enerally *R*ecognized *a*s *S*afe (GRAS). Their biotechnological relevance is a result of the many beneficial features that can be exploited, for instance, as starter cultures in the food industry, mediating the rapid acidification of raw material [4], or as probiotics, preventing the adherence, establishment, and replication of several enteric mucosal pathogens via exerting multiple antimicrobial activities [5]. Nevertheless, some LAB are opportunistic pathogens and can cause infections in individuals presenting some underlying disease or predisposing condition. The most prominent opportunistic pathogens are members of the genera *Streptococcus (S.)* and *Enterococcus* [6].

LAB are usually exposed to a wide range of harsh stresses, both in industrial environments and throughout the gastrointestinal tract. This includes acid, cold, drying, osmotic, and oxidative stresses [7]. Surviving these unfavorable conditions is a prerequisite to exert their expected activities [8]. While main stress-resistance systems have been documented in some LAB species, their regulation at the molecular level, including the role of non-coding RNAs (ncRNAs), is still far from being understood [9].

### 6S RNA

Over the last decades many small non-coding RNAs have been identified as key regulators in a variety of bacterial stress response pathways and in bacterial virulence [10–12]. A prominent example among these is 6S RNA encoded by a gene frequently termed *ssrS* according to the original gene designation in *Escherichia coli* [13, 14]. A 6S gene is found in nearly all bacterial genomes sequenced so far [15, 16]. This includes species with highly condensed genomes such as the hyperthermophile *Aquifex aeolicus*, species that obtain energy through photosynthesis like *Rhodobacter sphaeroides*, as well as pathogens such as *Helicobacter pylori* [16–19]. The dissemination of 6S RNA and its usually growth phase-dependent and condition-specific expression profile are indicators of the RNA’s regulatory impact. Its mechanistic features have been more intensely studied for the two model organisms *E. coli* and *Bacillus subtilis* [20, 21]. The latter belongs to the *Bacillales*, a sister-order of *Lactobacillales*. 6S RNA is about 160-200 nucleotides in length and adopts a rod-shaped structure with an enlarged internal loop or bulge flanked by large helical arms on both sides [22, 23]. 6S RNA can bind the DNA-dependent RNA polymerase (RNAP) in complex with the housekeeping sigma factor (*σ*^70^ in *E. coli* and *σ*^*A*^ in *B. subtilis*) in competition with regular DNA promoters. This sequestration of RNAP alters the housekeeping transcription at a global level that is seemingly advantageous when facing numerous types of stress [22, 24, 25]. When RNAP is bound, it can utilize 6S RNA as a template for the transcription of short product RNAs (pRNAs). Upon relief of stress, the transcribed pRNAs become increasingly long. When reaching a certain length (∼14 nt in *B. subtilis*), pRNAs can persistently rearrange the structure of 6S RNA to induce RNAP release, thus restoring regular transcription [21, 26–30]. Studies in *E. coli* have provided evidence that nutrients are metabolized faster in 6S RNA knockout strains than in the parental wild type strain [29, 31]. Furthermore, knockout strains might have the so far unexplored potential to produce elevated levels of secondary metabolites such as surfactants.

### 6S RNA in lactic acid bacteria

The importance of 6S RNA in LAB is indicated by studies that report its abundant expression as well as metabolic changes upon its knockout. However, specific 6S RNA analyses in this important group of bacteria are scarce or the studied ncRNA was not recognized as 6S RNA. It is annotated only in about half of all LAB species analyzed in this study (539/1,092 genomes). Here, we identified it in about 91% of all known LAB species. An example is *L. delbrueckii*, an industrial starter for dairy products, where a highly abundant ncRNA was reported [32]. Though its function could not be specified further, the authors suspected it to act as an antisense RNA. In our study, we identified this 210 nt long ncRNA as 6S RNA. In another study, 6S RNA was identified along with two types of pRNAs via RNA sequencing of *S. pyogenes* [33].

For *Lactococcus lactis*, the expression of 6S RNA has been linked to the carbon catabolite repression protein CcpA that binds to DNA at *cis*-acting sequences. These sites are called catabolite responsive elements (*cre*) [34]; *cre* sites are degenerate pseudo-palindromes. In Bacilli a CcpA dimer was shown to bind to dsDNA upon association with the Ser46-phosphorylated form of histidine-containing phosphocarrier protein (HPr-Ser46-P) [35]. In *L. lactis*, 6S RNA levels were found to be increased during stationary and exponential phase in the presence of galactose or cellobiose, but not fructose, as the sole carbon source. CcpA repression is known to be relieved by galactose and cellobiose, but not by fructose. Moreover, 6S RNA was found to be about 3-fold upregulated in a CcpA-deficient mutant [34] and a *cre* element was identified upstream of the -35 region of its promoter. This indicates a potential interaction between CcpA and the 6S RNA gene that might be relevant for LAB in general. Notably, *B. subtilis* 6S-1 and 6S-2 RNA were not identified as a target for CcpA [36].

For *E. faecalis*, a major opportunistic human pathogen, an additional transcript antisense to 6S RNA was detected [37]. The authors proposed its participation in degradation or maturation of 6S RNA as both ncRNA products were present in a processed form. To our knowledge, an equivalent antisense product is not described for *E. coli* [37], *B. subtilis* or any other species to date (own observation). However, interdependent expression of genes around the 6S RNA locus was noticed for other bacteria, e.g. *R. sphaeroides* (Proteobacteria), where a salt stress-induced membrane protein gene on the opposite strand immediately downstream of the 6S RNA locus is expressed at elevated levels in a 6S RNA knockout strain [18].

Apart from these isolated findings, little is known about the sequence, structure, and physiological role of this regulatory ncRNA in the large and widely heterogeneous group of LAB. In this study, we have annotated and analyzed 6S RNAs systematically to lay a foundation for further investigations regarding its role in stress responses, metabolic processes and interactions with eukaryotic cells. Moreover, we investigated how wide-spread and universally relevant the species-specific observations stated above are for LAB (link to CcpA and the presence of an antisense transcript). This is also the first comparative study covering 6S RNAs in a set of taxonomic families, thus making it possible to draw more representative conclusions than in species-wise studies.

## Results

### Dissemination & phylogeny

We searched 6S RNA sequences in 1,092 genomes covering strains from all 371 sequenced LAB species publicly available in the NCBI database at the time of this study [38]. While two 6S RNA copies were reported for some *Firmicutes* including *Bacillus subtilis*, *Bacillus halodurans*, *Clostridium acetobutylicum*, *Oceanobacillus iheyensis*, and *Thermoanaerobacter tengcongensis* [15], only one copy is present in LAB species. It shows more similarity to the major and well described *Bacillus subtilis* 6S-1 RNA than to its paralog 6S-2 RNA [39].

6S RNA was located in 1001 genomes (>91*%*). Additional File 1 lists all loci. Genomes in which a 6S RNA gene could not be identified are predominantly partial genomes with a large number of contigs or scaffolds. When a 6S RNA gene was found in genomes of closely related species/strains, we assumed that the ncRNA is present but not part of the assembly yet. A peculiarity is the genus *Weissella* of the *Leuconostocaceae* family, represented with 13 species in our dataset. While only a weak 6S RNA locus was predicted in no more than four species of this genus, a significant amount of transcription could be shown for the syntenically conserved intergenic region downstream of *rarA* in publicly available RNA-Seq data for *W. confusa* and *W. koreensis* [40, 41]. Moreover, this locus is confined by a transcription terminator in most *Weissella* species. See Additional File 8 for details. This indicates that 6S RNAs in *Weissella* have a distinct singularity that was hardly picked up by our covariance-based search strategy. The typical rod-shaped structure with a central loop or bulge could not be confirmed for these non-canonical candidates.

**Fig. 1:**
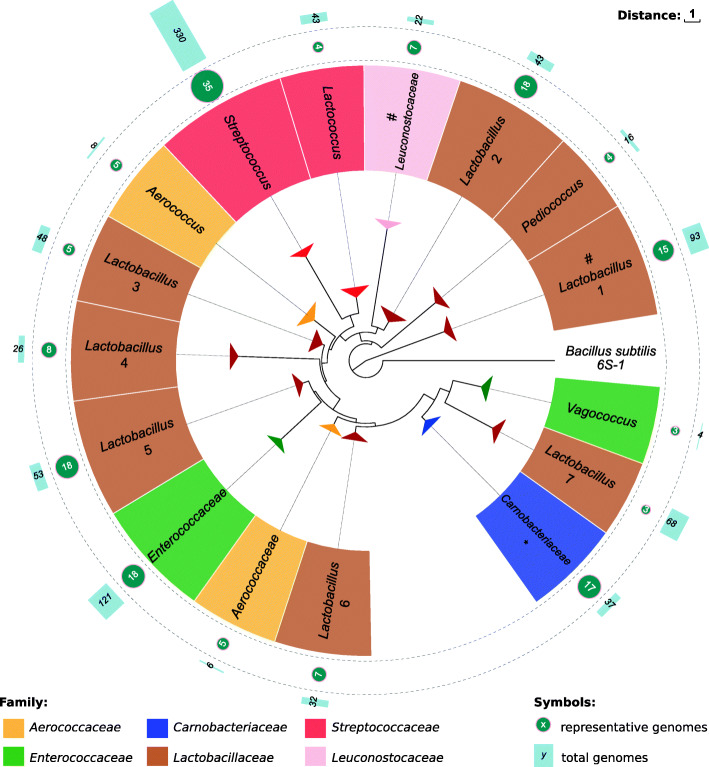
Phylogenetic reconstruction of LAB based on sequence and structure of 6S RNA. 6S-1 RNA from *B. subtilis* is used as an outgroup. The number of different LAB strains is indicated on the outer ring. Turquoise circles show the number of unique 6S RNA sequences within each group. The asterisk at *Carnobacteriaceae* indicates that two species in the group belong to another family. The number sign at *Leuconostocaceae* and *Lactobacillus* 1 remarks non-canonical secondary consensus structures

Figure 1 shows the phylogeny of canonical 6S RNAs identified here based on their sequences and structural properties reconstructed using RNAclust [42] and mlocarna [43]. An alternative version with a resolution that reaches the species level is provided in Additional File 2. The phylogeny well resembles the taxonomic units at the level of genera. A minor exception is the *Carnobacteriaceae* group (blue) that includes *Abiotrophia defectiva* (*Aerococcaceae*) and *Bavariicoccus seileri* (*Enterococcaceae*). At the level of taxonomic families, the genus *Vagococcus* is significantly different from other *Enterococcaceae* (green). Similarly, *Aerococcus* is different from other *Aerococcaceae*. *Lactobacillus* is known to be the most heterogeneous genus within LAB [1]. This is also reflected phylogenetically since the 6S RNAs of this genus are divided into eight well distinguishable groups (*Lactobacillus* 1-7, *Pediococcus*, brown).

### Relation to 16S rRNA phylogeny

The phylogenetic reconstruction of LAB species based on a sequence alignment of selected 16S rRNA sequences is shown analogous to the 6S RNA-based reconstruction in Additional File 3. As expected, the 16S rRNA-based approach better resembles the current taxonomic annotation [2, 44]. The majority of *Lactobacillaceae* species share a common subtree. Notably, a number of species from the Lactobacillus 6 group (6S RNA-based, see Fig. 1) is also located in a separate subtree in the 16S rRNA phylogeny. Similarly, the *Vagococcus* group is isolated from the remaining *Enterococcaceae* in both phylogenies and the same two family-foreign species are found within the *Carnobacteriaceae* subtree, namely *A. defectiva* (*Aerococcaceae*) and *B. seileri* (*Enterococcaceae*). In the 16S rRNA tree, the grouped *Aerococcaceae* are closely related to *Carnobacteriaceae*. The 6S RNA tree, in contrast, splits this group into two subgroups that are not closely related to *Carnobacteriaceae*.

### Synteny

**Fig. 2:**
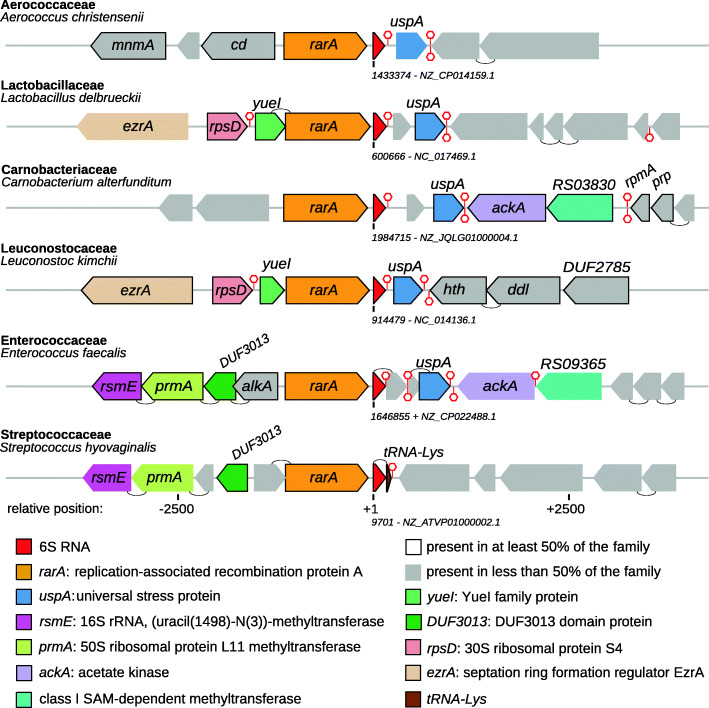
Genomic context of 6S RNA in LAB (4 kb upstream and downstream of the 6S RNA gene). For each LAB family, the genomic locus of one representative species is shown. Genes present in ≥50% of the respective family are indicated with a solid border. Genes found in multiple families are colored. Hypothetical and less conserved proteins are unmarked. Putative Rho-independent terminators are indicated by red hexagons. Genes in close proximity (<20 nt) are indicated by a semicircle connecting them. These could be part of a polycistronic transcript. The complete list of genomic contexts including the NCBI reference codes is provided in Additional File 4. Further gene locus abbreviations: *mnmA*, tRNA 2-thiouridine(34) synthase MnmA; *cd*, cystein desulfurase; *rpmA*, 50S ribosomal protein L27; *prp*, ribosomal-processing cysteine protease Prp; *hth*, helix-turn-helix domain-containing protein; *ddl*, D-alanine-D-alanine ligase; *alkA*, DNA-3-methyladenine glycosylase (adaptive response to alkylative DNA damage)

To characterize the genomic locus of 6S RNA in LAB, a synteny analysis was performed. Proteinortho [45] was used to group the protein-coding genes in the vicinity of the 6S RNA locus. An overview of the genomic context of 6S RNA in LAB is shown in Fig. 2 and in more detail in Additional File 4. The genomic neighborhood of 6S RNA is conserved at the family level. Typically, the same genes are encoded up- and downstream of 6S RNA in the majority of genera from the same taxonomic family but not across LAB in general. Exceptions are the replication-associated recombination protein A gene (*rarA*), that is found upstream of the 6S RNA locus in nearly all species, and the universal stress protein A gene (*uspA*), that is found downstream across almost all species except for *Streptococcaceae* and a few *Aerococcaceae* members.

The upstream *rarA* gene is part of a highly conserved family of ATPases found in prokaryotes as well as eukaryotes. Homologs are known as *mgsA* in *E. coli*, *mgs1* in yeast (maintenance of genome stability A/1), and *WRNIP1* (Werner interacting protein 1) in mammals. The encoded protein is involved in cellular responses to stalled or collapsed replication forks, likely by modulating replication restart [46–48].

The downstream *uspA* gene belongs to a superfamily that encompasses an ancient and highly conserved group of proteins that are widely distributed among bacteria, archaea, fungi, flies, and plants. It was found to be induced during metabolic, oxidative, and temperature stress in *Salmonella typhimurium* [49] and linked to cell sensitivity to ultraviolet light in *E. coli* [50]. *uspA* is known to be differentially expressed in response to a large number of different environmental stresses such as acid and salt stresses, starvation, exposure to heat, oxidants, metals, ethanol, antibiotics, and other stimulants - particularly within the genera *Lactobacillus*, *Streptococcus*, *Enterococcus* and *Lactococcus* [51–53].

### Structure and sequence conservation

**Fig. 3:**
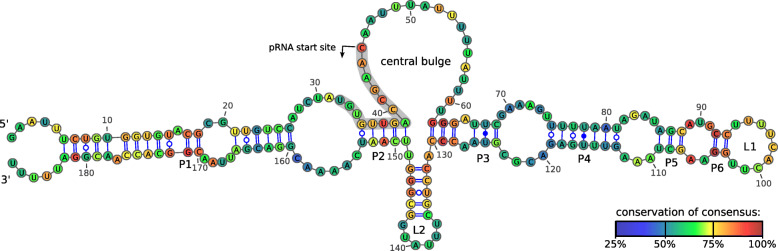
Consensus secondary structure of 6S RNA in LAB. The structure is derived from a sequence-structure-based alignment of 172 unique representative sequences (see Materials and Methods for further details). Colors indicate sequence conservation within LAB. Paired regions P1-P6, the 5’-central bulge, terminal loops L1/L2, and the putative transcription start site of pRNAs are indicated

The consensus structure and sequence conservation of 6S RNA in LAB based on a mLocARNA [43] alignment combined with RNAalifold [54] is illustrated in Fig. 3. Additional File 5 shows the consensus structures at the family level. The consensus of 6S RNA in LAB follows the well-known secondary structure of the canonical 6S RNA [15, 23], featuring an outer closing stem with smaller bulges and loops, a large 5’-central bulge and an apical stem with smaller internal loops capped by the terminal loop L1. Opposite to the 5’-central bulge a hairpin is predicted that was also shown to form in *B. subtilis* 6S-1 RNA [26]. The central bulge harbors the initiation site for product RNA (pRNA) transcription. This consensus and canonical 6S secondary structure is evident in most of the 6S RNA groups: *Aerococcaceae*, *Aerococcus*, *Carnobacteriaceae*, *Vagococcus*, *Enterococcaceae*, *Pediococcus*, *Lactobacillus* 2, 3, 4, 6, 7, *Streptococcus*, and *Lactococcus*, see Additional File 5.

### Product RNAs

**Fig. 4:**
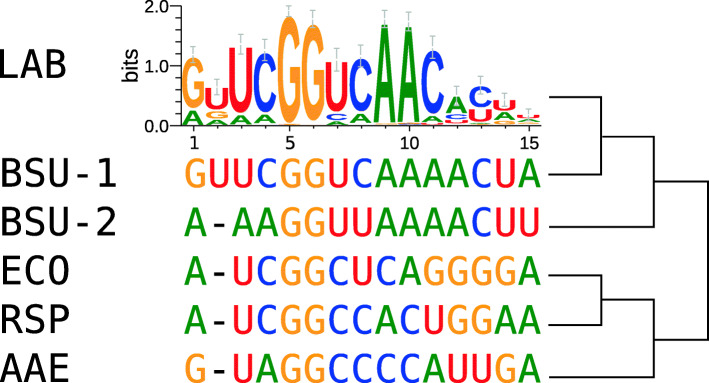
Consensus sequence motif of 6S RNA-derived pRNAs in LAB. The motif found in LAB is indicated at the top. Positions are numbered from the pRNA 5’-end. Known pRNA sequences of other organisms are shown below the motif (BSU-1/2: *B. subtilis* 6S-1 and 6S-2 RNA, ECO: *E. coli*, RSP: *R. spheroides*, AAE: *A. aeolicus*). The conserved GG at position 4/5 or 5/6 is also encoded in 6S RNAs of bacteria outside the LAB group. A neighbor-joining tree based on the LAB consensus and the pRNA sequences (positions 1-15) is indicated on the right

Putative pRNA transcription start sites were inferred from a structural alignment (see Materials and Methods) of 172 representative 6S RNA sequences from LAB species and in relation to those of *E. coli*, *R. spheroides* and *B. subtilis* for which the start sites are experimentally proven. Fig. 4 shows the overall sequence motif. The first eleven nucleotides of the pRNAs are well conserved. This conservation diminishes starting at position 12. GG at position 5/6 as well as AA at position 9/10 are the most conserved in this group. Two G residues are also conserved in experimentally verified pRNAs from more distantly related bacteria such as the Gram-negatives *E. coli*, *A. aeolicus* and *R. spheroides*, but in these cases at positions 4/5 (Fig. 4). Notably, a highly conserved adenine immediately upstream of the pRNA start sites was identified in the 6S RNAs of LAB species as well as in the reference 6S RNAs included in Fig. 4.

Based on the pRNA sequence (positions 1-15), LAB pRNAs are closely related to pRNAs synthesized from *B. subtilis* 6S-1 RNA as template (Fig. 4). Although the 6S-1 pRNA sequence shows differences to the LAB pRNA consensus, major hallmarks (upstream adenine, GG dinucleotide, AA at position 9/10) are still present. Hence, despite the considerable phylogenetic distance, similarities to the pRNA sequence found in LAB are clearly recognizable.

**Fig. 5:**
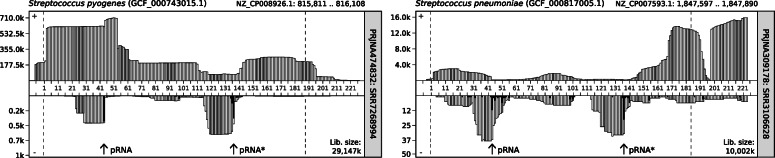
Publicly available RNA-Seq datasets of *Streptococcus pyogenes* (left) and *Streptococcus pneumoniae* (right) mapped to the 6S RNA locus. 6S RNA transcripts are shown in the upper part. pRNA sequences are shown in the lower part in antisense direction. In each case, two short antisense transcripts can be found (pRNA, pRNA^*^, arrows indicate start sites)

We screened 115 publicly available RNA-Seq datasets for expression of 6S RNA and the presence of pRNAs. These small transcripts are usually depleted in sample preparation for RNA-Seq or neglected in data processing that typically focuses on longer RNAs such as tRNAs or mRNAs. Moreover, we found that pRNAs are underrepresented in adapter ligation libraries compared to poly(A)-tailing libraries [55]. It is thus not surprising that only small numbers of pRNA reads were identified in most RNA-Seq libraries. We yet found robust evidence for pRNAs in *Streptococcus pneumoniae* and *Streptococcus pyogenes* RNA-Seq data (Fig. 5), which also supports the predicted pRNA start site (Figs. 3 and 4) [56, 57]. Two pRNA transcripts were previously reported for *S. pyogenes*, but their sequences were not provided [33]. Here we confirm these findings. We find one alternative transcription start site (pRNA^*^) located around position 136 that starts at the beginning of the L2 loop (see Fig. 3). The alternative pRNA transcript likely results from 6S RNA binding RNAP in inverse orientation. Similar observations have been made for *Helicobacter pylori* [19]. Notably, neither the pRNA nor the pRNA^*^ sequences have alternative matches in the respective genomes. It is thus unlikely that these transcripts derive from another locus. Additional File 6 illustrates further RNA-Seq results. While pRNAs were also found in libraries from *E. faecalis*, the number of reads is too low to draw safe conclusions.

### CcpA-binding catabolite responsive elements

**Fig. 6:**
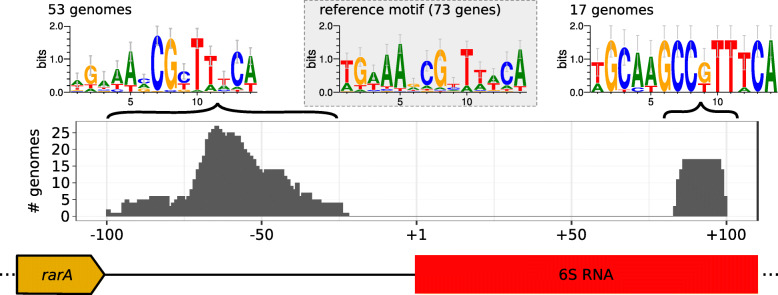
Position and motif of located *cre* sites. Motifs indicated at the top represent the *cre* sites upstream of the 6S RNA promoter (left) and within the 6S RNA gene (right). Both show high conservation. The experimentally verified *cre* motif of 73 genes of *L. lactis* [79] is shown in the center for comparison

A functional *cre* site upstream of the 6S RNA promoter was reported in *L. lactis*, suggesting that 6S RNA expression is regulated depending on the available carbon source [34]. An equivalent *cre* site could be found in about one-third of all LAB species. Fig. 6 illustrates the location and sequence conservation of the two *cre* sites at the 6S RNA locus. Additional File 2 shows a detailed overview of all species with *cre* sites in the 6S RNA region. Additional File 7 lists the respective motif sequences along with their positions and p-values. *cre* sites are most frequently found in *Enterococcaceae* but also in several *Streptococcaceae* and the *Lactobacillus* groups 6 and 7 (see Fig. 1). Mainly in *Streptococcaceae* and *Lactobacillus* group 6, potential *cre* sites were also identified within the 6S RNA coding sequence. Notably, *L. coryniformis*, *L. rennini*, *L. vaginalis*, *S. canis*, *S. didelphis*, *S. equi*, *S. pantholopis* and *S. phocae* do not have a strong, detectable *cre* site at the 6S RNA promoter but only within the 6S RNA coding region; both sites were detected in *L. backii*, *L. bifermentans*, *S. castoreus*, *S. gallolyticus*, *S. halotolerans*, *S. ictaluri*, *S. iniae*, *S. parauberis* and *S. uberis*.

### Expression and antisense transcripts

A total of 115 publicly available RNA-Seq libraries representing 24 different LAB genera were screened for the expression of 6S RNA, pRNAs and long antisense transcripts as described for the *Enterococcus faecalis* V583 strain [37]. Detailed results for each library are shown in Additional File 6.

6S RNA transcripts were highly abundant in general (usually 1-2% of all reads in the RNA-Seq libraries), indicating active transcription in LAB grown under a wide variety of culture conditions and stresses. In line with previous findings [37], however, we did not find evidence for long antisense transcripts of 6S RNA in any RNA-Seq library including those from other *Enterococcus faecalis* strains (OG1RF, 12030, and ATCC 29212), indicating that such transcripts are not a common trait among LAB.

## Discussion

Here we identified the 6S RNA gene at a well-conserved genomic locus in LAB species that distinguishes this bacterial group from related bacterial clades. While the consensus secondary structure is typically canonical as described for *B. subtilis* 6S-1 RNA, we could not verify this for candidates of the genus *Weissella*. Nevertheless, we identified evidence for significant transcription of the respective loci in publicly available RNA-Seq libraries for two strains, see Additional File 8. This confirms a weak 6S RNA candidate in *W. koreensis*. Although no relevant match was found for *W. confusa*, the intergenic region downstream of the syntenically conserved *rarA* showed transcription that matched a 6S RNA transcript even though its putative secondary structure did not match a canonical 6S RNA. A TATAAT sequence is present at the -10 region of all candidates reported for *Weissella*, indicating the presence of a promoter. Similarly, a rho-independent terminator was predicted at the RNA’s proposed 3’-end. Thus, the presence of an actively transcribed 6S RNA-like transcript can be assumed. It will be interesting to investigate the functional consequences of this structural alteration.

Carbon catabolite control is a major regulatory mechanism for the modulation of metabolic activity of microorganisms to optimize carbon metabolism and energy use. It involves both carbon catabolite repression and activation. In most low-GC-content Gram-positive bacteria this regulation is mediated by the catabolite control protein A (CcpA) that binds to DNA at *cis*-acting sequences. These are called catabolite responsive elements (*cre*) and are located either in the promoter region or within the coding sequence of the regulated gene [36]. CcpA can function as an activator or may repress transcription depending on its location within a regulated gene or operon [58]. We found strong evidence for *cre* sites upstream of the 6S RNA promoter in about a third of all LAB species, mainly in *Enterococcaceae* but also in *Streptococcaceae* and some *Lactobacillus* subgroups. For *Streptococcaceae* and *Enterococcaceae*, the presence and regulatory importance of these *cre* sites has been reported and studied previously [59, 60]. On the basis of previous reports, our findings suggest that 6S RNA expression is under the negative control of CcpA in many LAB species. This was shown e.g. for *L. lactis* where 6S RNA is 3-fold upregulated upon deletion of the *ccpA* gene [34].

For several 6S RNA genes, *cre* sites were also identified internally - in some cases in addition to the site at the 6S RNA promoter (see Additional File 2). The presence of two *cre* sites regulating the expression of *cid* and *lrg* genes in *Streptococcus mutans* has already been described, but in this case both sequences were upstream of the transcription start site of the above-mentioned genes [61]. In *B. subtilis*, *cre* sites upstream of promoters were found to be primarily activated by CcpA, while *cre* sites overlapping promoters had repressing effects [35]. As the *cre* sites in LAB overlap the -35 region of 6S RNA gene promoters (Fig. 6), CcpA-binding is likely inhibitory; *cre* sites located further downstream of the transcription start site may act as roadblocks or repress initiation of transcription through interaction with RNAP [62]. Future studies may address the interplay of the two *cre* sites at/within the 6S RNA gene. Although speculative at present, it is also a possibility that CcpA binds to 6S RNA at the internal *cre* site, taking into account that 6S RNAs mimic an open DNA promoter [22].

The identified *cre* sequences share a high degree of similarity to the consensus sequences previously described for other LAB such as *L. lactis*, (see Fig. 6) as well as to other Gram-positive bacteria such as *B. subtilis* [36, 63]. Recent studies on the promoter region of the *PTS-IIC* gene cluster of *L. lactis* demonstrated the importance of nucleotide identity at positions 7 and 12 of the 14-nt long *cre* site. Specific mutations within the -35 promoter element resulted in constitutive expression of the downstream gene in the presence of glucose, while other mutations enhanced promoter activity in the presence of cellobiose [63].

The prediction of transcription start sites for pRNAs was based on the structural alignment to other 6S RNAs and could be verified by RNA-Seq data in two cases. This study is the first that deduces pRNAs for a large taxonomic group covering multiple families. We found a highly conserved sequence up to around position 11. This may point to similar kinetics of pRNA synthesis and pRNA-induced 6S RNA refolding [26]. Strikingly, GG at positions 5/6 or 4/5 of the pRNAs appears to be a key feature conserved beyond LAB.

A general property of the 6S RNA locus in LAB is its location between the *rarA* and *uspA* genes. Gene order conservation can be used not only to evaluate the orthology of genomic regions but might also hint at functional relationships between genes [64]. RarA is proposed to act at stalled DNA replication forks upon DNA damage and UspA alters the expression of a variety of genes that help to cope with stresses. As 6S RNA was shown to have a role in cellular stress responses to ensure longtime cell survival, all three gene products might be part of an overachrching stress response network. The *rarA* gene is in close vicinity to the 6S RNA locus across all families including the 6S-1 RNA locus of the non-LAB firmicute *B. subtilis* (see Additional File 4). In the latter, however, *rarA* is encoded in the opposite direction and known to be monocistronic [65]. The RNA-Seq data presented in Additional Files 6 and 8 and the presence of a downstream terminator in most species indicates that the 6S RNA gene is monocistronic as well. However, several *Streptococcaceae* members encode a tRNA-Lys immediately downstream of 6S RNA, suggesting that both genes are part of the same operon. This assumption is supported by RNA-Seq data for *S. pneumoniae* (Additional File 6, p. 43) showing that both ncRNAs have the same transcript level [56]. Thus, both RNAs are likely processing products of the same primary transcript. Other notable syntenic bonds are not universally preserved for LAB but within and also across particular LAB families. Examples are the acetate kinase, class I SAM-dependent methyltransferase, 16S rRNA methyltransferase, and the 50S ribosomal protein L11 methyltransferase. While the function of the other frequently linked genes is unknown so far, this data suggests a cluster of growth-relevant and stress-related genes that 6S RNA is part of. Typically, these genes appear to be transcribed independently (with the exception of 6S RNA and tRNA-Lys in a number of *Streptococcaceae*). Therefore, the possibility of a common functional context remains vague at present.

## Conclusions

Lactic acid bacteria include highly heterogenous species and the study of the role of non-coding RNA molecules, particularly 6S RNA, in the regulation of the response of these bacteria to different stress conditions has many potential applications, both within industrial and health contexts. The global transcription regulator 6S RNA is present in nearly all species and well-conserved throughout this group. It generally resembles the canonical form that is well described for *B. subtilis* 6S-1 RNA. LAB 6S RNAs also share the syntenic proximity to *rarA*, located upstream of 6S RNA in nearly all LAB genomes. Many species additionally encode the UspA protein downstream of 6S RNA, which makes its identification comparably easy. The experimental evidence that was processed and analyzed in this study also demonstrated that 6S RNA is expressed in a multitude of LAB species across all taxonomic families and under varying culture conditions. This also highlights the important regulatory role of this ncRNA in bacterial metabolism, further supported by the frequent presence of *cre* sites in its promoter and coding region. The conservation of 6S RNAs makes it plausible to generally apply our findings to any LAB species in order to explore its biotechnological potential.

## Methods

### Genomes

Several thousand genomes representing 576 species that cover 48 genera were listed as part of the *Lactobacillales* order according to the NCBI taxonomy classification (date of retrieval 10/09/2018) [38]. In order to work with a reasonably representative set, we focused on the genomes with the best respective assembly status for each species. The species *Enterococcus faecium* for example comprises 1109 genomes/subspecies. Fifty-one out of these are marked as “Complete Genome” and were thus considered in the present work. *Lactobacillus fuchuensis* is represented with three genomes out of which the most complete assembly is marked as “Chromosome” that was thus considered, and so on. Additionally, we added 13 strains that were characterized by our institute (CERELA-CONICET) even though they did not meet this criterion. Species with yet unclear specific names (sp.) were neglected. A total of 1,092 genomes were considered in this study. An overview of the genera analyzed here can be found in Table 1. A detailed list of the species and genome assembly levels is provided in Additional File 1. The respective genomes and genomic annotations were downloaded via ftp.ncbi.nlm.nih.gov from the NCBI database [38].

**Table 1:** Genomes overview

Family	Genus	Genomes used / Genomes available
Aerococcaceae	*Abiotrophia*	1 / 2
	*Aerococcus*	8 / 61
	*Dolosicoccus*	2 / 3
	*Eremococcus*	1 / 2
	*Facklamia*	3 / 9
	*Globicatella*	1 / 4
Carnobacteriaceae	*Agitococcus*	1 / 1
	*Alkalibacterium*	1 / 8
	*Allofustis*	1 / 1
	*Atopobacter*	1 / 1
	*Atopococcus*	1 / 1
	*Carnobacterium*	9 / 41
	*Dolosigranulum*	10 / 12
	*Granulicatella*	1 / 7
	*Jeotgalibaca*	1 / 4
	*Lacticigenium*	1 / 1
	*Marinilactibacillus*	1 / 5
	*Trichococcus*	7 / 15
Enterococcaceae	*Bavariicoccus*	1 / 1
	*Enterococcus*	114 / 2105
	*Melissococcus*	2 / 14
	*Tetragenococcus*	5 / 19
	*Vagococcus*	4 / 6
Lactobacillaceae	*Lactobacillus*	460 / 1680
	*Pediococcus*	25 / 61
	*Sharpea*	1 / 4
Leuconostocaceae	*Convivina*	1 / 1
	*Fructobacillus*	5 / 9
	*Leuconostoc*	23 / 118
	*Oenococcus*	3 / 208
	*Weissella*	23 / 43
Streptococcaceae	*Floricoccus*	2 / 2
	*Lactococcus*	44 / 168
	*Streptococcus*	328 / 12076

Distribution and number of genomes that were retrieved and downloaded from the NCBI database according to the “most complete genome” criterion

### 6S RNA prediction

Putative 6S RNAs encoded in LAB genomes were identified in multiple steps. A BLAST-based approach was performed using available 6S RNA annotations given in the NCBI RefSeq annotation, from Wehner *et al.*, and from the Rfam seed sequences for the 6S/SsrS RNA family (RF00013, Version 14) to cover the currently known 6S RNAs [16, 66, 67]. An e-value threshold of 10^−30^ was applied. Previously not annotated 6S RNAs were identified with a covariance-based search performed with INFERNAL (v1.1.1) [68] using the “6S/SsrS RNA” family model as query (see above). Initially, no thresholds were set. Based on the assumption that each genome should encode at least one 6S RNA gene, the highest-scoring hit for each genome was assumed as a true hit. Compared to this, the e-values of the second-best hits were worse by orders of magnitude. A manual inspection on a sample basis confirmed that those were not likely to be valid 6S RNA candidates. Hence, an e-value threshold of 10^−8^ was applied. In this case, a primary hit was found in most species while unexpected secondary hits were rare and could be judged manually in later stages. Overlapping hits were joined. Hits were found in 973 out of 1092 genomes. Redundant sequences were merged to a single representative sequence resulting in 330 unique sequences that were aligned using Clustal Omega (v1.2.1) [69]. Sequences with an edit distance of ten or less were merged to their consensus sequence to further reduce the amount of redundancy. 188 representative 6S RNA sequences remained. We checked for isolated sequences in the secondary structure clustering analysis (see below) and non-canonical secondary structures using RNAfold (v2.1.9) [54]) as well as suspicious alignments to further remove non-canonical and doubtful hits. The following sixteen 6S RNA candidates were discarded manually in the first round: *Agitococcus lubricus*, *Lactococcus fujiensis*, *Facklamia hominis*, *Pediococcus damnosus*, *Lactobacillus babusae*, *Pediococcus cellicola*, *Lactobacillus cacaonum*, *Lactobacillus mucosae*, *Lactobacillus coleohominis*, *Lactobacillus gastricus*, *Lactobacillus equigenerosi*, *Lactobacillus malefermentans*, *Lactobacillus oryzae*, *Oenococcus oeni*, *Weissella kandleri*, and *Weissella koreensis*. In total 172 representative 6S RNA sequences covering 947 genomes remained. This set was used for further analyses.

For each genome without an annotated canonical 6S RNA (including those discarded manually in the first round), a second search iteration was performed with a LAB-specialized covariance model that was build based on all canonical 6S RNAs identified before. The e-value threshold was reduced to 0.1 and all search heuristics were turned off (cmsearch –max). In addition, the correct genomic locus was ensured by only allowing hits within 2000 nt from *upsA* and/or *rarA* homologs. Both are typically encoded in close vicinity to 6S RNA gene (see Results section “Synteny”). The homologs were annotated using BLAST (v2.8.1+) [66] with an e-value of 10^−40^ based on the sequences found in the synteny analysis. In this way, additional syntenically supported 6S RNA candidate genes were identified in 54 genomes. These are marked as “2nd-iteration” in Additional File 1 that lists all 6S RNAs annotated for LAB.

### Prediction of rho-independent terminators

Terminators were predicted using TransTermHP (v2.09) [70]. An adaptive threshold was used to ascertain significant predictions. Each genome was shuffled ten times while preserving its mono- and di-symbol composition. We then compared the number of hits above any given threshold between the shuffled genomes and original genome. The threshold was chosen such that the average number of hits in the shuffled genomes was no more than 5% compared to the hits in the original genome. E.g. if we find 100 hits above a score of 90 in the genome, the average number of hits in the shuffled genomes above the same score cannot exceed 5, otherwise a higher threshold is chosen. In the absence of significance values provided by the prediction tool, this method roughly estimates a p-value threshold of 0.05 for terminator hits. Overlapping hits were merged. In additon, RNIE (v0.01) was used with default parameters for a genome-wide prediction [71]. For the relevant regions, the results were a subset of the former predictions.

### Consensus secondary structure

All representative 6S RNA candidates were aligned using mLocARNA (v2.0.0RC8), a local structural alignment algorithm for RNA secondary structures [43]. To locate the putative start sites for pRNAs in LAB, three well-studied 6S RNA instances were added as references from which the start sites were then projected to the LAB 6S RNAs. Namely *Escherichia coli* K12 (GCF_000005845.2) and *Bacillus subtilis* 168 (GCF_000009045.1), which codes for two paralogs, 6S-1 and 6S-2 RNA (also known as BsrA and BsrB) [39, 72]. The consensus secondary structure was then calculated with RNAalifold (v2.4.13) [54] and visualized using VARNA (v.3.93) [73], excluding the folding references.

### Prediction of pRNAs

The transcription start of 6S RNA-derived pRNAs was determined based on the structural alignment mentioned above. Based on previously characterized transcription start sites in other bacteria [26, 55, 74], we assumed the equivalent positions within LAB 6S RNAs. The putative pRNA sequences of 16 nt length were aligned with Clustal Omega (v1.2.1) [69]. We found a strong consensus sequence motif (see Results) that we used to further adjust the pRNA start site by shifting it for up to three nucleotides in case of suboptimal matches. The motif composition was calculated using WebLogo (v2.8.2) [75].

### Phylogeny with secondary structure clusters

The sequences of the 6S RNA candidates identified in the first round were clustered hierarchically based on their structured RNA motifs using RNAclust [42]. This approach combines the base pair probability matrix of the secondary structure distributions (via RNAfold (v2.1.9) [54]) and a sequence-structure alignment based on LocARNA [43]. *Bacillus subtilis* 168 (GCF_000009045.1) 6S-1 RNA (BsrB) was added as an outgroup [39]. The resulting tree can be found in Additional File 2, while a condensed version is shown in Fig. 1, visualized using Evolview (v3) [76].

### 16S rRNA phylogeny

16S rRNA sequences were identified using BLAST (v2.8.1+) [66] with an e-value of 10^−20^ based on the 16S rRNA reference sequences provided by the NCBI database [38]. Redundant sequences were merged. Sequences were aligned using muscle (v3.8.1551) [77]. The 5’- and 3’-end of the 16S rRNA alignment were trimmed such that <25% of all sequences had remaining gaps in these regions. The phylogenetic reconstruction was performed with RAxML (v8.1.20) [78] using the General Time Reversible model (GTR) with optimization of substitution rates and the GAMMA model of rate heterogeneity and 1000 bootstrap iterations. The phylogenetic reconstruction was visualized using Evolview (v3) [76].

### Synteny

The amino acid sequences of ten protein-coding genes 5000 nt up- and downstream of the predicted 6S RNA locus were fetched from the NCBI database. Orthologous groups were predicted with Proteinortho (v6.13) [45]. To avoid an overrepresentation bias, equivalent and similar 6S RNA sequences were represented by a single reference strain rather than all strains of the respective species (see “Detection of 6S RNAs”). Genes found in fewer than 50% of each family were omitted from the analysis. For each LAB family, one species that best represented the genomic context of all family members was chosen.

### CcpA-binding catabolite responsive elements

The sequence motif for *cre* sites was derived from experimental *B. subtilis* data [36] that also fits previously derived *L. lactis* data [79] as shown in Fig. 6. However, we preferred the former as it yields a higher number of underlying sequences, which strengthens the derived p-values for motif matches and thus avoids false positive predictions. The 6S RNA sequences along with their 100 nt upstream regions were used to find sequences matching the *cre* motif using MAST [80]. Typically, this position overlapped with the 3’-end of the *rarA* gene. Hence, we did not expect binding sites further upstream to be relevant to 6S RNA. We used the dinucleotide distribution of the respective genomes as background for each e-value calculation. The default e-value threshold of 10 and p-value threshold of 10^−5^ was applied. The resulting motifs were separated in two groups: Upstream of the 6S RNA promoter and within the 6S RNA coding region as shown in Fig. 6.

### Expression

Available RNA-Seq datasets for LAB were located in the NCBISRA archive and downloaded on 12-112018 [38]. In total 115 RNA-Seq libraries were analyzed covering 24 different LAB species. Read sequences were extracted using the NCBI-provided fastq-dump (v2.8.2). Adapter removal and read trimming was performed using cutadapt (v1.12) [81] followed by a quality control with fastqc (v0.11.5) [82]. Processed reads were mapped to the respective genomes with segemehl (v0.2.0) [83]. An e-value threshold of 0.0001 was applied. The mapped data was visualized for each 6S RNA locus using custom scripts. Additional File 6 shows all results and data sources in detail.

## Supplementary Information

**Additional file 1** List of genomes and 6S RNAs (xls). List of LAB genomes used in this study including tax annotation, assembly status, location of the predicted 6S RNA.

**Additional file 2** Full 6S RNA phylogeny (pdf). Sequence- and structure-based reconstruction of 6S RNA phylogeny in LAB including the annotation of species with located *cre* sites. Full taxonomic resolution of Fig. 1.

**Additional file 3** 16S rRNA phylogeny (pdf). Phylogenetic reconstruction of LAB 16S rRNA.

**Additional file 4** Full genomic context of 6S RNA in LAB (pdf). Full genomic context of 6S RNA in LAB. Full taxonomic resolution of Fig. 2.

**Additional file 5** 6S RNA grouped consensus alignment (pdf). Folded consensus structure of the 6S RNA groups analogous to Fig. 3.

**Additional file 6** RNA-Seq results (pdf). Visualization of RNA-Seq libraries mapped to the respective 6S RNA loci.

**Additional file 7** Predicted *cre* site motifs (xls). Predicted *cre* sites sequences and positions relative to the 6S RNA start site.

**Additional file 8** 6S RNA evidence in *Weissella* (pdf). RNA-Seq data, genomic context and sequences of putative 6S RNA loci in *Weissella*.

## Data Availability

Genomes of lactic acid bacteria were downloaded from ftp://ftp.ncbi.nlm.nih.gov/genomes/ (2019-08-07). Species names, chromosome and tax ids, fasta paths and annotated 6S RNAs are provided in Additional File 1. RNA-Seq data was retrieved from NCBI SRA at https://www.ncbi.nlm.nih.gov/sra. Bioproject and SRA ids are listed in Additional Files 6 and 8. The 6S/SsrS RNA family seed sequences RF00013 provided by RFAM at http://rfam.xfam.org/family/RF00013 (Version 14) and the sequences by Wehner et al. provied at https://www.rna.uni-jena.de/supplements/6SRNA/ (2019-08-07) were used for initial 6S RNA prediction.

## Abbreviations

GRAS: Generally Recognized as Safe
LAB: Lactic acid bacteria
RNA: ribonucleic acid
RNAP: DNA-depended RNA polymerase complex
cre site: ccpA-binding catabolite responsive element
