# Supplementary material for: Insights into 6S RNA in lactic acid bacteria (LAB)
Source: BMC Genom Data. 2021 Sep 3;22:29. doi: 10.1186/s12863-021-00983-2 (PMC8414754; doi:10.1186/s12863-021-00983-2)

Additional File 2 — Full 6S RNA phylogeny

Sequence- and structure-based reconstruction of 6S RNA phylogeny in LAB. Canonical 6S RNAs were clustered hierarchically using `RNAclust` and `mlocarna`. Family membership is indicated by color. 6S-1 RNA from *B. subtilis* is used as outgroup. The full number of represented genomes is indicated in blue boxes in the outer ring. Circles in the outer ring indicate whether and where a potential cre-site were identified at the 6S RNA locus.

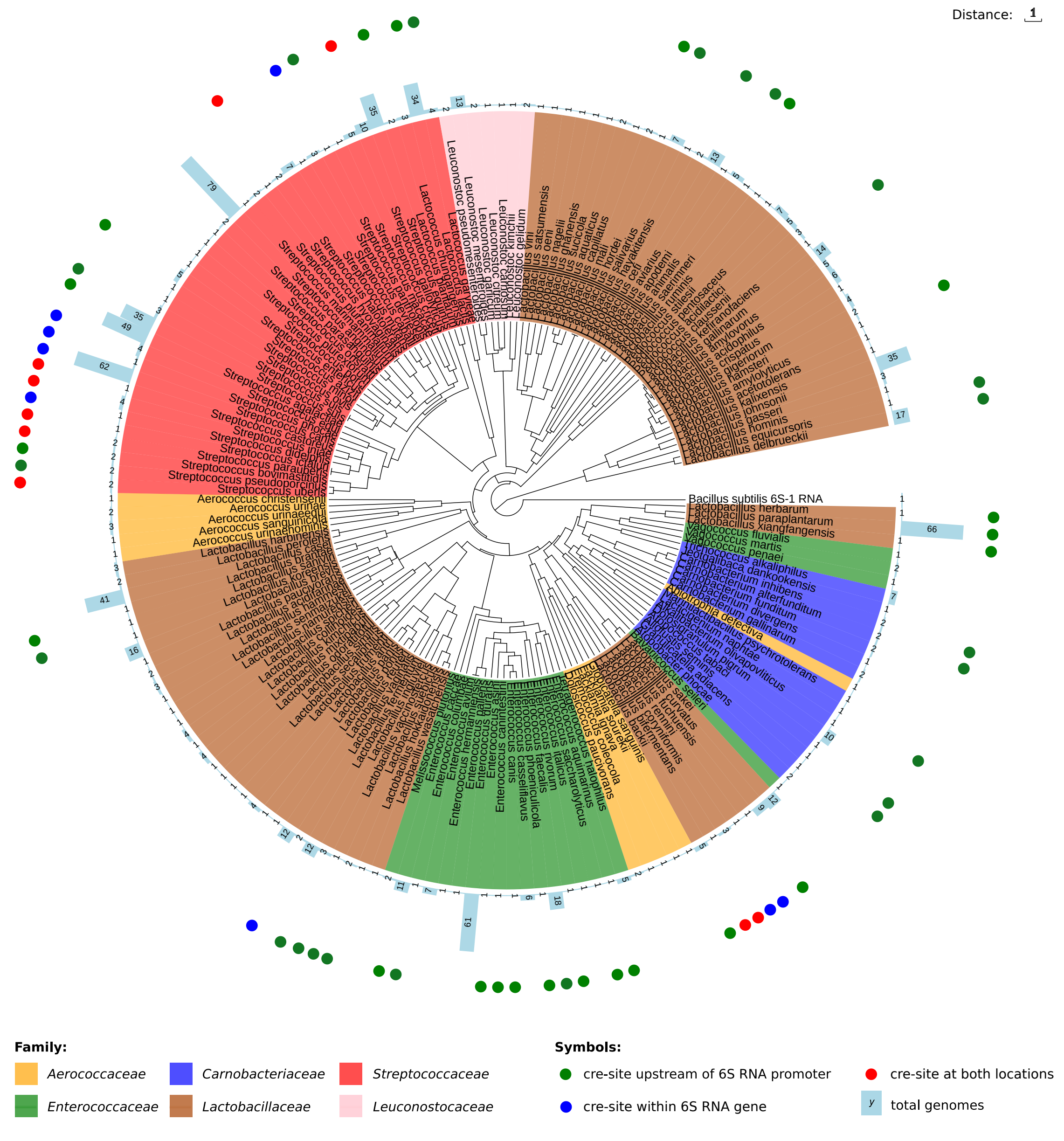

Supplement: Supplementary file 2 — Additional file 2 Full 6S RNA phylogeny (pdf). Sequence- and structure-based reconstruction of 6S RNA phylogeny in LAB including the annotation of species with located cre sites. Full taxonomic resolution of Fig. 1. [file 12863_2021_983_MOESM2_ESM.pdf]
