# Supplementary material for: Insights into 6S RNA in lactic acid bacteria (LAB)
Source: BMC Genom Data. 2021 Sep 3;22:29. doi: 10.1186/s12863-021-00983-2 (PMC8414754; doi:10.1186/s12863-021-00983-2)

Additional File 3 — 16S rRNA phylogeny

Supplemental Figure 2: Sequence-based reconstruction of 16S rRNA phylogeny in LAB. The phylogenetic reconstruction was performed with `RAxML` using the GTR model with an optimization of substitution rates and the GAMMA model of rate heterogeneity. 1000 bootstrap iterations.

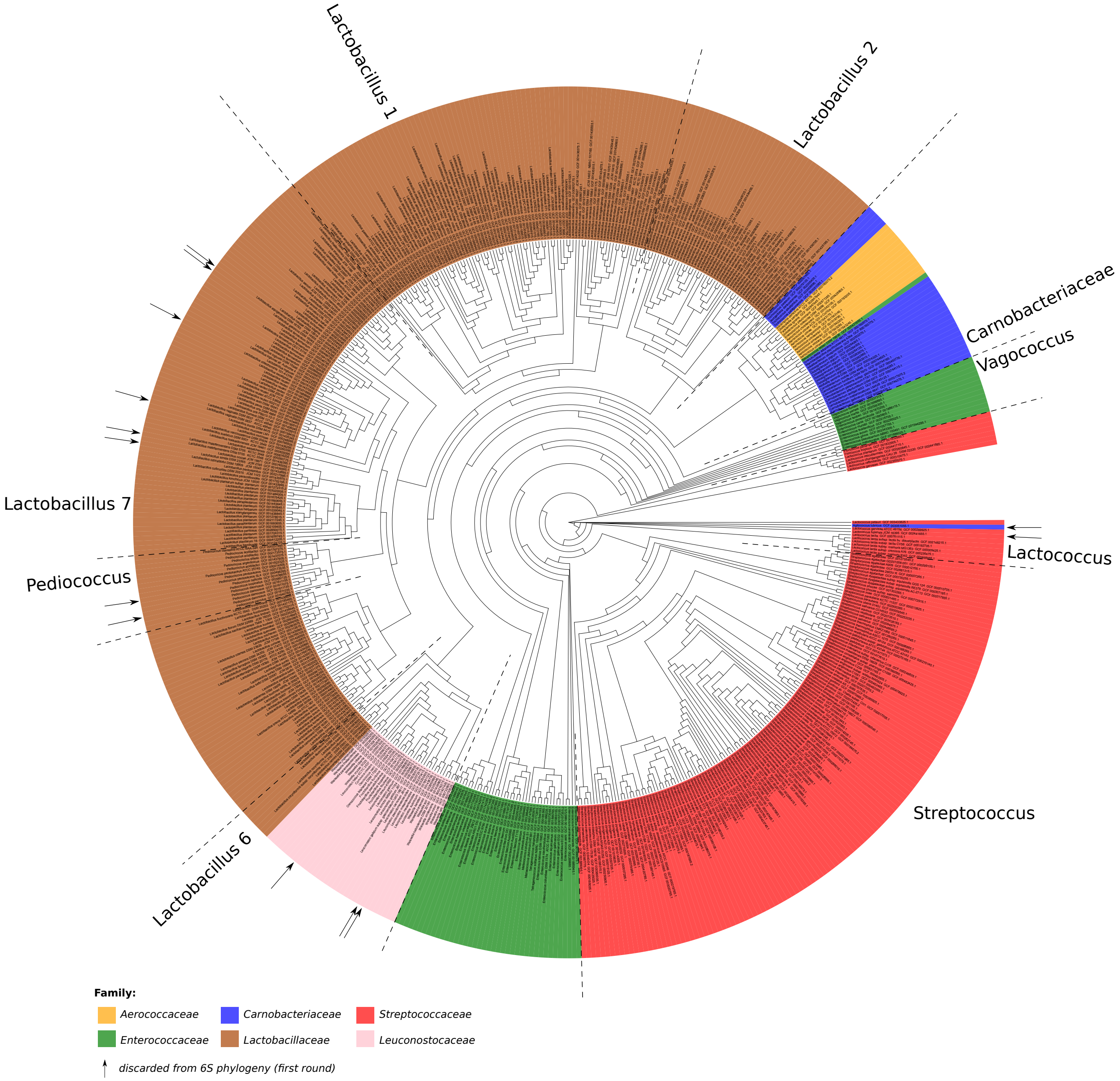

Supplement: Supplementary file 3 — Additional file 3 16S rRNA phylogeny (pdf). Phylogenetic reconstruction of LAB 16S rRNA. [file 12863_2021_983_MOESM3_ESM.pdf]
