# Supplementary material for: Insights into 6S RNA in lactic acid bacteria (LAB)
Source: BMC Genom Data. 2021 Sep 3;22:29. doi: 10.1186/s12863-021-00983-2 (PMC8414754; doi:10.1186/s12863-021-00983-2)
Supplement: Supplementary file 4 — Additional file 4 Full genomic context of 6S RNA in LAB (pdf). Full genomic context of 6S RNA in LAB. Full taxonomic resolution of Fig. 2. [file 12863_2021_983_MOESM4_ESM.pdf]

Additional File 4 — Full genomic context of 6S RNA in LAB

For each family the genomic context around the 6S RNA (±5000nt) is shown. `Proteinortho` was used to group the protein-coding genes. The displayed names represent the unique ortholog groups (# is used for groups with same name). If no ortholog were found the gene is marked with a underscore prefix and is thus excluded from the analysis. Genes with a solid border were found in $\geq 50\%$ of the respective family. Genes found in multiple families are colored. *rarA* and *uspA* are colored orange and blue respectively. Putative Rho-independent terminators are indicated by red hexagons. Genes in close proximity (<20 nt) are indicated by a semicircle connecting them. These could be part of a polycistronic transcript.

Each genomic context contains 5 lines of information to the left:

(1) family name and conservation score

(2) the group of conserved gene order

(3) species name

(4) species id

(5) chromosome name

The given conservation score describes the ratio of species containing this group of conserved gene order over the corresponding family.

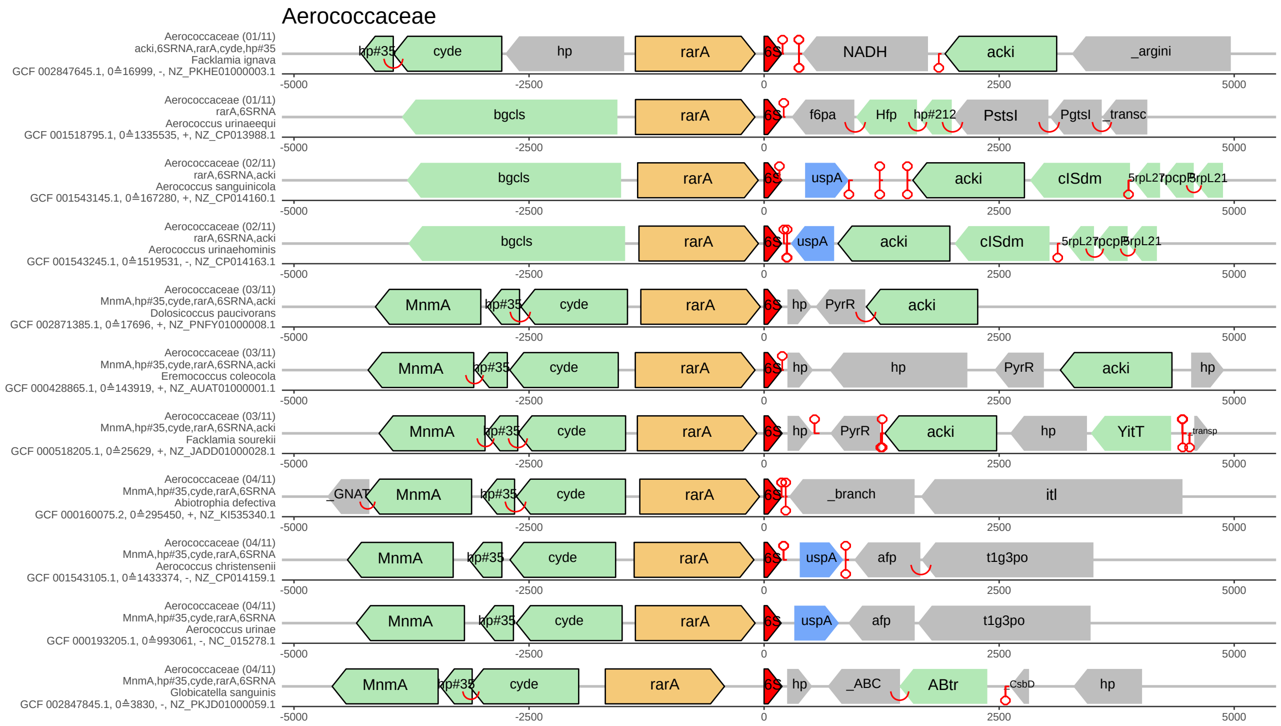
Aerococcaceae
Aerococcaceae (01/11)
acki,6SRNA,rarA,cyde,hp#35
Facklamia ignava
GCF 002847645.1, 0≙16999, -, NZ_PKHE01000003.1
hp#35
cyde
hp
rarA
NADH
acki
_argini
Aerococcaceae (01/11)
rarA,6SRNA
Aerococcus urinaeequi
GCF 001518795.1, 0≙1335535, +, NZ_CP013988.1
bgcls
rarA
f6pa
Hfp
hp#212
PstsI
PgtsI
_transc
Aerococcaceae (02/11)
rarA,6SRNA,acki
Aerococcus sanguinicola
GCF 001543145.1, 0≙167280, +, NZ_CP014160.1
bgcls
rarA
uspA
acki
cISdm
5rpL27
rpcpB
rpL21
Aerococcaceae (02/11)
rarA,6SRNA,acki
Aerococcus urinaehominis
GCF 001543245.1, 0≙1519531, -, NZ_CP014163.1
bgcls
rarA
uspA
acki
cISdm
5rpL27
rpcpB
rpL21
Aerococcaceae (03/11)
MnmA,hp#35,cyde,rarA,6SRNA,acki
Dolosicoccus paucivorans
GCF 002871385.1, 0≙17696, +, NZ_PNFY01000008.1
MnmA
hp#35
cyde
rarA
hp
PyrR
acki
Aerococcaceae (03/11)
MnmA,hp#35,cyde,rarA,6SRNA,acki
Eremococcus coleocola
GCF 000428865.1, 0≙143919, +, NZ_AUAT01000001.1
MnmA
hp#35
cyde
rarA
hp
hp
PyrR
acki
hp
Aerococcaceae (03/11)
MnmA,hp#35,cyde,rarA,6SRNA,acki
Facklamia sourekii
GCF 000518205.1, 0≙25629, +, NZ_JADD01000028.1
MnmA
hp#35
cyde
rarA
hp
PyrR
acki
hp
YitT
transp
Aerococcaceae (04/11)
MnmA,hp#35,cyde,rarA,6SRNA
Abiotrophia defectiva
GCF 000160075.2, 0≙295450, +, NZ_KI535340.1
_GNAT
MnmA
hp#35
cyde
rarA
_branch
itl
Aerococcaceae (04/11)
MnmA,hp#35,cyde,rarA,6SRNA
Aerococcus christensenii
GCF 001543105.1, 0≙1433374, -, NZ_CP014159.1
MnmA
hp#35
cyde
rarA
uspA
afp
t1g3po
Aerococcaceae (04/11)
MnmA,hp#35,cyde,rarA,6SRNA
Aerococcus urinae
GCF 000193205.1, 0≙993061, -, NC_015278.1
MnmA
hp#35
cyde
rarA
uspA
afp
t1g3po
Aerococcaceae (04/11)
MnmA,hp#35,cyde,rarA,6SRNA
Globicatella sanguinis
GCF 002847845.1, 0≙3830, -, NZ_PKJD01000059.1
MnmA
hp#35
cyde
rarA
hp
_ABC
ABtr
CsbD
hp
-5000
-2500
0
2500
5000

abbreviation	full description
5rpL21	50S ribosomal protein L21.
5rpL27	50S ribosomal protein L27.
_ABC	ABC transporter permease.
ABtr	ABC transporter.
acki	acetate kinase.
afp	aquaporin family protein.
_argini	arginine–tRNA ligase.
bgcls	bifunctional glutamate–cysteine ligase/glutathione synthetase.
_branch	branched-chain amino acid transport system II carrier protein.
cISdm	class I SAM-dependent methyltransferase.
_CsbD	CsbD family protein.
cyde	cysteine desulfurase.
f6pa	fructose-6-phosphate aldolase.
_GNAT	GNAT family N-acetyltransferase.
Hfp	HAD family phosphatase.
hp#212	hypothetical protein (orthology group #212).
hp#35	hypothetical protein (orthology group #35).
hp	hypothetical protein.
itl	isoleucine–tRNA ligase.
MnmA	tRNA 2-thiouridine(34) synthase MnmA.
NADH	NADH oxidase.
PgtsI	PTS glucitol/sorbitol transporter subunit IIC.
PstsI	PTS sorbitol transporter subunit IIB.
PyrR	bifunctional pyr operon transcriptional regulator/uracil phosphoribosyltransferase PyrR.
rarA	replication-associated recombination protein A.
rpcpP	ribosomal-processing cysteine protease Prp.
t1g3po	type 1 glycerol-3-phosphate oxidase.
_transc	transcriptional regulator.
_transp	transposase.
uspA	universal stress protein.
YitT	YitT family protein.

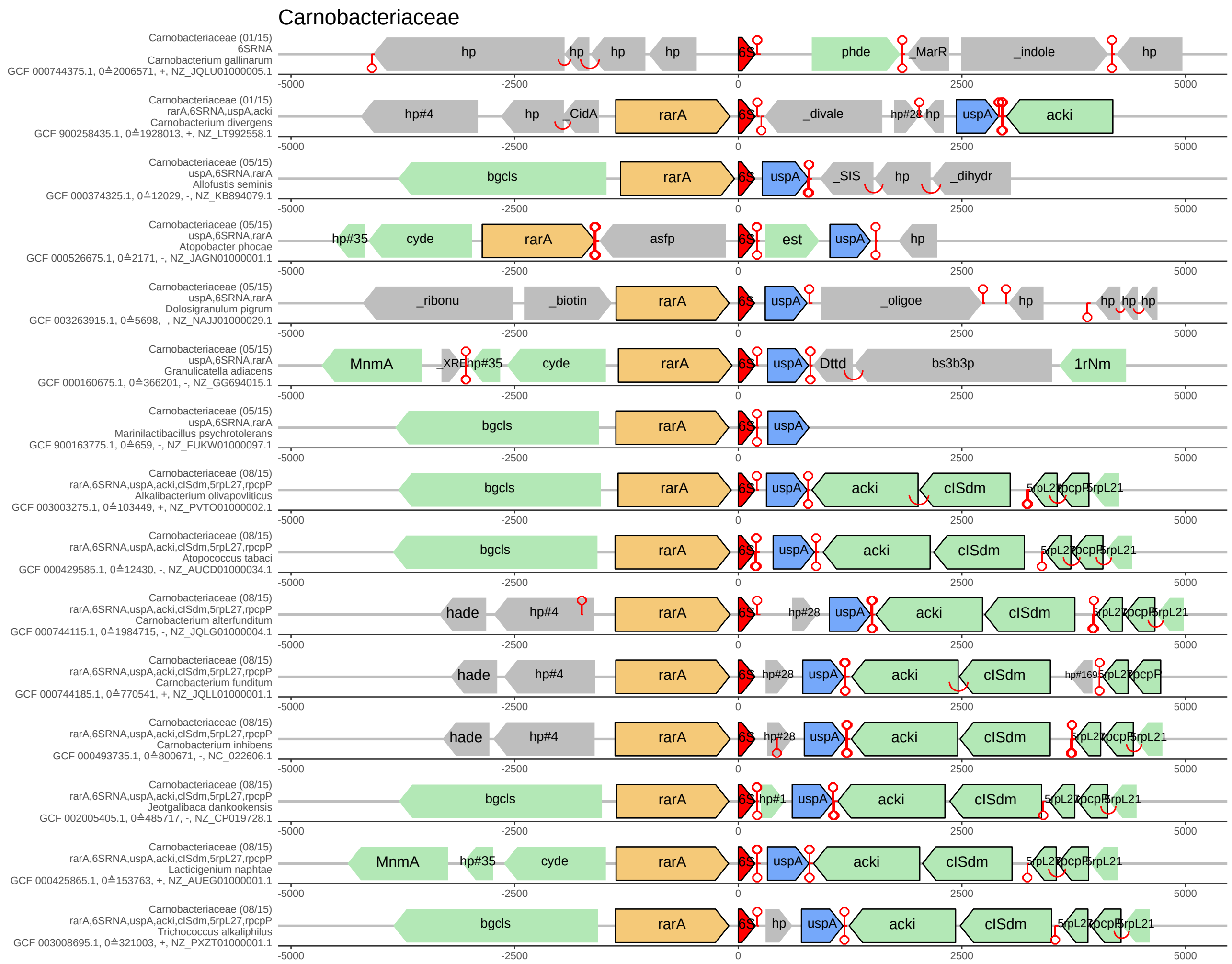
Carnobacteriaceae
Carnobacteriaceae (01/15)
6SRNA
Carnobacterium gallinarum
GCF 000744375.1, 0≙2006571, +, NZ_JQLU01000005.1
hp
hp
hp
hp
6S
phde
_MarR
_indole
hp
Carnobacteriaceae (01/15)
rarA,6SRNA,uspA,acki
Carnobacterium divergens
GCF 900258435.1, 0≙1928013, +, NZ_LT992558.1
hp#4
hp
_CidA
rarA
6S
_divale
hp#28
hp
uspA
acki
Carnobacteriaceae (05/15)
uspA,6SRNA,rarA
Allofustis seminis
GCF 000374325.1, 0≙12029, -, NZ_KB894079.1
bgcls
rarA
6S
uspA
_SIS
hp
_dihydr
Carnobacteriaceae (05/15)
uspA,6SRNA,rarA
Atopobacter phocae
GCF 000526675.1, 0≙2171, -, NZ_JAGN01000001.1
hp#35
cyde
rarA
asfp
6S
est
uspA
hp
Carnobacteriaceae (05/15)
uspA,6SRNA,rarA
Dolosigranulum pigrum
GCF 003263915.1, 0≙5698, -, NZ_NAJJ01000029.1
_ribonu
_biotin
rarA
6S
uspA
_oligoe
hp
hp
hp
hp
Carnobacteriaceae (05/15)
uspA,6SRNA,rarA
Granulicatella adiacens
GCF 000160675.1, 0≙366201, -, NZ_GG694015.1
MnmA
_XRE
hp#35
cyde
rarA
6S
uspA
Dttd
bs3b3p
1rNm
Carnobacteriaceae (05/15)
uspA,6SRNA,rarA
Marinilactibacillus psychrotolerans
GCF 900163775.1, 0≙659, -, NZ_FUKW01000097.1
bgcls
rarA
6S
uspA
Carnobacteriaceae (08/15)
rarA,6SRNA,uspA,acki,clSdm,5rpL27,rpcpP
Alkalibacterium olivapovliticus
GCF 003003275.1, 0≙103449, +, NZ_PVTO01000002.1
bgcls
rarA
6S
uspA
acki
clSdm
5rpL27
rpcpP
5rpL21
Carnobacteriaceae (08/15)
rarA,6SRNA,uspA,acki,clSdm,5rpL27,rpcpP
Atopococcus tabaci
GCF 000429585.1, 0≙12430, -, NZ_AUCD01000034.1
bgcls
rarA
6S
uspA
acki
clSdm
5rpL27
rpcpP
5rpL21
Carnobacteriaceae (08/15)
rarA,6SRNA,uspA,acki,clSdm,5rpL27,rpcpP
Carnobacterium alterfunditum
GCF 000744115.1, 0≙1984715, -, NZ_JQLG01000004.1
hade
hp#4
rarA
6S
hp#28
uspA
acki
clSdm
5rpL27
rpcpP
5rpL21
Carnobacteriaceae (08/15)
rarA,6SRNA,uspA,acki,clSdm,5rpL27,rpcpP
Carnobacterium funditum
GCF 000744185.1, 0≙770541, +, NZ_JQLL01000001.1
hade
hp#4
rarA
6S
hp#28
uspA
acki
clSdm
hp#169
5rpL27
rpcpP
Carnobacteriaceae (08/15)
rarA,6SRNA,uspA,acki,clSdm,5rpL27,rpcpP
Carnobacterium inhibens
GCF 000493735.1, 0≙800671, -, NC_022606.1
hade
hp#4
rarA
6S
hp#28
uspA
acki
clSdm
5rpL27
rpcpP
5rpL21
Carnobacteriaceae (08/15)
rarA,6SRNA,uspA,acki,clSdm,5rpL27,rpcpP
Jeotgalibaca dankookensis
GCF 002005405.1, 0≙485717, -, NZ_CP019728.1
bgcls
rarA
6S
hp#1
uspA
acki
clSdm
5rpL27
rpcpP
5rpL21
Carnobacteriaceae (08/15)
rarA,6SRNA,uspA,acki,clSdm,5rpL27,rpcpP
Lacticigenium naphtae
GCF 000425865.1, 0≙153763, +, NZ_AUEG01000001.1
MnmA
hp#35
cyde
rarA
6S
uspA
acki
clSdm
5rpL27
rpcpP
5rpL21
Carnobacteriaceae (08/15)
rarA,6SRNA,uspA,acki,clSdm,5rpL27,rpcpP
Trichococcus alkaliphilus
GCF 003008695.1, 0≙321003, +, NZ_PXZT01000001.1
bgcls
rarA
6S
hp
uspA
acki
clSdm
5rpL27
rpcpP
5rpL21
-5000
-2500
0
2500
5000

abbreviation	full description
1rNm	16S rRNA (uracil(1498)-N(3))-methyltransferase.
5rpL21	50S ribosomal protein L21.
5rpL27	50S ribosomal protein L27.
acki	acetate kinase.
asfp	alanine:cation symporter family protein.
bgcls	bifunctional glutamate–cysteine ligase/glutathione synthetase.
_biotin	biotin–[acetyl-CoA-carboxylase] ligase.
bs3b3p	bifunctional (p)ppGpp synthetase/guanosine-3',5'-bis(diphosphate) 3'-pyrophosphohydrolase.
_CidA	CidA/LrgA family protein.
cISdm	class I SAM-dependent methyltransferase.
cyde	cysteine desulfurase.
_dihydr	dihydrodipicolinate synthase family protein.
_divale	divalent metal cation transporter.
Dttd	D-tyrosyl-tRNA(Tyr) deacylase.
est	esterase.
hade	haloacid dehalogenase.
hp#169	hypothetical protein (orthology group #169).
hp#1	hypothetical protein (orthology group #1).
hp#28	hypothetical protein (orthology group #28).
hp#35	hypothetical protein (orthology group #35).
hp#4	hypothetical protein (orthology group #4).
hp	hypothetical protein.
_indole	indole-3-pyruvate decarboxylase.
_MarR	MarR family transcriptional regulator.
MnmA	tRNA 2-thiouridine(34) synthase MnmA.
_oligoe	oligoendopeptidase.
phde	phosphoglycerate dehydrogenase.
rarA	replication-associated recombination protein A.
_ribonu	ribonuclease J.
rpcpP	ribosomal-processing cysteine protease Prp.
_SIS	SIS domain-containing protein.
uspA	universal stress protein.
_XRE	XRE family transcriptional regulator.

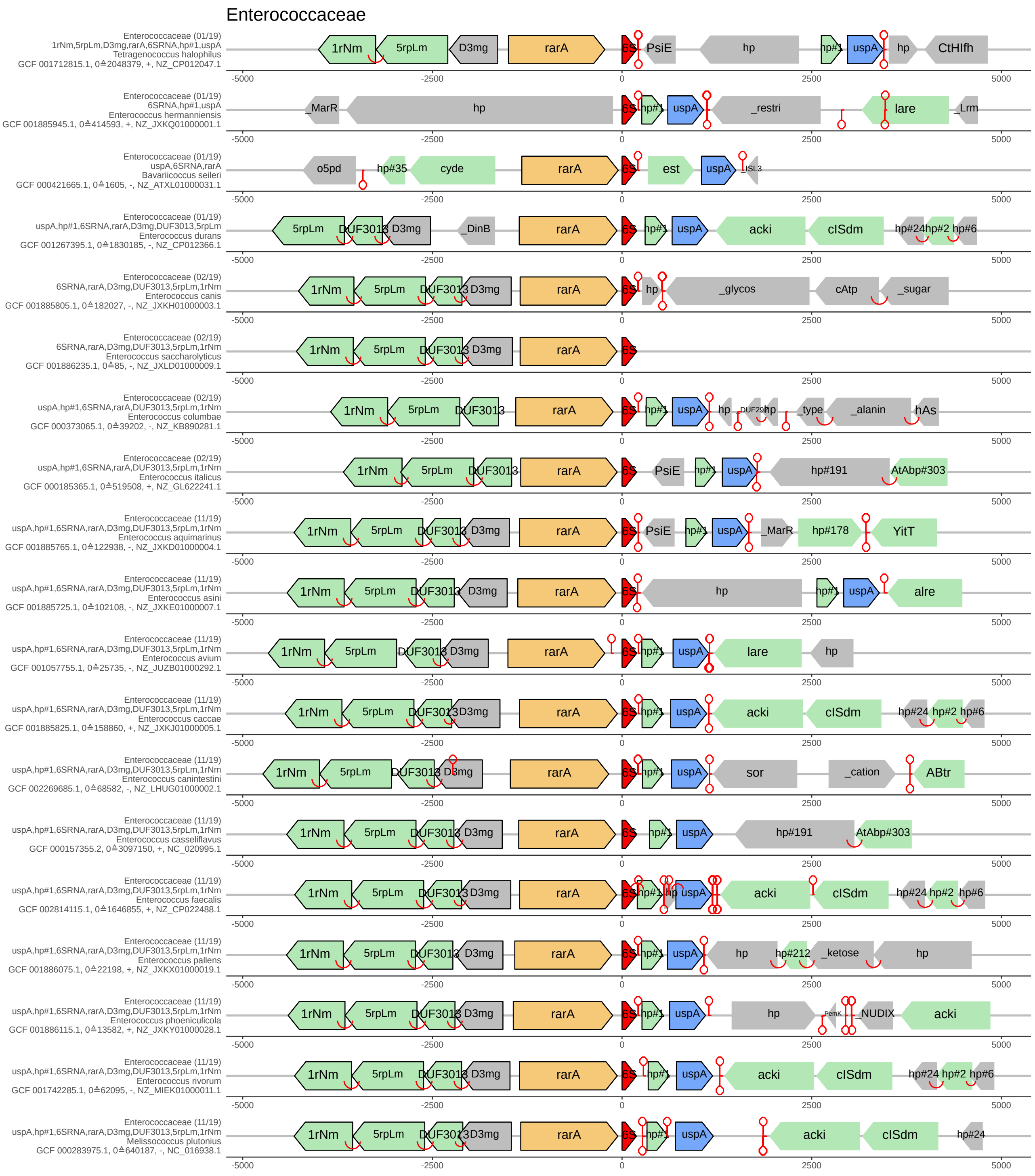
Enterococcaceae
Enterococcaceae (01/19)
1rNm,5rpLm,D3mg,rarA,6SRNA,hp#1,uspA
Tetragenococcus halophilus
GCF 001712815.1, 0≙2048379, +, NZ_CP012047.1
1rNm 5rpLm D3mg rarA 6S PsiE hp hp#1 uspA hp CtHIfh
Enterococcaceae (01/19)
6SRNA,hp#1,uspA
Enterococcus hermanniensis
GCF 001885945.1, 0≙414593, +, NZ_JXKQ01000001.1
_MarR hp 6S hp#1 uspA _restri lare _Lrm
Enterococcaceae (01/19)
uspA,6SRNA,rarA
Bavariicoccus seileri
GCF 000421665.1, 0≙1605, -, NZ_ATXL01000031.1
o5pd hp#35 cyde rarA 6S est uspA _ISL3
Enterococcaceae (01/19)
uspA,hp#1,6SRNA,rarA,D3mg,DUF3013,5rpLm
Enterococcus durans
GCF 001267395.1, 0≙1830185, -, NZ_CP012366.1
5rpLm DUF3013 D3mg _DinB rarA 6S hp#1 uspA acki clSdm hp#24 hp#2 hp#6
Enterococcaceae (02/19)
6SRNA,rarA,D3mg,DUF3013,5rpLm,1rNm
Enterococcus canis
GCF 001885805.1, 0≙182027, -, NZ_JXKH01000003.1
1rNm 5rpLm DUF3013 D3mg rarA 6S hp _glycos cAtp _sugar
Enterococcaceae (02/19)
6SRNA,rarA,D3mg,DUF3013,5rpLm,1rNm
Enterococcus saccharolyticus
GCF 001886235.1, 0≙85, -, NZ_JXLD01000009.1
1rNm 5rpLm DUF3013 D3mg rarA 6S
Enterococcaceae (02/19)
uspA,hp#1,6SRNA,rarA,DUF3013,5rpLm,1rNm
Enterococcus columbae
GCF 000373065.1, 0≙39202, -, NZ_KB890281.1
1rNm 5rpLm DUF3013 rarA 6S hp#1 uspA hp hp _type _alanin hAs
Enterococcaceae (02/19)
uspA,hp#1,6SRNA,rarA,DUF3013,5rpLm,1rNm
Enterococcus italicus
GCF 000185365.1, 0≙519508, +, NZ_GL622241.1
1rNm 5rpLm DUF3013 rarA 6S PsiE hp#1 uspA hp#191 AtAbp#303
Enterococcaceae (11/19)
uspA,hp#1,6SRNA,rarA,D3mg,DUF3013,5rpLm,1rNm
Enterococcus aquimarinus
GCF 001885765.1, 0≙122938, -, NZ_JXKD01000004.1
1rNm 5rpLm DUF3013 D3mg rarA 6S PsiE hp#1 uspA _MarR hp#178 YitT
Enterococcaceae (11/19)
uspA,hp#1,6SRNA,rarA,D3mg,DUF3013,5rpLm,1rNm
Enterococcus asini
GCF 001885725.1, 0≙102108, -, NZ_JXKE01000007.1
1rNm 5rpLm DUF3013 D3mg rarA 6S hp hp#1 uspA alre
Enterococcaceae (11/19)
uspA,hp#1,6SRNA,rarA,D3mg,DUF3013,5rpLm,1rNm
Enterococcus avium
GCF 001057755.1, 0≙25735, -, NZ_JUZB01000292.1
1rNm 5rpLm DUF3013 D3mg rarA 6S hp#1 uspA lare hp
Enterococcaceae (11/19)
uspA,hp#1,6SRNA,rarA,D3mg,DUF3013,5rpLm,1rNm
Enterococcus caccae
GCF 001885825.1, 0≙158860, +, NZ_JXKJ01000005.1
1rNm 5rpLm DUF3013 D3mg rarA 6S hp#1 uspA acki clSdm hp#24 hp#2 hp#6
Enterococcaceae (11/19)
uspA,hp#1,6SRNA,rarA,D3mg,DUF3013,5rpLm,1rNm
Enterococcus canintestini
GCF 002269685.1, 0≙68582, -, NZ_LHUG01000002.1
1rNm 5rpLm DUF3013 D3mg rarA 6S hp#1 uspA sor _cation ABtr
Enterococcaceae (11/19)
uspA,hp#1,6SRNA,rarA,D3mg,DUF3013,5rpLm,1rNm
Enterococcus casseliflavus
GCF 000157355.2, 0≙3097150, +, NC_020995.1
1rNm 5rpLm DUF3013 D3mg rarA 6S hp#1 uspA hp#191 AtAbp#303
Enterococcaceae (11/19)
uspA,hp#1,6SRNA,rarA,D3mg,DUF3013,5rpLm,1rNm
Enterococcus faecalis
GCF 002814115.1, 0≙1646855, +, NZ_CP022488.1
1rNm 5rpLm DUF3013 D3mg rarA 6S hp#1 hp uspA acki clSdm hp#24 hp#2 hp#6
Enterococcaceae (11/19)
uspA,hp#1,6SRNA,rarA,D3mg,DUF3013,5rpLm,1rNm
Enterococcus pallens
GCF 001886075.1, 0≙22198, +, NZ_JXKX01000019.1
1rNm 5rpLm DUF3013 D3mg rarA 6S hp#1 uspA hp hp#212 _ketose hp
Enterococcaceae (11/19)
uspA,hp#1,6SRNA,rarA,D3mg,DUF3013,5rpLm,1rNm
Enterococcus phoeniculicola
GCF 001886115.1, 0≙13582, +, NZ_JXKY01000028.1
1rNm 5rpLm DUF3013 D3mg rarA 6S hp#1 uspA hp _PemK _NUDIX acki
Enterococcaceae (11/19)
uspA,hp#1,6SRNA,rarA,D3mg,DUF3013,5rpLm,1rNm
Enterococcus rivorum
GCF 001742285.1, 0≙62095, -, NZ_MIEK01000011.1
1rNm 5rpLm DUF3013 D3mg rarA 6S hp#1 uspA acki clSdm hp#24 hp#2 hp#6
Enterococcaceae (11/19)
uspA,hp#1,6SRNA,rarA,D3mg,DUF3013,5rpLm,1rNm
Melissococcus plutonius
GCF 000283975.1, 0≙640187, -, NC_016938.1
1rNm 5rpLm DUF3013 D3mg rarA 6S hp#1 uspA acki clSdm hp#24
-5000 -2500 0 2500 5000

abbreviation	full description
1rNm	16S rRNA (uracil(1498)-N(3))-methyltransferase.
5rpLm	50S ribosomal protein L11 methyltransferase.
ABtr	ABC transporter.
acki	acetate kinase.
_alanin	alanine racemase.
alre	aldo/keto reductase.
AtAbp#303	ABC transporter ATP-binding protein (orthology group #303).
_cation	cation transporter.
cAtp	carbohydrate ABC transporter permease.
cISdm	class I SAM-dependent methyltransferase.
CtHIfh	Cof-type HAD-IIB family hydrolase.
cyde	cysteine desulfurase.
D3mg	DNA-3-methyladenine glycosylase.
_DinB	DinB family protein.
_DUF292	DUF2922 domain-containing protein.
DUF3013	DUF3013 domain-containing protein.
est	esterase.
_glycos	glycoside hydrolase family 127 protein.
hAs	holo-ACP synthase.
hp#178	hypothetical protein (orthology group #178).
hp#191	hypothetical protein (orthology group #191).
hp#1	hypothetical protein (orthology group #1).
hp#212	hypothetical protein (orthology group #212).
hp#24	hypothetical protein (orthology group #24).
hp#2	hypothetical protein (orthology group #2).
hp#35	hypothetical protein (orthology group #35).
hp#6	hypothetical protein (orthology group #6).
hp	hypothetical protein.
_ISL3	ISL3 family transposase.
_ketose	ketose-bisphosphate aldolase.
lare	lactaldehyde reductase.
_Lrm	L-rhamnose mutarotase.
_MarR	MarR family transcriptional regulator.
_NUDIX	NUDIX domain-containing protein.
o5pd	orotidine-5'-phosphate decarboxylase.
_PemK	PemK family transcriptional regulator.
PsiE	phosphate-starvation-inducible protein PsiE.
rarA	replication-associated recombination protein A.
_restri	restriction endonuclease.
sor	sortase.
_sugar	sugar ABC transporter permease.
_type	type II toxin-antitoxin system PemK/MazF family toxin.
uspA	universal stress protein.
YitT	YitT family protein.

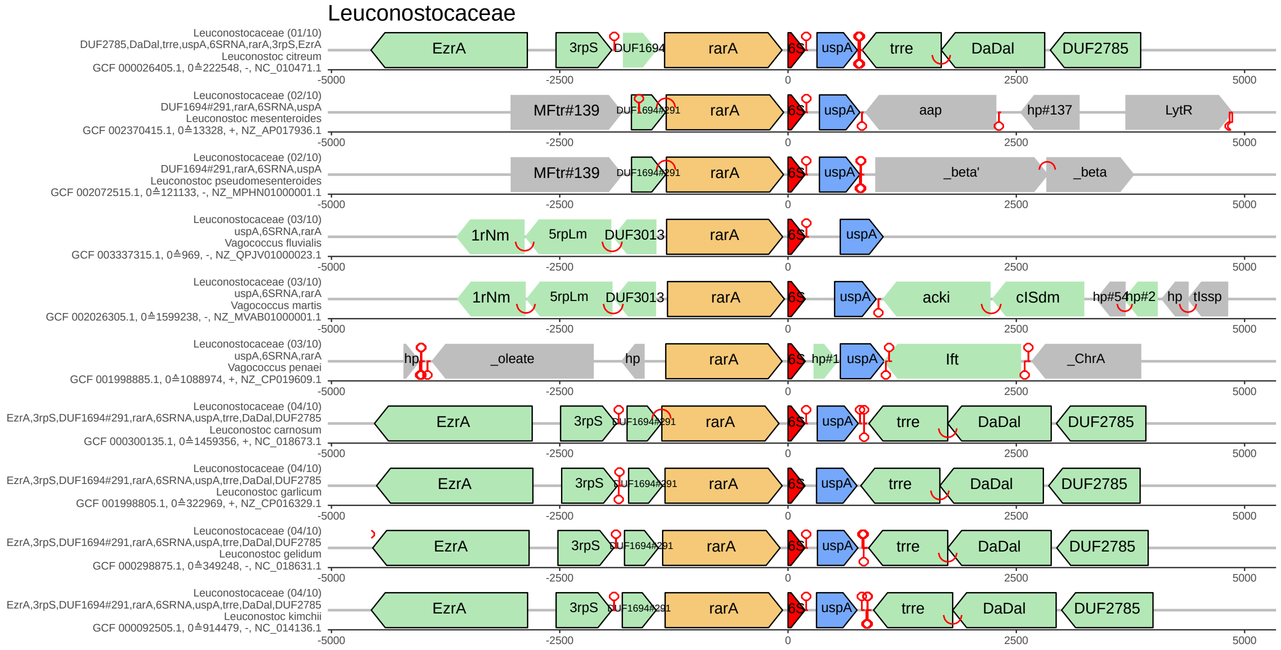

abbreviation	full description
1rNm	16S rRNA (uracil(1498)-N(3))-methyltransferase.
3rpS	30S ribosomal protein S4.
5rpLm	50S ribosomal protein L11 methyltransferase.
aap	amino acid permease.
acki	acetate kinase.
_beta'	beta-galactosidase.
_beta	beta-galactosidase small subunit.
_ChrA	ChrA protein.
cISdm	class I SAM-dependent methyltransferase.
DaDal	D-alanine–D-alanine ligase.
DUF1694#291	DUF1694 domain-containing protein (orthology group #291).
DUF1694	DUF1694 domain-containing protein.
DUF2785	DUF2785 domain-containing protein.
DUF3013	DUF3013 domain-containing protein.
EzrA	septation ring formation regulator EzrA.
hp#137	hypothetical protein (orthology group #137).
hp#1	hypothetical protein (orthology group #1).
hp#2	hypothetical protein (orthology group #2).
hp#54	hypothetical protein (orthology group #54).
hp	hypothetical protein.
Ift	IS5/IS1182 family transposase.
LytR	LytR family transcriptional regulator.
MFtr#139	MFS transporter (orthology group #139).
_oleate	oleate hydratase.
rarA	replication-associated recombination protein A.
tIssp	type II secretion system protein.
trre	transcriptional regulator.
uspA	universal stress protein.

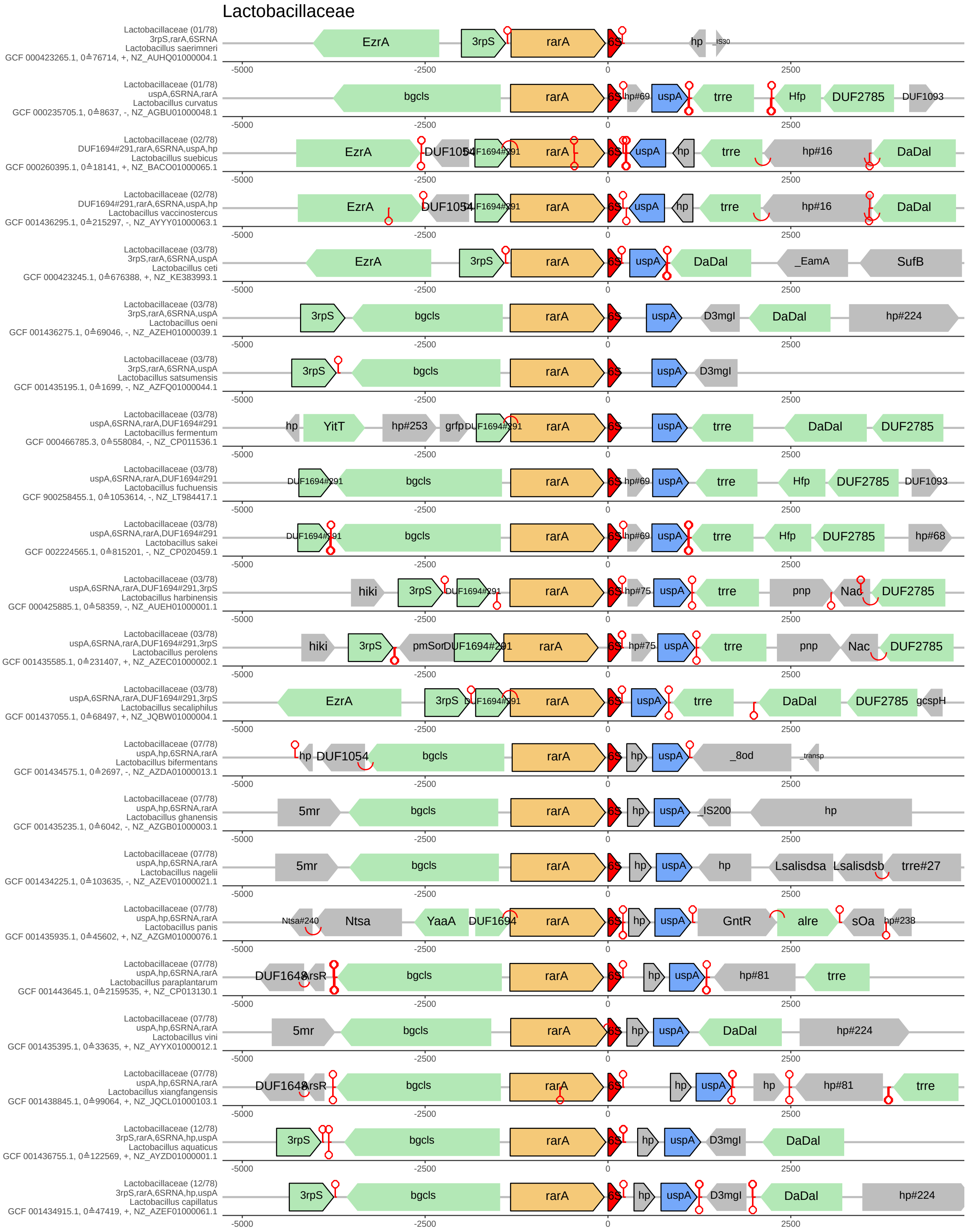
Lactobacillaceae
Lactobacillaceae (01/78)
3rpS,rarA,6SRNA
Lactobacillus saerimneri
GCF 000423265.1, 0≙76714, +, NZ_AUHQ01000004.1
EzrA 3rpS rarA 6S hp _IS30
Lactobacillaceae (01/78)
uspA,6SRNA,rarA
Lactobacillus curvatus
GCF 000235705.1, 0≙8637, -, NZ_AGBU01000048.1
bgcls rarA 6S hp#69 uspA trre Hfp DUF2785 DUF1093
Lactobacillaceae (02/78)
DUF1694#291,rarA,6SRNA,uspA,hp
Lactobacillus suebicus
GCF 000260395.1, 0≙18141, +, NZ_BACO01000065.1
EzrA DUF1054 DUF1694#291 rarA 6S uspA hp trre hp#16 DaDal
Lactobacillaceae (02/78)
DUF1694#291,rarA,6SRNA,uspA,hp
Lactobacillus vaccinostercus
GCF 001436295.1, 0≙215297, -, NZ_AYYY01000063.1
EzrA DUF1054 DUF1694#291 rarA 6S uspA hp trre hp#16 DaDal
Lactobacillaceae (03/78)
3rpS,rarA,6SRNA,uspA
Lactobacillus ceti
GCF 000423245.1, 0≙676388, +, NZ_KE383993.1
EzrA 3rpS rarA 6S uspA DaDal _EamA SufB
Lactobacillaceae (03/78)
3rpS,rarA,6SRNA,uspA
Lactobacillus oeni
GCF 001436275.1, 0≙69046, -, NZ_AZEH01000039.1
3rpS bgcls rarA 6S uspA D3mgl DaDal hp#224
Lactobacillaceae (03/78)
3rpS,rarA,6SRNA,uspA
Lactobacillus satsumensis
GCF 001435195.1, 0≙1699, -, NZ_AZFQ01000044.1
3rpS bgcls rarA 6S uspA D3mgl
Lactobacillaceae (03/78)
uspA,6SRNA,rarA,DUF1694#291
Lactobacillus fermentum
GCF 000466785.3, 0≙558084, -, NZ_CP011536.1
hp YitT hp#253 grfp DUF1694#291 rarA 6S uspA trre DaDal DUF2785
Lactobacillaceae (03/78)
uspA,6SRNA,rarA,DUF1694#291
Lactobacillus fuchuensis
GCF 900258455.1, 0≙1053614, -, NZ_LT984417.1
DUF1694#291 bgcls rarA 6S hp#69 uspA trre Hfp DUF2785 DUF1093
Lactobacillaceae (03/78)
uspA,6SRNA,rarA,DUF1694#291
Lactobacillus sakei
GCF 002224565.1, 0≙815201, -, NZ_CP020459.1
DUF1694#291 bgcls rarA 6S hp#69 uspA trre Hfp DUF2785 hp#68
Lactobacillaceae (03/78)
uspA,6SRNA,rarA,DUF1694#291,3rpS
Lactobacillus harbinensis
GCF 000425885.1, 0≙58359, -, NZ_AUEH01000001.1
hiki 3rpS DUF1694#291 rarA 6S hp#75 uspA trre pnp Nac DUF2785
Lactobacillaceae (03/78)
uspA,6SRNA,rarA,DUF1694#291,3rpS
Lactobacillus perolens
GCF 001435585.1, 0≙231407, +, NZ_AZEC01000002.1
hiki 3rpS pmSor DUF1694#291 rarA 6S hp#75 uspA trre pnp Nac DUF2785
Lactobacillaceae (03/78)
uspA,6SRNA,rarA,DUF1694#291,3rpS
Lactobacillus secaliphilus
GCF 001437055.1, 0≙68497, +, NZ_JQBW01000004.1
EzrA 3rpS DUF1694#291 rarA 6S uspA trre DaDal DUF2785 gcspH
Lactobacillaceae (07/78)
uspA,hp,6SRNA,rarA
Lactobacillus bifermentans
GCF 001434575.1, 0≙2697, -, NZ_AZDA01000013.1
hp DUF1054 bgcls rarA 6S hp uspA _8od _transp
Lactobacillaceae (07/78)
uspA,hp,6SRNA,rarA
Lactobacillus ghanensis
GCF 001435235.1, 0≙6042, -, NZ_AZGB01000003.1
5mr bgcls rarA 6S hp uspA _IS200 hp
Lactobacillaceae (07/78)
uspA,hp,6SRNA,rarA
Lactobacillus nagelii
GCF 001434225.1, 0≙103635, -, NZ_AZEV01000021.1
5mr bgcls rarA 6S hp uspA hp Lsalisdsa Lsalisdsb tre#27
Lactobacillaceae (07/78)
uspA,hp,6SRNA,rarA
Lactobacillus panis
GCF 001435935.1, 0≙45602, +, NZ_AZGM01000076.1
Ntsa#240 Ntsa YaaA DUF1694 rarA 6S hp uspA GntR alre sOa hp#238
Lactobacillaceae (07/78)
uspA,hp,6SRNA,rarA
Lactobacillus paraplantarum
GCF 001443645.1, 0≙2159535, +, NZ_CP013130.1
DUF1648 arsR bgcls rarA 6S hp uspA hp#81 trre
Lactobacillaceae (07/78)
uspA,hp,6SRNA,rarA
Lactobacillus vini
GCF 001435395.1, 0≙33635, +, NZ_AYYX01000012.1
5mr bgcls rarA 6S hp uspA DaDal hp#224
Lactobacillaceae (07/78)
uspA,hp,6SRNA,rarA
Lactobacillus xiangfangensis
GCF 001438845.1, 0≙99064, +, NZ_JQCL01000103.1
DUF1648 arsR bgcls rarA 6S hp uspA hp hp#81 trre
Lactobacillaceae (12/78)
3rpS,rarA,6SRNA,hp,uspA
Lactobacillus aquaticus
GCF 001436755.1, 0≙122569, +, NZ_AYZD01000001.1
3rpS bgcls rarA 6S hp uspA D3mgl DaDal
Lactobacillaceae (12/78)
3rpS,rarA,6SRNA,hp,uspA
Lactobacillus capillatus
GCF 001434915.1, 0≙47419, +, NZ_AZEF01000061.1
3rpS bgcls rarA 6S hp uspA D3mgl DaDal hp#224
-5000 -2500 0 2500

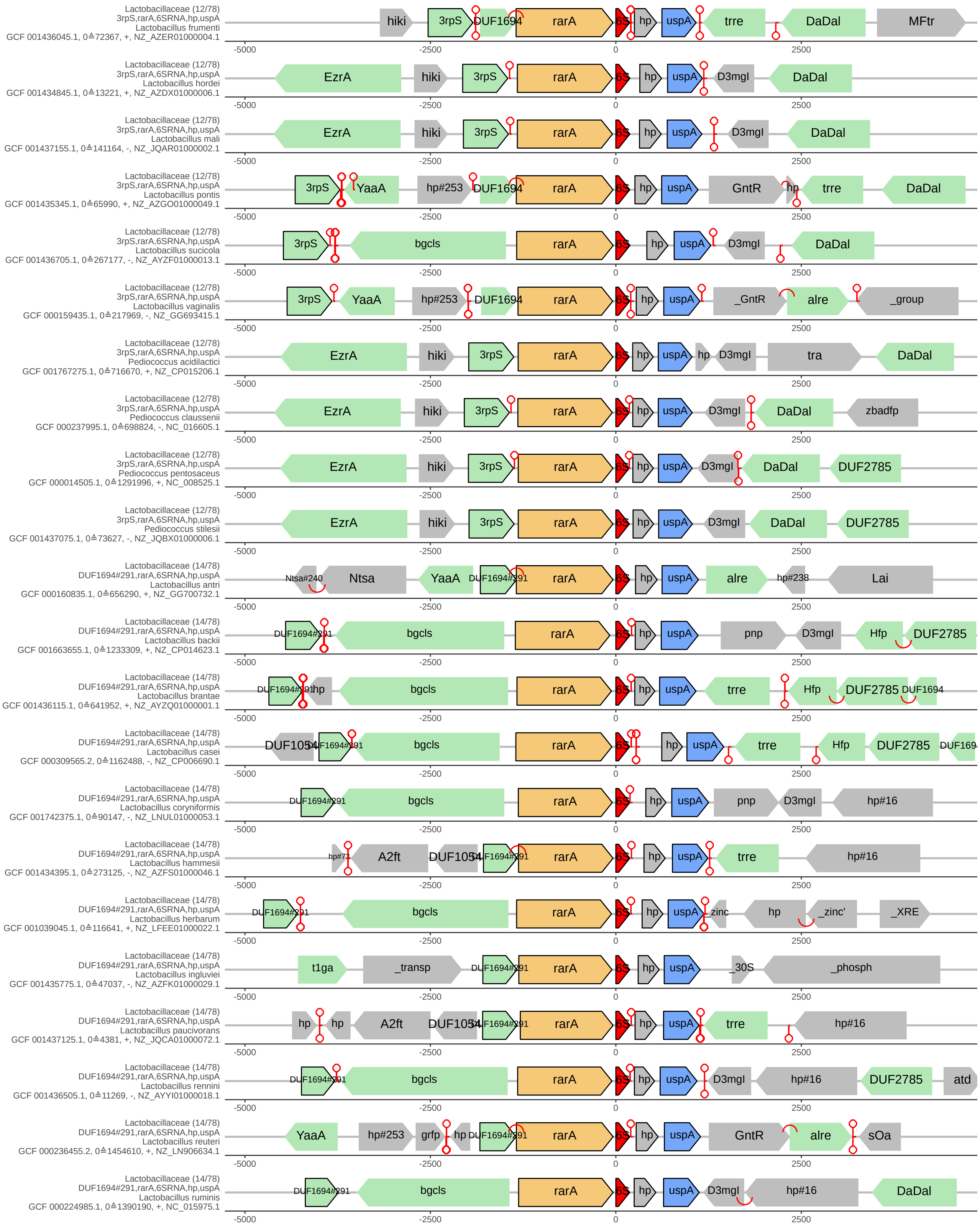

Lactobacillaceae (12/78)
3rpS,rarA,6SRNA,hp,uspA
Lactobacillus frumenti
GCF 001436045.1, 0≙72367, +, NZ_AZER01000004.1
hiki
3rpS
DUF1694
rarA
6S
hp
uspA
trre
DaDal
MFtr
Lactobacillaceae (12/78)
3rpS,rarA,6SRNA,hp,uspA
Lactobacillus hordei
GCF 001434845.1, 0≙13221, +, NZ_AZDX01000006.1
EzrA
hiki
3rpS
rarA
6S
hp
uspA
D3mgl
DaDal
Lactobacillaceae (12/78)
3rpS,rarA,6SRNA,hp,uspA
Lactobacillus mali
GCF 001437155.1, 0≙141164, -, NZ_JQAR01000002.1
EzrA
hiki
3rpS
rarA
6S
hp
uspA
D3mgl
DaDal
Lactobacillaceae (12/78)
3rpS,rarA,6SRNA,hp,uspA
Lactobacillus pontis
GCF 001435345.1, 0≙65990, +, NZ_AZGO01000049.1
3rpS
YaaA
hp#253
DUF1694
rarA
6S
hp
uspA
GntR
hp
trre
DaDal
Lactobacillaceae (12/78)
3rpS,rarA,6SRNA,hp,uspA
Lactobacillus sucicola
GCF 001436705.1, 0≙267177, -, NZ_AYZF01000013.1
3rpS
bgcls
rarA
6S
hp
uspA
D3mgl
DaDal
Lactobacillaceae (12/78)
3rpS,rarA,6SRNA,hp,uspA
Lactobacillus vaginalis
GCF 000159435.1, 0≙217969, -, NZ_GG693415.1
3rpS
YaaA
hp#253
DUF1694
rarA
6S
hp
uspA
_GntR
alre
_group
Lactobacillaceae (12/78)
3rpS,rarA,6SRNA,hp,uspA
Pediococcus acidilactici
GCF 001767275.1, 0≙716670, +, NZ_CP015206.1
EzrA
hiki
3rpS
rarA
6S
hp
uspA
hp
D3mgl
tra
DaDal
Lactobacillaceae (12/78)
3rpS,rarA,6SRNA,hp,uspA
Pediococcus claussenii
GCF 000237995.1, 0≙698824, -, NC_016605.1
EzrA
hiki
3rpS
rarA
6S
hp
uspA
D3mgl
DaDal
zbadfp
Lactobacillaceae (12/78)
3rpS,rarA,6SRNA,hp,uspA
Pediococcus pentosaceus
GCF 000014505.1, 0≙1291996, +, NC_008525.1
EzrA
hiki
3rpS
rarA
6S
hp
uspA
D3mgl
DaDal
DUF2785
Lactobacillaceae (12/78)
3rpS,rarA,6SRNA,hp,uspA
Pediococcus stilesii
GCF 001437075.1, 0≙73627, -, NZ_JQBX01000006.1
EzrA
hiki
3rpS
rarA
6S
hp
uspA
D3mgl
DaDal
DUF2785
Lactobacillaceae (14/78)
DUF1694#291,rarA,6SRNA,hp,uspA
Lactobacillus antri
GCF 000160835.1, 0≙656290, +, NZ_GG700732.1
Ntsa#240
Ntsa
YaaA
DUF1694#291
rarA
6S
hp
uspA
alre
hp#238
Lai
Lactobacillaceae (14/78)
DUF1694#291,rarA,6SRNA,hp,uspA
Lactobacillus backii
GCF 001663655.1, 0≙1233309, +, NZ_CP014623.1
DUF1694#291
bgcls
rarA
6S
hp
uspA
pnp
D3mgl
Hfp
DUF2785
Lactobacillaceae (14/78)
DUF1694#291,rarA,6SRNA,hp,uspA
Lactobacillus brantae
GCF 001436115.1, 0≙641952, +, NZ_AYZQ01000001.1
DUF1694#291
hp
bgcls
rarA
6S
hp
uspA
trre
Hfp
DUF2785
DUF1694
Lactobacillaceae (14/78)
DUF1694#291,rarA,6SRNA,hp,uspA
Lactobacillus casei
GCF 000309565.2, 0≙1162488, -, NZ_CP006690.1
DUF1054
DUF1694#291
bgcls
rarA
6S
hp
uspA
trre
Hfp
DUF2785
Lactobacillaceae (14/78)
DUF1694#291,rarA,6SRNA,hp,uspA
Lactobacillus coryniformis
GCF 001742375.1, 0≙90147, -, NZ_LNUL01000053.1
DUF1694#291
bgcls
rarA
6S
hp
uspA
pnp
D3mgl
hp#16
Lactobacillaceae (14/78)
DUF1694#291,rarA,6SRNA,hp,uspA
Lactobacillus hammesii
GCF 001434395.1, 0≙273125, -, NZ_AZFS01000046.1
hp#73
A2ft
DUF1054
DUF1694#291
rarA
6S
hp
uspA
trre
hp#16
Lactobacillaceae (14/78)
DUF1694#291,rarA,6SRNA,hp,uspA
Lactobacillus herbarum
GCF 001039045.1, 0≙116641, +, NZ_LFEE01000022.1
DUF1694#291
bgcls
rarA
6S
hp
uspA
_zinc
hp
_zinc'
_XRE
Lactobacillaceae (14/78)
DUF1694#291,rarA,6SRNA,hp,uspA
Lactobacillus ingluviei
GCF 001435775.1, 0≙47037, -, NZ_AZFK01000029.1
t1ga
_transp
DUF1694#291
rarA
6S
hp
uspA
_30S
_phosph
Lactobacillaceae (14/78)
DUF1694#291,rarA,6SRNA,hp,uspA
Lactobacillus paucivorans
GCF 001437125.1, 0≙4381, +, NZ_JQCA01000072.1
hp
hp
A2ft
DUF1054
DUF1694#291
rarA
6S
hp
uspA
trre
hp#16
Lactobacillaceae (14/78)
DUF1694#291,rarA,6SRNA,hp,uspA
Lactobacillus rennini
GCF 001436505.1, 0≙11269, -, NZ_AYYI01000018.1
DUF1694#291
bgcls
rarA
6S
hp
uspA
D3mgl
hp#16
DUF2785
atd
Lactobacillaceae (14/78)
DUF1694#291,rarA,6SRNA,hp,uspA
Lactobacillus reuteri
GCF 000236455.2, 0≙1454610, +, NZ_LN906634.1
YaaA
hp#253
grfp
hp
DUF1694#291
rarA
6S
hp
uspA
GntR
alre
sOa
Lactobacillaceae (14/78)
DUF1694#291,rarA,6SRNA,hp,uspA
Lactobacillus ruminis
GCF 000224985.1, 0≙1390190, +, NC_015975.1
DUF1694#291
bgcls
rarA
6S
hp
uspA
D3mgl
hp#16
DaDal
-5000
-2500
0
2500

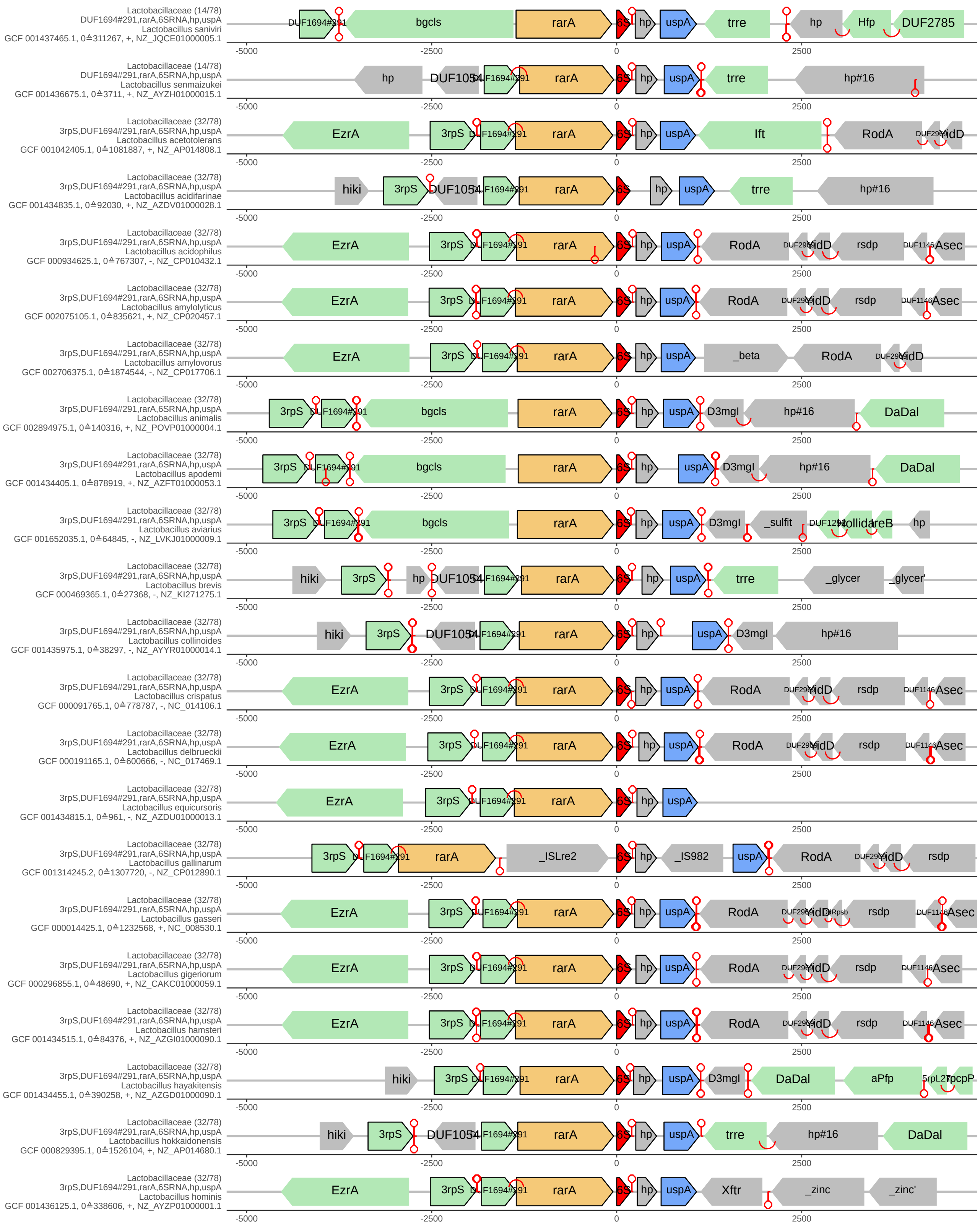

Lactobacillaceae (14/78)
DUF1694#291,rarA,6SRNA,hp,uspA
Lactobacillus saniviri
GCF 001437465.1, 0≙311267, +, NZ_JQCE01000005.1
Lactobacillaceae (14/78)
DUF1694#291,rarA,6SRNA,hp,uspA
Lactobacillus senmaizukei
GCF 001436675.1, 0≙3711, +, NZ_AYZH01000015.1
Lactobacillus acetotolerans
GCF 001042405.1, 0≙1081887, +, NZ_AP014808.1
Lactobacillus acidifarinae
GCF 001434835.1, 0≙92030, +, NZ_AZDV01000028.1
Lactobacillus acidophilus
GCF 000934625.1, 0≙767307, -, NZ_CP010432.1
Lactobacillus amylolyticus
GCF 002075105.1, 0≙835621, +, NZ_CP020457.1
Lactobacillus amylovorus
GCF 002706375.1, 0≙1874544, -, NZ_CP017706.1
Lactobacillus animalis
GCF 002894975.1, 0≙140316, +, NZ_POVP01000004.1
Lactobacillus apodemi
GCF 001434405.1, 0≙878919, +, NZ_AZFT01000053.1
Lactobacillus aviarius
GCF 001652035.1, 0≙64845, -, NZ_LVKJ01000009.1
Lactobacillus brevis
GCF 000469365.1, 0≙27368, -, NZ_KI271275.1
Lactobacillus collinoides
GCF 001435975.1, 0≙38297, -, NZ_AYYR01000014.1
Lactobacillus crispatus
GCF 000091765.1, 0≙778787, -, NC_014106.1
Lactobacillus delbrueckii
GCF 000191165.1, 0≙600666, -, NC_017469.1
Lactobacillus equicursoris
GCF 001434815.1, 0≙961, -, NZ_AZDU01000013.1
Lactobacillus gallinarum
GCF 001314245.2, 0≙1307720, -, NZ_CP012890.1
Lactobacillus gasseri
GCF 000014425.1, 0≙1232568, +, NC_008530.1
Lactobacillus gigeriorum
GCF 000296855.1, 0≙48690, +, NZ_CAKC01000059.1
Lactobacillus hamsteri
GCF 001434515.1, 0≙84376, +, NZ_AZGI01000090.1
Lactobacillus hayakitensis
GCF 001434455.1, 0≙390258, +, NZ_AZGD01000090.1
Lactobacillus hokkaidonensis
GCF 000829395.1, 0≙1526104, +, NZ_AP014680.1
Lactobacillus hominis
GCF 001436125.1, 0≙338606, +, NZ_AYZP01000001.1
Lactobacillaceae (32/78)
3rpS,DUF1694#291,rarA,6SRNA,hp,uspA
-5000
-2500
0
2500

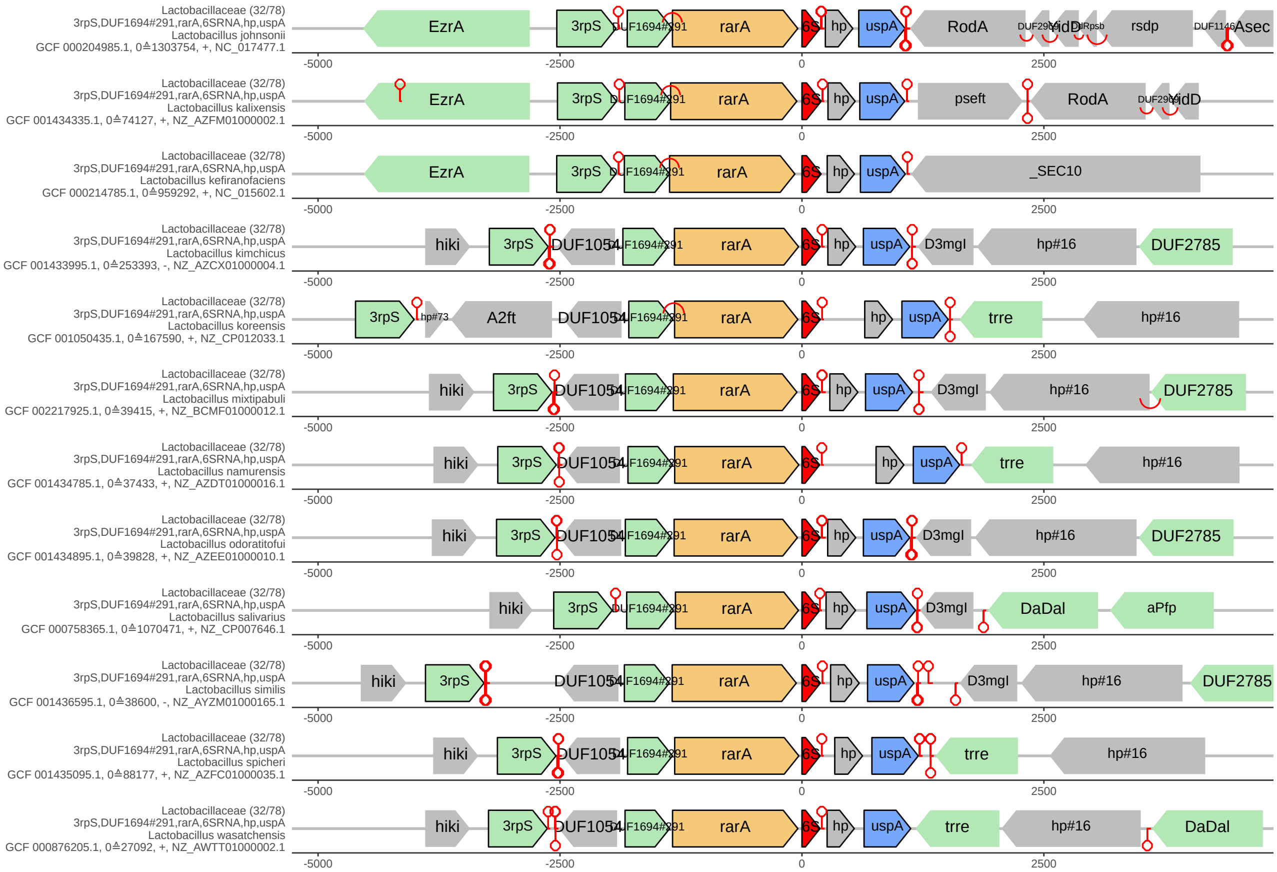

Lactobacillaceae (32/78)
3rpS,DUF1694#291,rarA,6SRNA,hp,uspA
Lactobacillus johnsonii
GCF 000204985.1, 0≙1303754, +, NC_017477.1
EzrA
3rpS
DUF1694#291
rarA
6S
hp
uspA
RodA
DUF29
YidD
rsdp
DUF1146
Asec
Lactobacillaceae (32/78)
3rpS,DUF1694#291,rarA,6SRNA,hp,uspA
Lactobacillus kalixensis
GCF 001434335.1, 0≙74127, +, NZ_AZFM01000002.1
EzrA
3rpS
DUF1694#291
rarA
6S
hp
uspA
pseft
RodA
YidD
Lactobacillaceae (32/78)
3rpS,DUF1694#291,rarA,6SRNA,hp,uspA
Lactobacillus kefiranofaciens
GCF 000214785.1, 0≙959292, +, NC_015602.1
EzrA
3rpS
DUF1694#291
rarA
6S
hp
uspA
_SEC10
Lactobacillaceae (32/78)
3rpS,DUF1694#291,rarA,6SRNA,hp,uspA
Lactobacillus kimchicus
GCF 001433995.1, 0≙253393, -, NZ_AZCX01000004.1
hiki
3rpS
DUF1054
DUF1694#291
rarA
6S
hp
uspA
D3mgl
hp#16
DUF2785
Lactobacillaceae (32/78)
3rpS,DUF1694#291,rarA,6SRNA,hp,uspA
Lactobacillus koreensis
GCF 001050435.1, 0≙167590, +, NZ_CP012033.1
3rpS
hp#73
A2ft
DUF1054
DUF1694#291
rarA
6S
hp
uspA
trre
hp#16
Lactobacillaceae (32/78)
3rpS,DUF1694#291,rarA,6SRNA,hp,uspA
Lactobacillus mixtipabuli
GCF 002217925.1, 0≙39415, +, NZ_BCMF01000012.1
hiki
3rpS
DUF1054
DUF1694#291
rarA
6S
hp
uspA
D3mgl
hp#16
DUF2785
Lactobacillaceae (32/78)
3rpS,DUF1694#291,rarA,6SRNA,hp,uspA
Lactobacillus namurensis
GCF 001434785.1, 0≙37433, +, NZ_AZDT01000016.1
hiki
3rpS
DUF1054
DUF1694#291
rarA
6S
hp
uspA
trre
hp#16
Lactobacillaceae (32/78)
3rpS,DUF1694#291,rarA,6SRNA,hp,uspA
Lactobacillus odoratitofui
GCF 001434895.1, 0≙39828, +, NZ_AZEE01000010.1
hiki
3rpS
DUF1054
DUF1694#291
rarA
6S
hp
uspA
D3mgl
hp#16
DUF2785
Lactobacillaceae (32/78)
3rpS,DUF1694#291,rarA,6SRNA,hp,uspA
Lactobacillus salivarius
GCF 000758365.1, 0≙1070471, +, NZ_CP007646.1
hiki
3rpS
DUF1694#291
rarA
6S
hp
uspA
D3mgl
DaDal
aPfp
Lactobacillaceae (32/78)
3rpS,DUF1694#291,rarA,6SRNA,hp,uspA
Lactobacillus similis
GCF 001436595.1, 0≙38600, -, NZ_AYZM01000165.1
hiki
3rpS
DUF1054
DUF1694#291
rarA
6S
hp
uspA
D3mgl
hp#16
DUF2785
Lactobacillaceae (32/78)
3rpS,DUF1694#291,rarA,6SRNA,hp,uspA
Lactobacillus spicheri
GCF 001435095.1, 0≙88177, +, NZ_AZFC01000035.1
hiki
3rpS
DUF1054
DUF1694#291
rarA
6S
hp
uspA
trre
hp#16
Lactobacillaceae (32/78)
3rpS,DUF1694#291,rarA,6SRNA,hp,uspA
Lactobacillus wasatchensis
GCF 000876205.1, 0≙27092, +, NZ_AWTT01000002.1
hiki
3rpS
DUF1054
DUF1694#291
rarA
6S
hp
uspA
trre
hp#16
DaDal
-5000
-2500
0
2500

abbreviation	full description
_30S	30S ribosomal protein S14.
_30S	30S ribosomal protein S14.
3rpS	30S ribosomal protein S4.
5mr	5,10-methylenetetrahydrofolate reductase.
5rpL27	50S ribosomal protein L27.
_8od	8-oxoguanine deaminase.
A2ft	AI-2E family transporter.
alre	aldo/keto reductase.
aPfp	aminopeptidase P family protein.
ArsR	ArsR family transcriptional regulator.
Asec	ATP synthase epsilon chain.
atd	aminoacyl-tRNA deacylase.
_beta	beta-N-acetylhexosaminidase.
bgcls	bifunctional glutamate–cysteine ligase/glutathione synthetase.
D3mgI	DNA-3-methyladenine glycosylase I.
DaDal	D-alanine–D-alanine ligase.
DdRpsb	DNA-directed RNA polymerase subunit beta.
DUF1054	DUF1054 domain-containing protein.
DUF1093	DUF1093 domain-containing protein.
DUF1146	DUF1146 domain-containing protein.
DUF1292	DUF1292 domain-containing protein.
DUF1648	DUF1648 domain-containing protein.
DUF1694#291	DUF1694 domain-containing protein (orthology group #291).
DUF1694	DUF1694 domain-containing protein.
DUF2785	DUF2785 domain-containing protein.
DUF2969	DUF2969 domain-containing protein.
_EamA	EamA family transporter.
EzrA	septation ring formation regulator EzrA.
gcspH	glycine cleavage system protein H.
_glycer	glycerophosphodiester phosphodiesterase.
_glycer'	glycerophosphodiester phosphodiesterase family protein.
_GntR	GntR family transcriptional regulator.
GntR	GntR family transcriptional regulator.
grfp	glyoxalase/bleomycin resistance/dioxygenase family protein.
_group	group II intron reverse transcriptase/maturase.
Hfp	HAD family phosphatase.
hiki	histidine kinase.
Hollida	Holliday junction resolvase RuvX.
hp#16	hypothetical protein (orthology group #16).
hp#224	hypothetical protein (orthology group #224).
hp#238	hypothetical protein (orthology group #238).
hp#253	hypothetical protein (orthology group #253).
hp#68	hypothetical protein (orthology group #68).
hp#69	hypothetical protein (orthology group #69).
hp#73	hypothetical protein (orthology group #73).
hp#75	hypothetical protein (orthology group #75).
hp#81	hypothetical protein (orthology group #81).
hp	hypothetical protein.
Ift	IS5/IS1182 family transposase.
IreB	IreB family regulatory phosphoprotein.
_IS200	IS200/IS605 family transposase.
_IS30	IS30 family transposase.
_IS982	IS982 family transposase.

_ISLre2	ISLre2 family transposase.
Lai	L-arabinose isomerase.
Lsalisdsa	L-serine ammonia-lyase, iron-sulfur-dependent, subunit alpha.
Lsalisdsb	L-serine ammonia-lyase, iron-sulfur-dependent, subunit beta.
MFtr	MFS transporter.
Nac	N-acetyltransferase.
Ntsa#240	NAD(P) transhydrogenase subunit alpha (orthology group #240).
Ntsa	NAD(P) transhydrogenase subunit alpha.
_phosph	phosphoenolpyruvate synthase.
pmSor	peptide-methionine (S)-S-oxide reductase.
pnp	phosphate–nucleotide phosphotransferase.
pseft	putative sulfate exporter family transporter.
rarA	replication-associated recombination protein A.
RodA	rod shape-determining protein RodA.
rpcpP	ribosomal-processing cysteine protease Prp.
rsdp	rod shape-determining protein.
_SEC10	SEC10/PgrA surface exclusion domain-containing protein.
sOa	sugar O-acetyltransferase.
SufB	Fe-S cluster assembly protein SufB.
_sulfit	sulfite exporter TauE/SafE family protein.
t1ga	type 1 glutamine amidotransferase.
_transp	transposase.
tra	transposase.
trre#27	transcriptional regulator (orthology group #27).
trre	transcriptional regulator.
uspA	universal stress protein.
Xftr	XRE family transcriptional regulator.
_XRE	XRE family transcriptional regulator.
YaaA	peroxide stress protein YaaA.
YidD	membrane protein insertion efficiency factor YidD.
YitT	YitT family protein.
zbadfp	zinc-binding alcohol dehydrogenase family protein.
_zinc	zinc ribbon domain-containing protein.
_zinc'	zinc ribbon domain-containing protein.

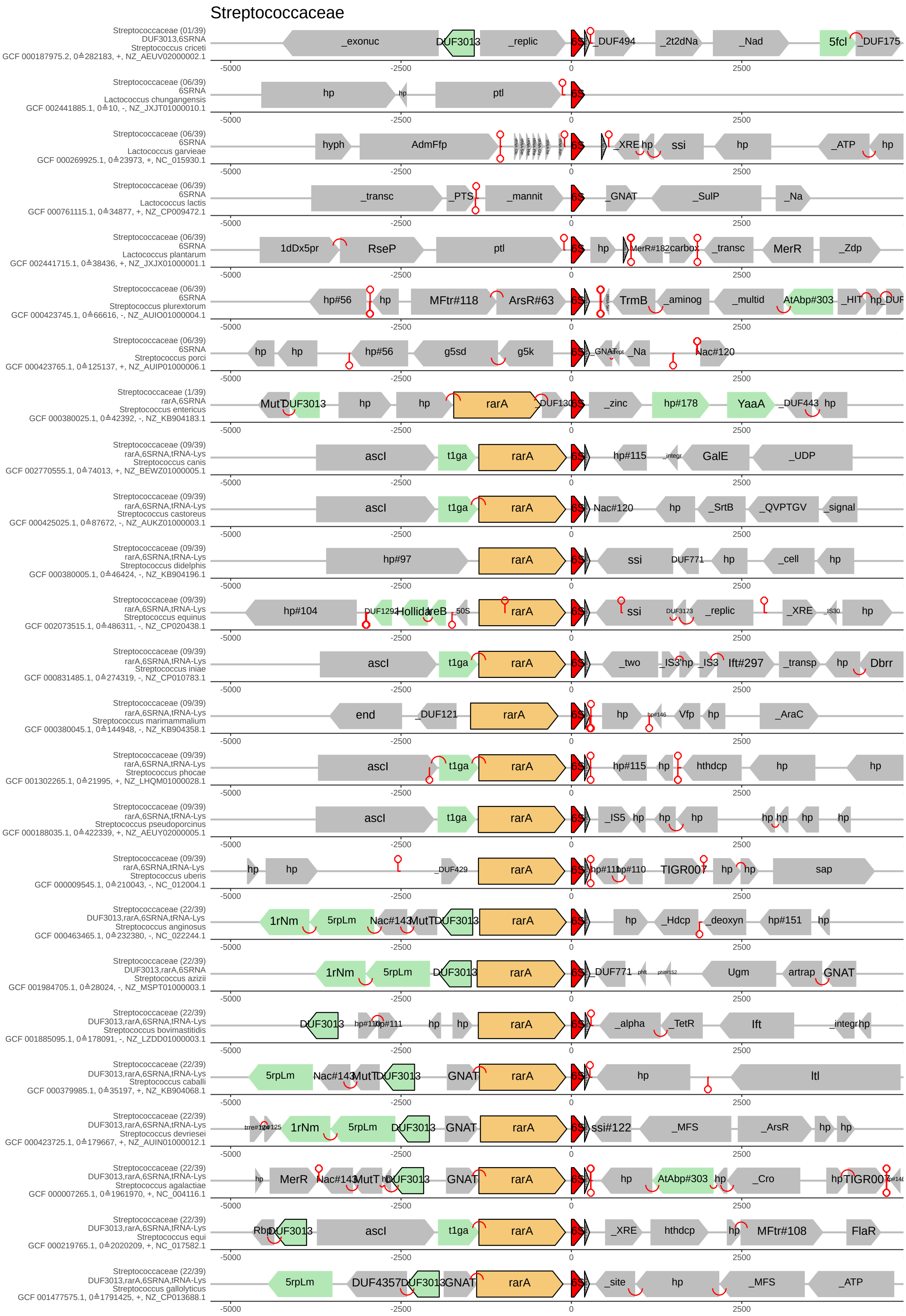
Streptococcaceae
Streptococcaceae (01/39) DUF3013,6SRNA Streptococcus criceti GCF 000187975.2, 0≙282183, +, NZ_AEUV02000002.1
Streptococcaceae (06/39) 6SRNA Lactococcus chungangensis GCF 002441885.1, 0≙10, -, NZ_JXJT01000010.1
Streptococcaceae (06/39) 6SRNA Lactococcus garvieae GCF 000269925.1, 0≙23973, +, NC_015930.1
Streptococcaceae (06/39) 6SRNA Lactococcus lactis GCF 000761115.1, 0≙34877, +, NZ_CP009472.1
Streptococcaceae (06/39) 6SRNA Lactococcus plantarum GCF 002441715.1, 0≙38436, +, NZ_JXJX01000001.1
Streptococcaceae (06/39) 6SRNA Streptococcus plurextorum GCF 000423745.1, 0≙66616, -, NZ_AUIO01000004.1
Streptococcaceae (06/39) 6SRNA Streptococcus porci GCF 000423765.1, 0≙125137, +, NZ_AUIP01000006.1
Streptococcaceae (1/39) rarA,6SRNA Streptococcus entericus GCF 000380025.1, 0≙42392, -, NZ_KB904183.1
Streptococcaceae (09/39) rarA,6SRNA,tRNA-Lys Streptococcus canis GCF 002770555.1, 0≙74013, +, NZ_BEWZ01000005.1
Streptococcaceae (09/39) rarA,6SRNA,tRNA-Lys Streptococcus castoreus GCF 000425025.1, 0≙87672, -, NZ_AUKZ01000003.1
Streptococcaceae (09/39) rarA,6SRNA,tRNA-Lys Streptococcus didelphis GCF 000380005.1, 0≙46424, -, NZ_KB904196.1
Streptococcaceae (09/39) rarA,6SRNA,tRNA-Lys Streptococcus equinus GCF 002073515.1, 0≙486311, -, NZ_CP020438.1
Streptococcaceae (09/39) rarA,6SRNA,tRNA-Lys Streptococcus iniae GCF 000831485.1, 0≙274319, -, NZ_CP010783.1
Streptococcaceae (09/39) rarA,6SRNA,tRNA-Lys Streptococcus marimammalium GCF 000380045.1, 0≙144948, -, NZ_KB904358.1
Streptococcaceae (09/39) rarA,6SRNA,tRNA-Lys Streptococcus phocae GCF 001302265.1, 0≙21995, +, NZ_LHQM01000028.1
Streptococcaceae (09/39) rarA,6SRNA,tRNA-Lys Streptococcus pseudoporcinus GCF 000188035.1, 0≙422339, +, NZ_AEUY02000005.1
Streptococcaceae (09/39) rarA,6SRNA,tRNA-Lys Streptococcus uberis GCF 000009545.1, 0≙210043, -, NC_012004.1
Streptococcaceae (22/39) DUF3013,rarA,6SRNA,tRNA-Lys Streptococcus anginosus GCF 000463465.1, 0≙232380, -, NC_022244.1
Streptococcaceae (22/39) DUF3013,rarA,6SRNA Streptococcus azizii GCF 001984705.1, 0≙28024, -, NZ_MSPT01000003.1
Streptococcaceae (22/39) DUF3013,rarA,6SRNA,tRNA-Lys Streptococcus bovimastitidis GCF 001885095.1, 0≙178091, -, NZ_LZDD01000003.1
Streptococcaceae (22/39) DUF3013,rarA,6SRNA,tRNA-Lys Streptococcus caballi GCF 000379985.1, 0≙35197, +, NZ_KB904068.1
Streptococcaceae (22/39) DUF3013,rarA,6SRNA,tRNA-Lys Streptococcus devriesei GCF 000423725.1, 0≙179667, +, NZ_AUIN01000012.1
Streptococcaceae (22/39) DUF3013,rarA,6SRNA,tRNA-Lys Streptococcus agalactiae GCF 000007265.1, 0≙1961970, +, NC_004116.1
Streptococcaceae (22/39) DUF3013,rarA,6SRNA,tRNA-Lys Streptococcus equi GCF 000219765.1, 0≙2020209, +, NC_017582.1
Streptococcaceae (22/39) DUF3013,rarA,6SRNA,tRNA-Lys Streptococcus gallolyticus GCF 001477575.1, 0≙1791425, +, NZ_CP013688.1
-5000
-2500
0
2500

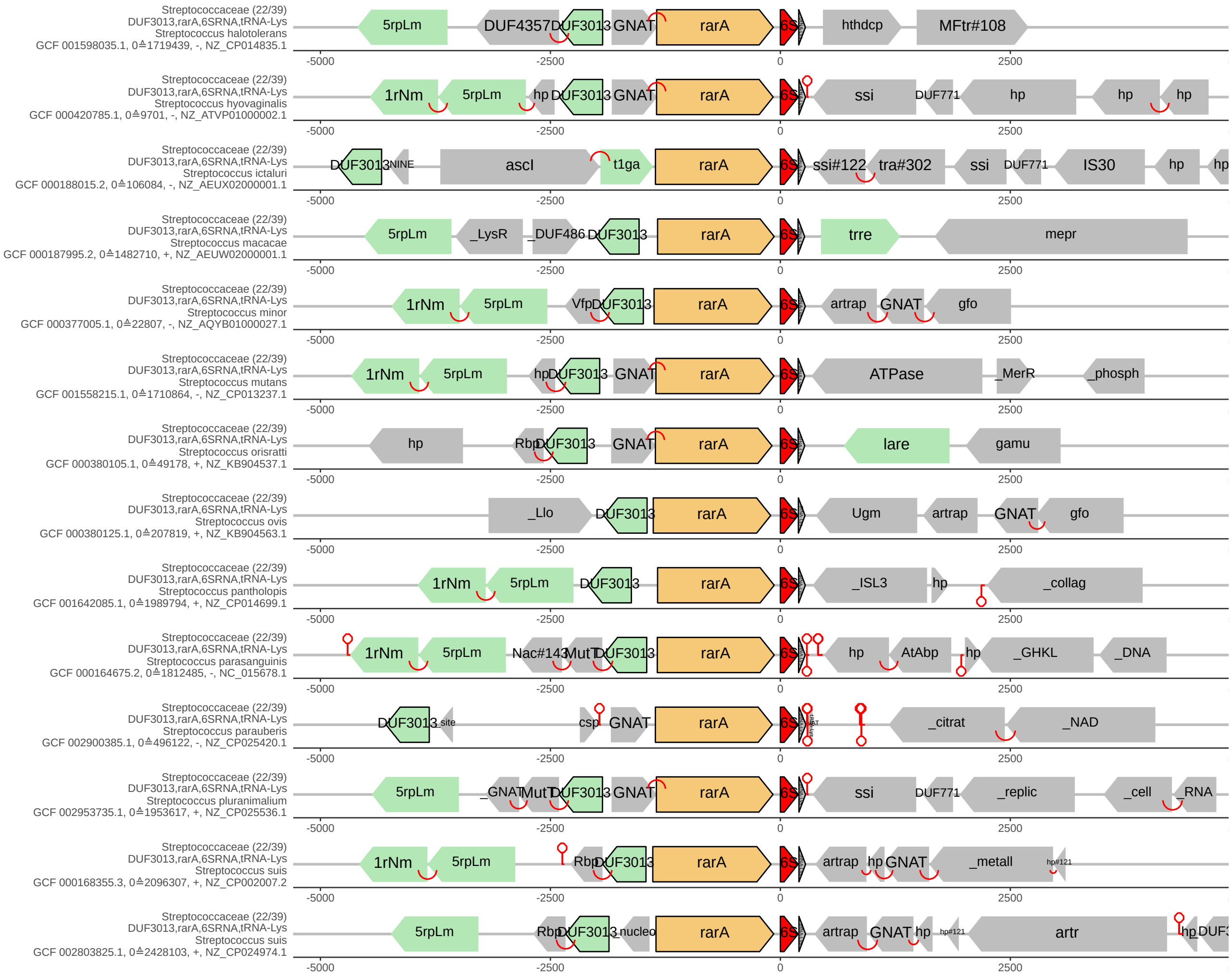
Streptococcaceae (22/39)
DUF3013,rarA,6SRNA,tRNA-Lys
Streptococcus halotolerans
GCF 001598035.1, 0≙1719439, -, NZ_CP014835.1
5rpLm
DUF4357
DUF3013
GNAT
rarA
hthdcp
MFtr#108
Streptococcaceae (22/39)
DUF3013,rarA,6SRNA,tRNA-Lys
Streptococcus hyovaginalis
GCF 000420785.1, 0≙9701, -, NZ_ATVP01000002.1
1rNm
5rpLm
hp
DUF3013
GNAT
rarA
ssi
DUF771
hp
hp
hp
Streptococcaceae (22/39)
DUF3013,rarA,6SRNA,tRNA-Lys
Streptococcus ictaluri
GCF 000188015.2, 0≙106084, -, NZ_AEUX02000001.1
DUF3013
NINE
ascl
t1ga
rarA
ssi#122
tra#302
ssi
DUF771
IS30
hp
hp
Streptococcaceae (22/39)
DUF3013,rarA,6SRNA,tRNA-Lys
Streptococcus macacae
GCF 000187995.2, 0≙1482710, +, NZ_AEUW02000001.1
5rpLm
_LysR
_DUF486
DUF3013
rarA
trre
mepr
Streptococcaceae (22/39)
DUF3013,rarA,6SRNA,tRNA-Lys
Streptococcus minor
GCF 000377005.1, 0≙22807, -, NZ_AQYB01000027.1
1rNm
5rpLm
Vfp
DUF3013
rarA
artrap
GNAT
gfo
Streptococcaceae (22/39)
DUF3013,rarA,6SRNA,tRNA-Lys
Streptococcus mutans
GCF 001558215.1, 0≙1710864, -, NZ_CP013237.1
1rNm
5rpLm
hp
DUF3013
GNAT
rarA
ATPase
_MerR
_phosph
Streptococcaceae (22/39)
DUF3013,rarA,6SRNA,tRNA-Lys
Streptococcus orisratti
GCF 000380105.1, 0≙49178, +, NZ_KB904537.1
hp
Rbp
DUF3013
GNAT
rarA
lare
gamu
Streptococcaceae (22/39)
DUF3013,rarA,6SRNA,tRNA-Lys
Streptococcus ovis
GCF 000380125.1, 0≙207819, +, NZ_KB904563.1
_Llo
DUF3013
rarA
Ugm
artrap
GNAT
gfo
Streptococcaceae (22/39)
DUF3013,rarA,6SRNA,tRNA-Lys
Streptococcus pantholopis
GCF 001642085.1, 0≙1989794, +, NZ_CP014699.1
1rNm
5rpLm
DUF3013
rarA
_ISL3
hp
_collag
Streptococcaceae (22/39)
DUF3013,rarA,6SRNA,tRNA-Lys
Streptococcus parasanguinis
GCF 000164675.2, 0≙1812485, -, NC_015678.1
1rNm
5rpLm
Nac#143
MutT
DUF3013
rarA
hp
AtAbp
hp
_GHKL
_DNA
Streptococcaceae (22/39)
DUF3013,rarA,6SRNA,tRNA-Lys
Streptococcus parauberis
GCF 002900385.1, 0≙496122, -, NZ_CP025420.1
DUF3013
site
csp
GNAT
rarA
_citrat
_NAD
Streptococcaceae (22/39)
DUF3013,rarA,6SRNA,tRNA-Lys
Streptococcus pluranimalium
GCF 002953735.1, 0≙1953617, +, NZ_CP025536.1
5rpLm
_GNAT
MutT
DUF3013
GNAT
rarA
ssi
DUF771
_replic
_cell
_RNA
Streptococcaceae (22/39)
DUF3013,rarA,6SRNA,tRNA-Lys
Streptococcus suis
GCF 000168355.3, 0≙2096307, +, NZ_CP002007.2
1rNm
5rpLm
Rbp
DUF3013
rarA
artrap
hp
GNAT
_metall
hp#121
Streptococcaceae (22/39)
DUF3013,rarA,6SRNA,tRNA-Lys
Streptococcus suis
GCF 002803825.1, 0≙2428103, +, NZ_CP024974.1
5rpLm
Rbp
DUF3013
nucleo
rarA
artrap
GNAT
hp
hp#121
artr
hp
-5000
-2500
0
2500

abbreviation	full description
1dDx5pr	1-deoxy-D-xylulose-5-phosphate reductoisomerase.
1rNm	16S rRNA (uracil(1498)-N(3))-methyltransferase.
_2t2dNa	2,3,4,5-tetrahydropyridine-2,6-dicarboxylate N-acetyltransferase.
_50S	50S ribosomal protein L28.
5fcl	5-formyltetrahydrofolate cyclo-ligase.
5rpLm	50S ribosomal protein L11 methyltransferase.
AdmFfp	ATP-dependent metallopeptidase FtsH/Yme1/Tma family protein.
_alpha	alpha/beta hydrolase.
_aminog	aminoglycoside phosphotransferase.
_AraC	AraC family transcriptional regulator.
ArsR#63	ArsR family transcriptional regulator (orthology group #63).
_ArsR	ArsR family transcriptional regulator.
artr	anaerobic ribonucleoside-triphosphate reductase.
artrap	anaerobic ribonucleoside-triphosphate reductase activating protein.
ascI	aminodeoxychorismate synthase, component I.
AtAbp#303	ABC transporter ATP-binding protein (orthology group #303).
AtAbp	ABC transporter ATP-binding protein.
ATPase	heavy metal translocating P-type ATPase.
_ATP	ATP-binding protein.
_ATP	ATP-grasp domain-containing protein.
_carbox	carboxymuconolactone decarboxylase family protein.
_cell	cell division protein FtsK.
_cell	cell surface protein.
_citrat	citrate:sodium symporter.
_collag	collagen-binding protein.
_Cro	Cro/CI family transcriptional regulator.
csp	cold-shock protein.
Dbrr	DNA-binding response regulator.
_deoxyn	deoxynucleoside kinase.
_DNA	DNA-binding response regulator.
_DUF121	DUF1211 domain-containing protein.
DUF1292	DUF1292 domain-containing protein.
_DUF130	DUF1304 domain-containing protein.
_DUF175	DUF1751 domain-containing protein.
DUF3013	DUF3013 domain-containing protein.
_DUF302	DUF3021 domain-containing protein.
DUF3173	DUF3173 domain-containing protein.
_DUF429	DUF4298 domain-containing protein.
DUF4357	DUF4357 domain-containing protein.
_DUF443	DUF4430 domain-containing protein.
_DUF486	DUF4865 domain-containing protein.
_DUF494	DUF4947 domain-containing protein.
_DUF59	DUF59 domain-containing protein.
_DUF771	DUF771 domain-containing protein.
DUF771	DUF771 domain-containing protein.
end	endonuclease.
_exonuc	exonuclease SbcC.
FlaR	DNA topology modulation protein FlaR.
g5k	glutamate 5-kinase.
g5sd	glutamate-5-semialdehyde dehydrogenase.
GalE	UDP-glucose 4-epimerase GalE.
gamu	galactose mutarotase.
gfo	gfo/Idh/MocA family oxidoreductase.
_GHKL	GHKL domain-containing protein.
GNAT	GNAT family acetyltransferase.
_GNAT	GNAT family N-acetyltransferase.
GNAT	GNAT family N-acetyltransferase.
_Hdcp	HD domain-containing protein.
_HIT	HIT family protein.
Hollida	Holliday junction resolvase RuvX.
hp	hypothetical protein.

hp#104	hypothetical protein (orthology group #104).
hp#110	hypothetical protein (orthology group #110).
hp#111	hypothetical protein (orthology group #111).
hp#115	hypothetical protein (orthology group #115).
hp#121	hypothetical protein (orthology group #121).
hp#125	hypothetical protein (orthology group #125).
hp#146	hypothetical protein (orthology group #146).
hp#151	hypothetical protein (orthology group #151).
hp#178	hypothetical protein (orthology group #178).
hp#56	hypothetical protein (orthology group #56).
hp#97	hypothetical protein (orthology group #97).
hthdcp	helix-turn-helix domain-containing protein.
hyph	hypoxanthine phosphoribosyltransferase.
Ift#297	IS3 family transposase (orthology group #297).
Ift	IS3 family transposase.
_integr	integrase.
IreB	IreB family regulatory phosphoprotein.
_IS30	IS30 family transposase.
IS30	IS30 family transposase.
_IS3	IS3 family transposase.
_IS3'	IS3 family transposase.
_IS5	IS5/IS1182 family transposase.
_ISL3	ISL3 family transposase.
lare	lactaldehyde reductase.
_Llo	L-lactate oxidase.
ltl	leucine–tRNA ligase.
_LysR	LysR family transcriptional regulator.
_mannit	mannitol-1-phosphate 5-dehydrogenase.
mepr	membrane protein.
MerR#182	MerR family transcriptional regulator (orthology group #182).
_MerR	MerR family DNA-binding transcriptional regulator.
MerR	MerR family transcriptional regulator.
_metall	metallophosphatase.
_MFS	MFS transporter.
MFtr#108	MFS transporter (orthology group #108).
MFtr#118	MFS transporter (orthology group #118).
_multid	multidrug ABC transporter permease.
MutT	DNA mismatch repair protein MutT.
Nac#120	N-acetyltransferase (orthology group #120).
Nac#143	N-acetyltransferase (orthology group #143).
_Nad	N-acetyldiaminopimelate deacetylase.
_NAD	NAD-dependent malic enzyme.
_Na	N-acetyltransferase.
_NINE	NINE protein.
_nucleo	nucleotide pyrophosphohydrolase.
phlt#152	putative holin-like toxin (orthology group #152).
phlt	putative holin-like toxin.
_phosph	phosphopantetheinyl transferase.
ptl	proline–tRNA ligase.
_PTS	PTS mannitol transporter subunit IIA.
_QVPTGV	QVPTGV class sortase B protein-sorting domain-containing protein.
Rbp	RNA-binding protein.
rarA	replication-associated recombination protein A.
_replic	replication-associated recombination protein A.

_replic	replication initiation protein.
_RNA	RNA-binding protein.
RseP	RIP metalloprotease RseP.
sap	surface-anchored protein.
_signal	signal peptidase I.
_site	site-specific integrase.
_SrtB	SrtB family sortase.
ssi#122	site-specific integrase (orthology group #122).
ssi	site-specific integrase.
_strept	streptomycin resistance protein.
_SulP	SulP family inorganic anion transporter.
t1ga	type 1 glutamine amidotransferase.
_TetR	TetR/AcrR family transcriptional regulator.
TIGR007	TIGR00730 family Rossman fold protein.
tra#302	transposase (orthology group #302).
_transc	transcriptional regulator.
_transc	transcription antiterminator.
_transp	transposase.
TrmB	tRNA (guanosine(46)-N7)-methyltransferase TrmB.
trre#124	transcriptional regulator (orthology group #124).
trre	transcriptional regulator.
_two	two-component sensor histidine kinase.
_UDP	UDP-glucose–hexose-1-phosphate uridylyltransferase.
Ugm	UDP-galactopyranose mutase.
Vfp	VOC family protein.
_XRE	XRE family transcriptional regulator.
YaaA	peroxide stress protein YaaA.
_Zdp	Zn-dependent protease.
_zinc	zinc transporter ZupT.

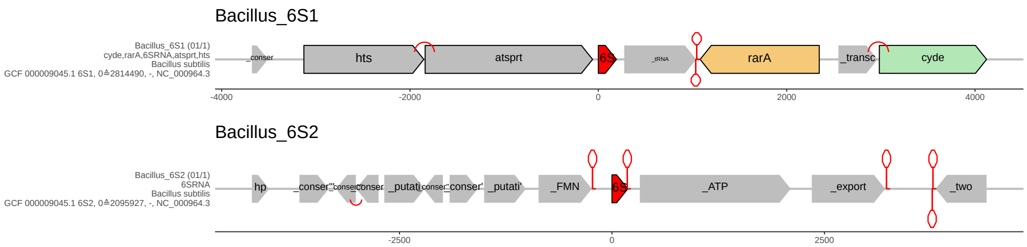

abbreviation	full description
atsprt	aspartyl-tRNA synthetase, promiscuous (also recognizes tRNAasn).
_conser	conserved protein of unknown function.
cyde	cysteine desulfurase.
hts	histidyl-tRNA synthetase.
rarA	replication-associated recombination protein A.
_transc	transcriptional regulator of cysteine biosynthesis.
_tRNA	tRNA threonylcarbamoyladenosine dehydratase (t(6)A37 dehydratase).
_ATP	ATP-dependent helicase.
_conser"	conserved hypothetical phage protein.
_conser	conserved protein of unknown function.
_conser'	conserved protein of unknown function.
_conser"'	conserved protein of unknown function (mother cell in sporulation).
_conser""	conserved protein of unknown function (sporulation-related).
_export	exported cell wall lytic enzyme.
_FMN	FMN-dependent NADH-azoreductase.
hp	conserved hypothetical protein.
_putati'	putative chaperone.
_putati	putative general stress protein.
_two	two-component response regulator [DesK].
