# Supplementary material for: Insights into 6S RNA in lactic acid bacteria (LAB)
Source: BMC Genom Data. 2021 Sep 3;22:29. doi: 10.1186/s12863-021-00983-2 (PMC8414754; doi:10.1186/s12863-021-00983-2)
Supplement: Supplementary file 5 — Additional file 5 6S RNA grouped consensus alignment (pdf). Folded consensus structure of the 6S RNA groups analogous to Fig. 3. [file 12863_2021_983_MOESM5_ESM.pdf]

Additional File 5 — 6S RNA grouped consensus alignment (pdf)

Folded consensus structure of the 6S RNA groups generated with `RNAclust` (the names next to the colored square boxes indicate the family). The consensus secondary structures were calculated with `RNAalifold` and visualized using `VARNA`. The name right above the structure indicates the `RNAclust` group name in line with Fig. 1. Colors indicate sequence conservation within the respective LAB family.

Aerococcaceae

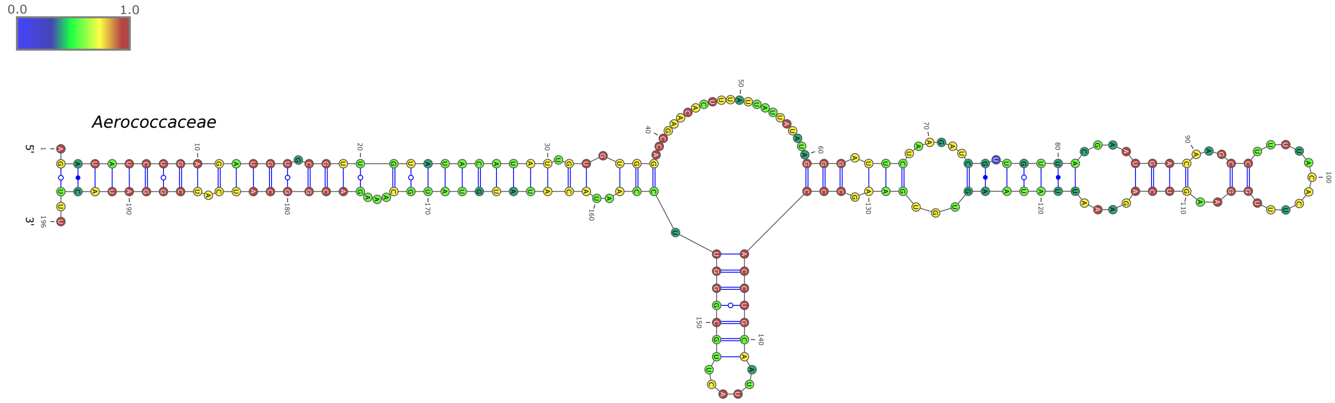

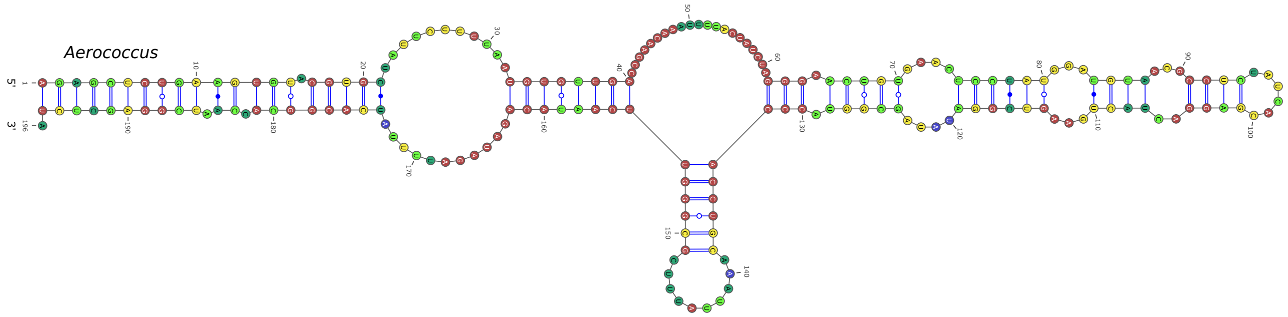

Carnobacteriaceae

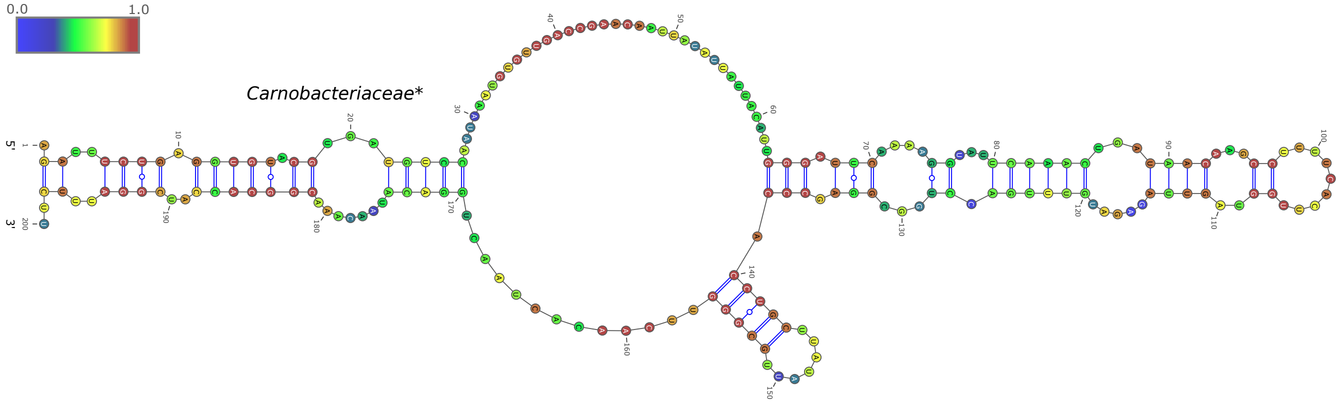

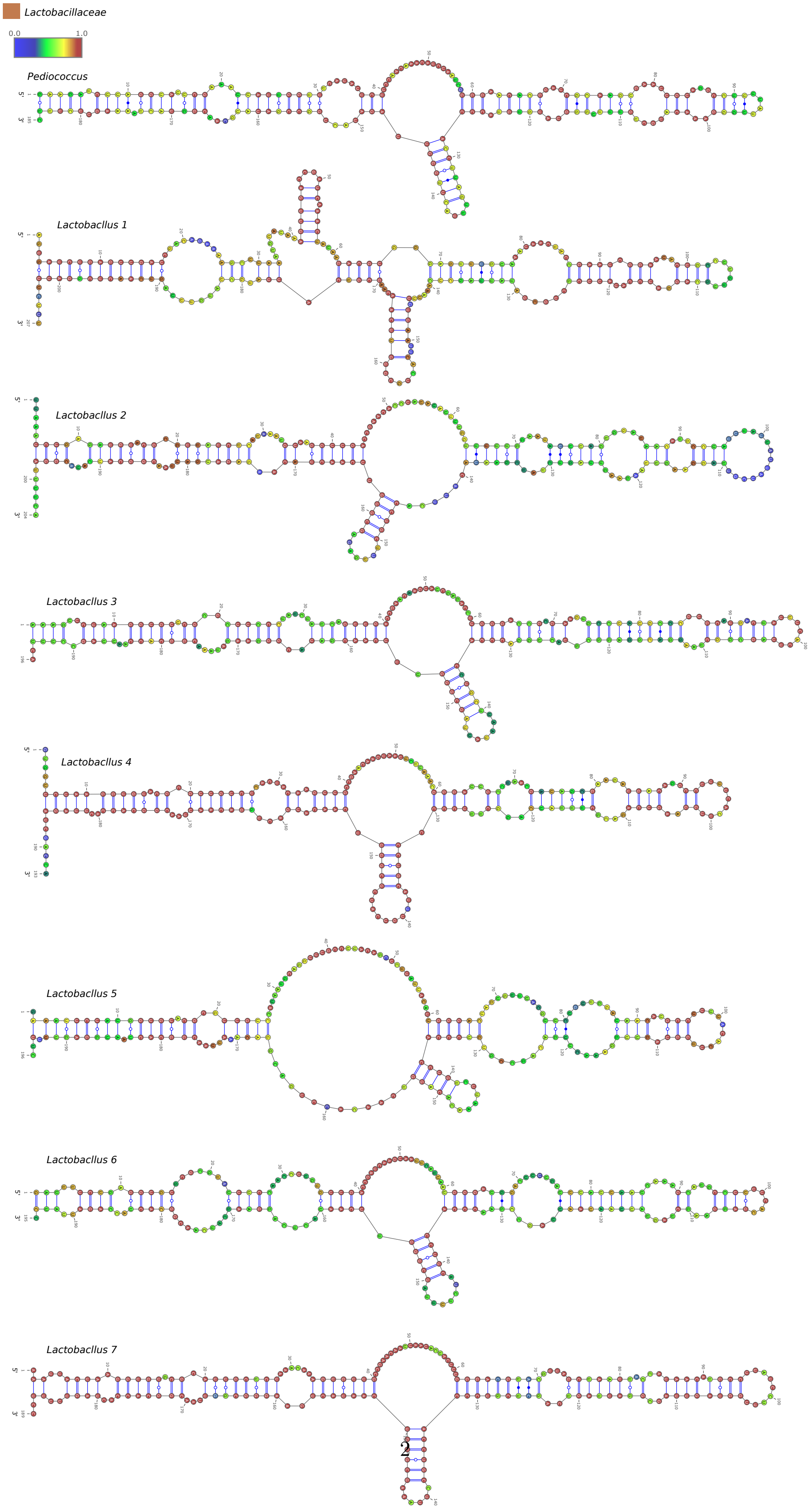
Lactobacillaceae
0.0
1.0
Pediococcus
Lactobacllus 1
Lactobacllus 2
Lactobacllus 3
Lactobacllus 4
Lactobacllus 5
Lactobacllus 6
Lactobacllus 7

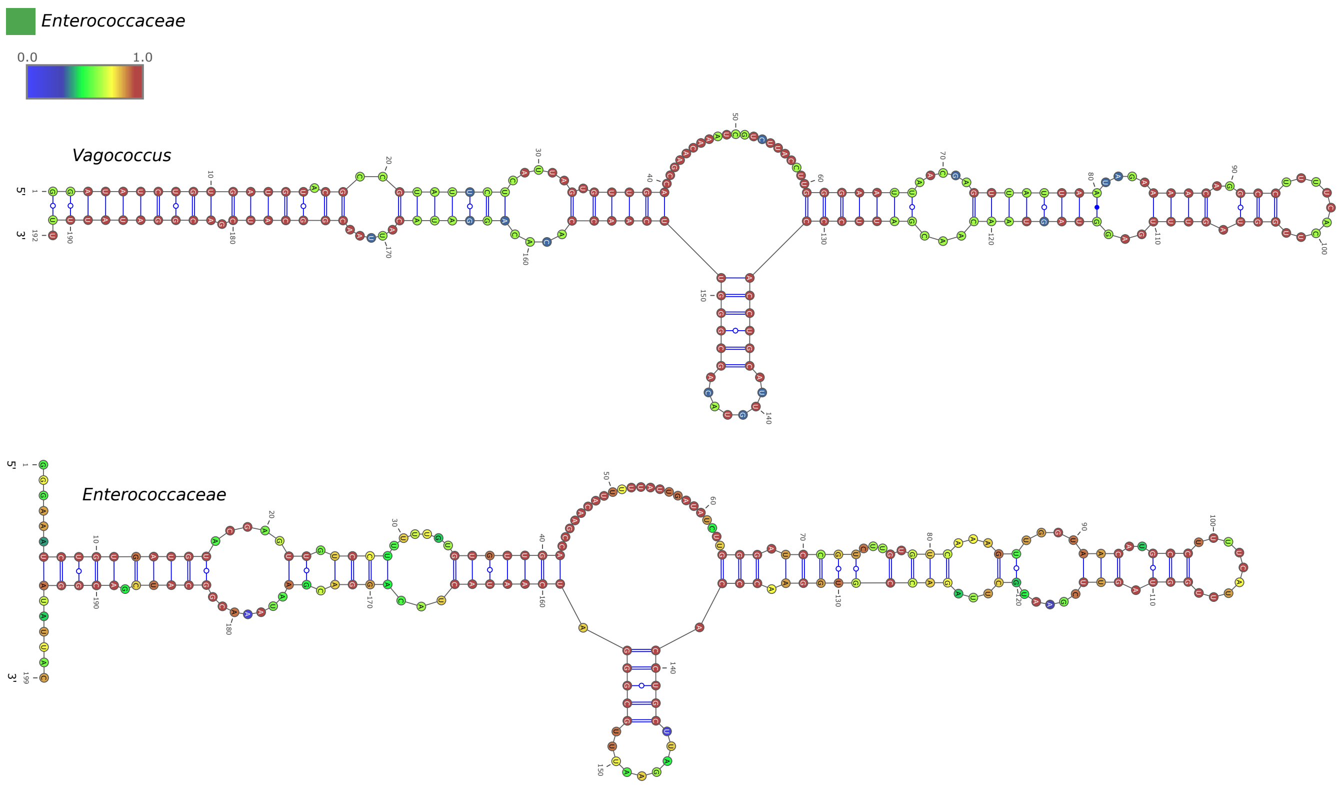
Enterococcaceae
0.0
1.0
Vagococcus
5'
3'
Enterococcaceae
5'
3'

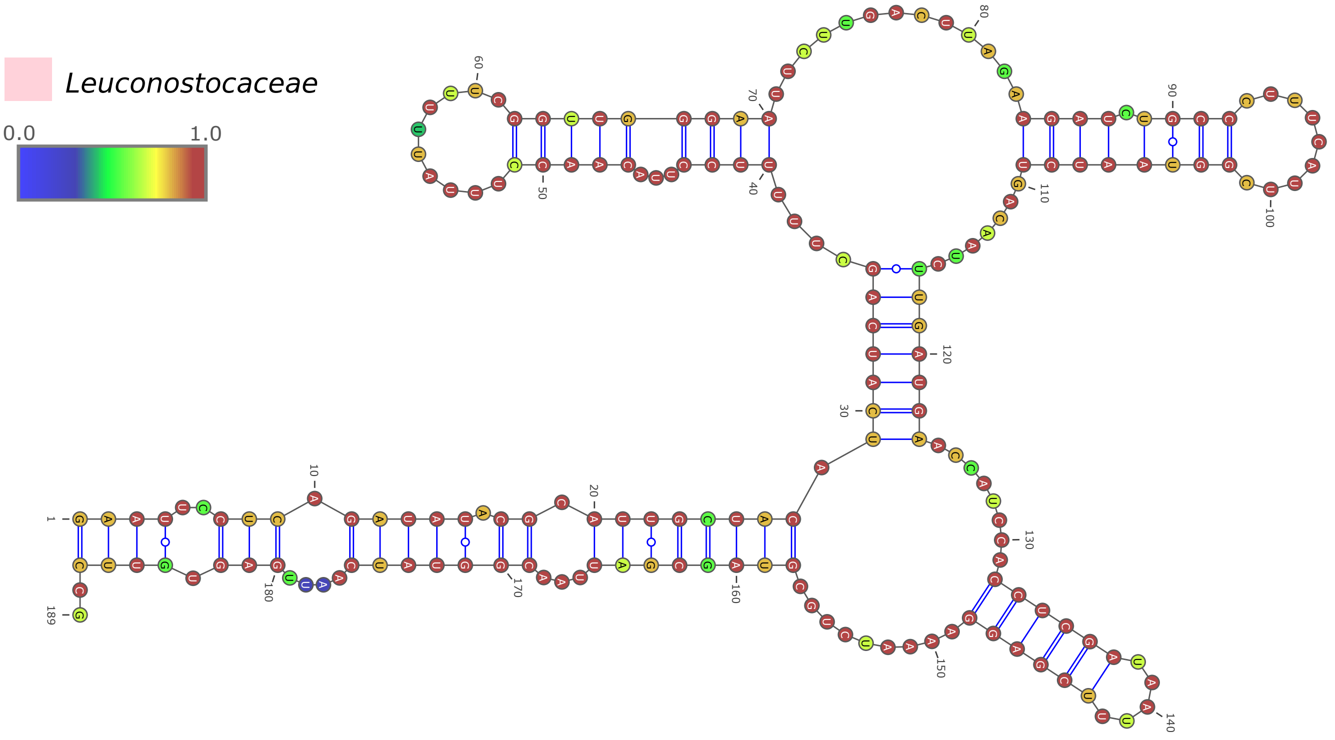
Leuconostocaceae
0.0
1.0

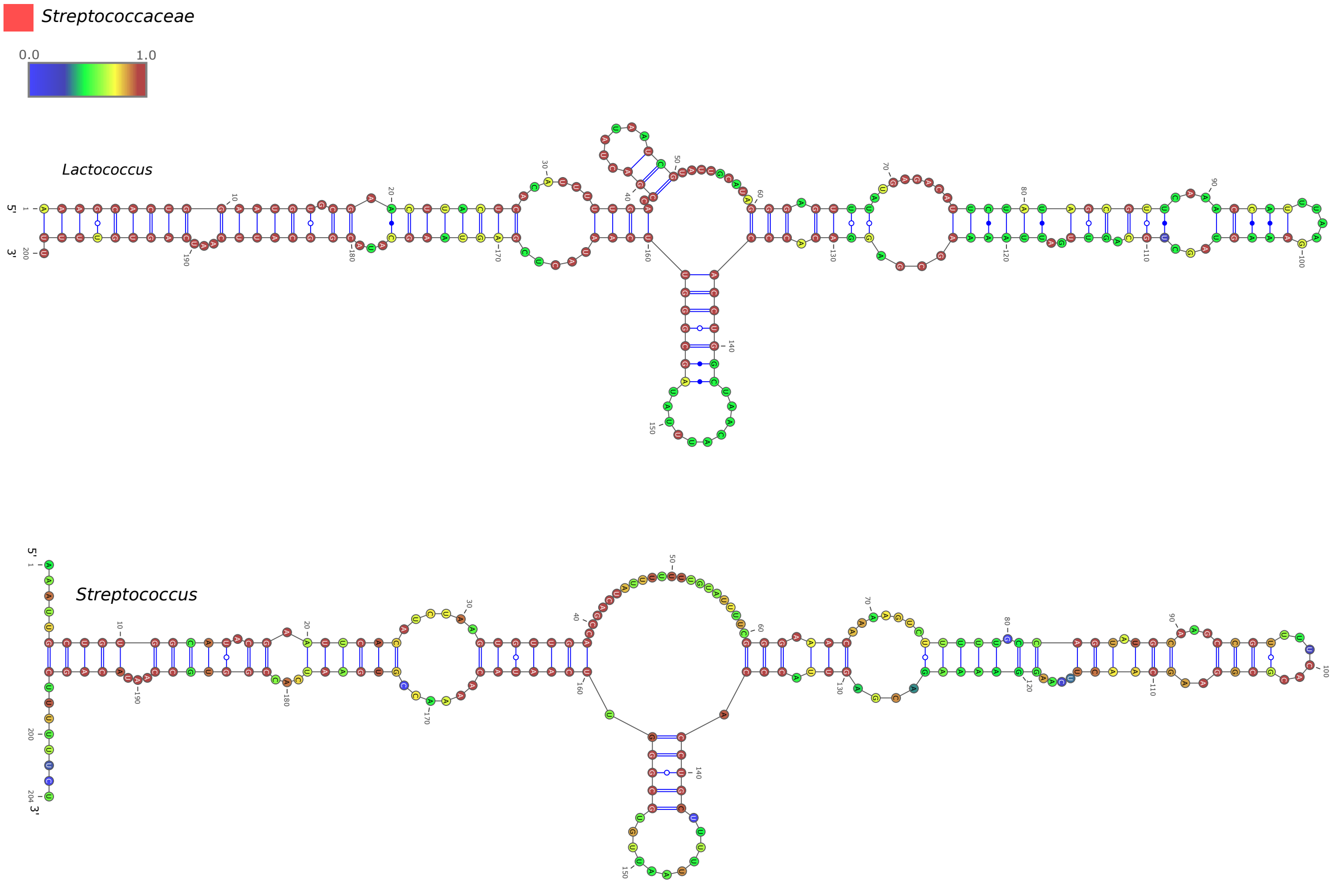
Streptococcaceae
0.0
1.0
Lactococcus
5'
3'
Streptococcus
5'
3'
