# Supplementary material for: Insights into 6S RNA in lactic acid bacteria (LAB)
Source: BMC Genom Data. 2021 Sep 3;22:29. doi: 10.1186/s12863-021-00983-2 (PMC8414754; doi:10.1186/s12863-021-00983-2)
Supplement: Supplementary file 6 — Additional file 6 RNA-Seq results (pdf). Visualization of RNA-Seq libraries mapped to the respective 6S RNA loci. [file 12863_2021_983_MOESM6_ESM.pdf]

# Additional File 6 — RNA-Seq data for 6S RNA

Publicly available LAB RNA-Seq data was fetched from NCBI and mapped to the 6S RNA locus identified in this study. Strain and target genome assembly are shown in the upper left. The upper right shows the respective chromosome-id and position. Bioproject and SRA-ID are indicated on the right. Bioprojects are denoted by different colors to ease reading. Library size (mapped reads only) is indicated in the bottom left. Mapping and positions are shown relative to the 6S RNA locus (framed by dashed lines). The putative pRNA-site is highlighted in red. Upper pannel shows 6S RNA reads (+), lower panel shows anti-sense reads (-). In the latter, read starts at potential pRNA sites are indicated by filled bars to ease the determination of the pRNA start positions.

In case of paired-end sequencing data, only the first strand library was mapped. Note that many libraries were not well described. Whenever issues were noticed, a note was added below each figure. E.g. “Looks like strand-independent sequencing” indicates that the number of reads on + and - strands are about equal indicating that they actually arose from the same strand. If no reads were found on the + strand, reads were “reverse complemented”.

# Enterococcaceae

## *Enterococcus faecalis ATCC 29212 (4)*

*Enterococcus faecalis (GCF_000742975.1)* NZ_CP008816.1: 956,503 .. 956,796

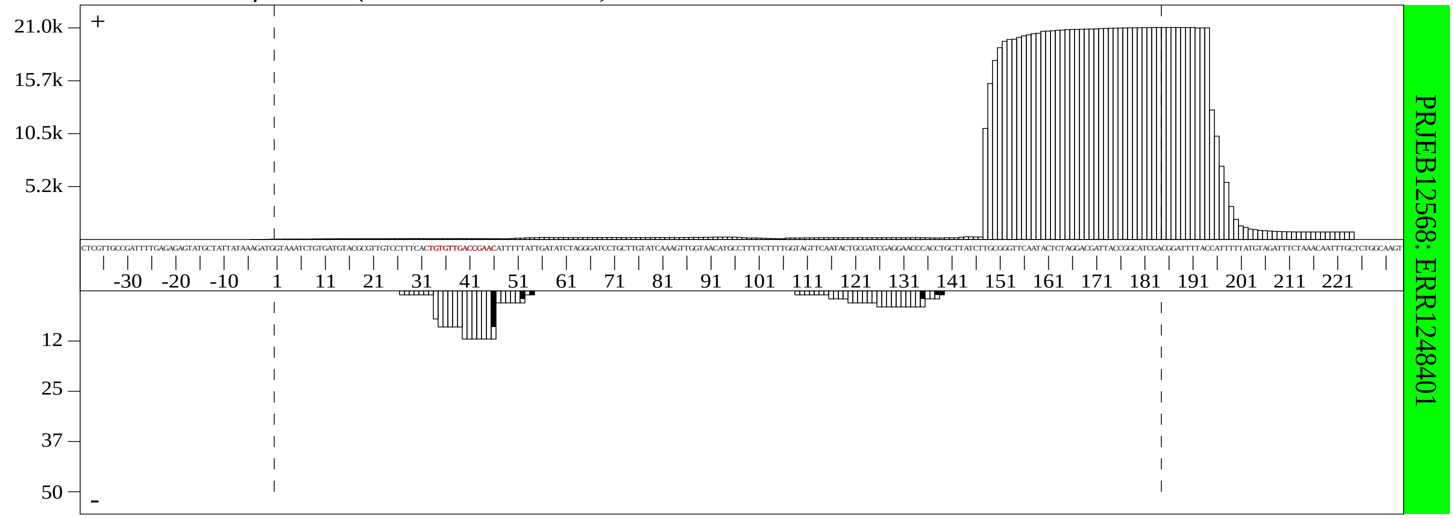


Lib size: 1,796k proposed pRNA: GUUCGGUCAA ...

Note: All reads reverse complemented

*Enterococcus faecalis (GCF_000742975.1)* NZ_CP008816.1: 956,503 .. 956,796

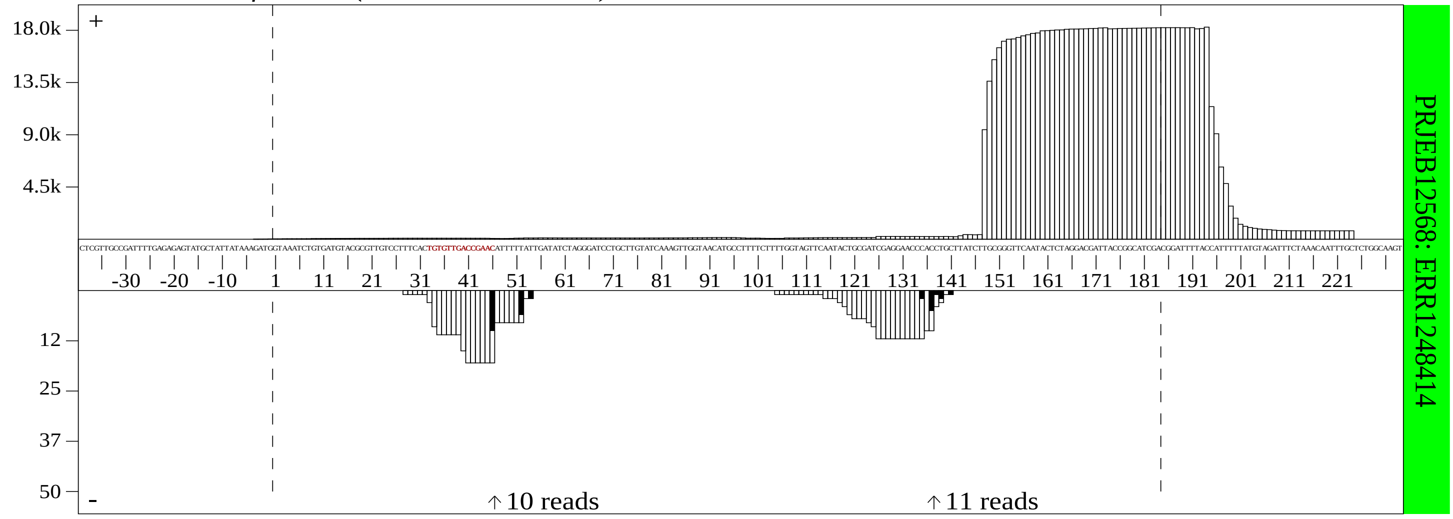


Lib size: 1,834k proposed pRNA: GUUCGGUCAA ...

Note: All reads reverse complemented Found pRNA: GUUCGGUCAA ...

*Enterococcus faecalis (GCF_000742975.1)* NZ_CP008816.1: 956,503 .. 956,796

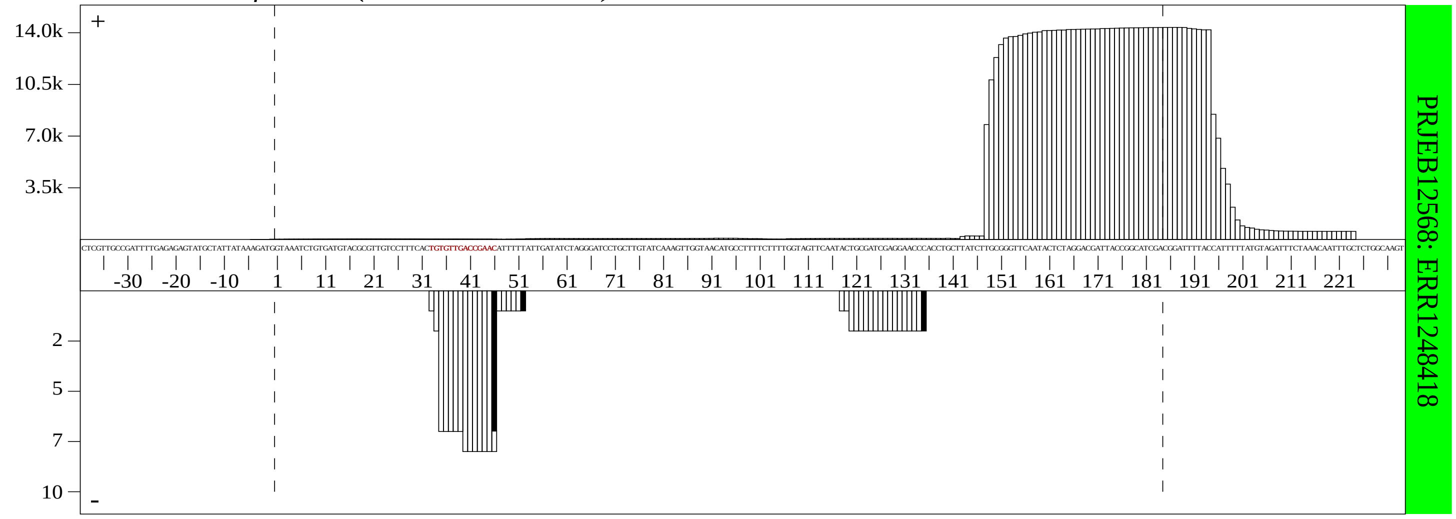


Lib size: 1,318k proposed pRNA: GUUCGGUCAA ...

Note: All reads reverse complemented

# Enterococcaceae

## *Enterococcus faecalis ATCC 29212 (4)*

*Enterococcus faecalis (GCF_000742975.1)* NZ_CP008816.1: 956,503 .. 956,796

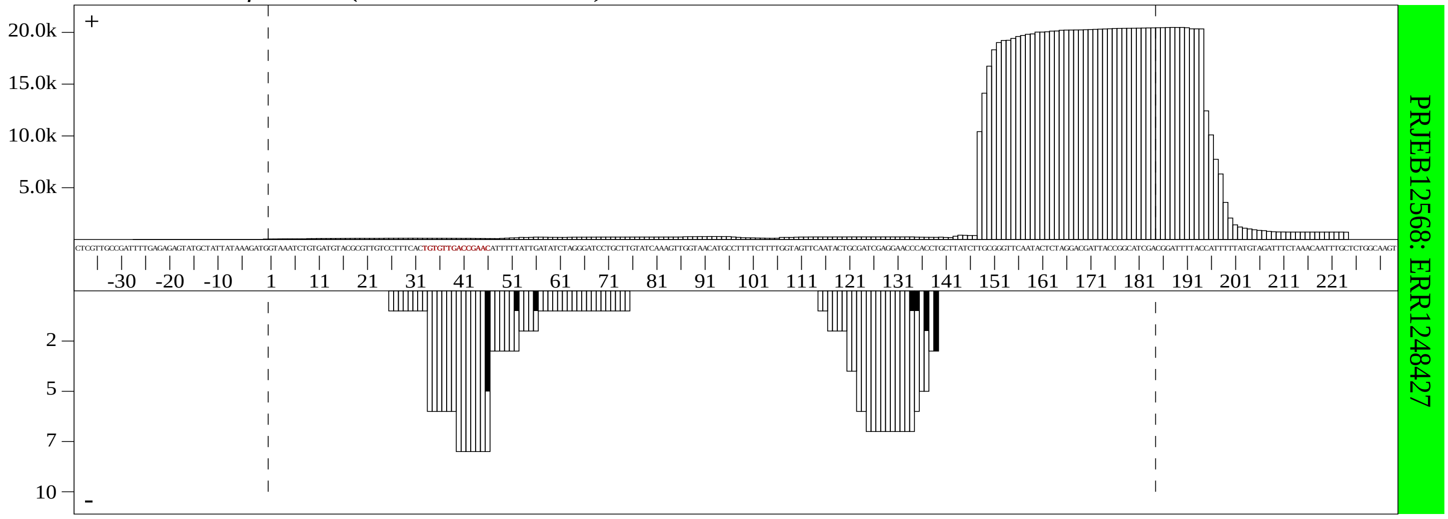


Lib size: 1,418k proposed pRNA: GUUCGGUCAA ...

Note: All reads reverse complemented

# Enterococcaceae

## *Enterococcus faecalis (13)*

*Enterococcus faecalis (GCF_001598635.1)* NZ_CP014949.1: 854,768 .. 855,061

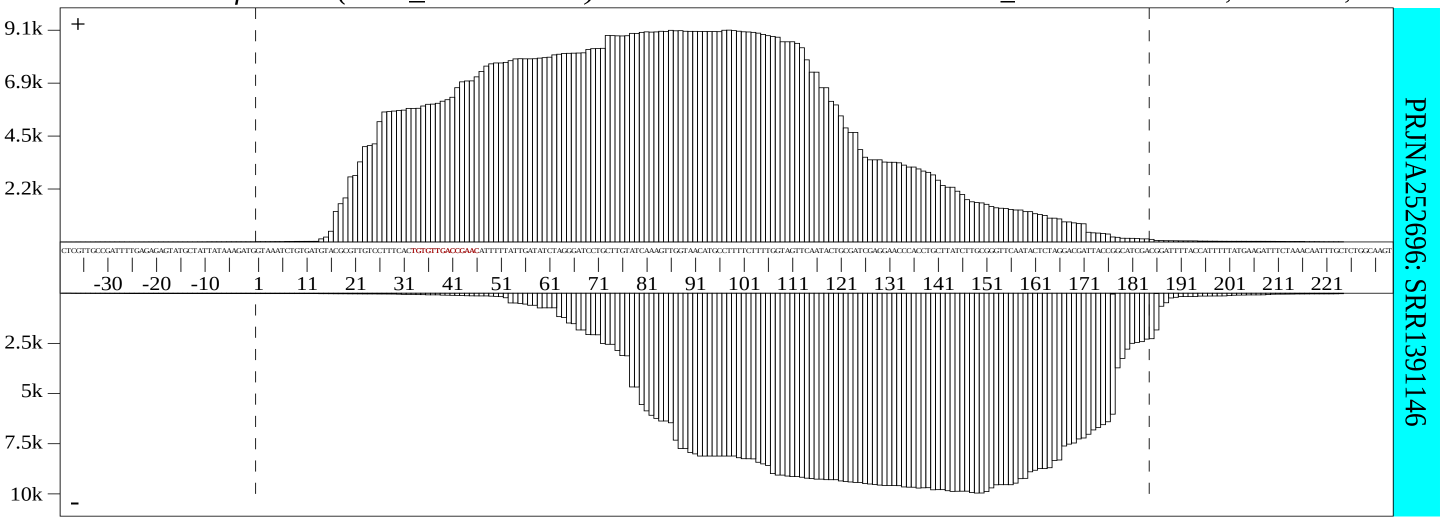


Lib size: 10,358k proposed pRNA: GUUCGGUCAA ...

Note: Looks like strand-independed sequencing

*Enterococcus faecalis (GCF_001598635.1)* NZ_CP014949.1: 854,768 .. 855,061

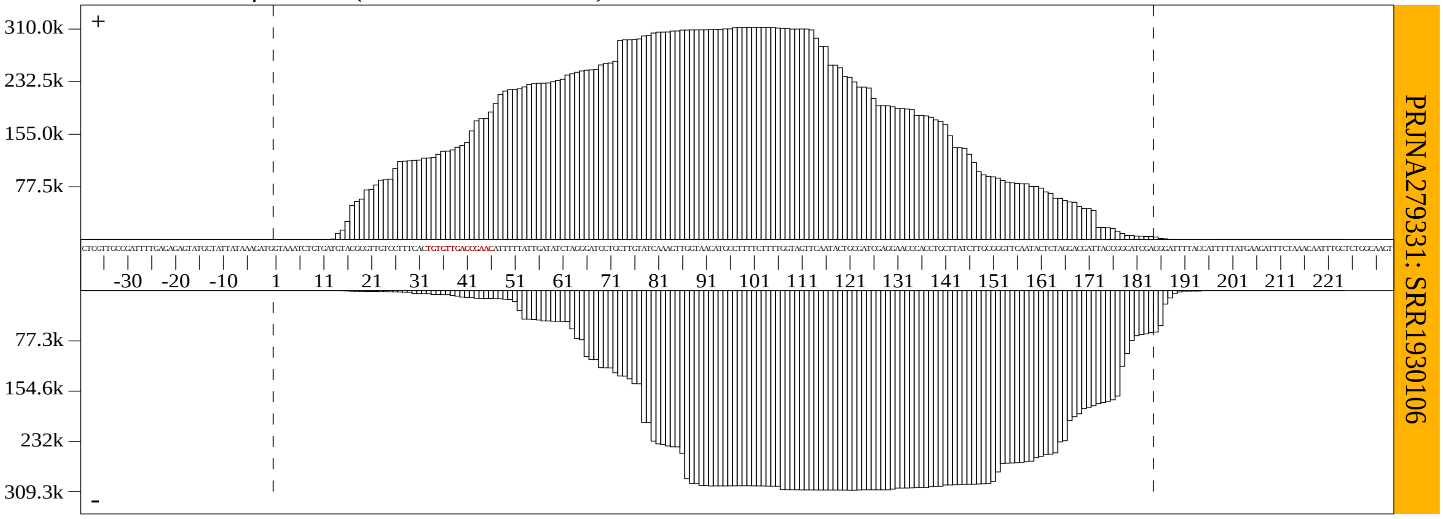


Lib size: 7,286k proposed pRNA: GUUCGGUCAA ...

Note: Looks like strand-independed sequencing

*Enterococcus faecalis (GCF_001598635.1)* NZ_CP014949.1: 854,768 .. 855,061

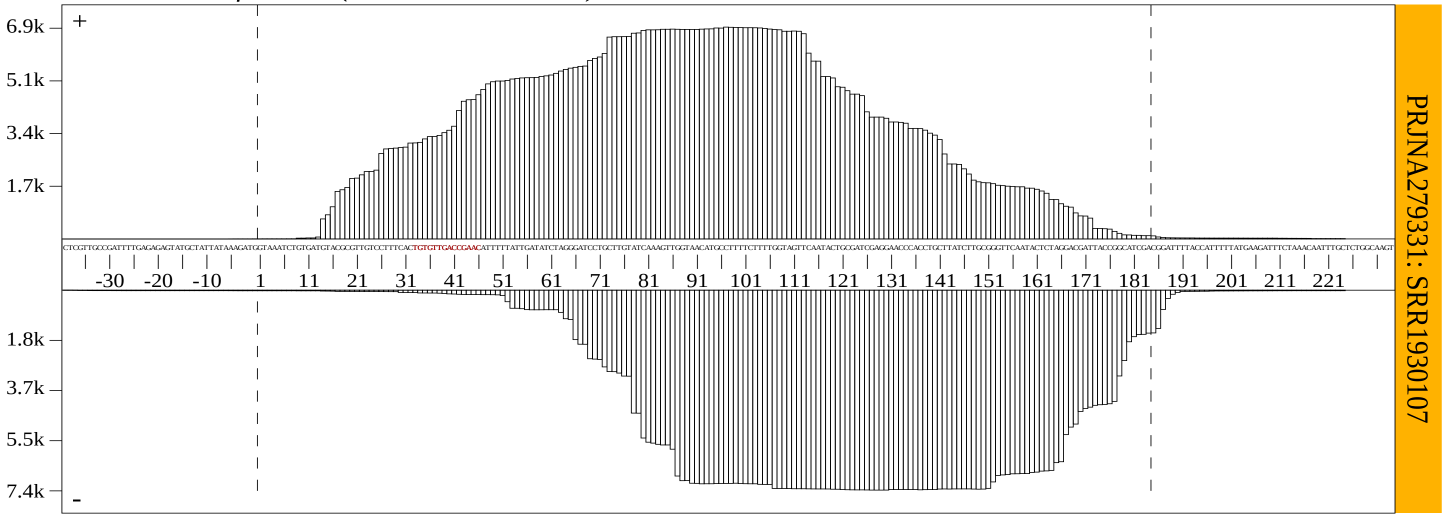


Lib size: 9,377k proposed pRNA: GUUCGGUCAA ...

Note: Looks like strand-independed sequencing

# Enterococcaceae

## *Enterococcus faecalis (13)*

*Enterococcus faecalis (GCF_001598635.1)* NZ_CP014949.1: 854,768 .. 855,061

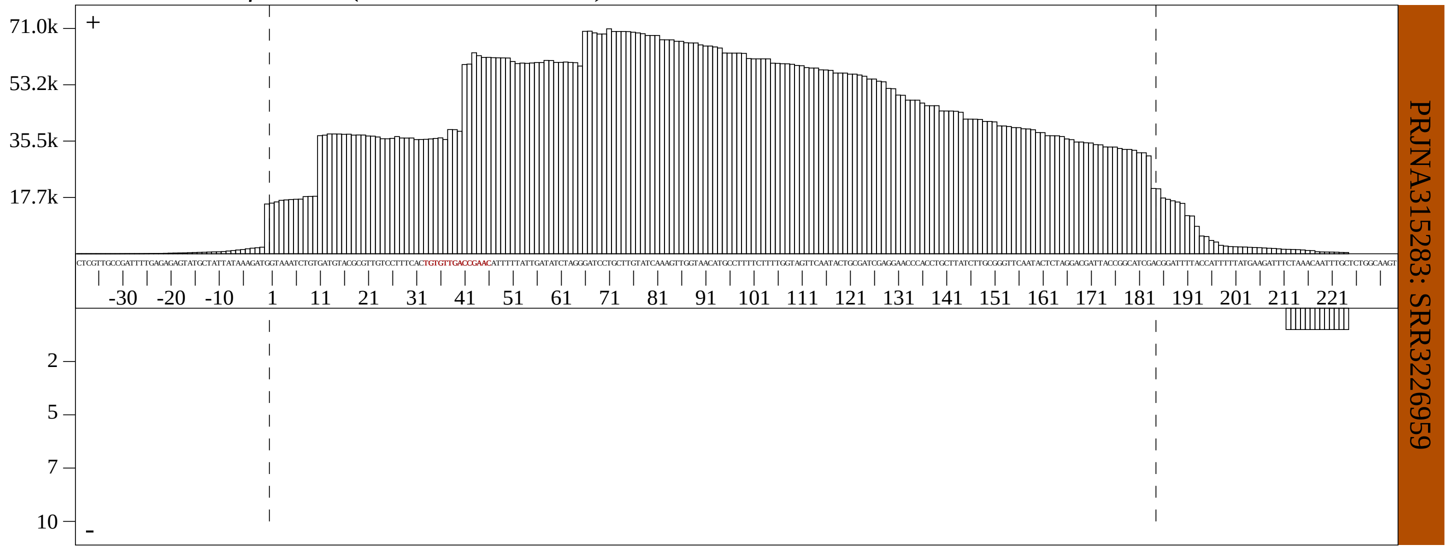


Lib size: 4,771k proposed pRNA: GUUCGGUCAA ...

*Enterococcus faecalis (GCF_001598635.1)* NZ_CP014949.1: 854,768 .. 855,061

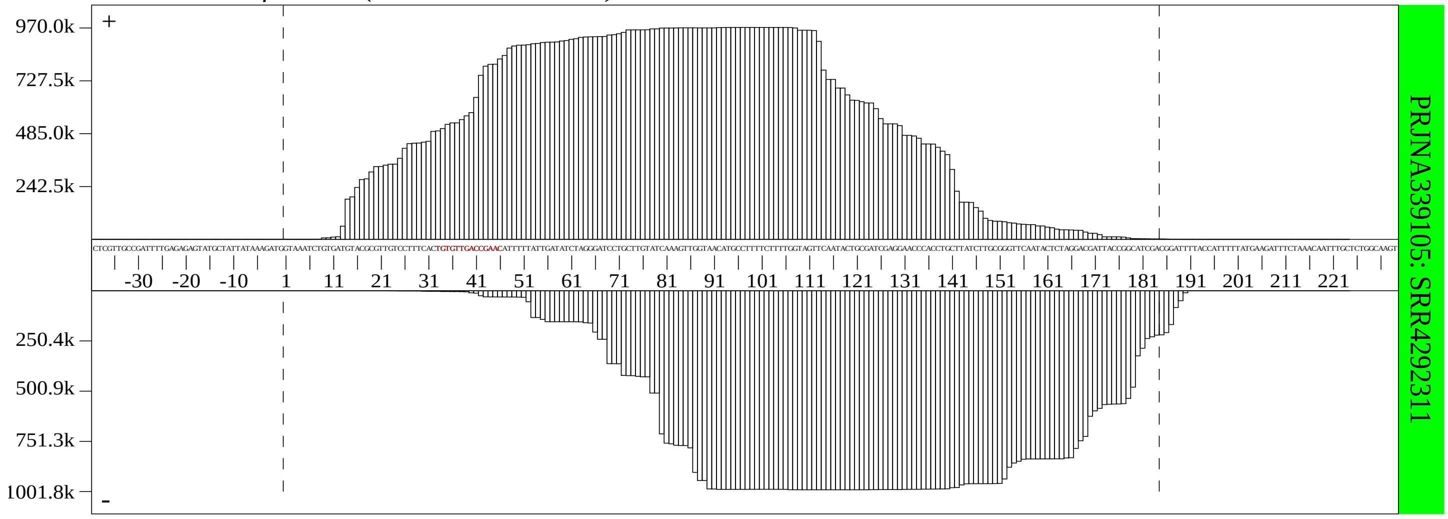


Lib size: 13,190k proposed pRNA: GUUCGGUCAA ...

Note: Looks like strand-independed sequencing

# Enterococcaceae

## *Enterococcus faecium Aus0004 (6)*

*Enterococcus faecium (GCF_000250945.1)* NC_017022.1: 2,529,576 .. 2,529,871

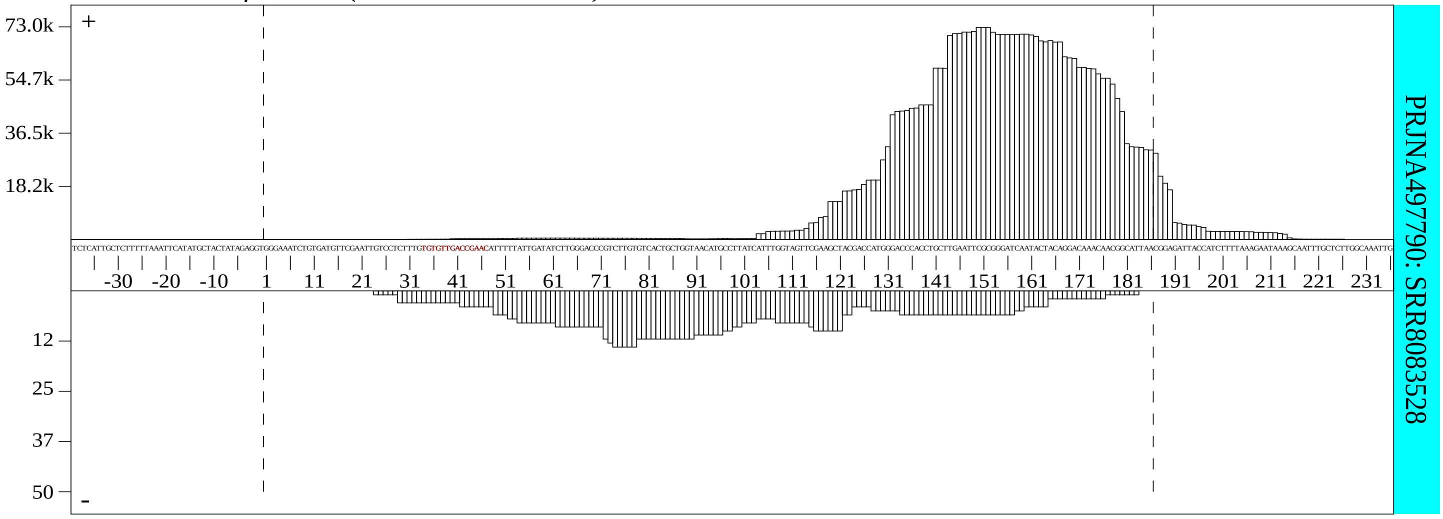


Lib size: 10,501k

proposed pRNA: GUUCGGUCAA ...

Note: All reads reverse complemented

*Enterococcus faecium (GCF_001298485.1)* NZ_CP012522.1: 1,158,967 .. 1,159,262

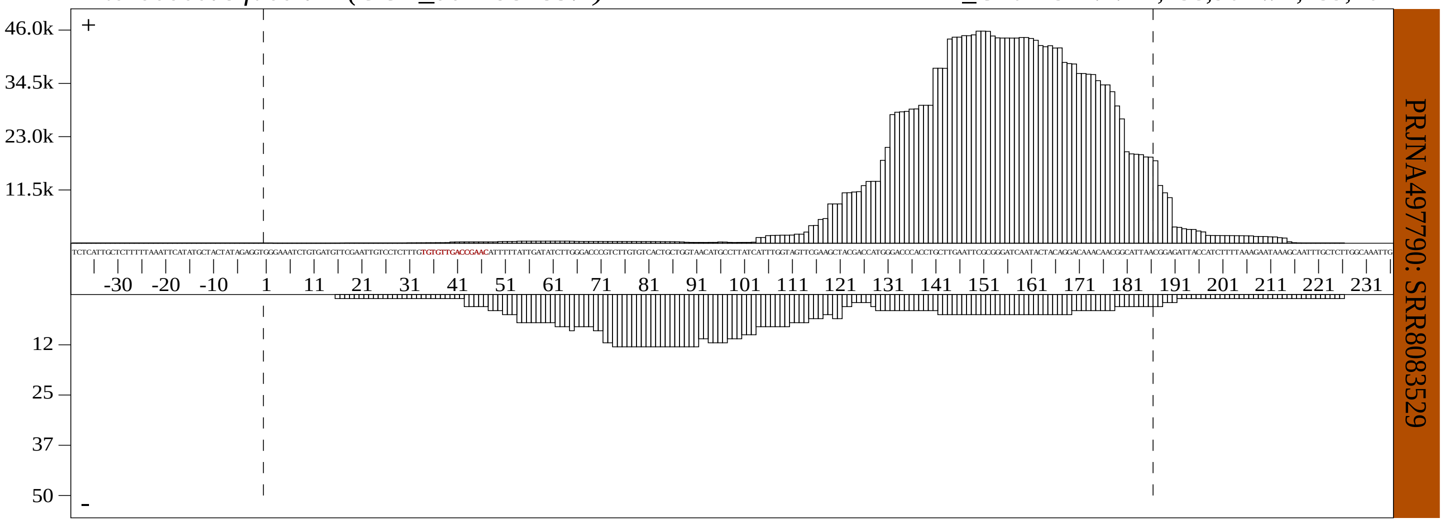


Lib size: 10,939k

proposed pRNA: GUUCGGUCAA ...

Note: All reads reverse complemented

*Enterococcus faecium (GCF_000250945.1)* NC_017022.1: 2,529,576 .. 2,529,871

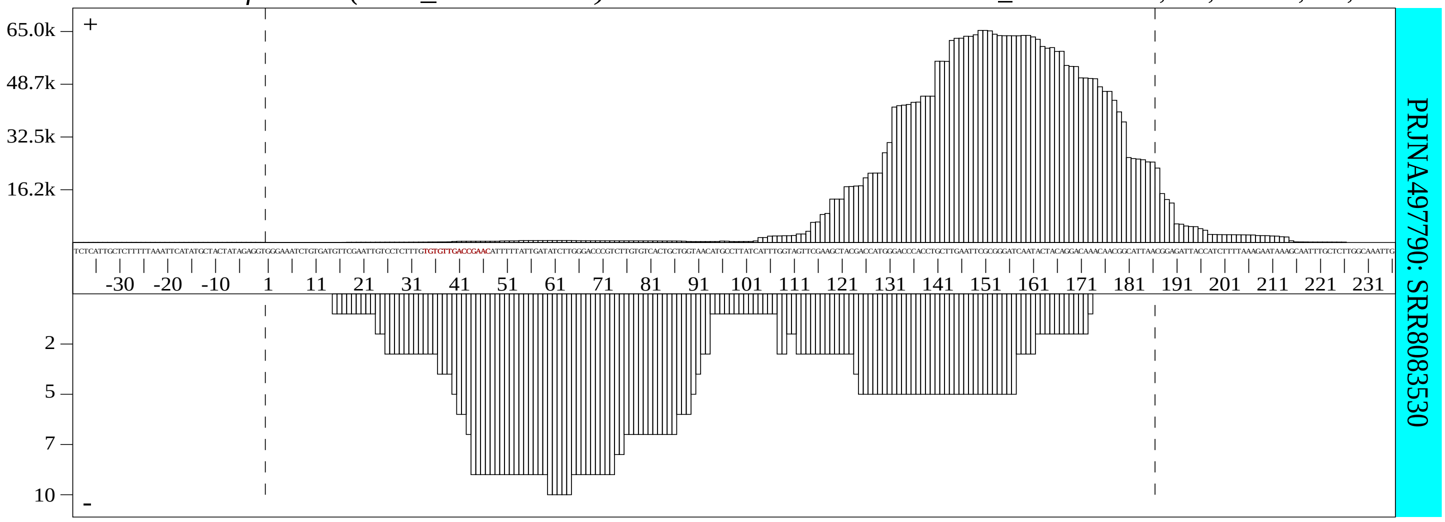


Lib size: 11,996k

proposed pRNA: GUUCGGUCAA ...

Note: All reads reverse complemented

# Enterococcaceae

## *Enterococcus faecium Aus0004 (6)*

*Enterococcus faecium (GCF_000250945.1)* NC_017022.1: 2,529,576 .. 2,529,871

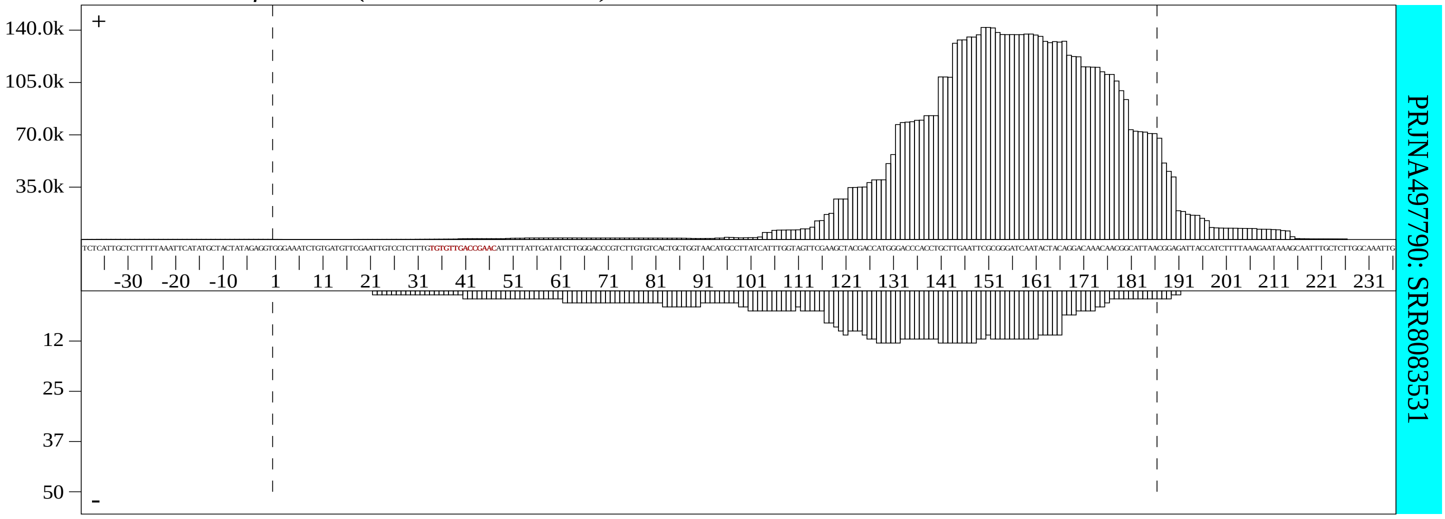


Lib size: 9,194k proposed pRNA: GUUCGGUCAA ...

Note: All reads reverse complemented

*Enterococcus faecium (GCF_000250945.1)* NC_017022.1: 2,529,576 .. 2,529,871

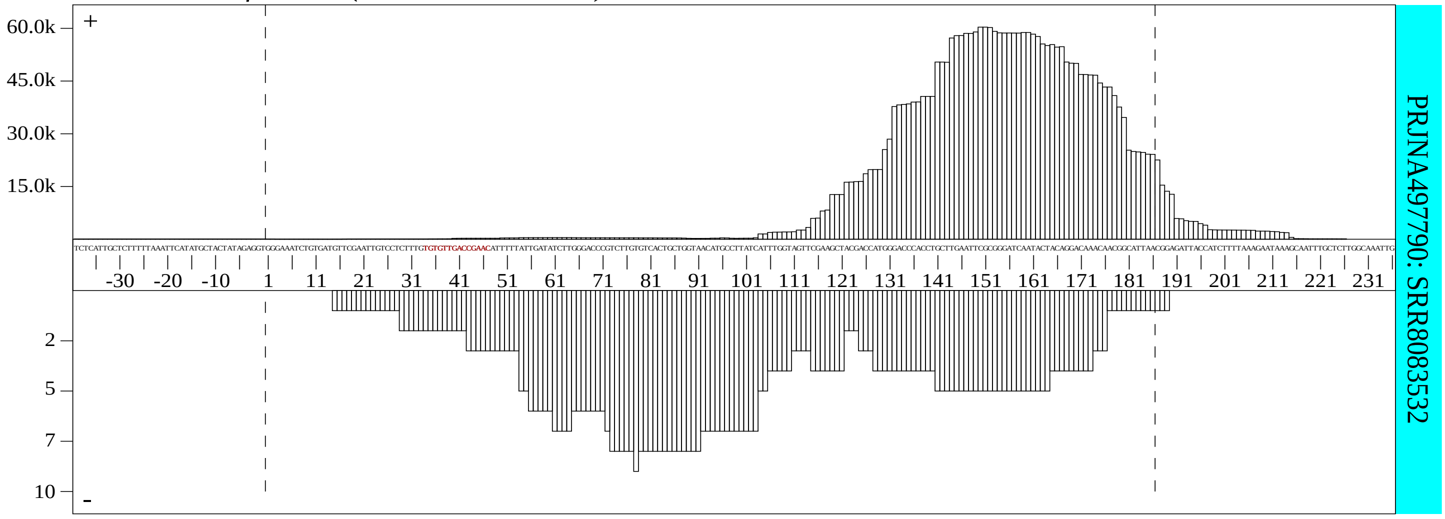


Lib size: 11,517k proposed pRNA: GUUCGGUCAA ...

Note: All reads reverse complemented

*Enterococcus faecium (GCF_000250945.1)* NC_017022.1: 2,529,576 .. 2,529,871

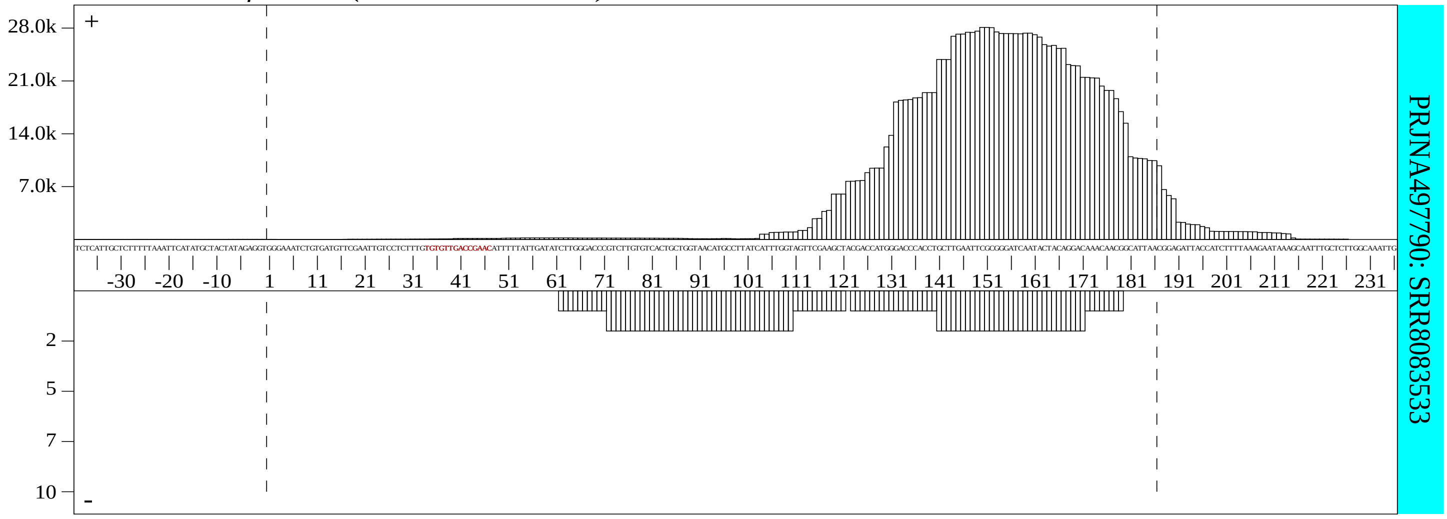


Lib size: 11,313k proposed pRNA: GUUCGGUCAA ...

Note: All reads reverse complemented

# Enterococcaceae

## *Enterococcus faecium (7)*

*Enterococcus faecium (GCF_001298485.1)* NZ_CP012522.1: 1,158,967 .. 1,159,262

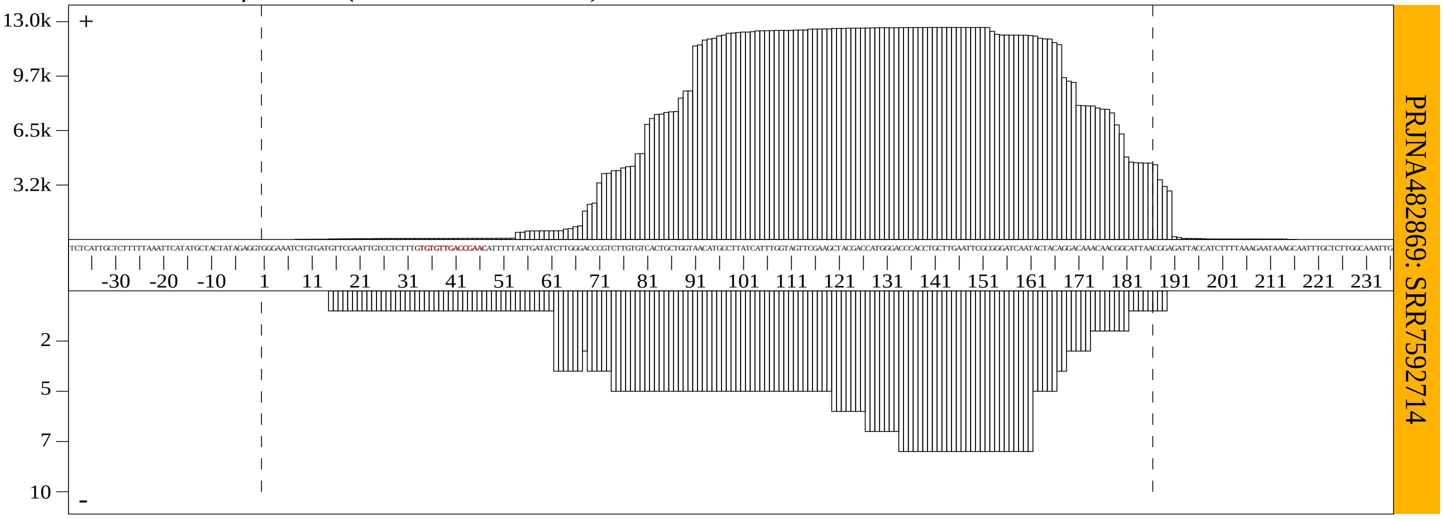


Lib size: 30,513k proposed pRNA: GUUCGGUCAA ...

Note: All reads reverse complemented

*Enterococcus faecium (GCF_001298485.1)* NZ_CP012522.1: 1,158,967 .. 1,159,262

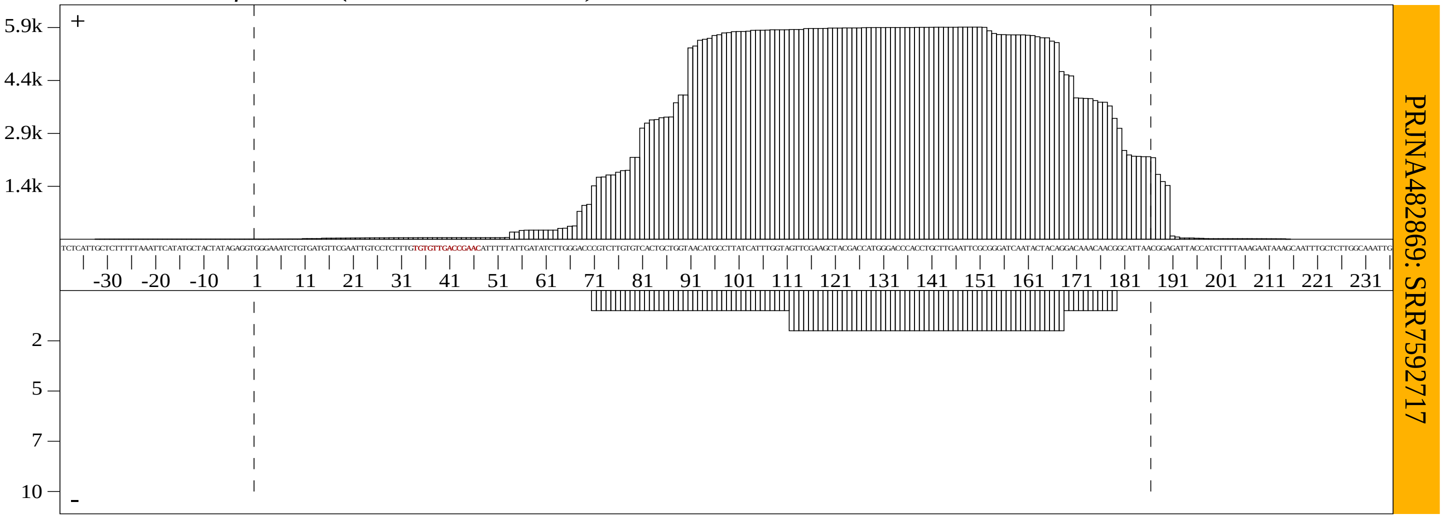


Lib size: 17,955k proposed pRNA: GUUCGGUCAA ...

Note: All reads reverse complemented

*Enterococcus faecium (GCF_001298485.1)* NZ_CP012522.1: 1,158,967 .. 1,159,262

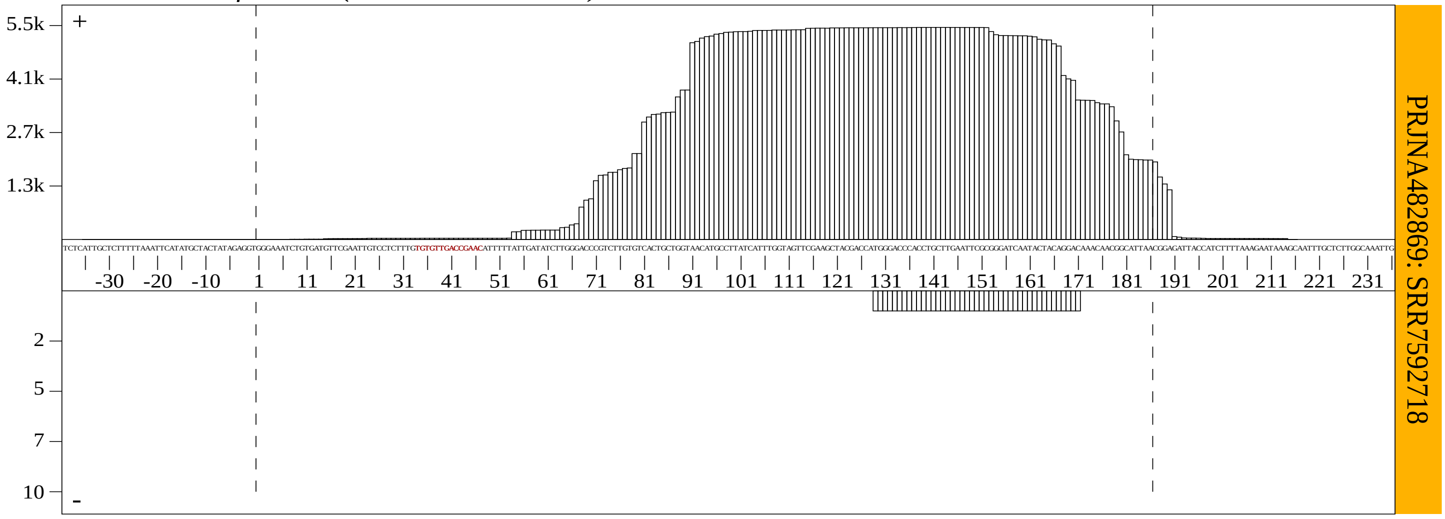


Lib size: 17,050k proposed pRNA: GUUCGGUCAA ...

Note: All reads reverse complemented

# Enterococcaceae

## *Enterococcus faecium (7)*

*Enterococcus faecium (GCF_001298485.1)* NZ_CP012522.1: 1,158,967 .. 1,159,262

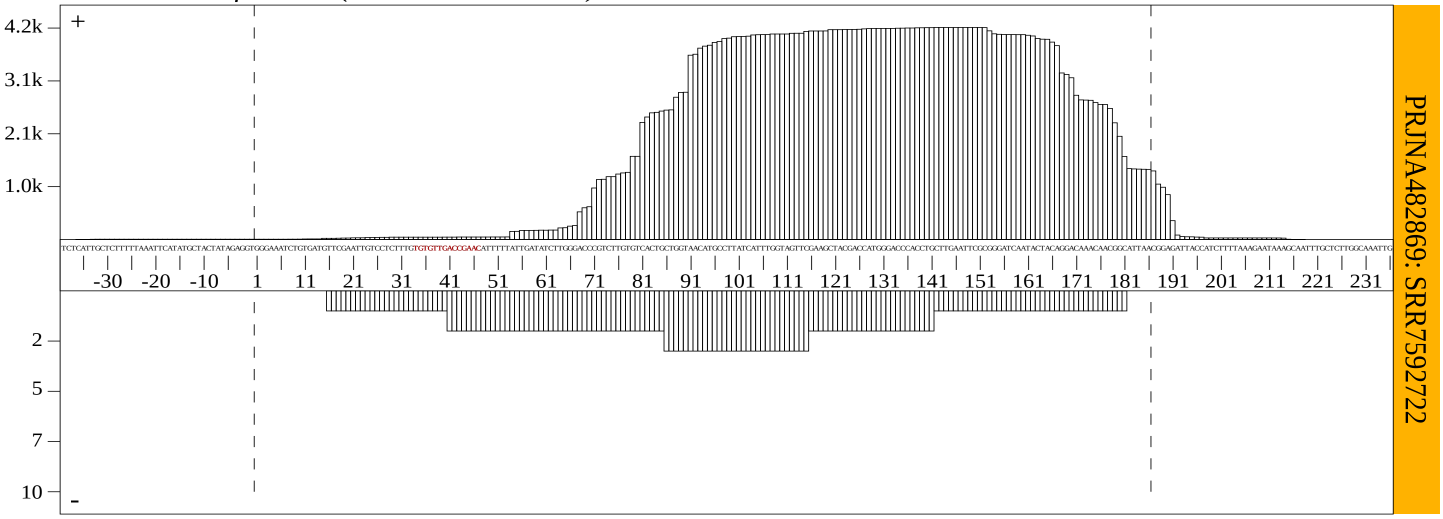


Lib size: 19,093k proposed pRNA: GUUCGGUCAA ...

Note: All reads reverse complemented

*Enterococcus faecium (GCF_001298485.1)* NZ_CP012522.1: 1,158,967 .. 1,159,262

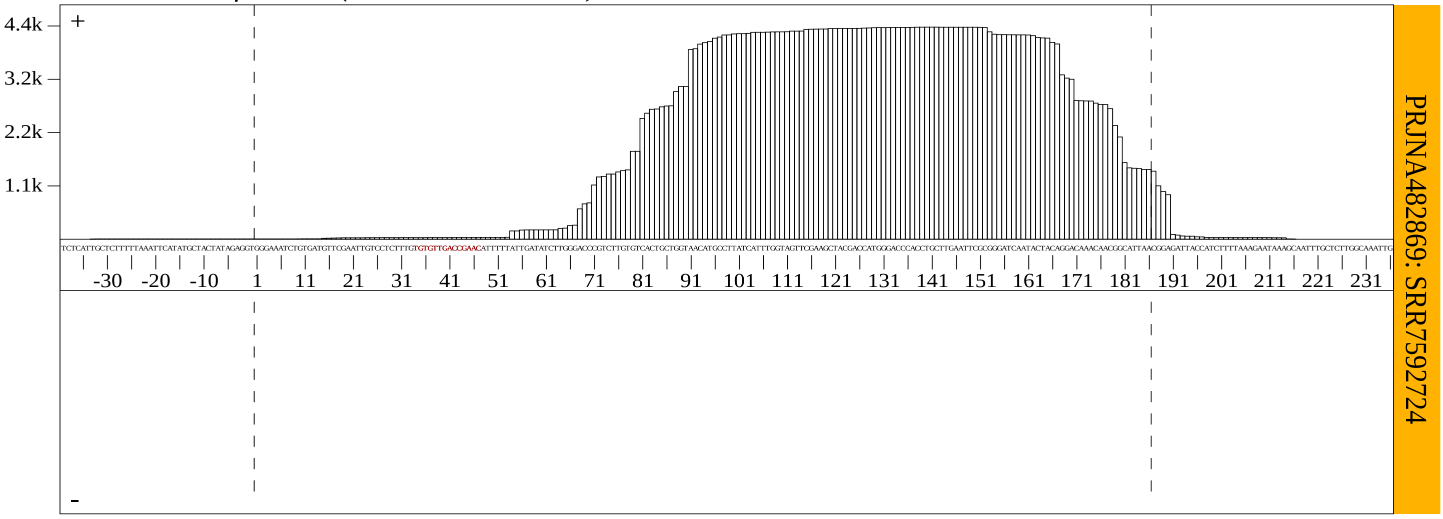


Lib size: 17,067k proposed pRNA: GUUCGGUCAA ...

Note: All reads reverse complemented No reads on - strand

*Enterococcus faecium (GCF_001298485.1)* NZ_CP012522.1: 1,158,967 .. 1,159,262

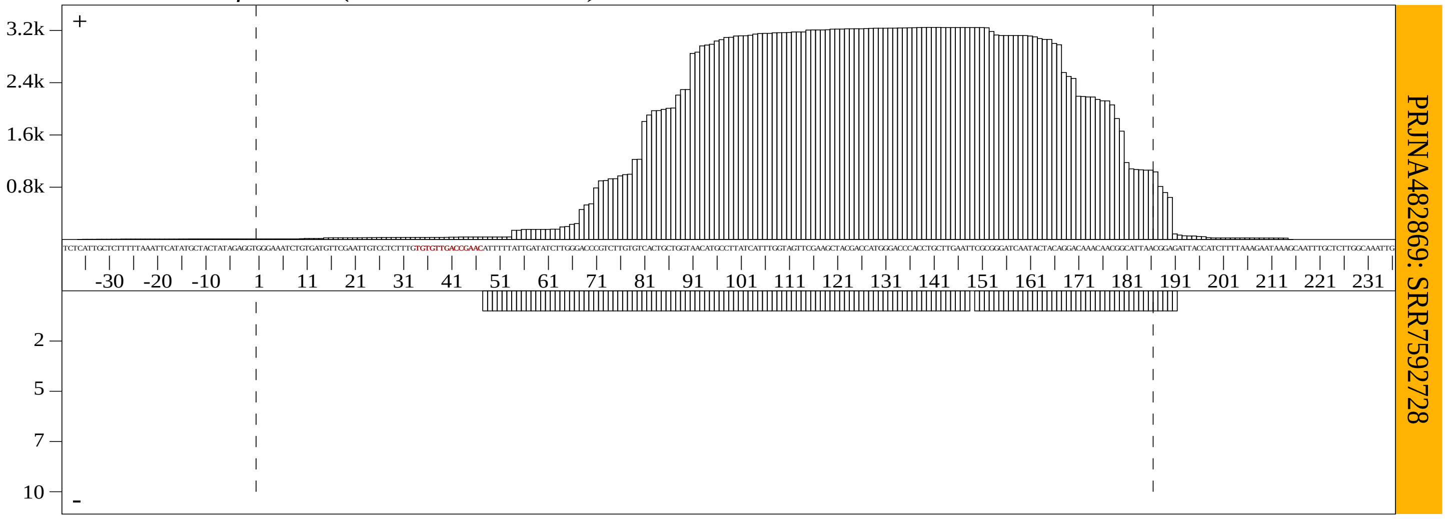


Lib size: 20,068k proposed pRNA: GUUCGGUCAA ...

Note: All reads reverse complemented

# Enterococcaceae

## *Enterococcus faecium (7)*

*Enterococcus faecium (GCF_001298485.1)* NZ_CP012522.1: 1,158,967 .. 1,159,262

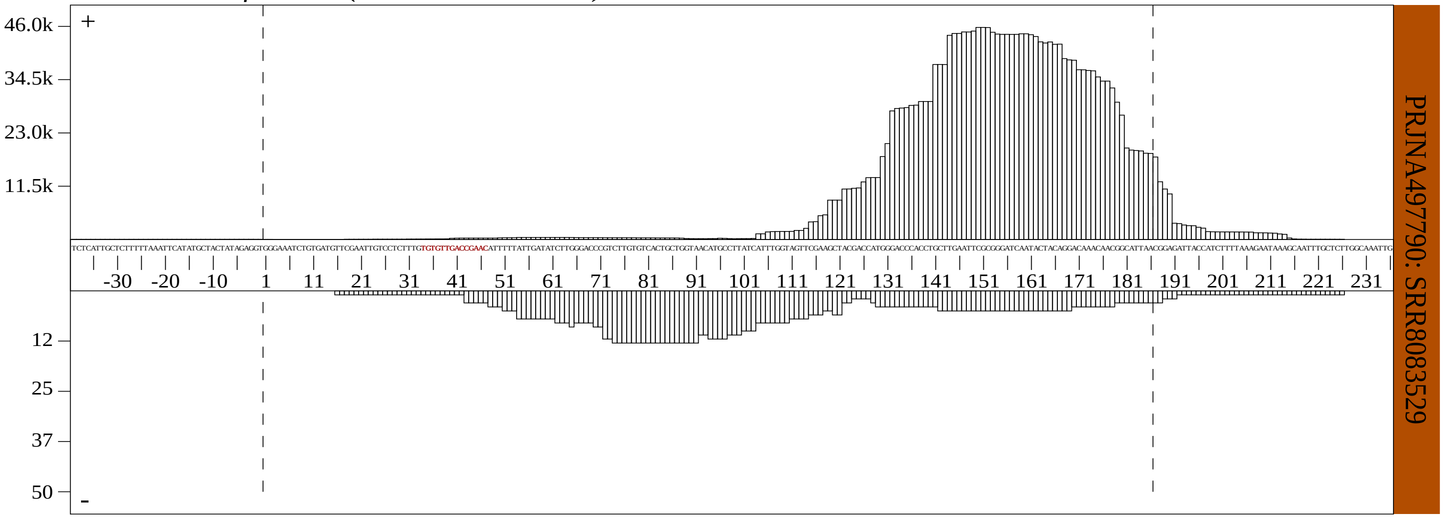


Lib size: 10,939k proposed pRNA: GUUCGGUCAA ...

Note: All reads reverse complemented

# Enterococcaceae

## *Tetragenococcus halophilus (3)*

*Tetragenococcus halophilus (GCF_001712815.1)* NZ_CP012047.1: 2,048,329 .. 2,048,624

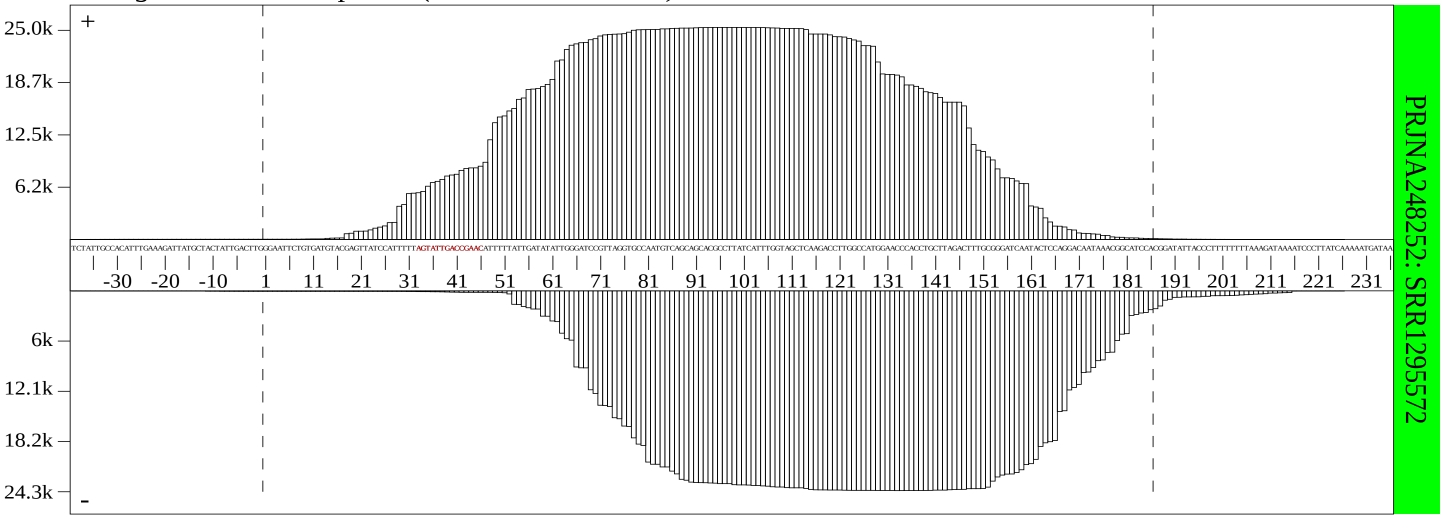


Lib size: 7,954k proposed pRNA: GUUCGGUCAA ...

Note: Looks like strand-independed sequencing

*Tetragenococcus halophilus (GCF_001712815.1)* NZ_CP012047.1: 2,048,329 .. 2,048,624

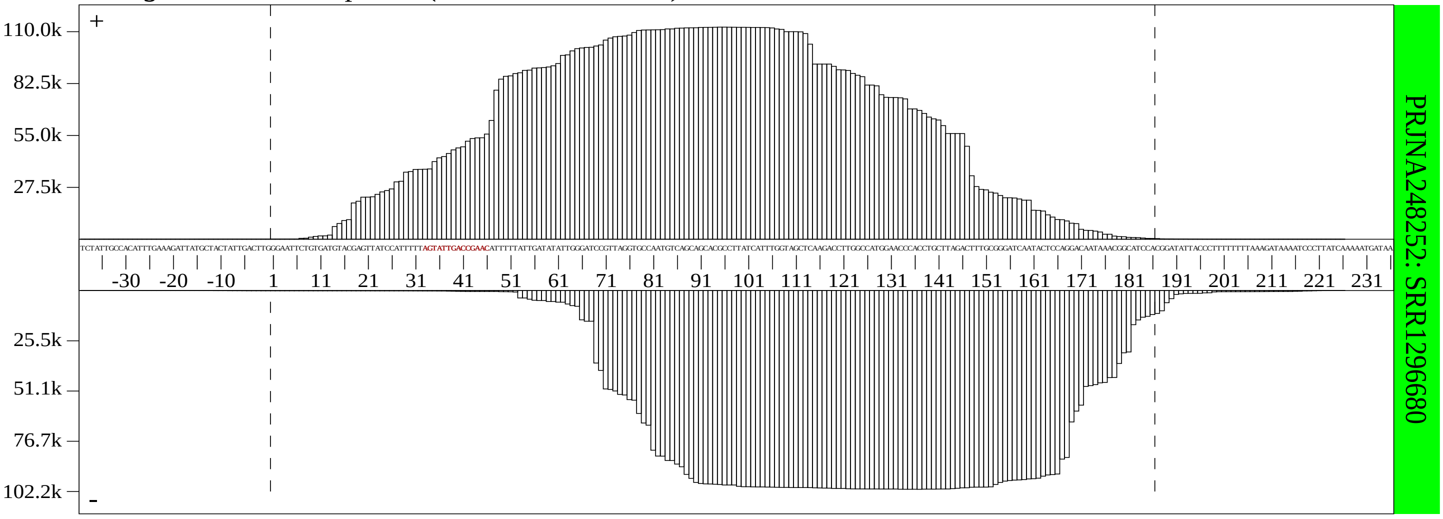


Lib size: 7,671k proposed pRNA: GUUCGGUCAA ...

Note: Looks like strand-independed sequencing

*Tetragenococcus halophilus (GCF_001712815.1)* NZ_CP012047.1: 2,048,329 .. 2,048,624

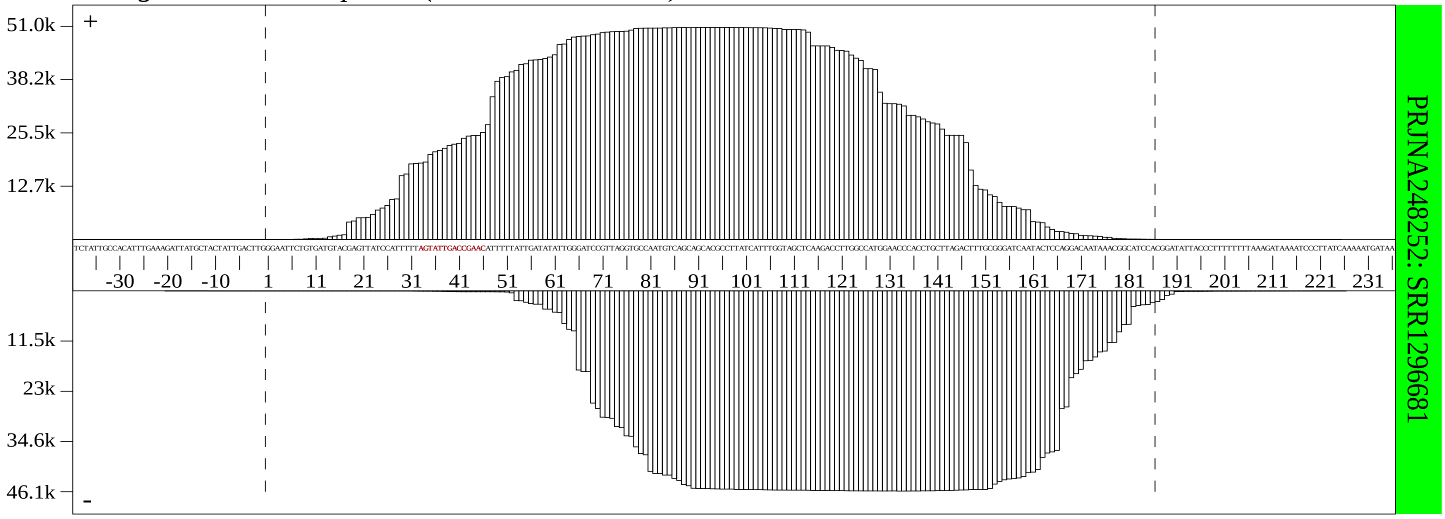


Lib size: 8,264k proposed pRNA: GUUCGGUCAA ...

Note: Looks like strand-independed sequencing

# Lactobacillaceae

## *Lactobacillus acetotolerans (4)*

*Lactobacillus acetotolerans (GCF_001042405.1)* NZ_AP014808.1: 1,081,837 .. 1,082,135

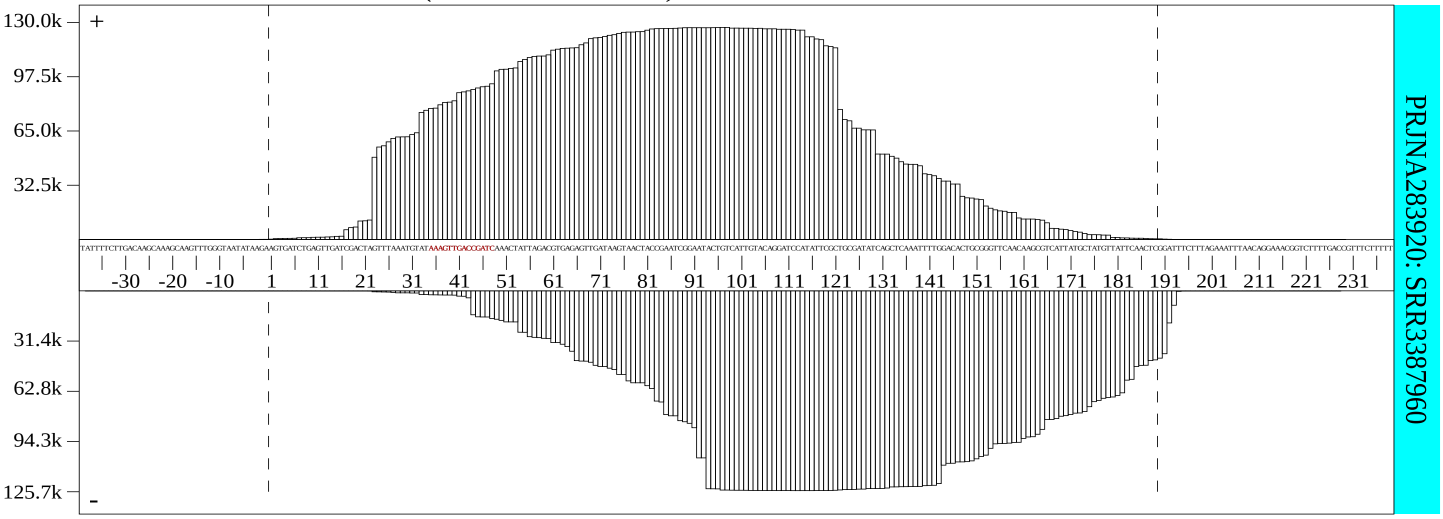


Lib size: 7,763k proposed pRNA: GAUCGGUCAA ...

Note: Looks like strand-independed sequencing

*Lactobacillus acetotolerans (GCF_001042405.1)* NZ_AP014808.1: 1,081,837 .. 1,082,135

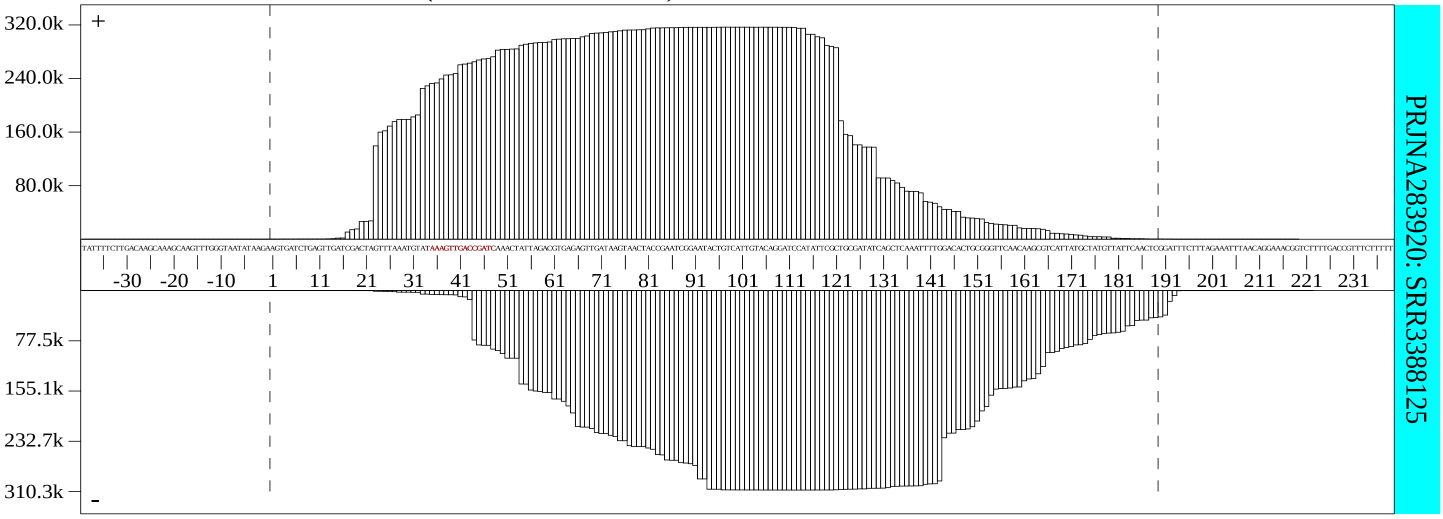


Lib size: 10,800k proposed pRNA: GAUCGGUCAA ...

Note: Looks like strand-independed sequencing

*Lactobacillus acetotolerans (GCF_001042405.1)* NZ_AP014808.1: 1,081,837 .. 1,082,135

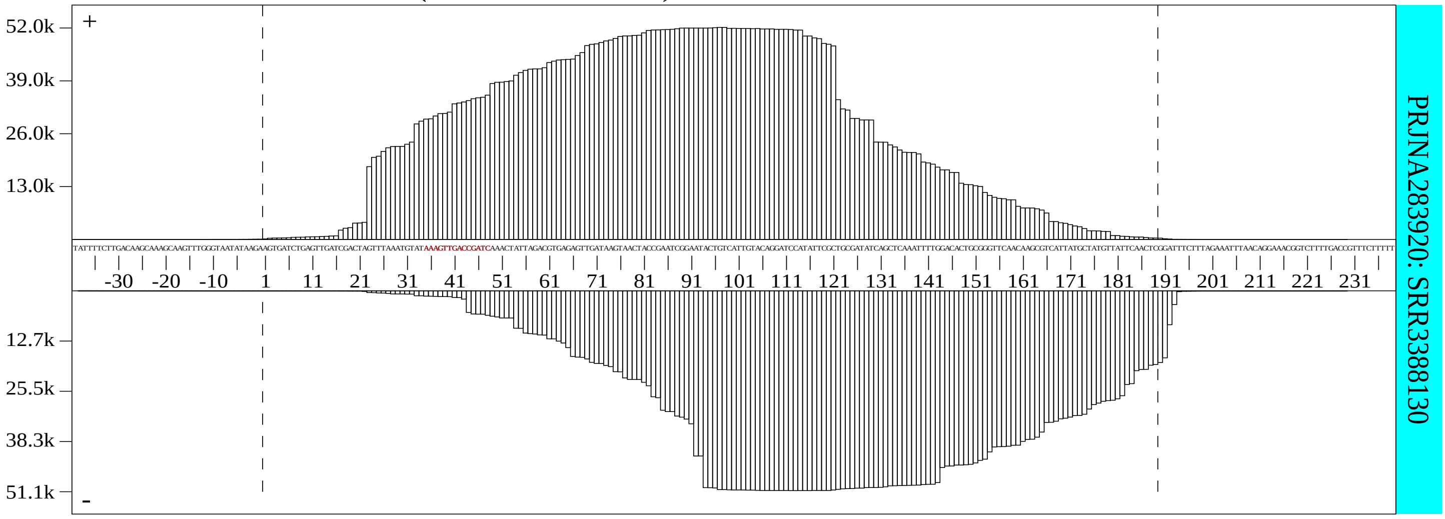


Lib size: 9,229k proposed pRNA: GAUCGGUCAA ...

Note: Looks like strand-independed sequencing

# Lactobacillaceae

## *Lactobacillus acidophilus (7)*

*Lactobacillus acidophilus (GCF_000934625.1)* NZ_CP010432.1: 767,555 .. 767,257

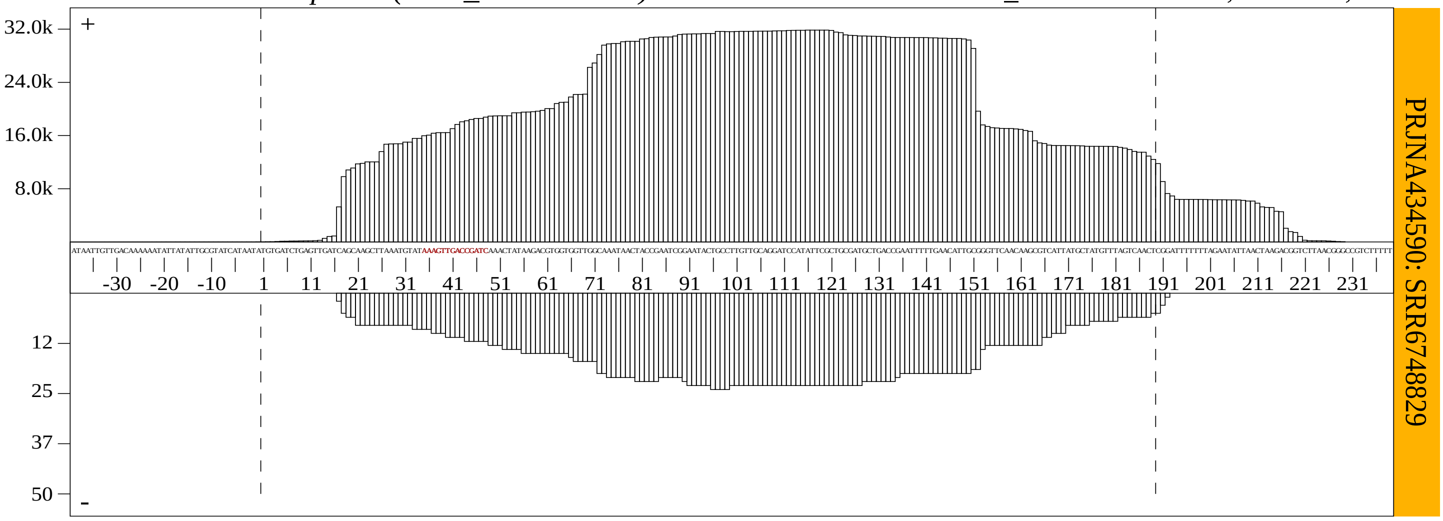


Lib size: 12,429k proposed pRNA: GAUCGGUCAA ...

Note: All reads reverse complemented

*Lactobacillus acidophilus (GCF_000934625.1)* NZ_CP010432.1: 767,555 .. 767,257

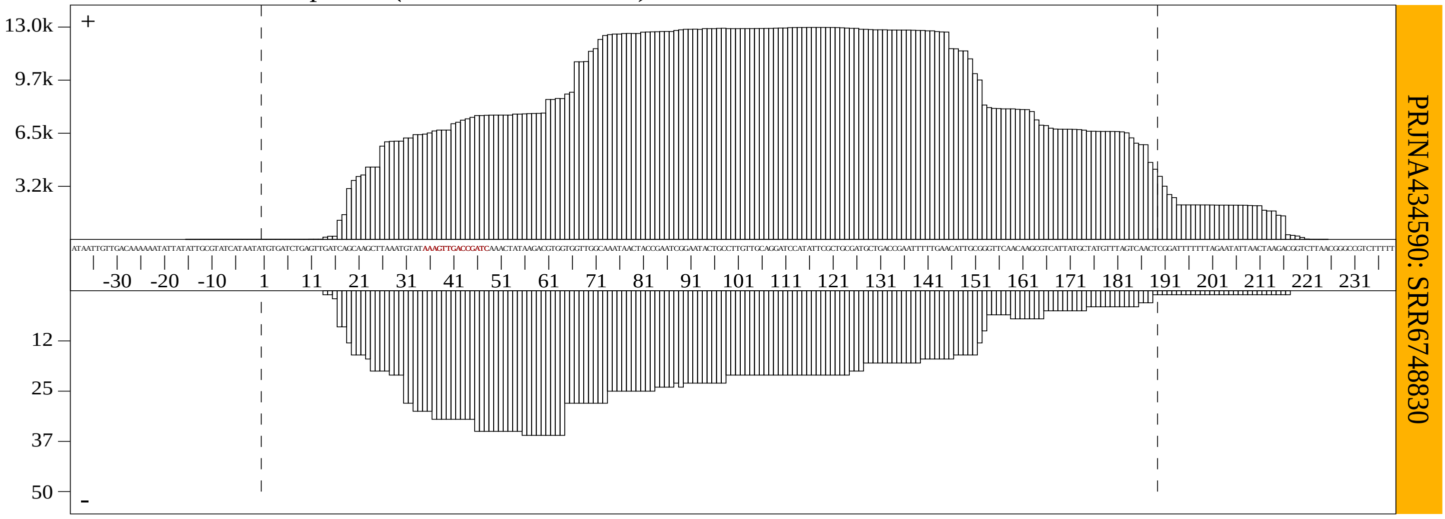


Lib size: 13,431k proposed pRNA: GAUCGGUCAA ...

Note: All reads reverse complemented

*Lactobacillus acidophilus (GCF_000934625.1)* NZ_CP010432.1: 767,555 .. 767,257

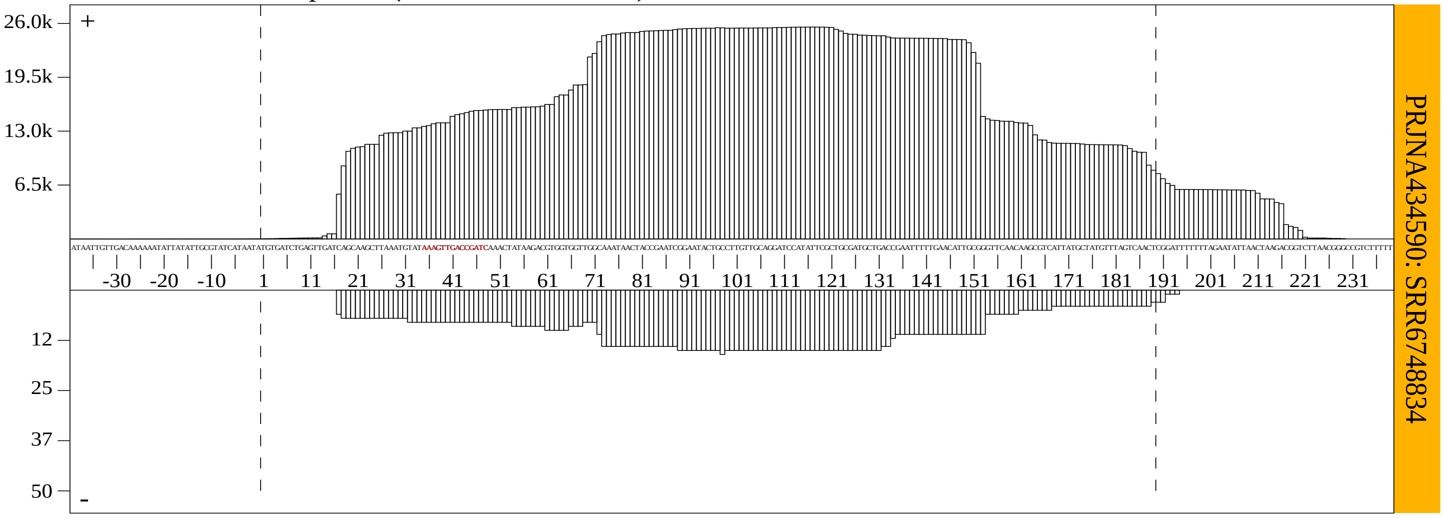


Lib size: 12,189k proposed pRNA: GAUCGGUCAA ...

Note: All reads reverse complemented

# Lactobacillaceae

## *Lactobacillus acidophilus (7)*

*Lactobacillus acidophilus (GCF_000934625.1)* NZ_CP010432.1: 767,555 .. 767,257

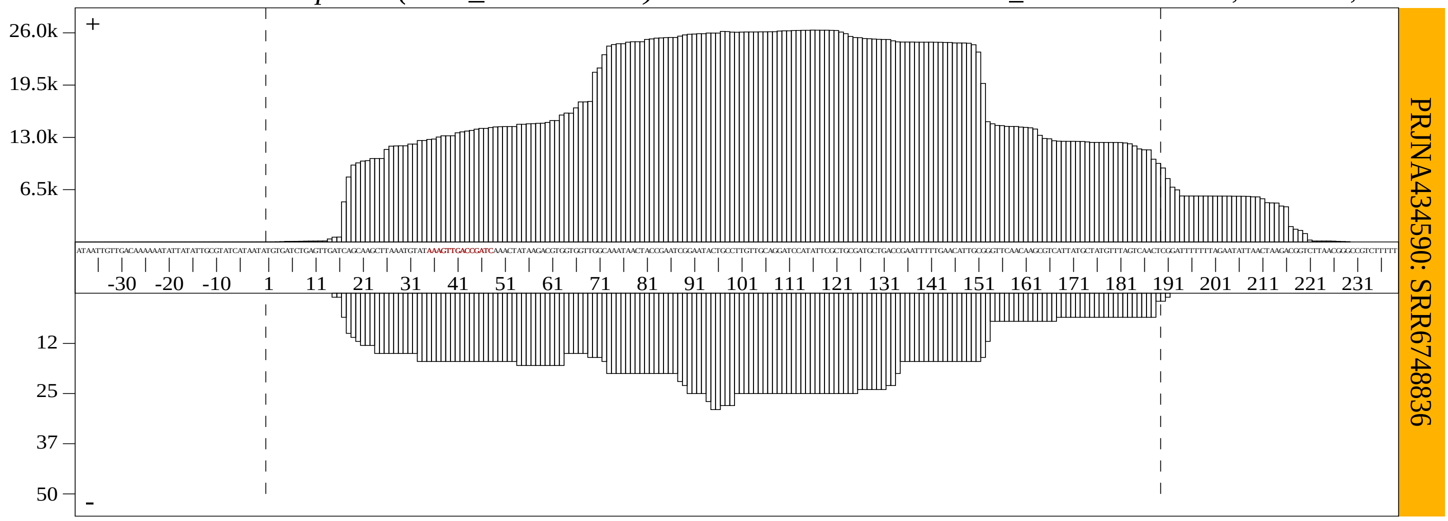

Lib size: 12,550k proposed pRNA: GAUCGGUCAA ...

Note: All reads reverse complemented

*Lactobacillus acidophilus (GCF_000934625.1)* NZ_CP010432.1: 767,555 .. 767,257

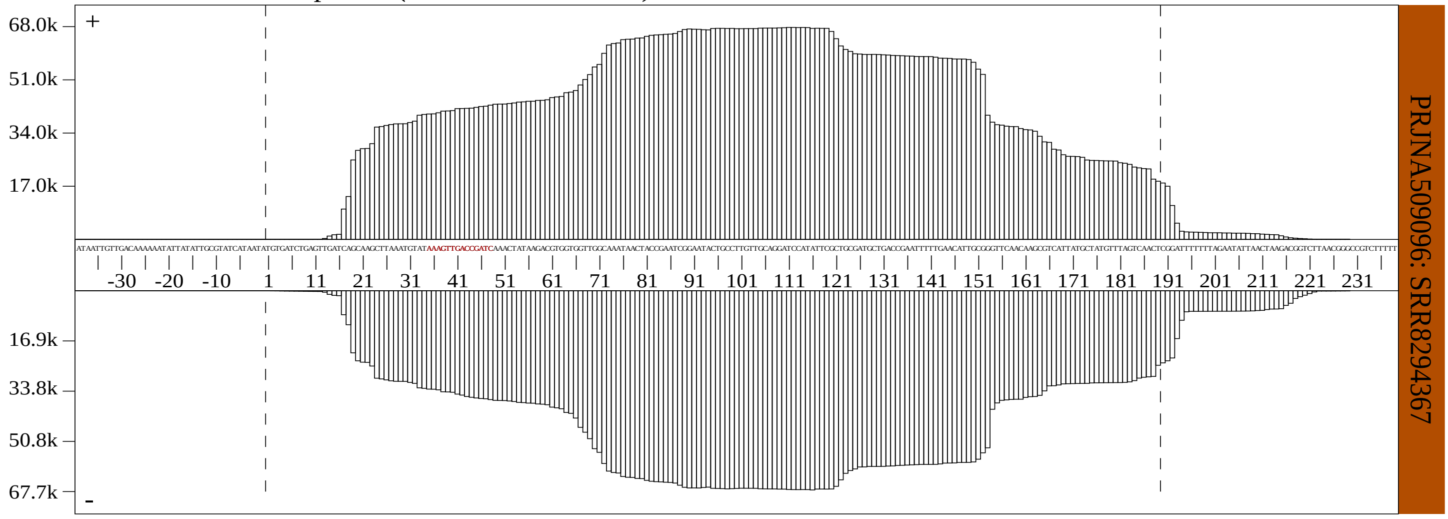

Lib size: 7,199k proposed pRNA: GAUCGGUCAA ...

Note: Looks like strand-independed sequencing

*Lactobacillus acidophilus (GCF_000934625.1)* NZ_CP010432.1: 767,555 .. 767,257

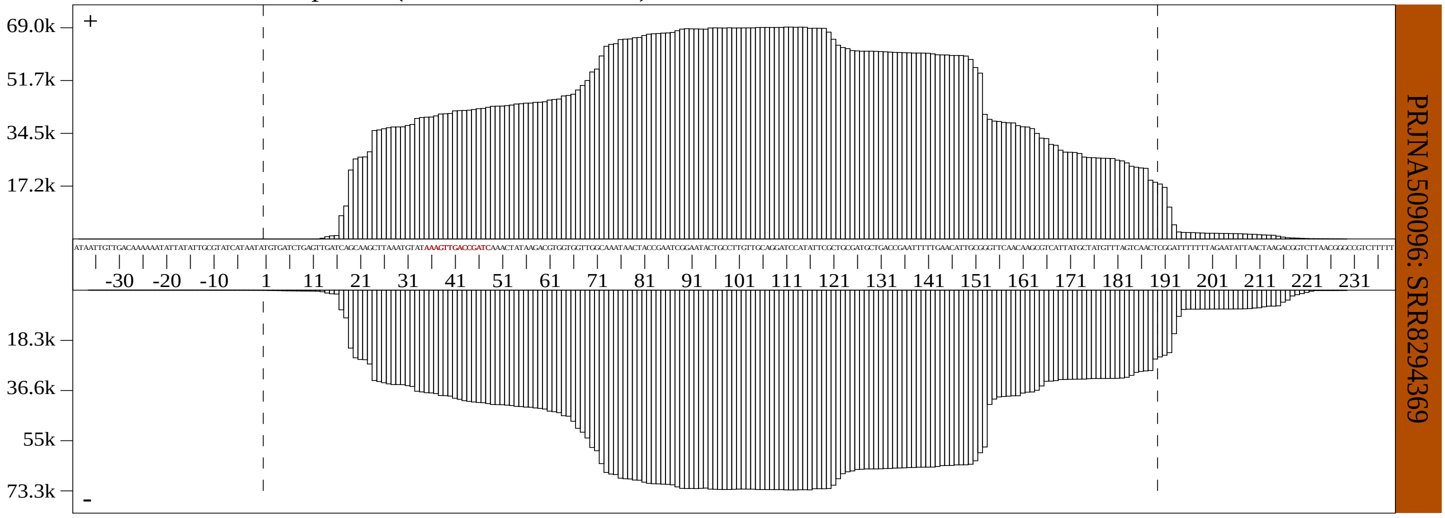

Lib size: 7,826k proposed pRNA: GAUCGGUCAA ...

Note: Looks like strand-independed sequencing

# Lactobacillaceae

## *Lactobacillus acidophilus (7)*

*Lactobacillus acidophilus (GCF_000934625.1)* NZ_CP010432.1: 767,555 .. 767,257

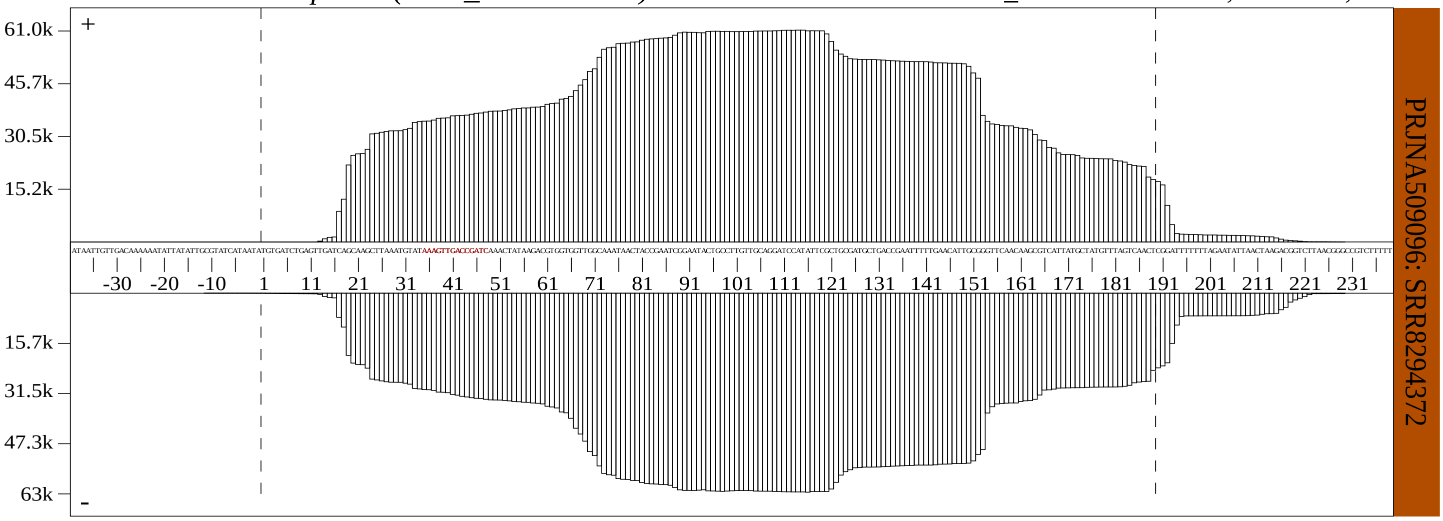


Lib size: 6,780k proposed | pRNA: GAUCGGUCAA ...

Note: Looks like strand-independed sequencing

# Lactobacillaceae

## *Lactobacillus amylovorus DSM 20531 (1)*

*Lactobacillus amylovorus (GCF_002706375.1)* NZ_CP017706.1: 1,874,791 .. 1,874,494

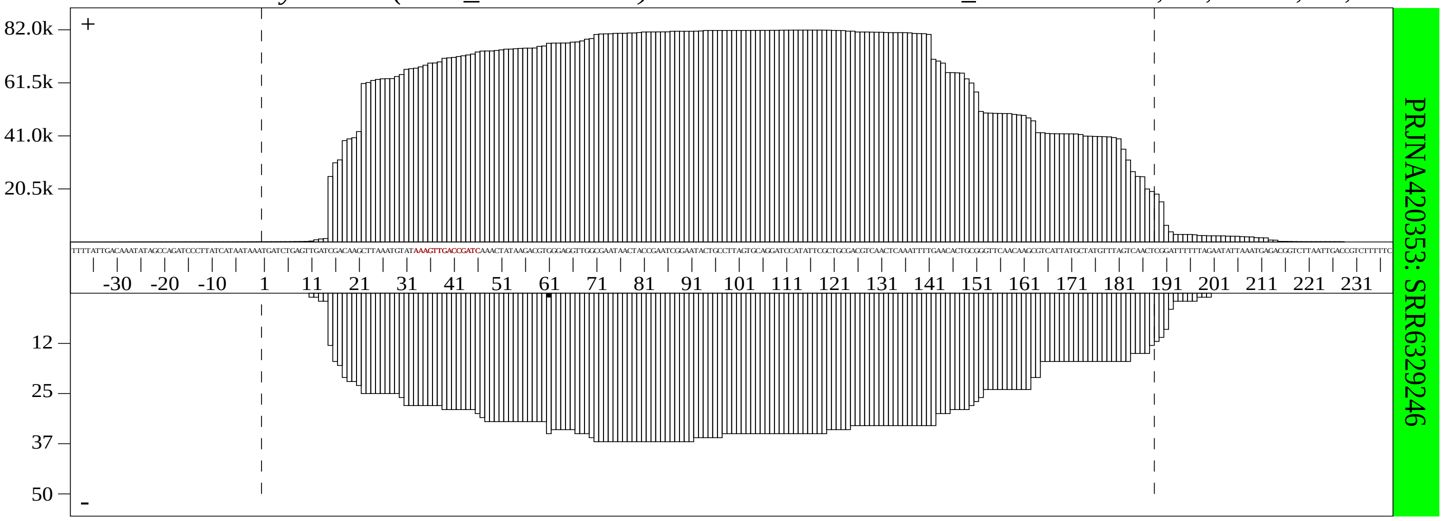


Lib size: 10,249k proposed pRNA: GAUCGGUCAA ...

Note: All reads reverse complemented

# Lactobacillaceae

## *Lactobacillus amylovorus (2)*

*Lactobacillus amylovorus (GCF_000191545.1)* NC_015214.1: 804,755 .. 804,457

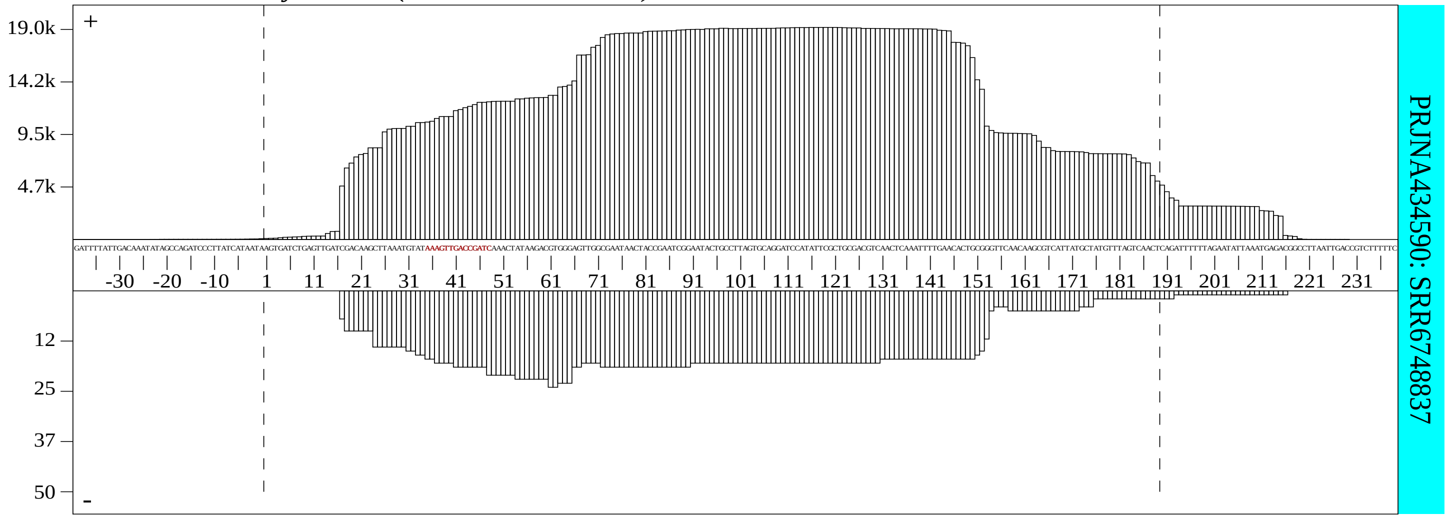


Lib size: 13,400k proposed pRNA: GAUCGGUCAA ...

Note: All reads reverse complemented

*Lactobacillus amylovorus (GCF_000191545.1)* NC_015214.1: 804,755 .. 804,457

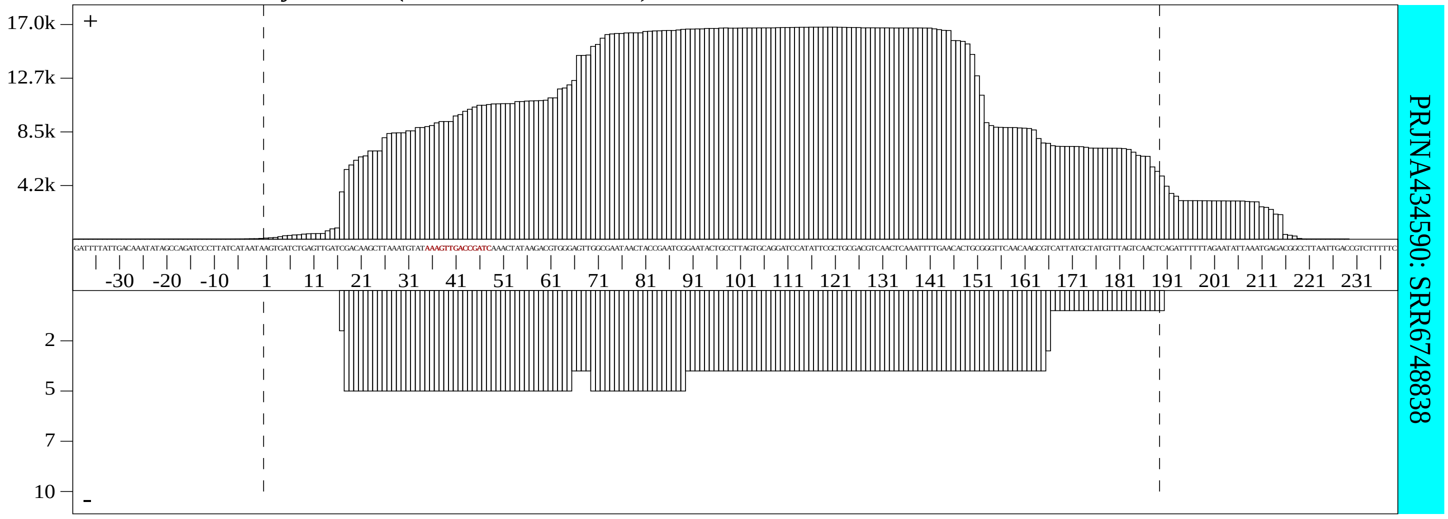


Lib size: 11,923k proposed pRNA: GAUCGGUCAA ...

Note: All reads reverse complemented

# Lactobacillaceae

## *Lactobacillus crispatus ST1 (3)*

*Lactobacillus crispatus (GCF_000091765.1)* NC_014106.1: 779,036 .. 778,737

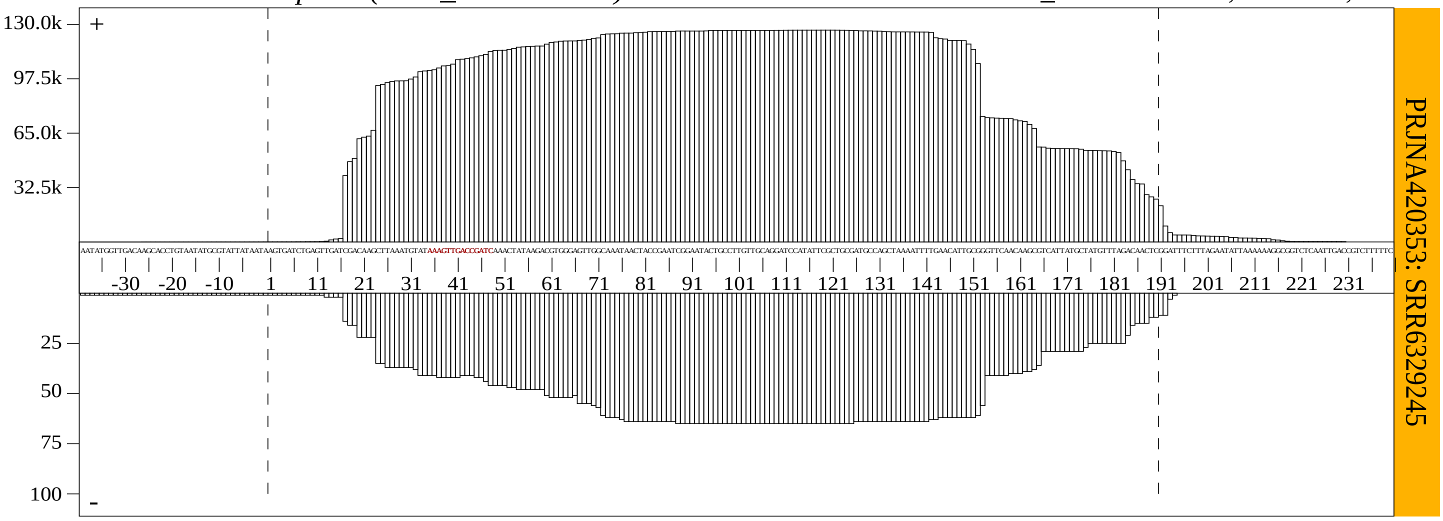


Lib size: 9,395k proposed pRNA: GAUCGGUCAA ...

Note: All reads reverse complemented

*Lactobacillus crispatus (GCF_000091765.1)* NC_014106.1: 779,036 .. 778,737

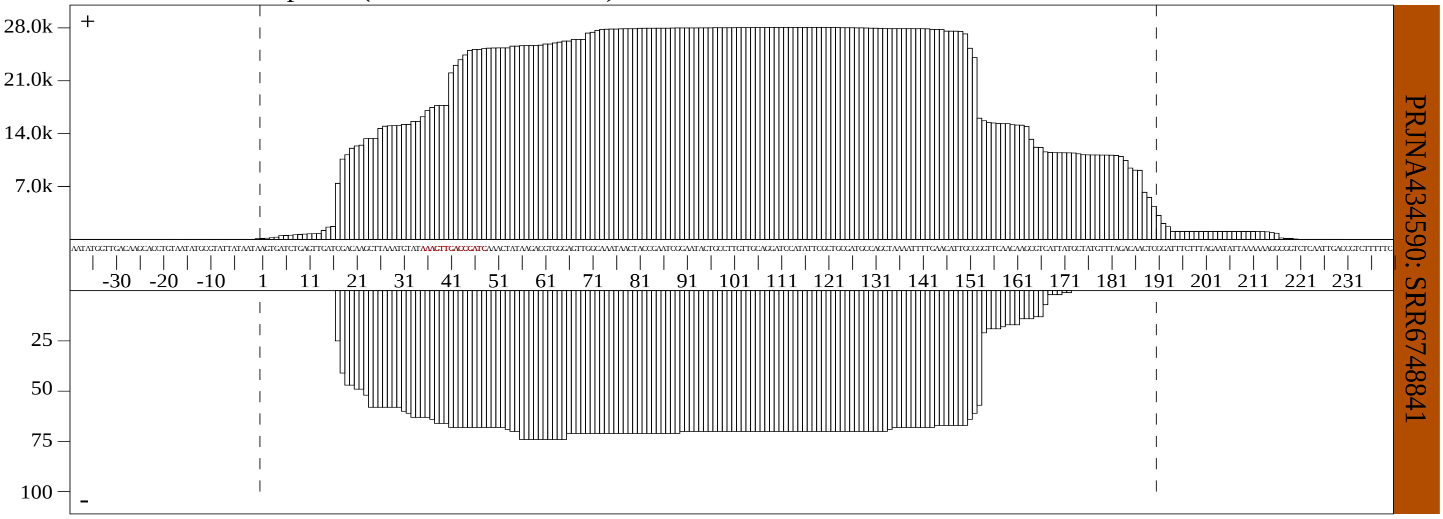


Lib size: 7,408k proposed pRNA: GAUCGGUCAA ...

Note: All reads reverse complemented

*Lactobacillus crispatus (GCF_000091765.1)* NC_014106.1: 779,036 .. 778,737

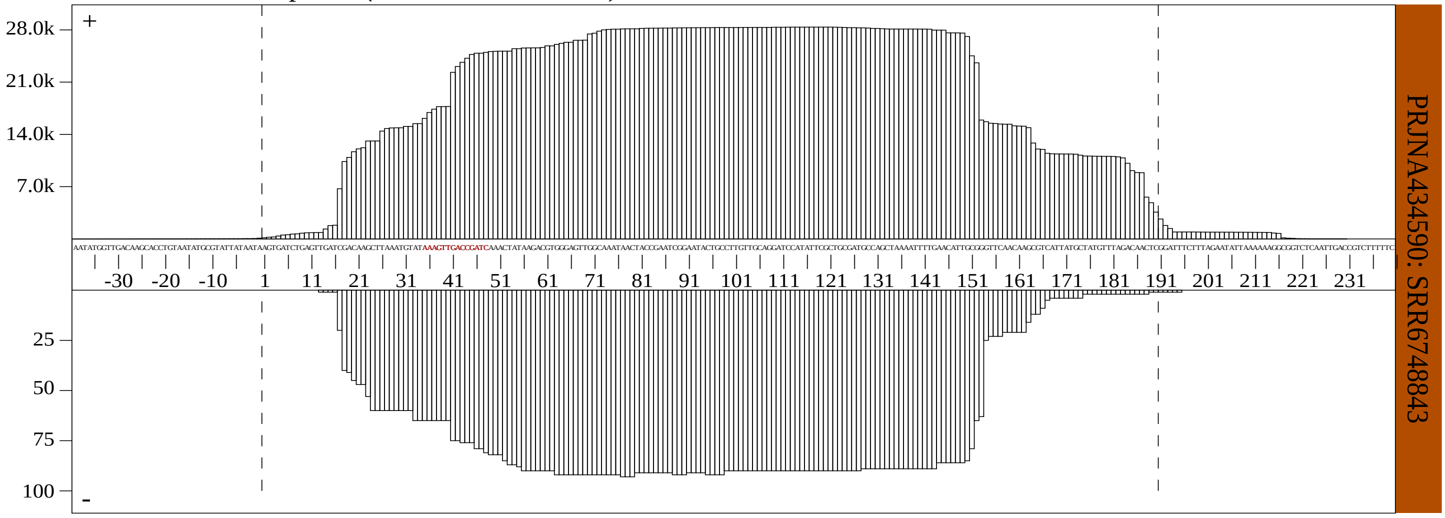


Lib size: 8,610k proposed pRNA: GAUCGGUCAA ...

Note: All reads reverse complemented

# Lactobacillaceae

## *Lactobacillus delbrueckii subsp bulgaricus 2038 (2)*

*Lactobacillus delbrueckii (GCF_000191165.1)* NC_017469.1: 600,914 .. 600,616

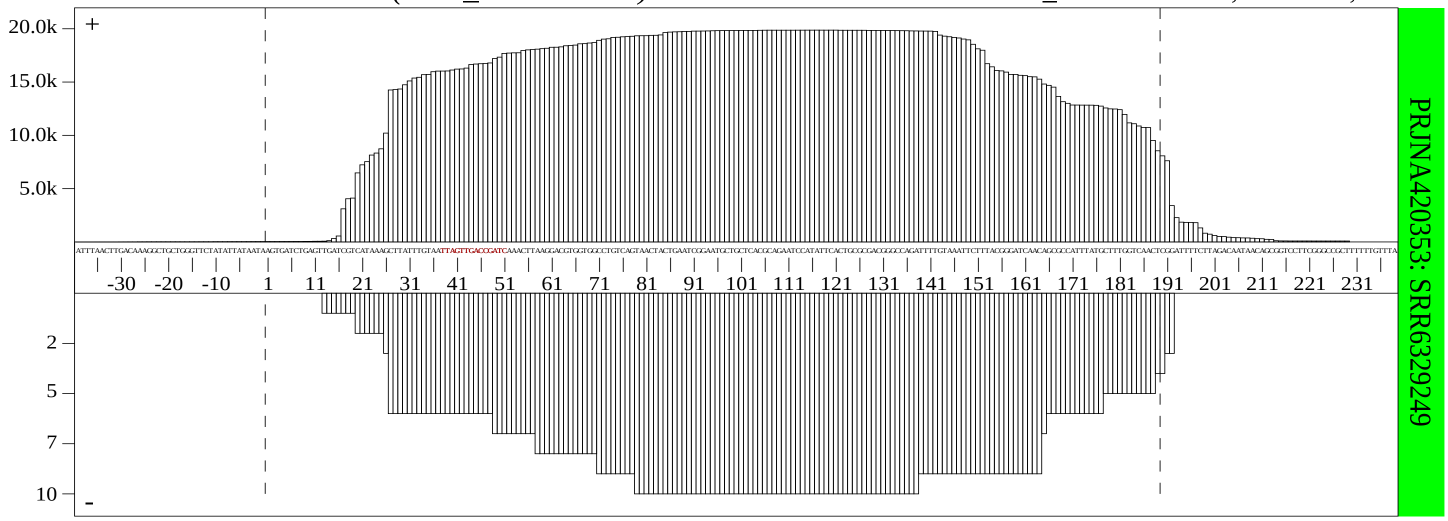


Lib size: 9,515k proposed pRNA: GAUCGGUCAA ...

Note: All reads reverse complemented

*Lactobacillus delbrueckii (GCF_000191165.1)* NC_017469.1: 600,914 .. 600,616

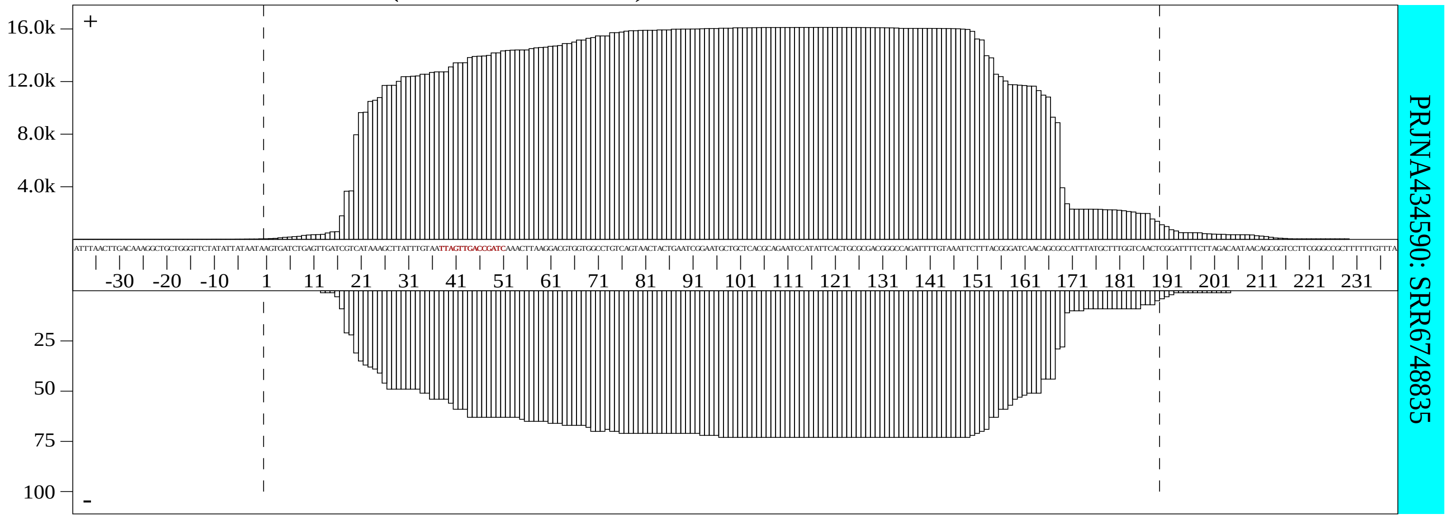


Lib size: 7,780k proposed pRNA: GAUCGGUCAA ...

Note: All reads reverse complemented

# Lactobacillaceae

## *Lactobacillus gasseri (2)*

*Lactobacillus gasseri (GCF_002158885.1)* NZ_CP021427.1: 1,628,848 .. 1,628,561

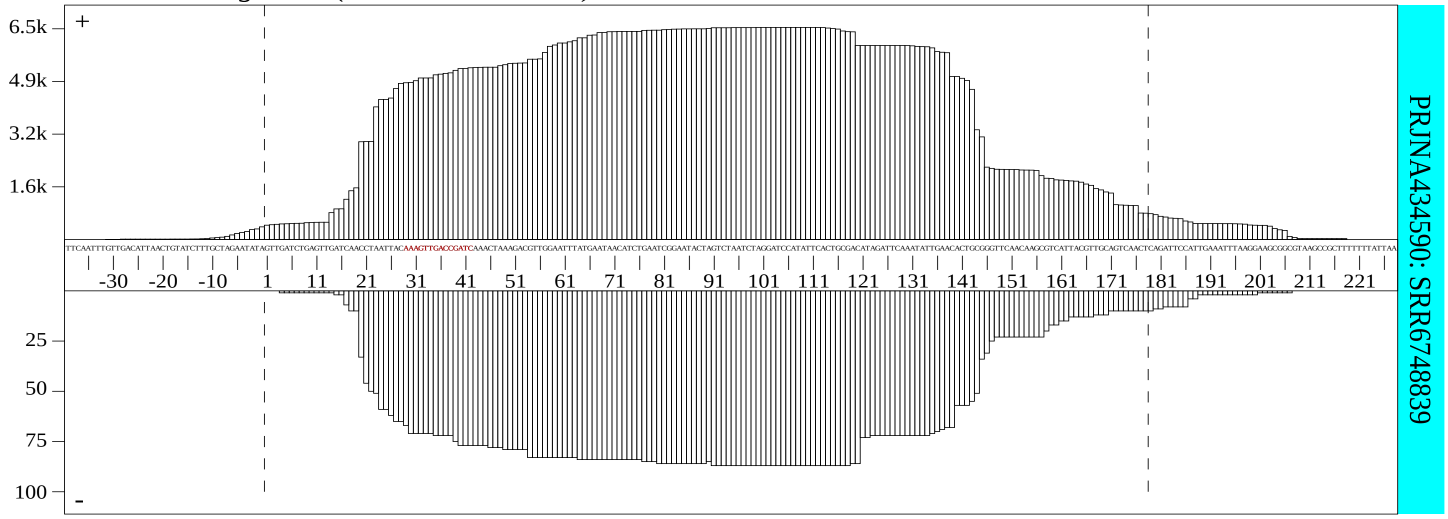


Lib size: 9,169k proposed pRNA: GAUCGGUCAA ...

Note: All reads reverse complemented

*Lactobacillus gasseri (GCF_002158885.1)* NZ_CP021427.1: 1,628,848 .. 1,628,561

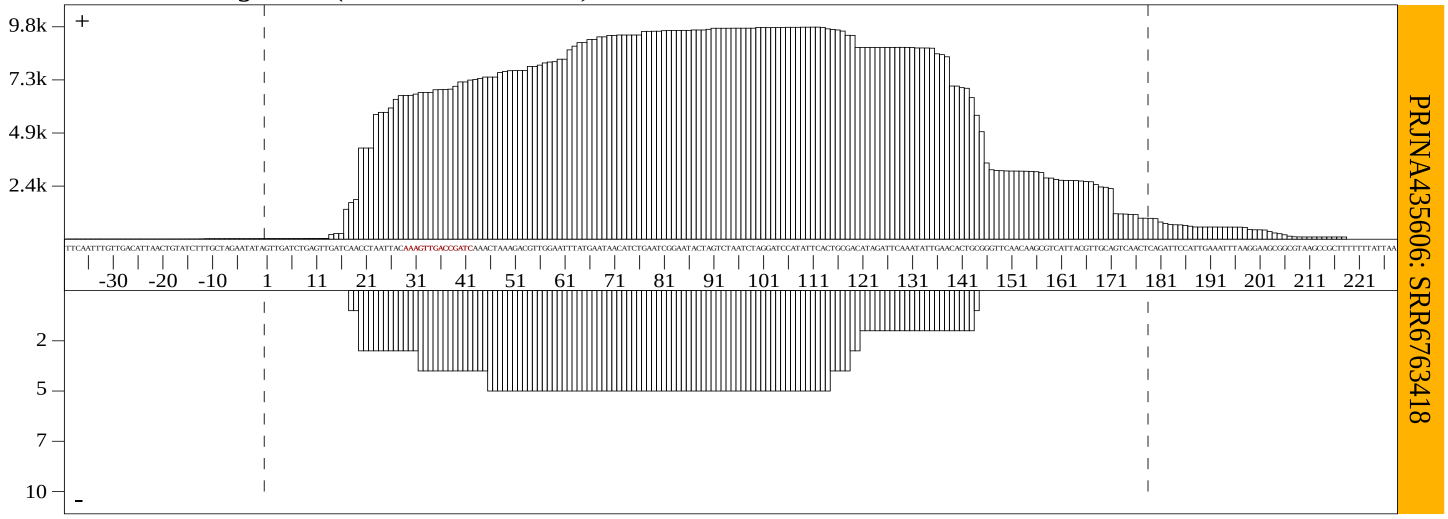


Lib size: 7,906k proposed pRNA: GAUCGGUCAA ...

Note: All reads reverse complemented

# Lactobacillaceae

## *Lactobacillus helveticus CNRZ32 (2)*

*Lactobacillus helveticus (GCF_000422165.1)* NC_021744.1: 898,619 .. 898,322

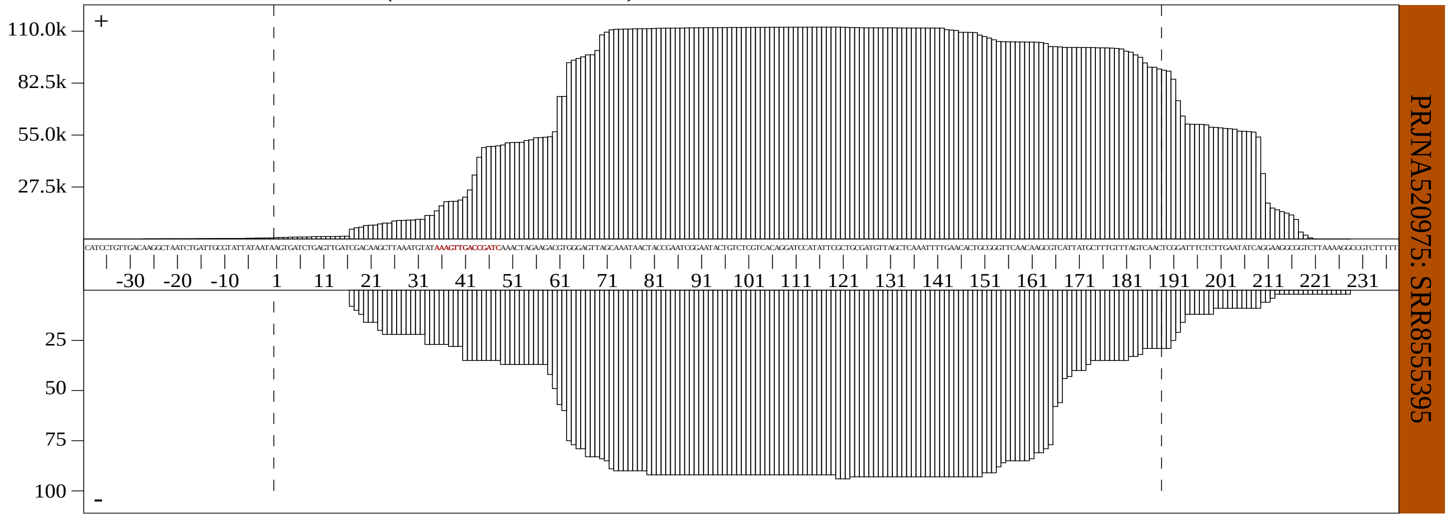


Lib size: 31,680k

Note: All reads reverse complemented

proposed pRNA: GAUCGGUCAA ...

*Lactobacillus helveticus (GCF_000422165.1)* NC_021744.1: 898,619 .. 898,322

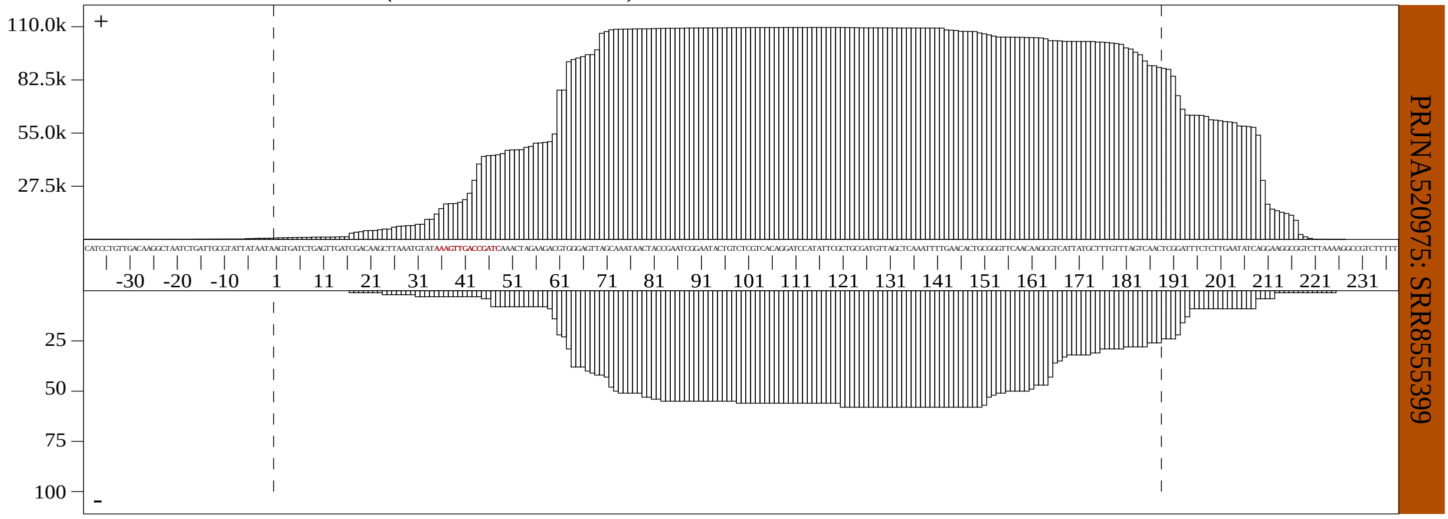


Lib size: 31,162k

Note: All reads reverse complemented

proposed pRNA: GAUCGGUCAA ...

# Lactobacillaceae

## *Lactobacillus helveticus (3)*

*Lactobacillus helveticus (GCF_000961015.1)* NZ_CP009907.1: 1,566,532 .. 1,566,829

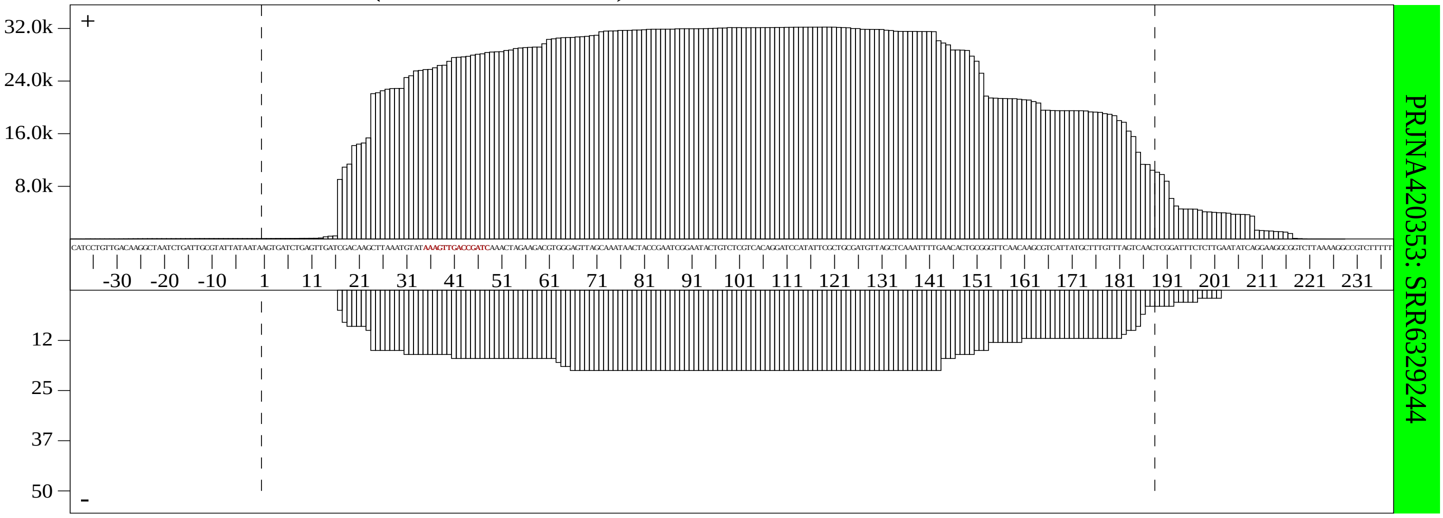


Lib size: 8,835k proposed pRNA: GAUCGGUCAA ...

Note: All reads reverse complemented

*Lactobacillus helveticus (GCF_000961015.1)* NZ_CP009907.1: 1,566,532 .. 1,566,829

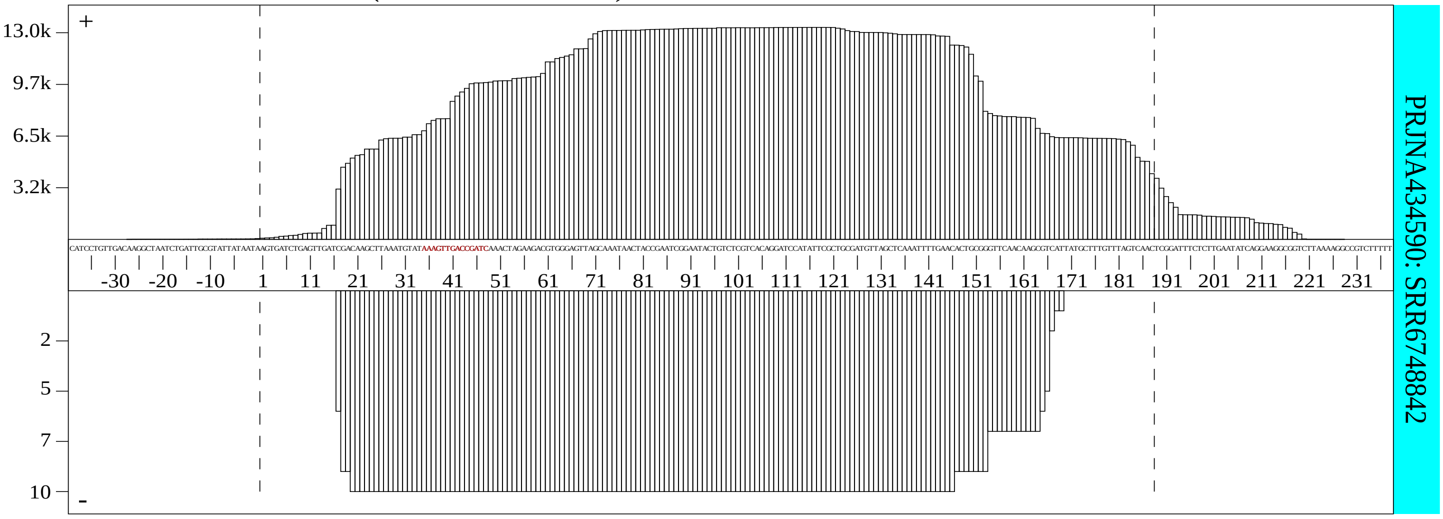


Lib size: 9,832k proposed pRNA: GAUCGGUCAA ...

Note: All reads reverse complemented

*Lactobacillus helveticus (GCF_000961015.1)* NZ_CP009907.1: 1,566,532 .. 1,566,829

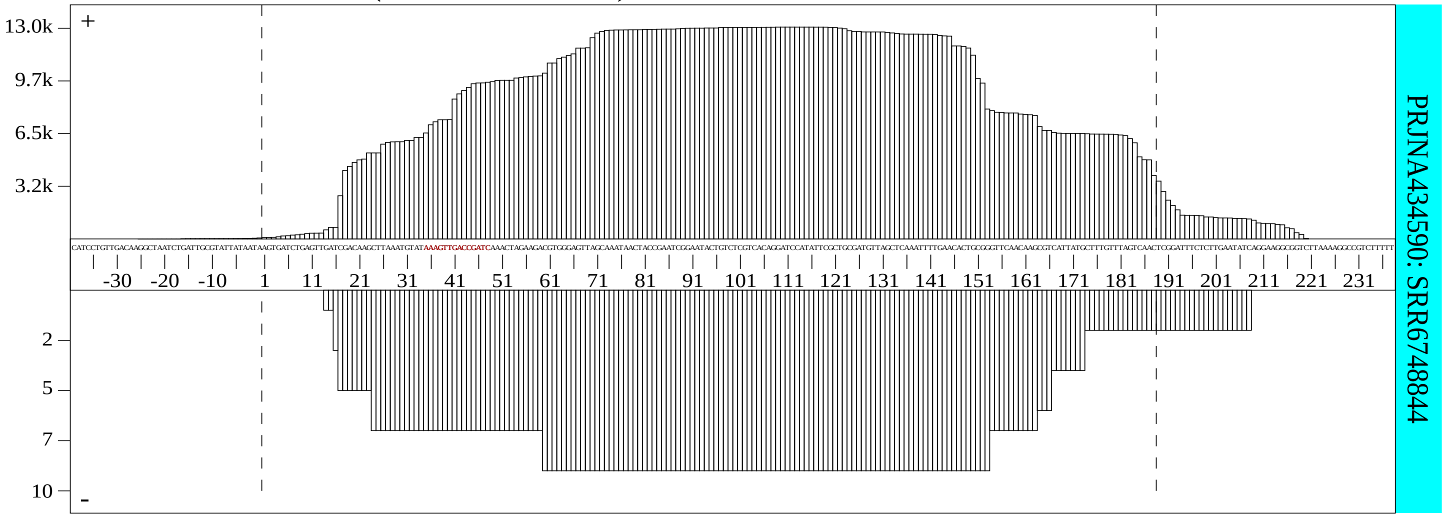


Lib size: 7,892k proposed pRNA: GAUCGGUCAA ...

Note: All reads reverse complemented

# Lactobacillaceae

## *Lactobacillus paracasei (4)*

*Lactobacillus paracasei (GCF_001191565.1)* NZ_CP012187.1: 1,121,832 .. 1,121,546

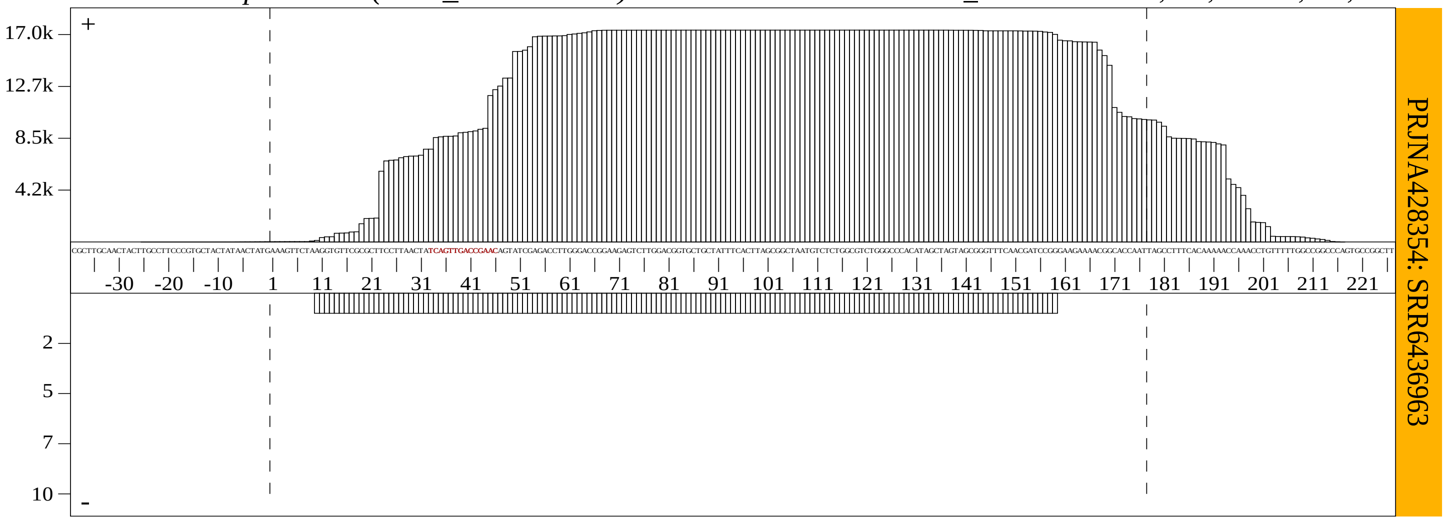


Lib size: 5,546k proposed pRNA: GUUCGGUCAA ...

Note: All reads reverse complemented

*Lactobacillus paracasei (GCF_001191565.1)* NZ_CP012187.1: 1,121,832 .. 1,121,546

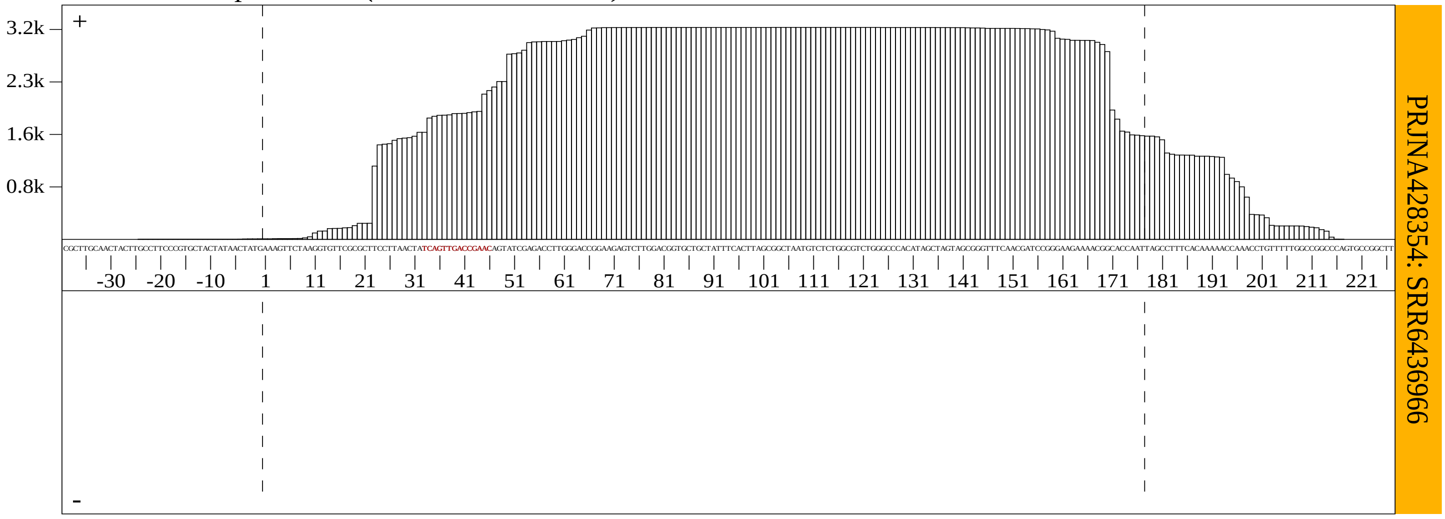


Lib size: 5,101k proposed pRNA: GUUCGGUCAA ...

Note: All reads reverse complemented No reads on - strand

*Lactobacillus paracasei (GCF_001191565.1)* NZ_CP012187.1: 1,121,832 .. 1,121,546

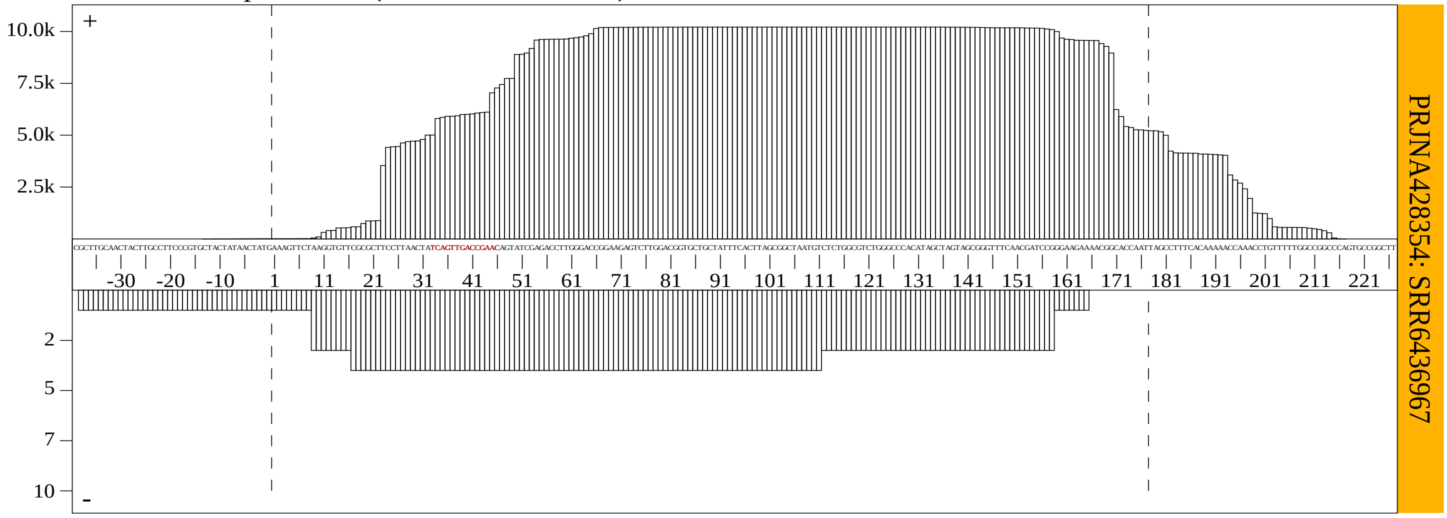


Lib size: 5,493k proposed pRNA: GUUCGGUCAA ...

Note: All reads reverse complemented

# Lactobacillaceae

## *Lactobacillus paracasei (4)*

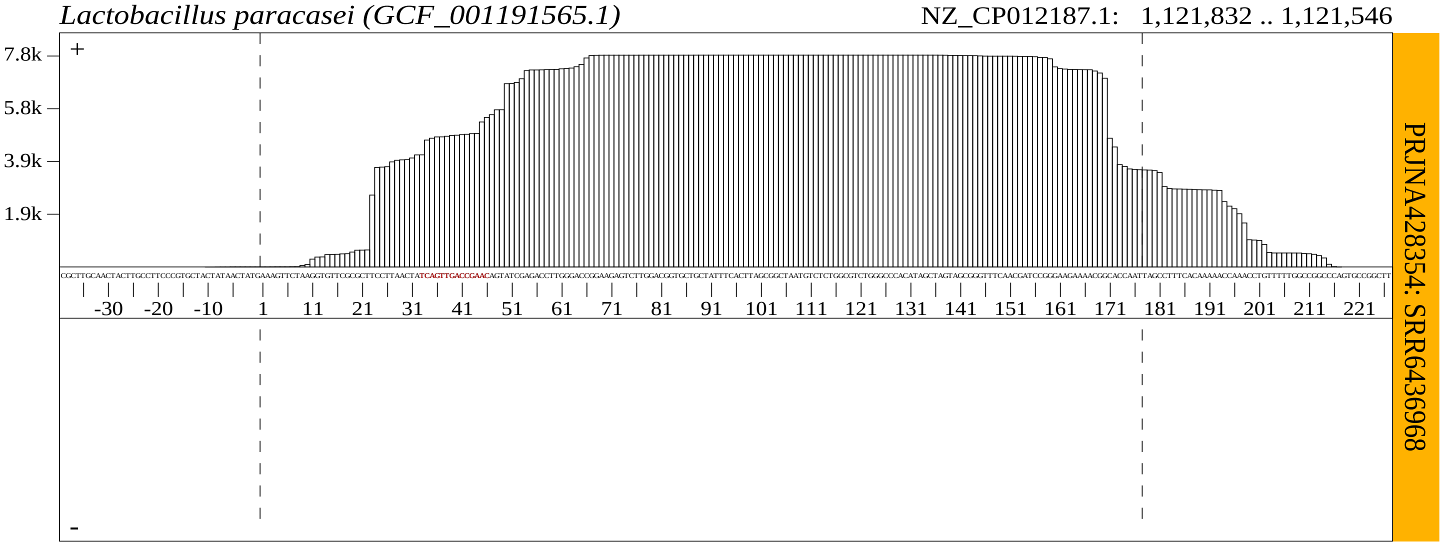


Lib size: 4,952k

Note: All reads reverse complemented

proposed pRNA: GUUCGGUCAA ...

No reads on - strand

# Lactobacillaceae

## *Lactobacillus plantarum (8)*

*Lactobacillus plantarum (GCF_000931425.2)* NZ_CP010528.1: 2,138,238 .. 2,138,525

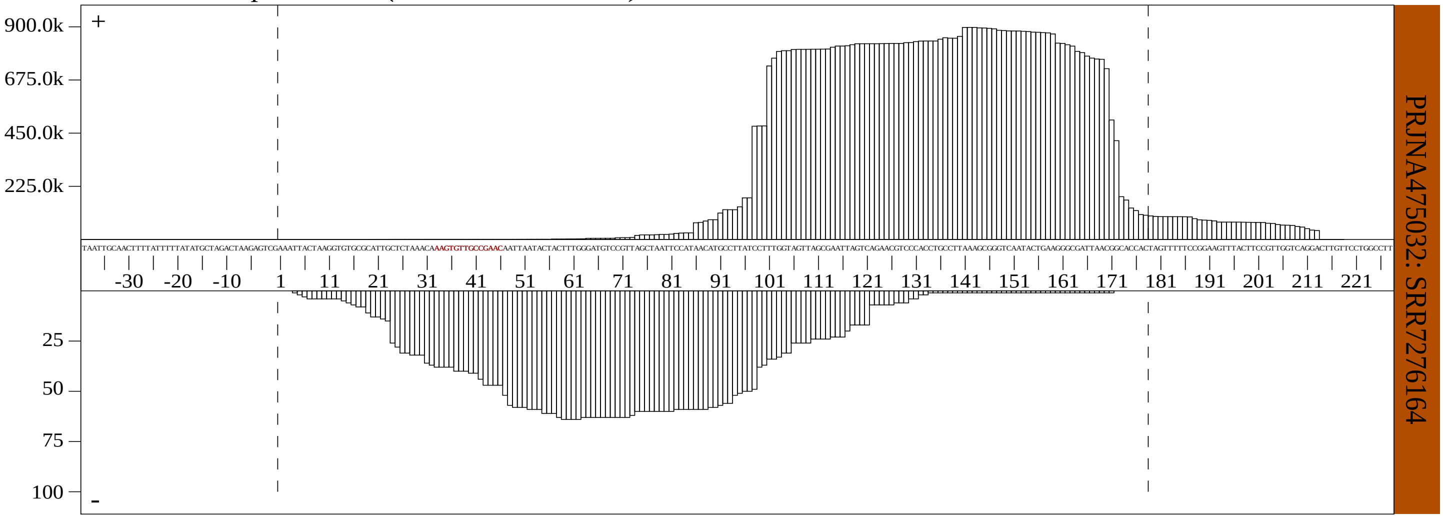


Lib size: 25,945k proposed pRNA: GUUCGGCAAC ...

Note: All reads reverse complemented

*Lactobacillus plantarum (GCF_000931425.2)* NZ_CP010528.1: 2,138,238 .. 2,138,525

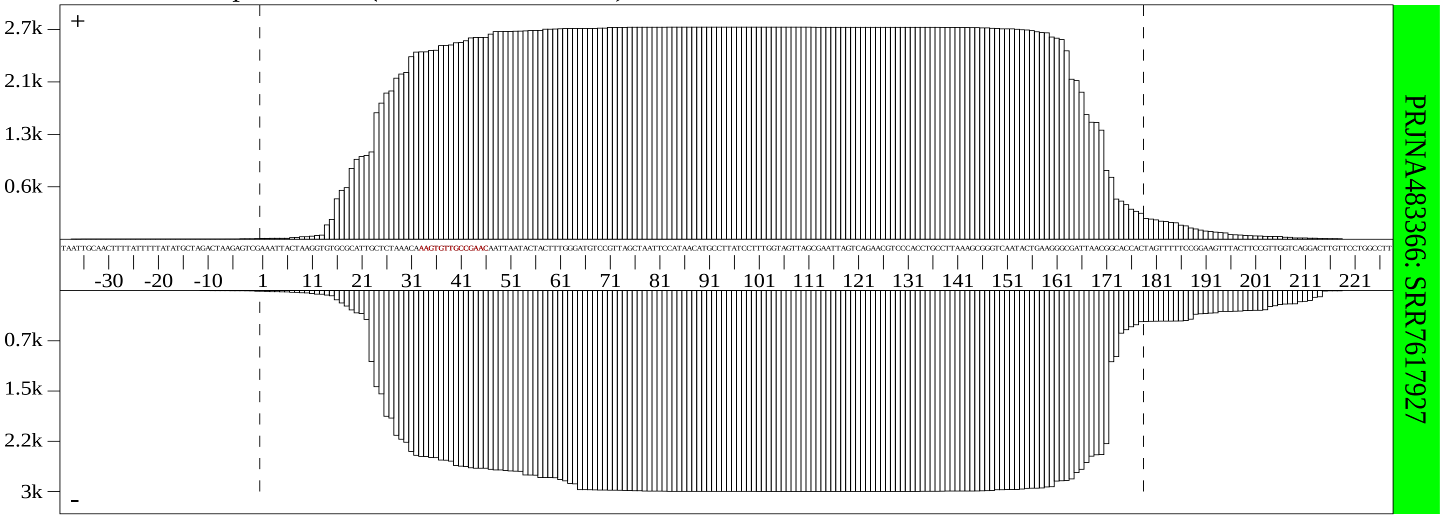


Lib size: 7,803k proposed pRNA: GUUCGGCAAC ...

Note: Looks like strand-independed sequencing

*Lactobacillus plantarum (GCF_000931425.2)* NZ_CP010528.1: 2,138,238 .. 2,138,525

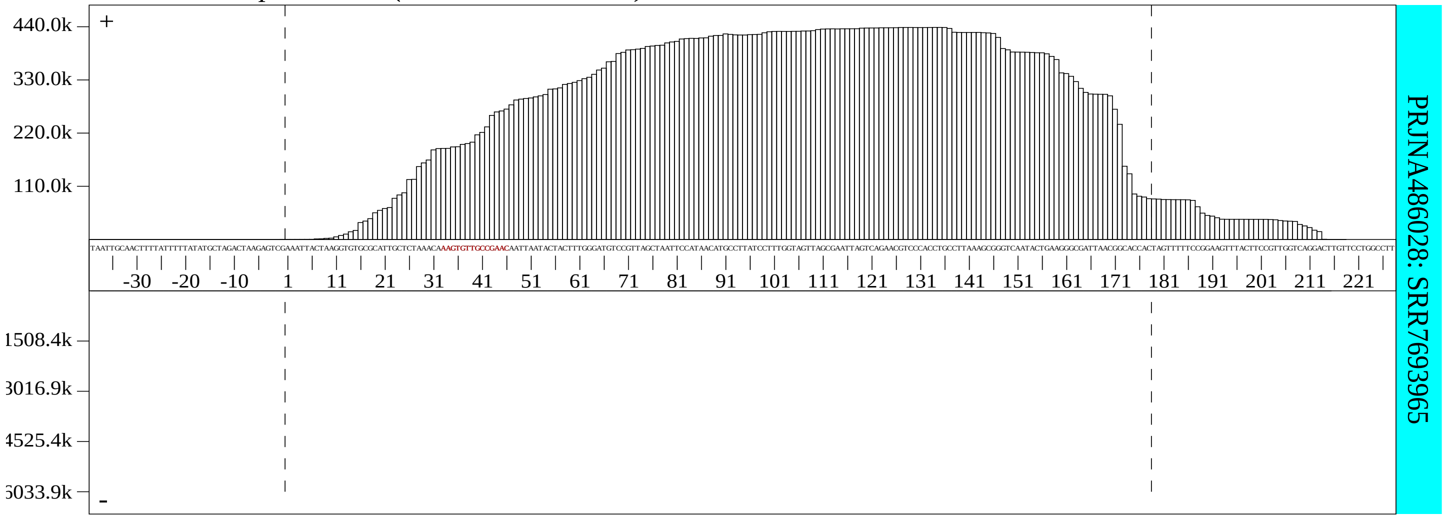


Lib size: 25,490k proposed pRNA: GUUCGGCAAC ...

Note: All reads reverse complemented

# Lactobacillaceae

## *Lactobacillus reuteri (1)*

*Lactobacillus reuteri (GCF_001046835.1)* NZ_CP011024.1: 550,646 .. 550,359

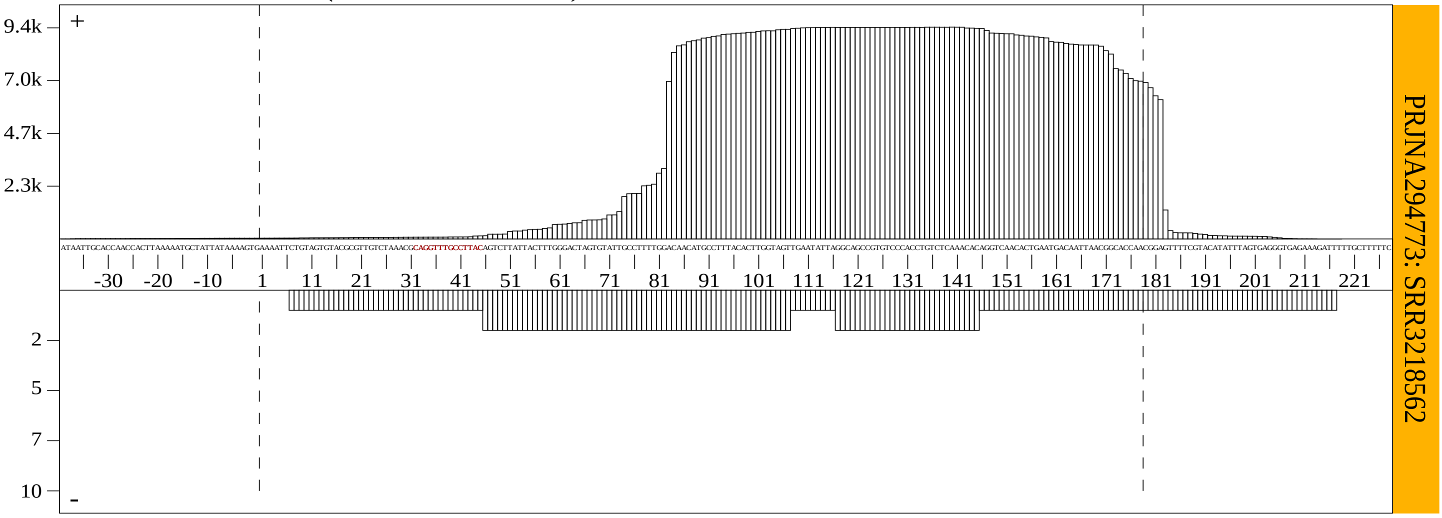


Lib size: 14,457k

proposed pRNA: GUAAGGCAAA ...

Note: All reads reverse complemented

# Lactobacillaceae

## *Lactobacillus rhamnosus (6)*

*Lactobacillus rhamnosus (GCF_001988935.1)* NZ_CP014201.1: 371,828 .. 371,542

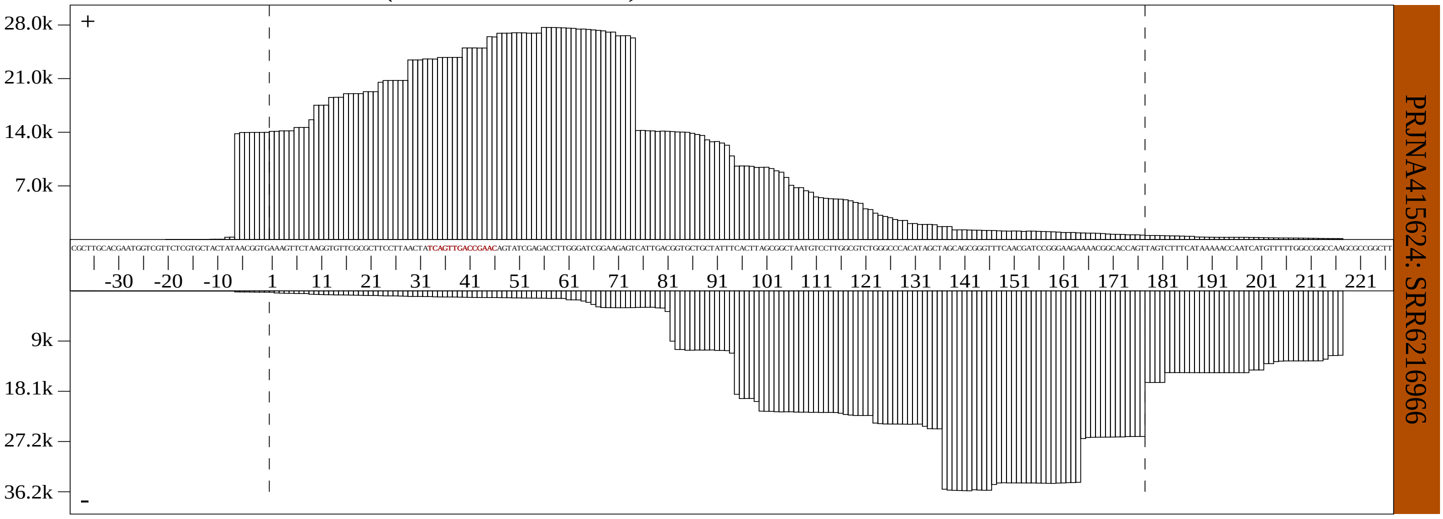


Lib size: 29,010k proposed pRNA: GUUCGGUCAA ...

Note: Looks like strand-independed sequencing

*Lactobacillus rhamnosus (GCF_001988935.1)* NZ_CP014201.1: 371,828 .. 371,542

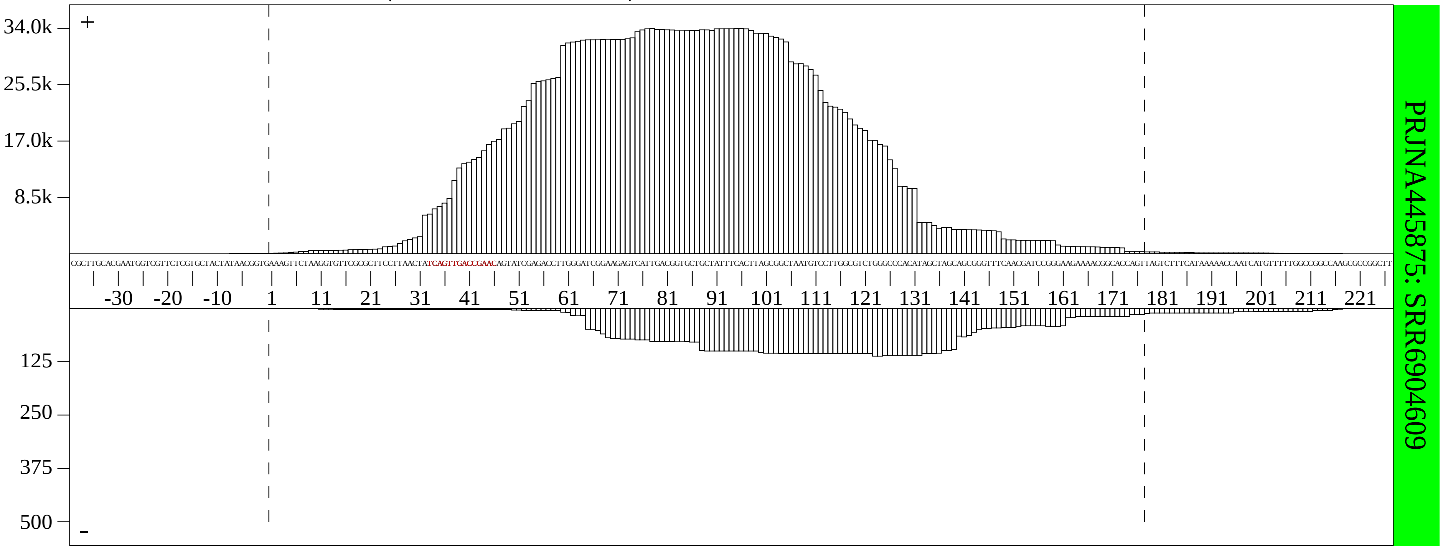


Lib size: 33,498k proposed pRNA: GUUCGGUCAA ...

*Lactobacillus rhamnosus (GCF_001988935.1)* NZ_CP014201.1: 371,828 .. 371,542

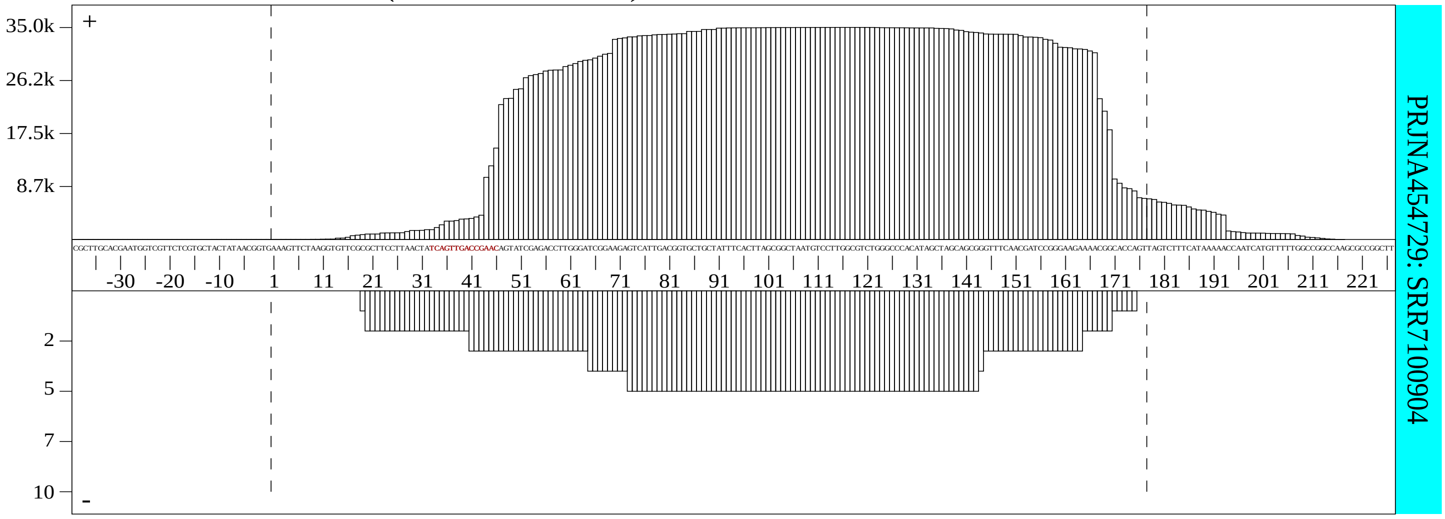


Lib size: 5,055k proposed pRNA: GUUCGGUCAA ...

Note: All reads reverse complemented

# Lactobacillaceae

## *Lactobacillus rhamnosus (6)*

*Lactobacillus rhamnosus (GCF_001988935.1)* NZ_CP014201.1: 371,828 .. 371,542

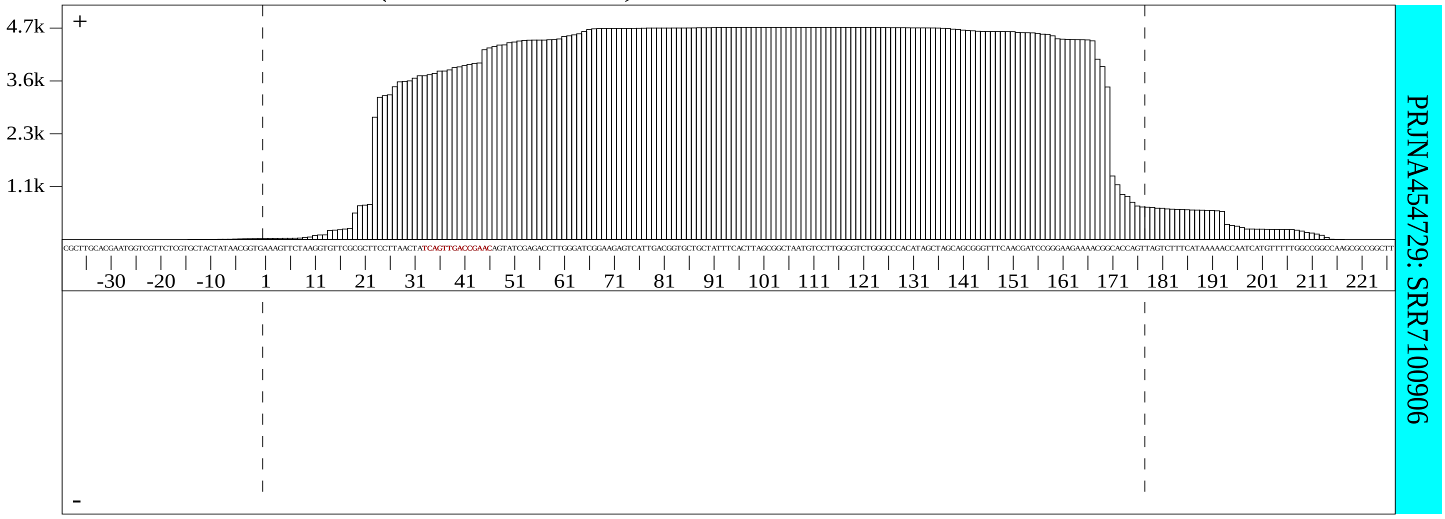


Lib size: 4,039k proposed pRNA: GUUCGGUCAA ...

Note: All reads reverse complemented No reads on - strand

*Lactobacillus rhamnosus (GCF_001988935.1)* NZ_CP014201.1: 371,828 .. 371,542

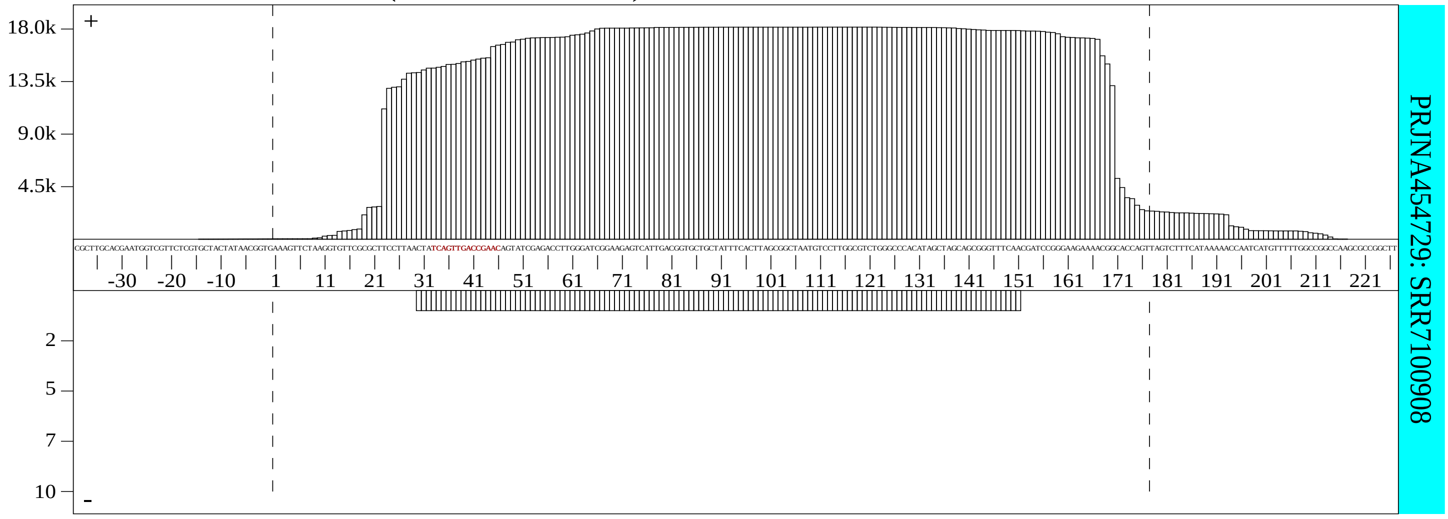


Lib size: 4,043k proposed pRNA: GUUCGGUCAA ...

Note: All reads reverse complemented

*Lactobacillus rhamnosus (GCF_001988935.1)* NZ_CP014201.1: 371,828 .. 371,542

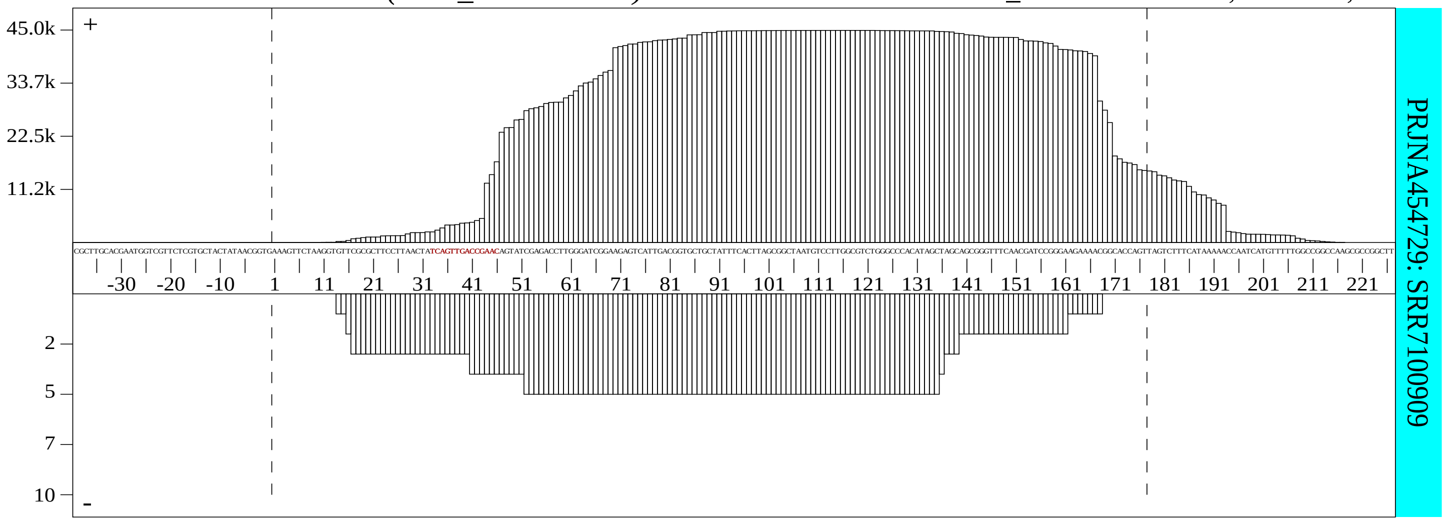


Lib size: 5,119k proposed pRNA: GUUCGGUCAA ...

Note: All reads reverse complemented

# Lactobacillaceae

## *Lactobacillus sakei (5)*

*Lactobacillus sakei (GCF_002224565.1)* NZ_CP020459.1: 815,439 .. 815,151

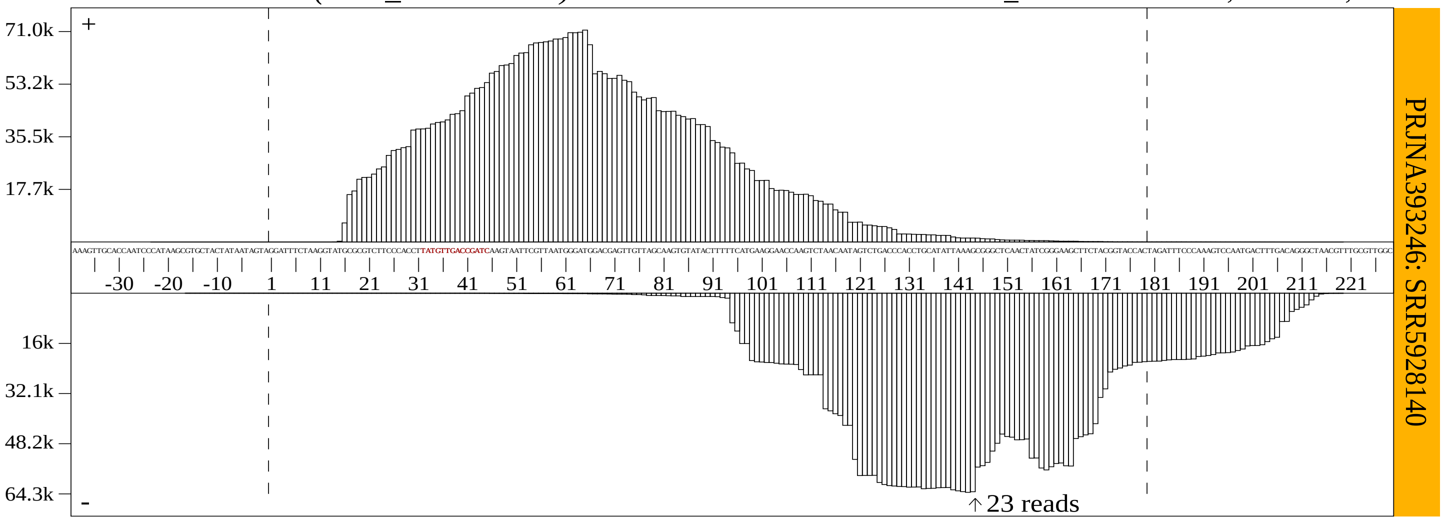


Lib size: 15,991k

proposed pRNA: GAUCGGUCAA ...

Note: Looks like strand-independed sequencing

*Lactobacillus sakei (GCF_002224565.1)* NZ_CP020459.1: 815,439 .. 815,151

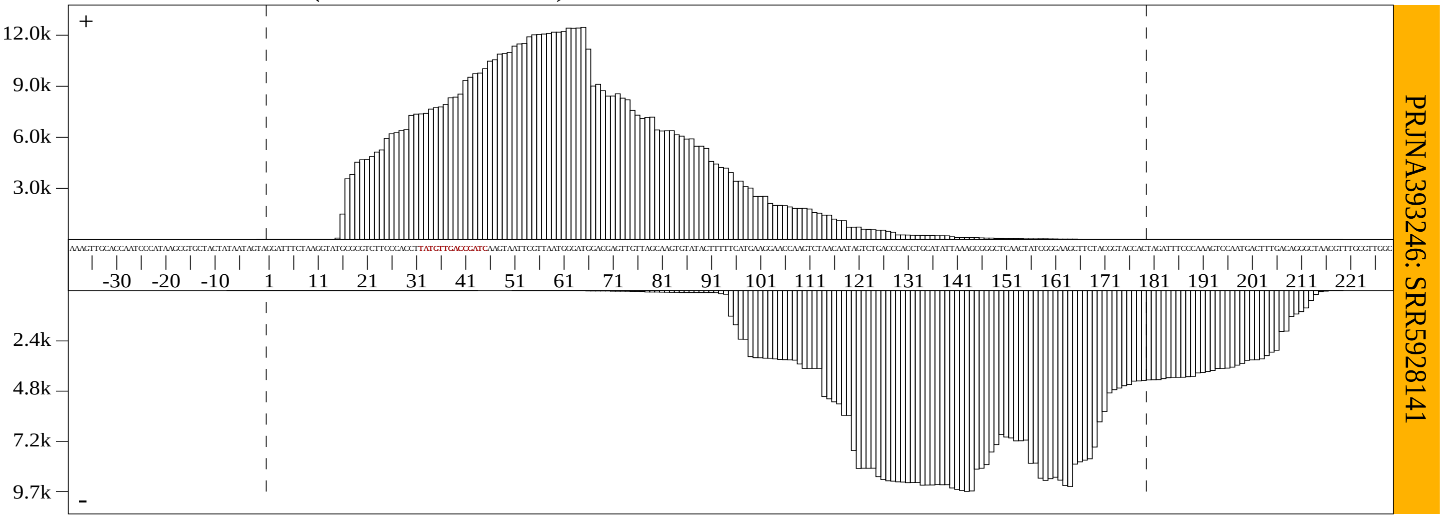


Lib size: 5,761k

proposed pRNA: GAUCGGUCAA ...

Note: Looks like strand-independed sequencing

*Lactobacillus sakei (GCF_002224565.1)* NZ_CP020459.1: 815,439 .. 815,151

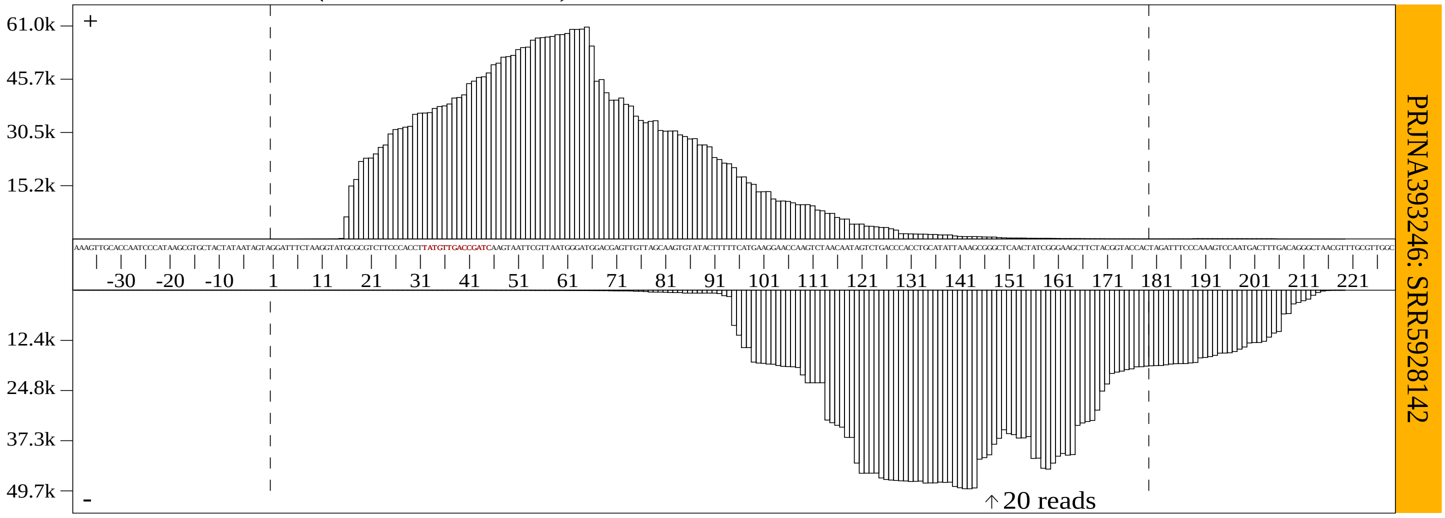


Lib size: 15,631k

proposed pRNA: GAUCGGUCAA ...

Note: Looks like strand-independed sequencing

# Lactobacillaceae

## *Lactobacillus sakei (5)*

*Lactobacillus sakei (GCF_002224565.1)* NZ_CP020459.1: 815,439 .. 815,151

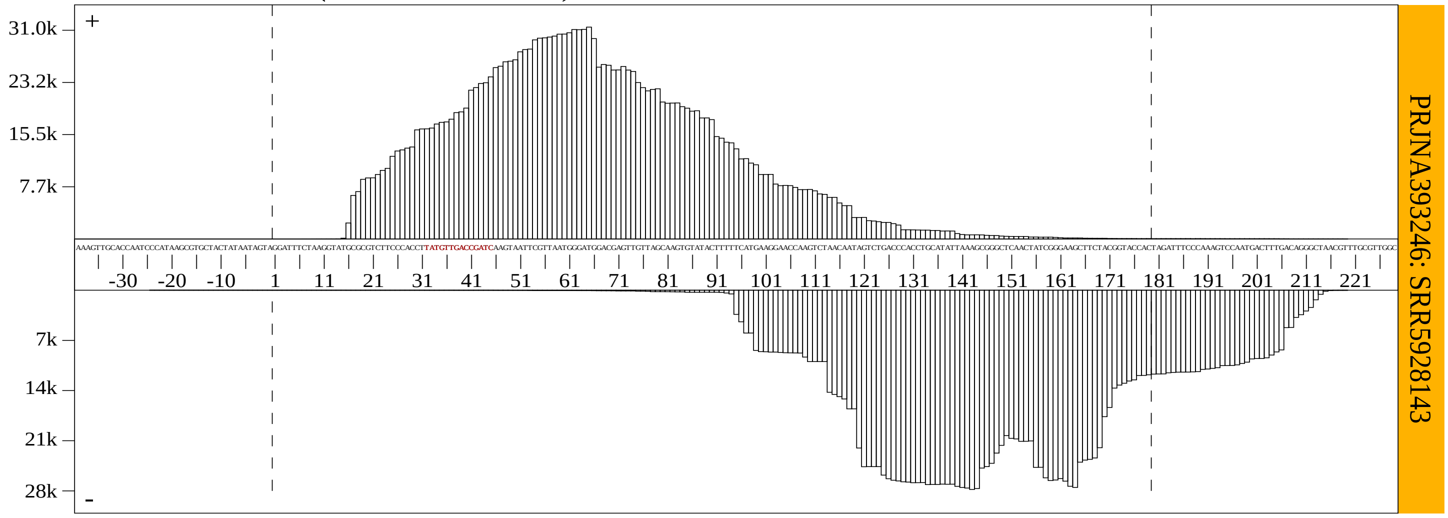


Lib size: 17,062k proposed pRNA: GAUCGGUCAA ...

Note: Looks like strand-independed sequencing

*Lactobacillus sakei (GCF_002224565.1)* NZ_CP020459.1: 815,439 .. 815,151

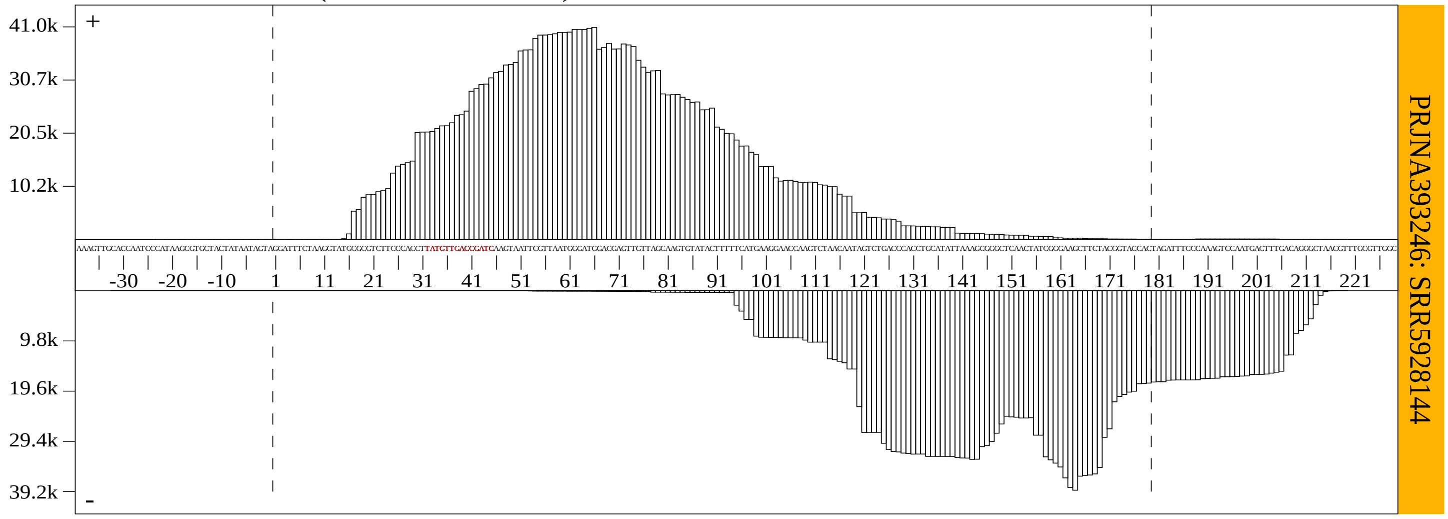


Lib size: 30,822k proposed pRNA: GAUCGGUCAA ...

Note: Looks like strand-independed sequencing

# Lactobacillaceae

## *Lactobacillus salivarius UCC118 (3)*

*Lactobacillus salivarius (GCF_000008925.1)* NC_007929.1: 1,104,478 .. 1,104,762

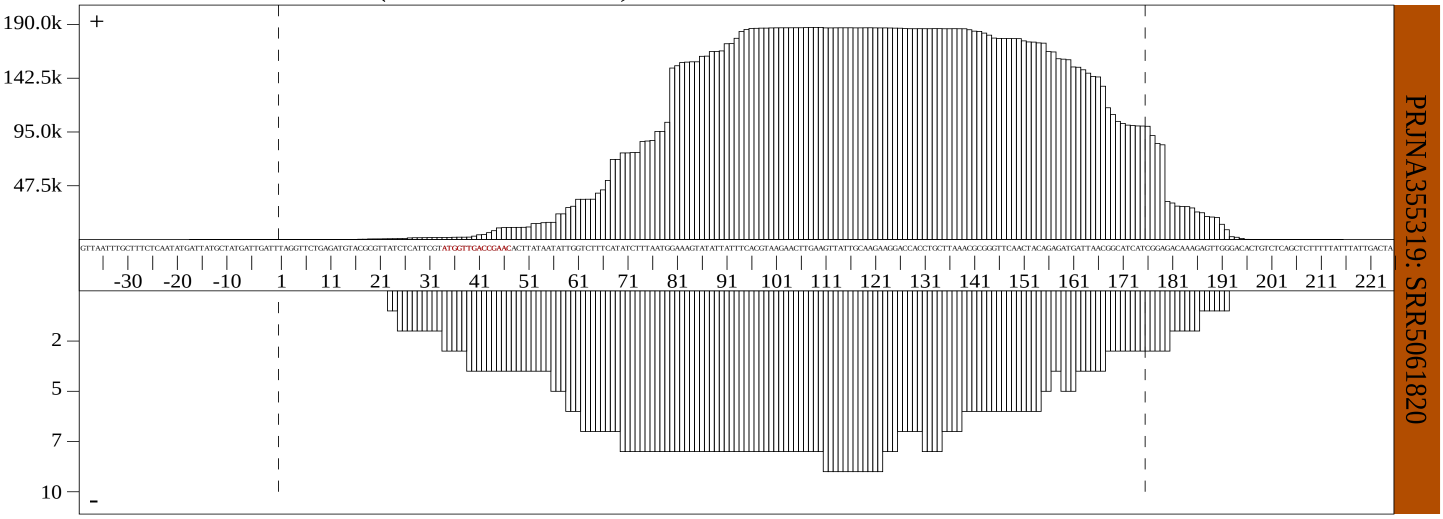


Lib size: 12,917k

proposed pRNA: GUUCGGUCAA ...

Note: All reads reverse complemented

*Lactobacillus salivarius (GCF_000008925.1)* NC_007929.1: 1,104,478 .. 1,104,762

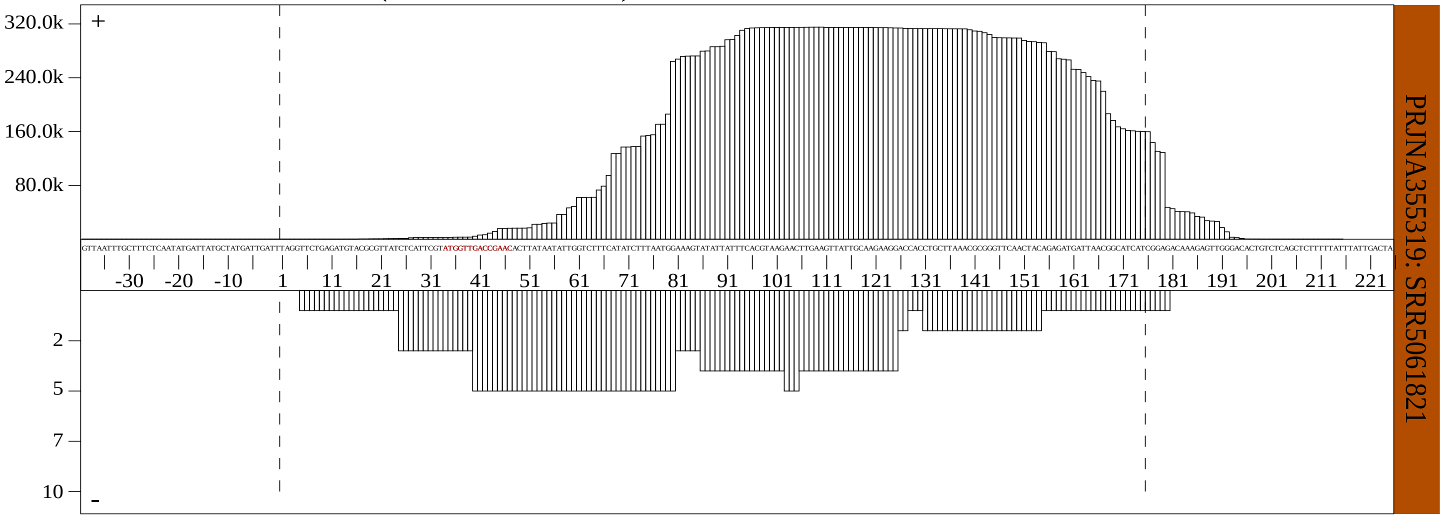


Lib size: 16,466k

proposed pRNA: GUUCGGUCAA ...

Note: All reads reverse complemented

*Lactobacillus salivarius (GCF_000008925.1)* NC_007929.1: 1,104,478 .. 1,104,762

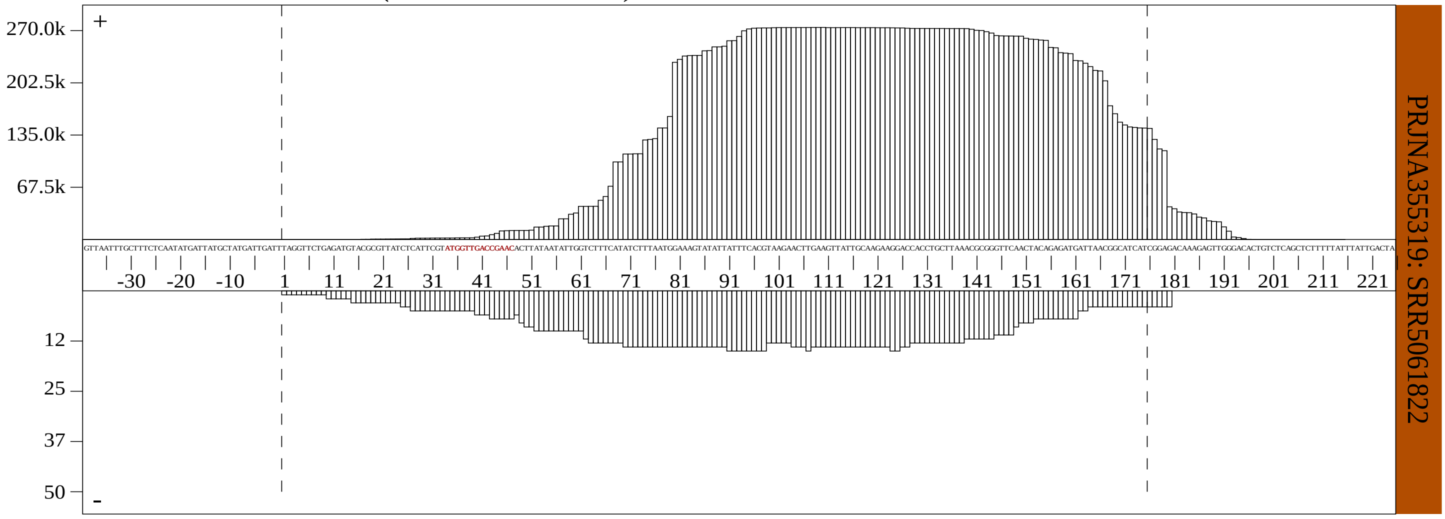


Lib size: 15,049k

proposed pRNA: GUUCGGUCAA ...

Note: All reads reverse complemented

# Lactobacillaceae

## *Lactococcus lactis (18)*

*Lactococcus lactis (GCF_000761115.1)* NZ_CP009472.1: 34,827 .. 35,119

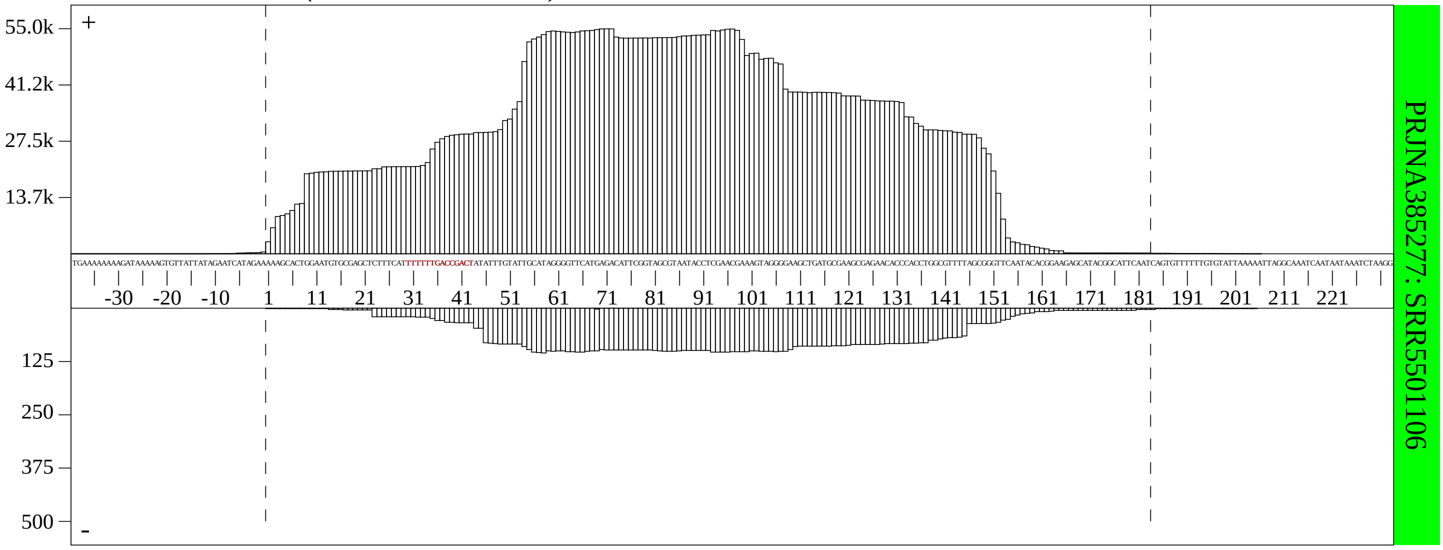


Lib size: 7,845k proposed pRNA: AGUCGGUCAA ...

*Lactococcus lactis (GCF_000761115.1)* NZ_CP009472.1: 34,827 .. 35,119

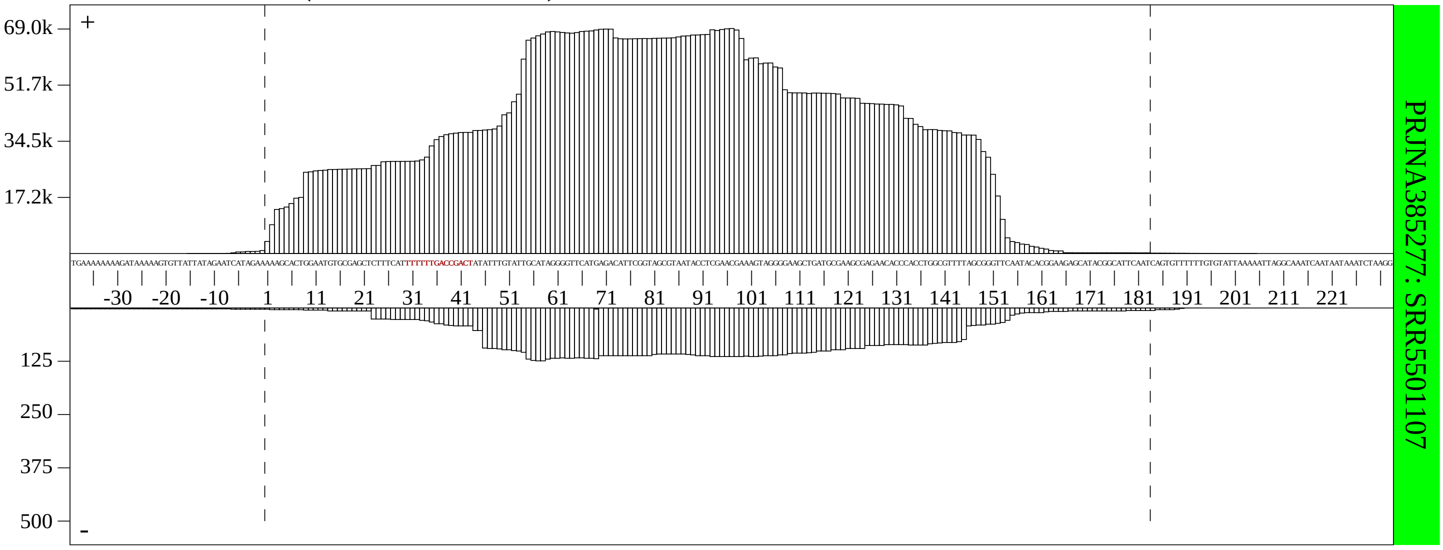


Lib size: 10,087k proposed pRNA: AGUCGGUCAA ...

*Lactococcus lactis (GCF_000761115.1)* NZ_CP009472.1: 34,827 .. 35,119

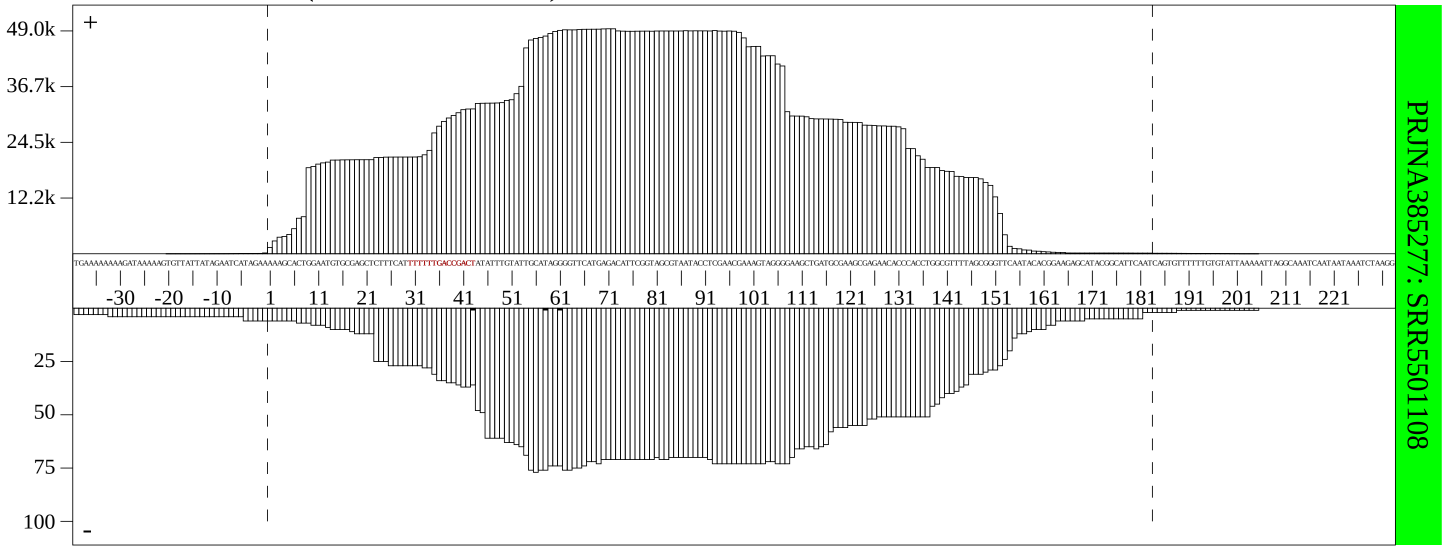


Lib size: 4,404k proposed pRNA: AGUCGGUCAA ...

# Lactobacillaceae

## *Lactococcus lactis (18)*

*Lactococcus lactis (GCF_000761115.1)* NZ_CP009472.1: 34,827 .. 35,119

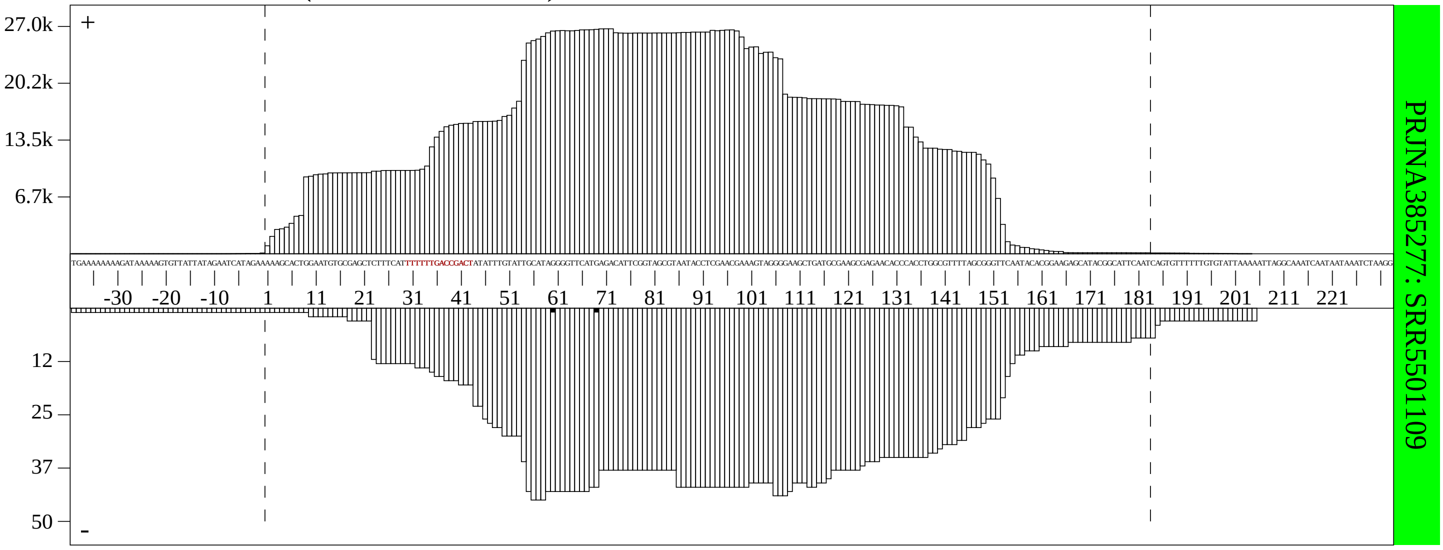


Lib size: 6,022k proposed pRNA: AGUCGGUCAA ...

*Lactococcus lactis (GCF_000761115.1)* NZ_CP009472.1: 34,827 .. 35,119

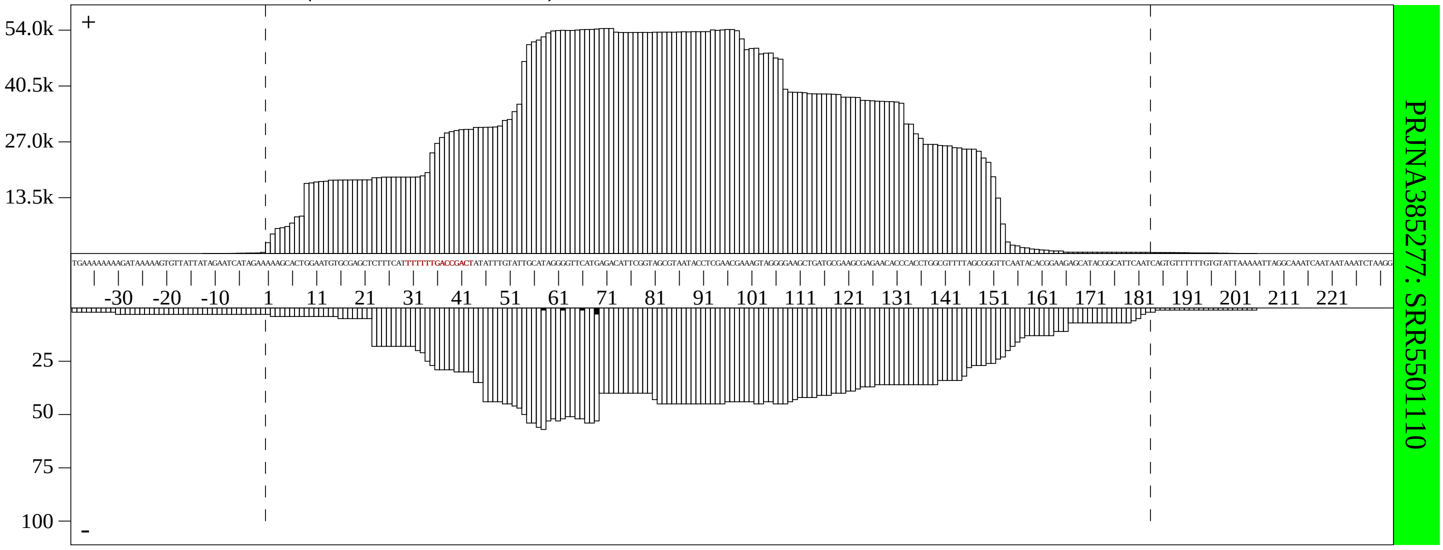


Lib size: 8,484k proposed pRNA: AGUCGGUCAA ...

*Lactococcus lactis (GCF_000761115.1)* NZ_CP009472.1: 34,827 .. 35,119

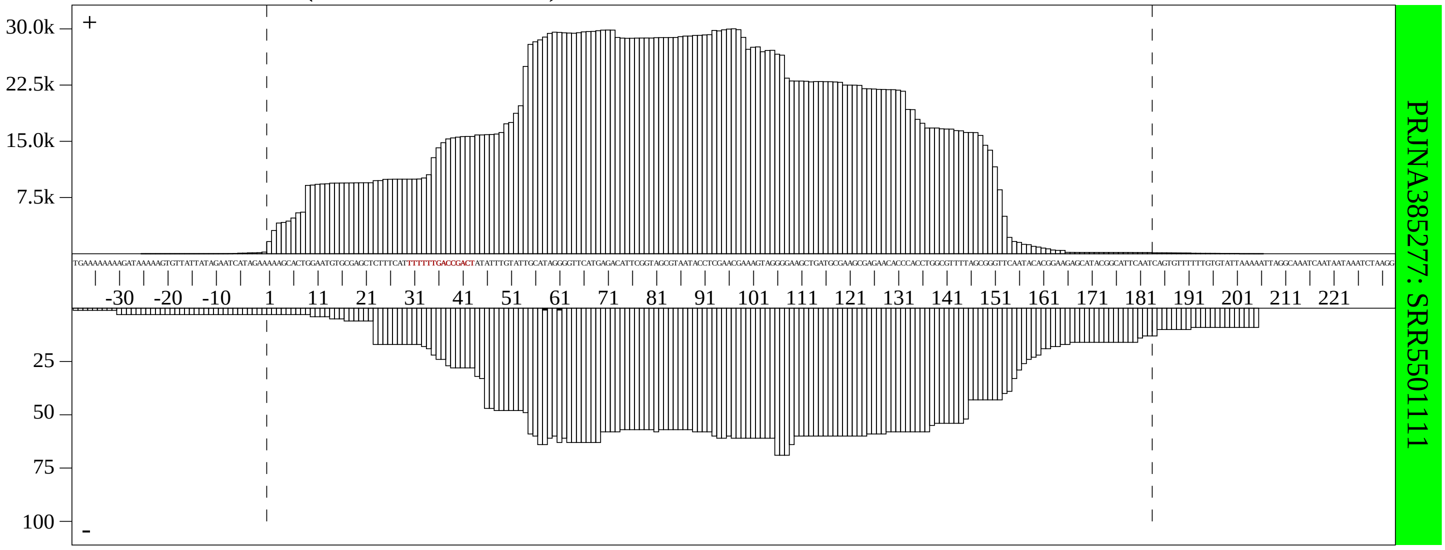


Lib size: 7,258k proposed pRNA: AGUCGGUCAA ...

# Lactobacillaceae

## *Lactococcus lactis (18)*

*Lactococcus lactis (GCF_000761115.1)* NZ_CP009472.1: 34,827 .. 35,119

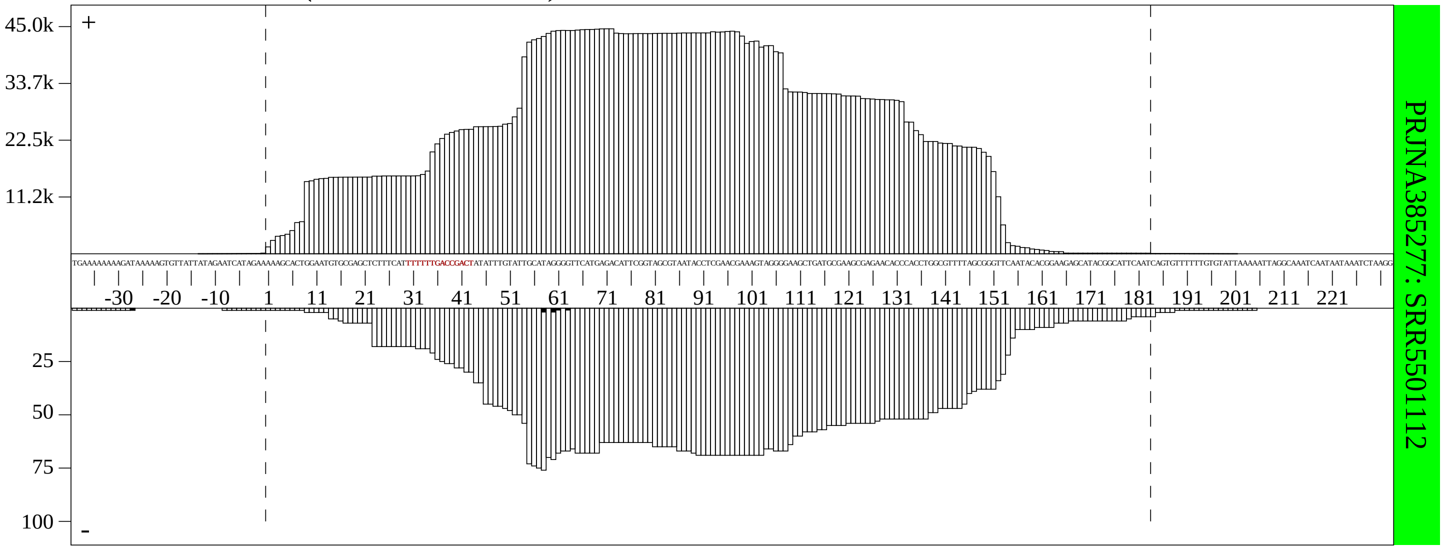


Lib size: 5,455k proposed pRNA: AGUCGGUCAA ...

*Lactococcus lactis (GCF_000761115.1)* NZ_CP009472.1: 34,827 .. 35,119

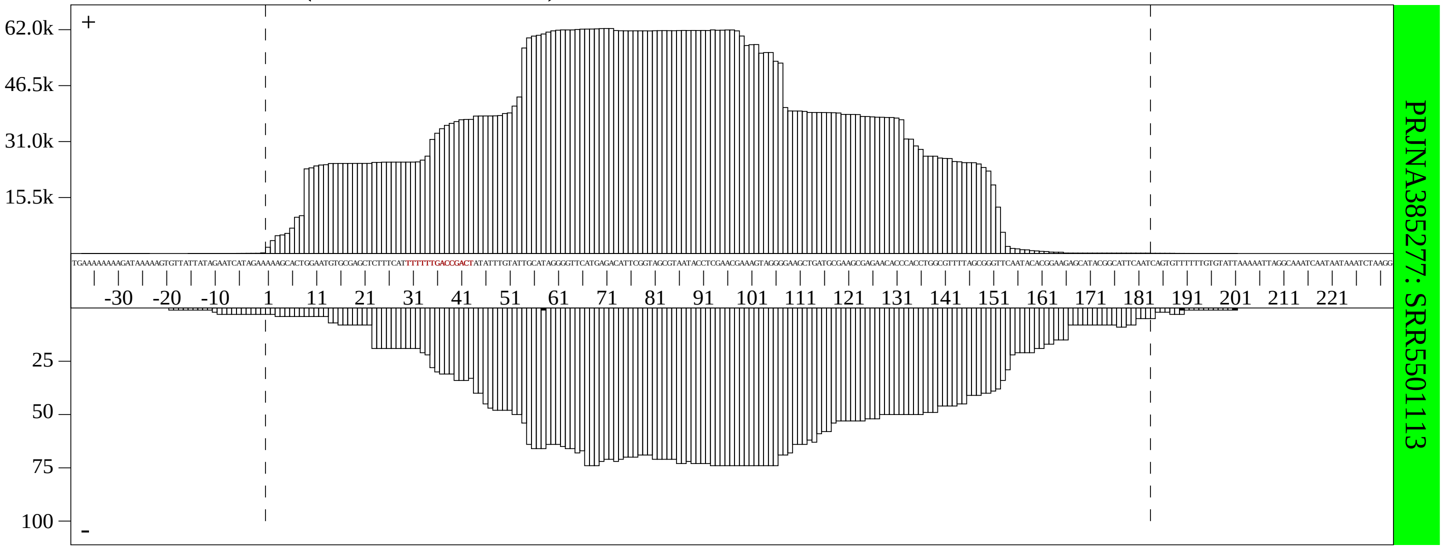


Lib size: 5,696k proposed pRNA: AGUCGGUCAA ...

*Lactococcus lactis (GCF_000761115.1)* NZ_CP009472.1: 34,827 .. 35,119

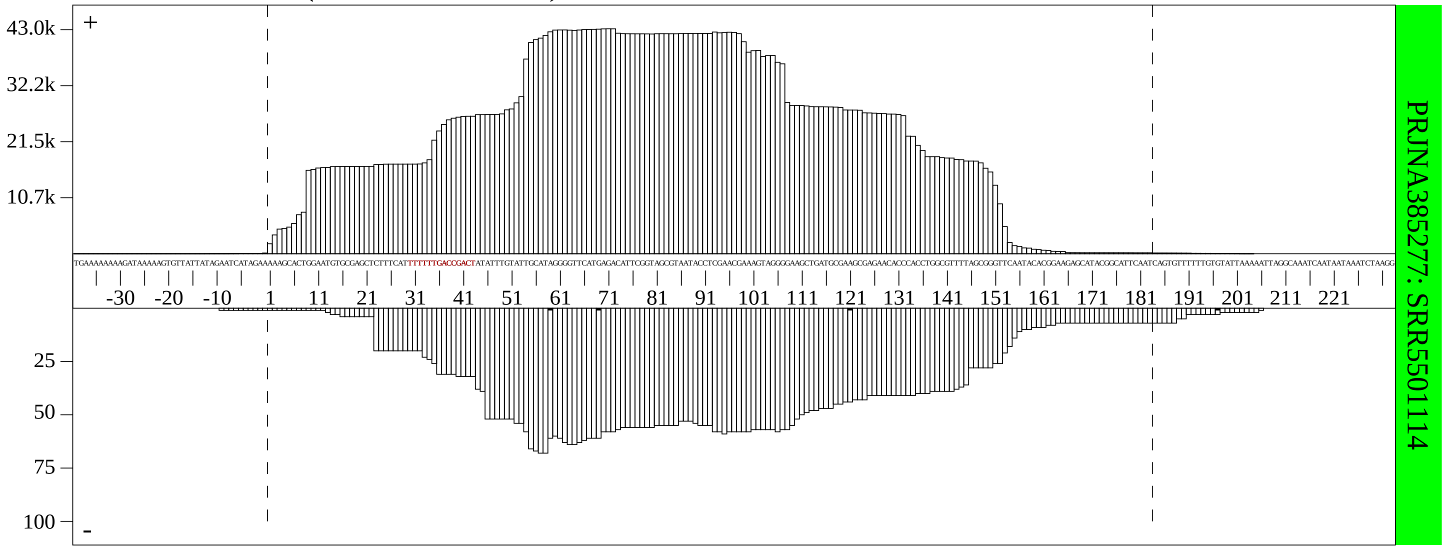


Lib size: 6,941k proposed pRNA: AGUCGGUCAA ...

# Lactobacillaceae

## *Lactococcus lactis (18)*

*Lactococcus lactis (GCF_000761115.1)* NZ_CP009472.1: 34,827 .. 35,119

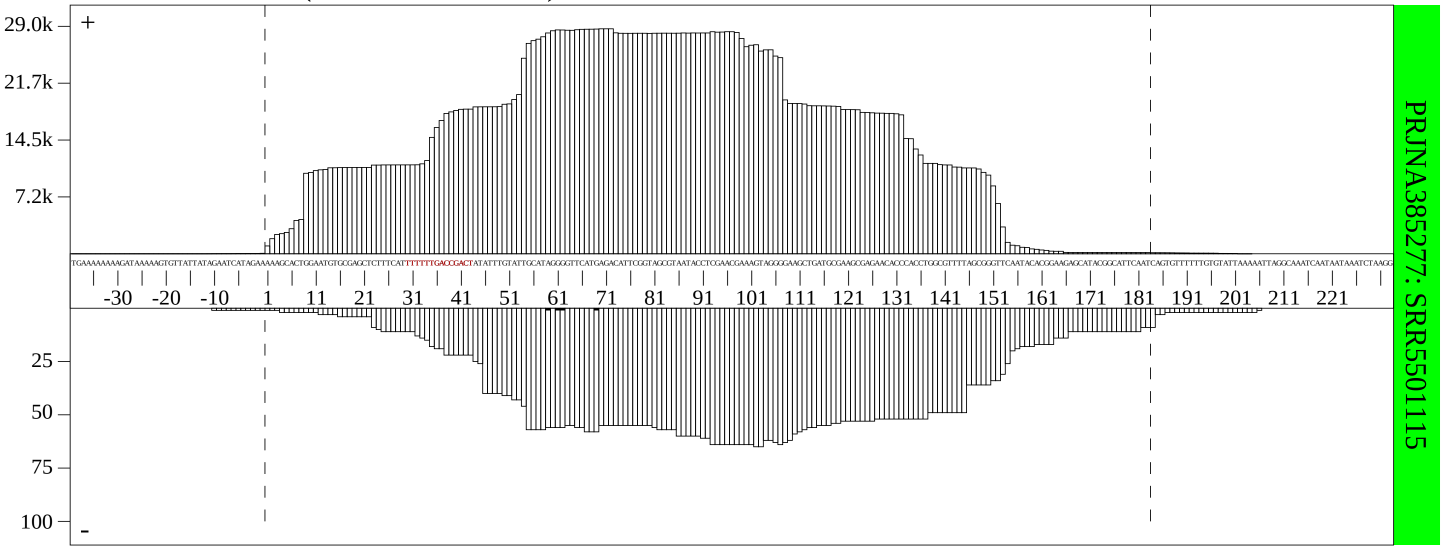


Lib size: 6,886k proposed pRNA: AGUCGGUCAA ...

*Lactococcus lactis (GCF_000761115.1)* NZ_CP009472.1: 34,827 .. 35,119

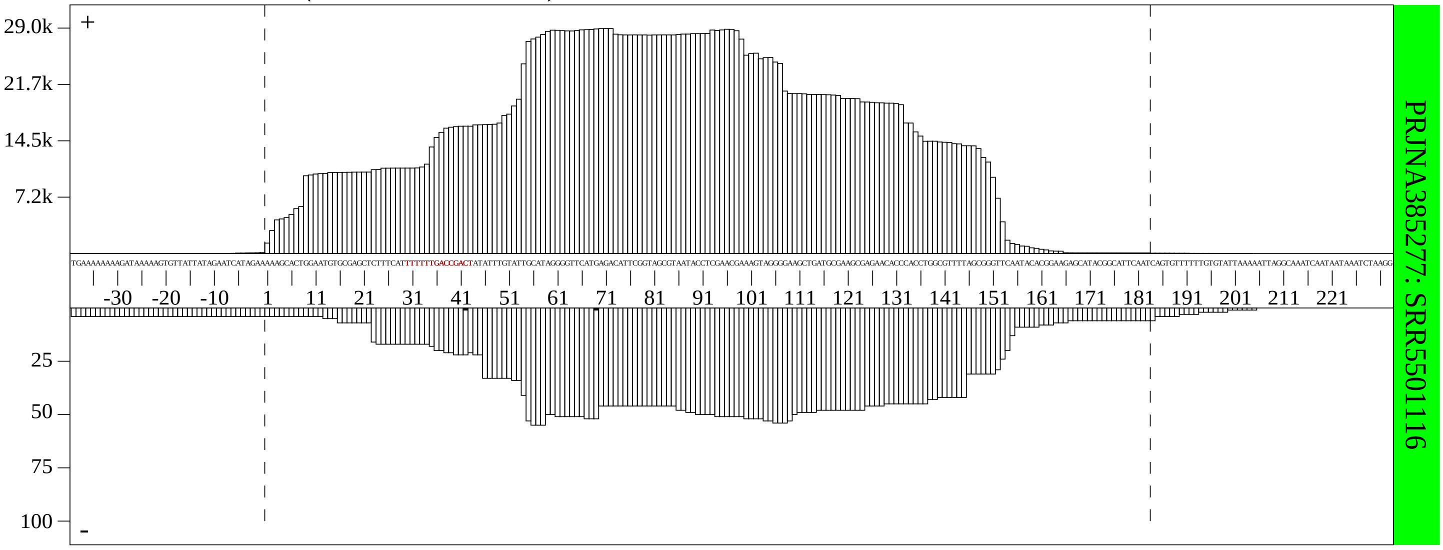


Lib size: 7,773k proposed pRNA: AGUCGGUCAA ...

*Lactococcus lactis (GCF_000761115.1)* NZ_CP009472.1: 34,827 .. 35,119

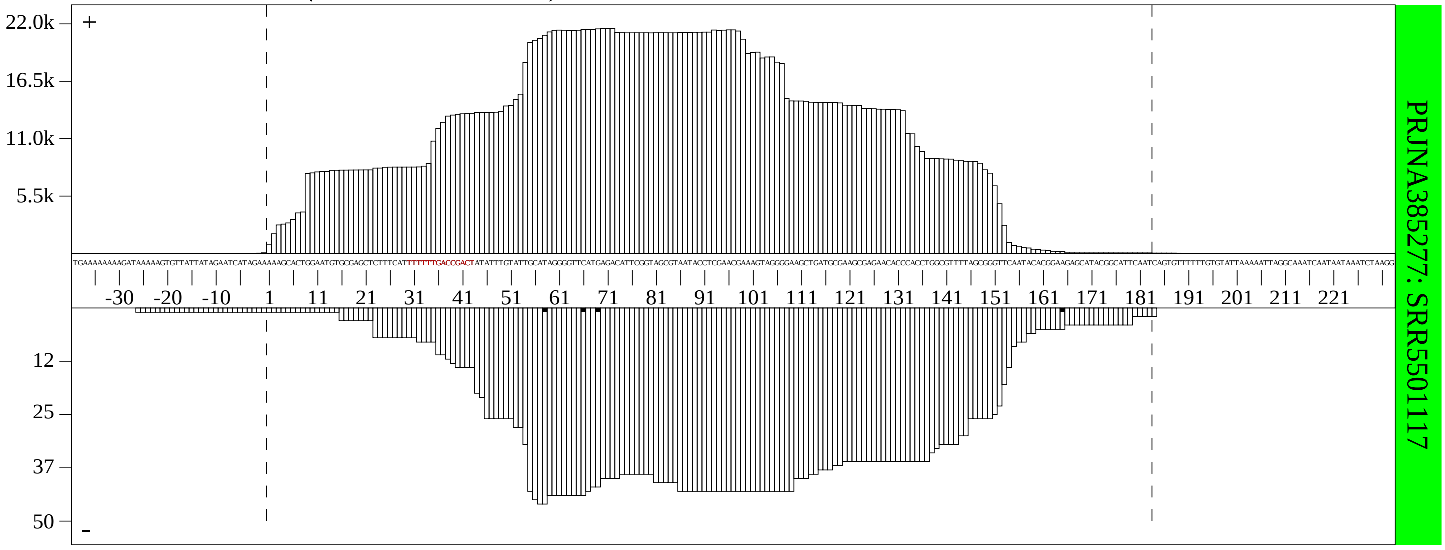


Lib size: 5,406k proposed pRNA: AGUCGGUCAA ...

# Lactobacillaceae

## *Lactococcus lactis (18)*

*Lactococcus lactis (GCF_000761115.1)* NZ_CP009472.1: 34,827 .. 35,119

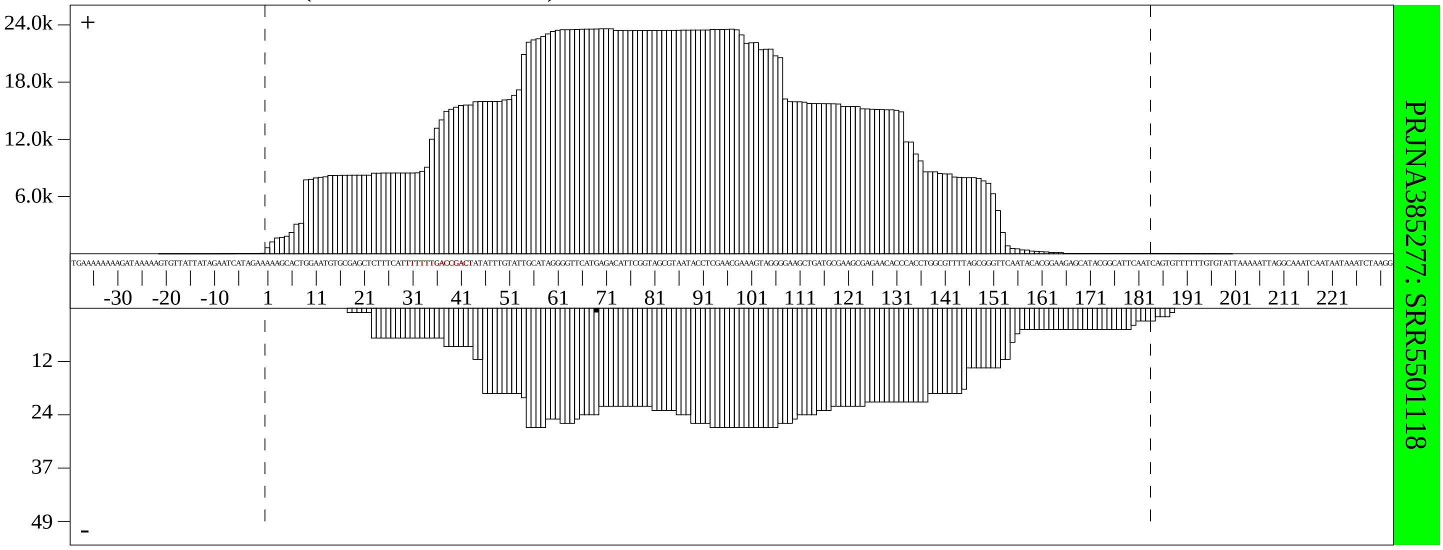


Lib size: 2,794k proposed pRNA: AGUCGGUCAA ...

*Lactococcus lactis (GCF_000761115.1)* NZ_CP009472.1: 34,827 .. 35,119

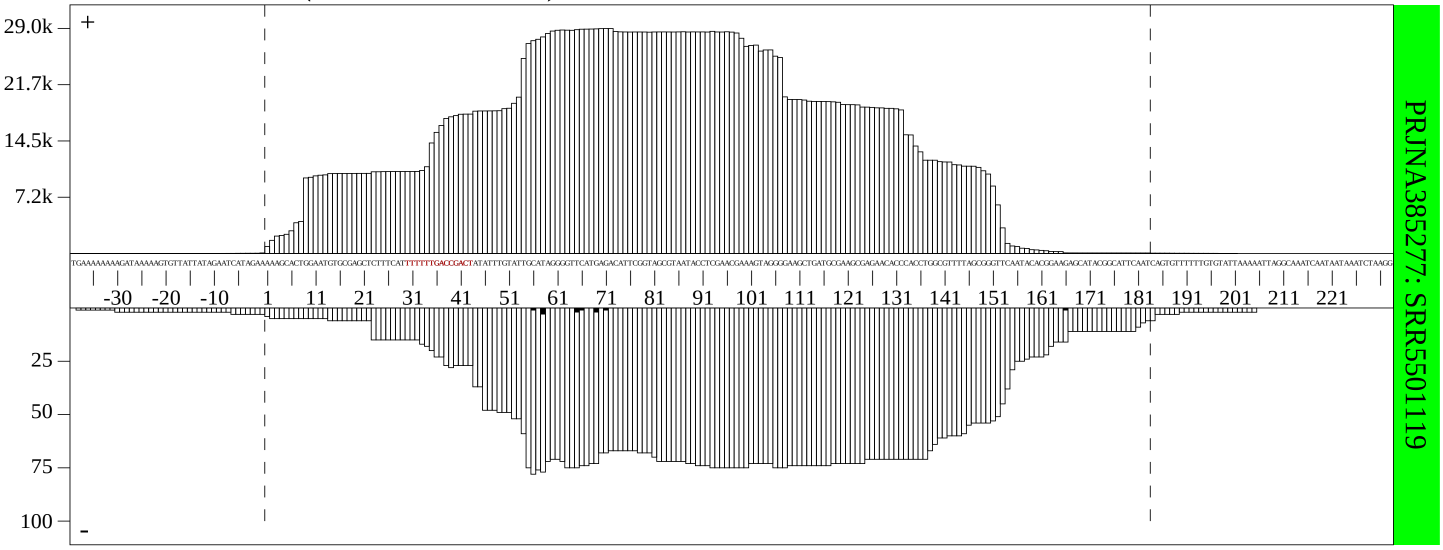


Lib size: 4,823k proposed pRNA: AGUCGGUCAA ...

*Lactococcus lactis (GCF_000761115.1)* NZ_CP009472.1: 34,827 .. 35,119

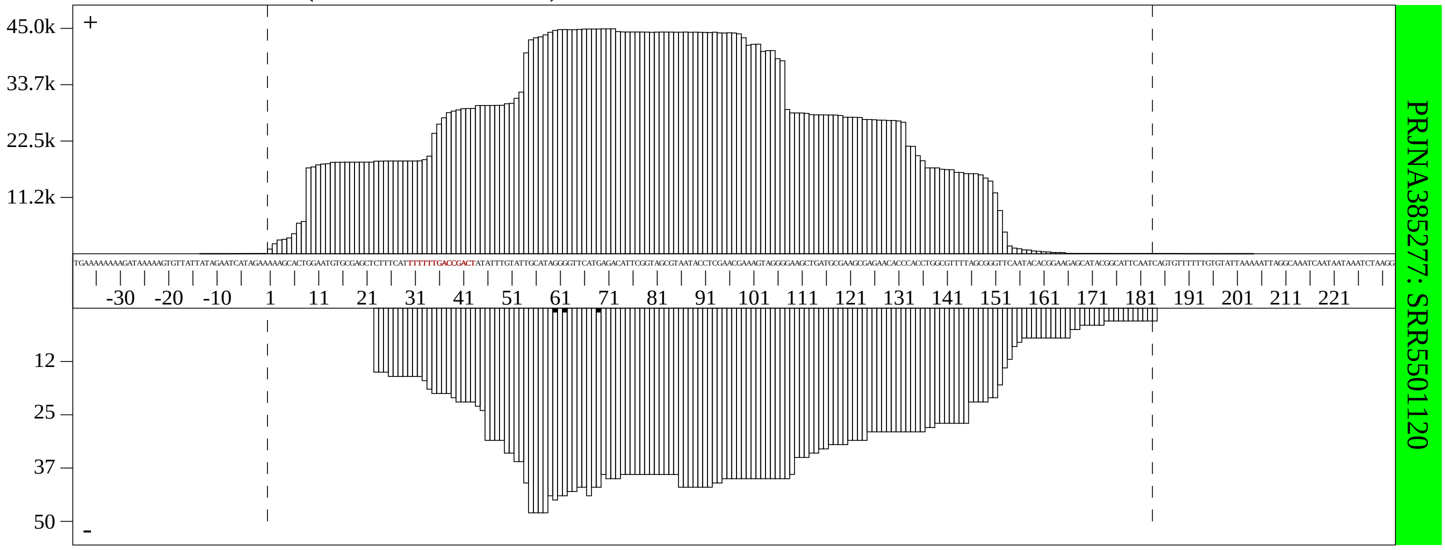


Lib size: 4,563k proposed pRNA: AGUCGGUCAA ...

# Lactobacillaceae

## *Lactococcus lactis (18)*

*Lactococcus lactis (GCF_000761115.1)* NZ_CP009472.1: 34,827 .. 35,119

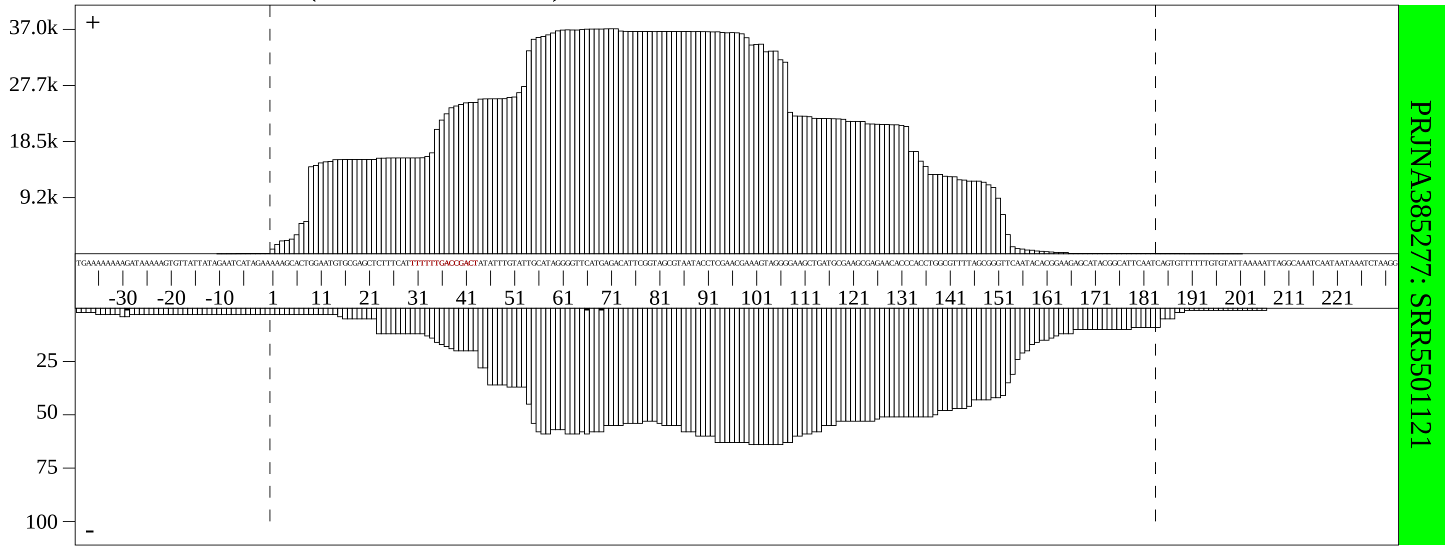


Lib size: 4,031k proposed pRNA: AGUCGGUCAA ...

*Lactococcus lactis (GCF_000761115.1)* NZ_CP009472.1: 34,827 .. 35,119

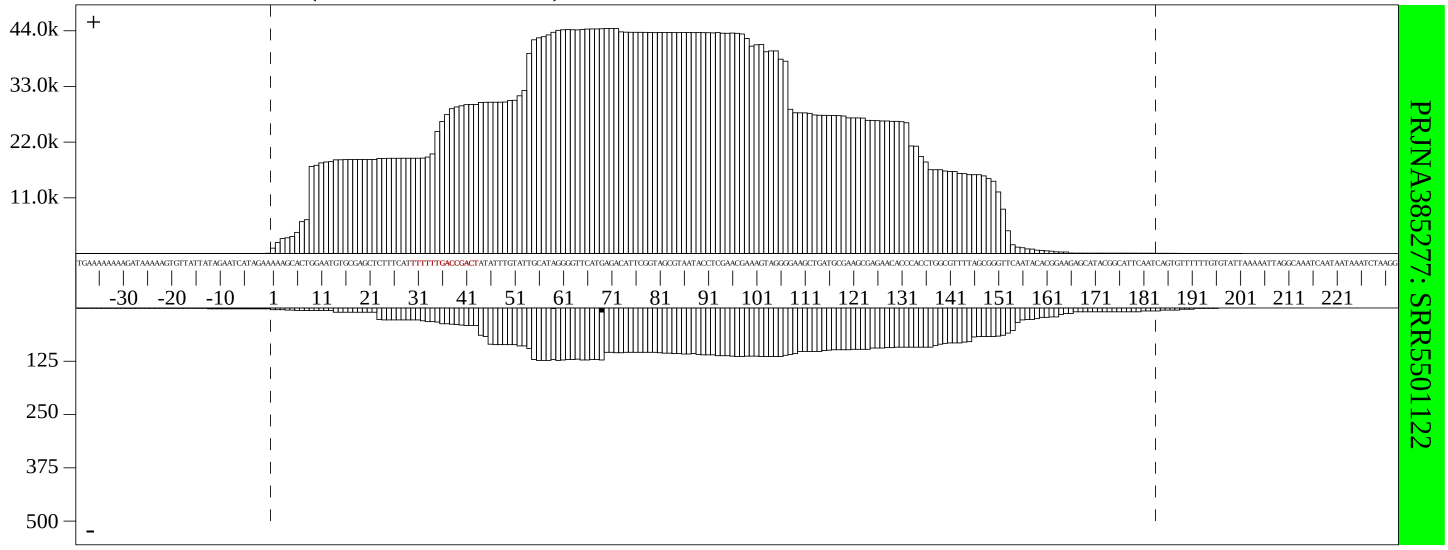


Lib size: 5,744k proposed pRNA: AGUCGGUCAA ...

*Lactococcus lactis (GCF_000761115.1)* NZ_CP009472.1: 34,827 .. 35,119

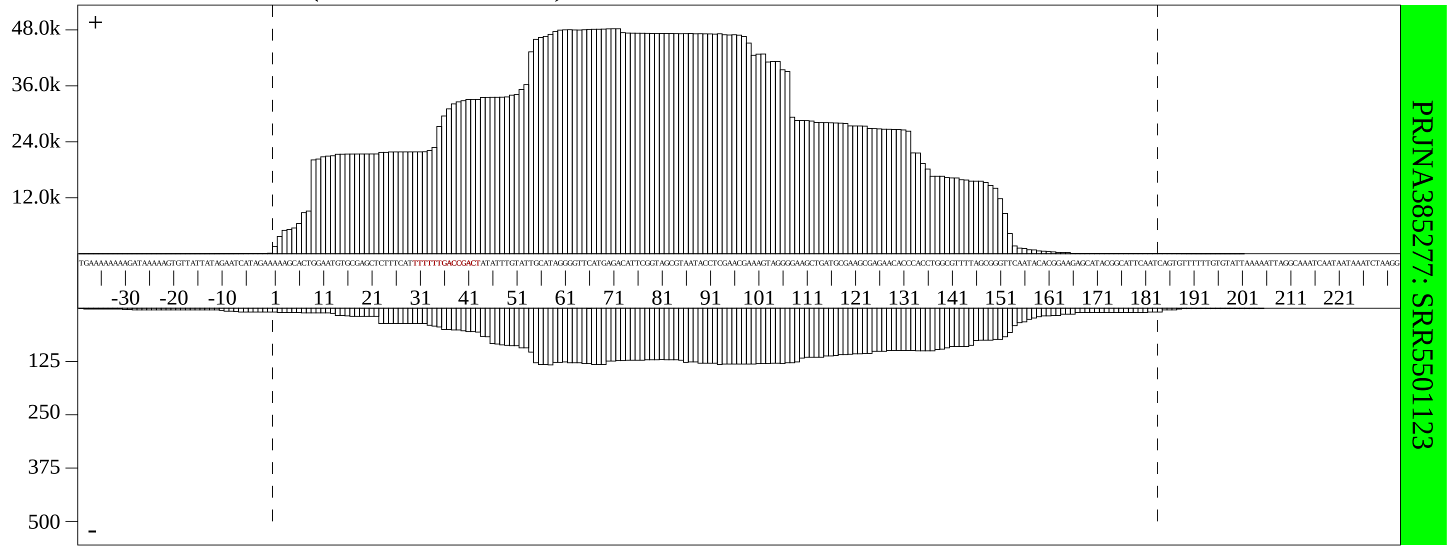


Lib size: 4,616k proposed pRNA: AGUCGGUCAA ...

# Lactobacillaceae

## *Oenococcus oeni (3)*

*Oenococcus oeni (GCF_003264795.1)* NZ_CP027431.1: 1,377,732 .. 1,377,448

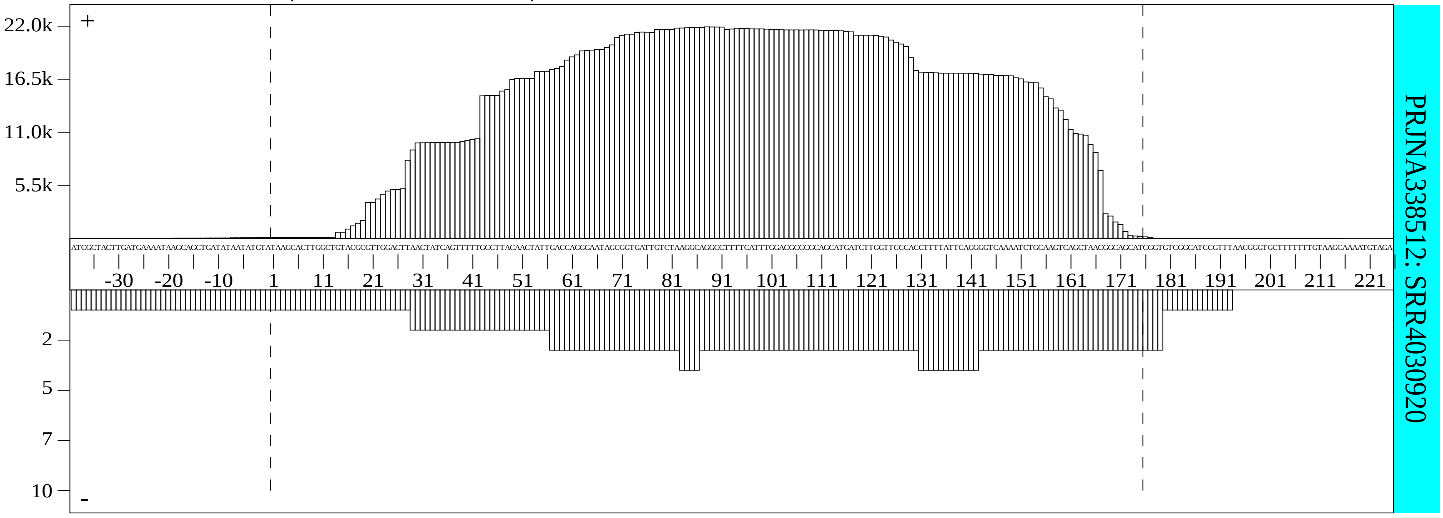


Lib size: 15,554k

Note: All reads reverse complemented

*Oenococcus oeni (GCF_003264795.1)* NZ_CP027431.1: 1,377,732 .. 1,377,448

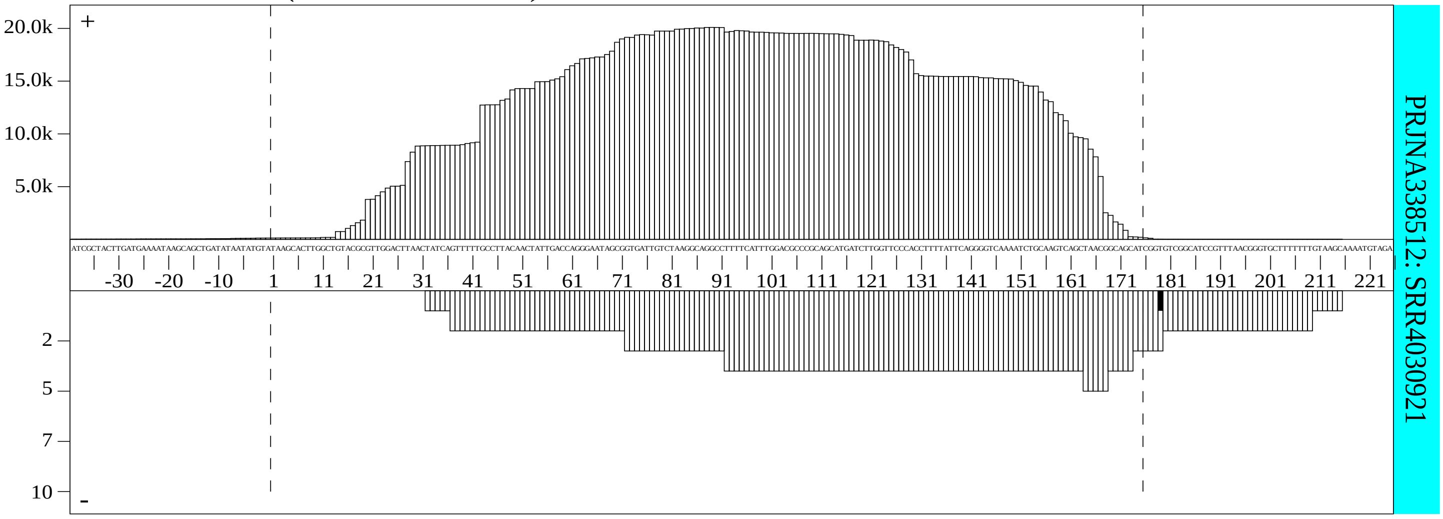


Lib size: 12,137k

Note: All reads reverse complemented

*Oenococcus oeni (GCF_003264795.1)* NZ_CP027431.1: 1,377,732 .. 1,377,448

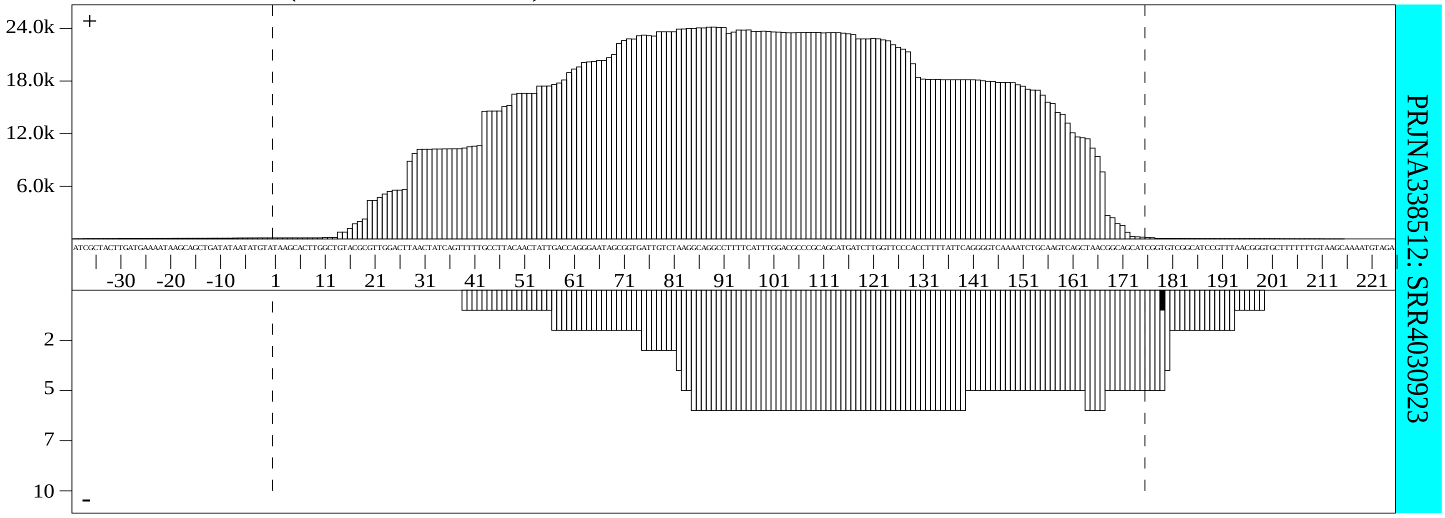


Lib size: 12,623k

Note: All reads reverse complemented

# Lactobacillaceae

## *Pediococcus claussenii ATCC BAA-344 (4)*

*Pediococcus claussenii (GCF_000237995.1)* NC_016605.1: 699,055 .. 698,774

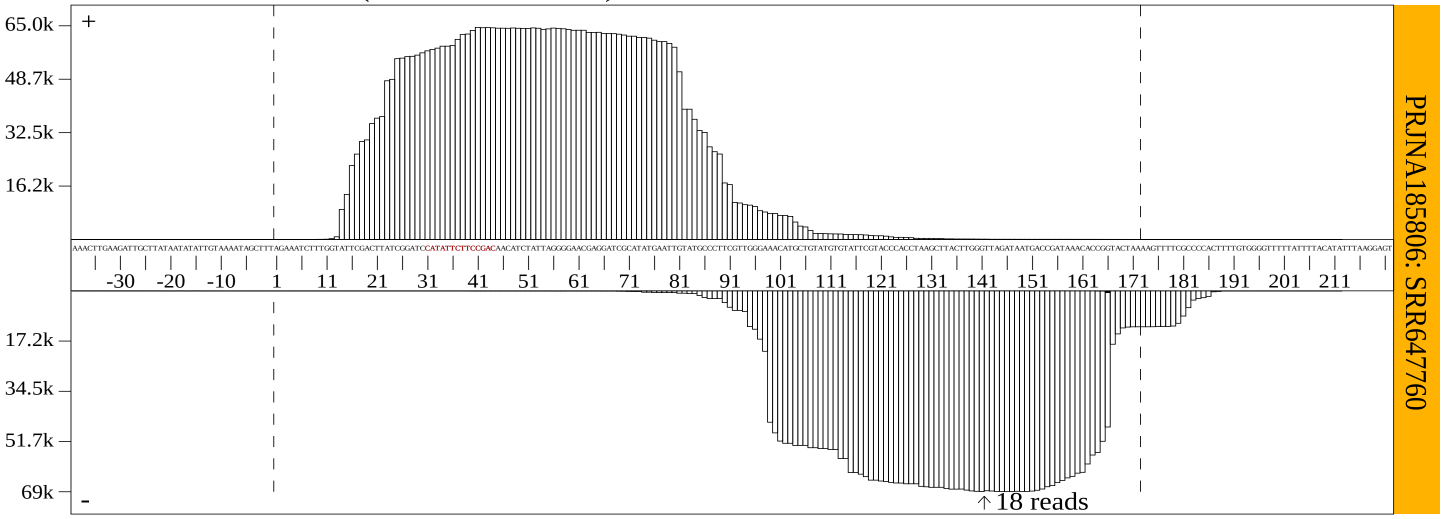


Lib size: 11,426k proposed pRNA: GUCGGAAGAA ...

Note: Looks like strand-independed sequencing

*Pediococcus claussenii (GCF_000237995.1)* NC_016605.1: 699,055 .. 698,774

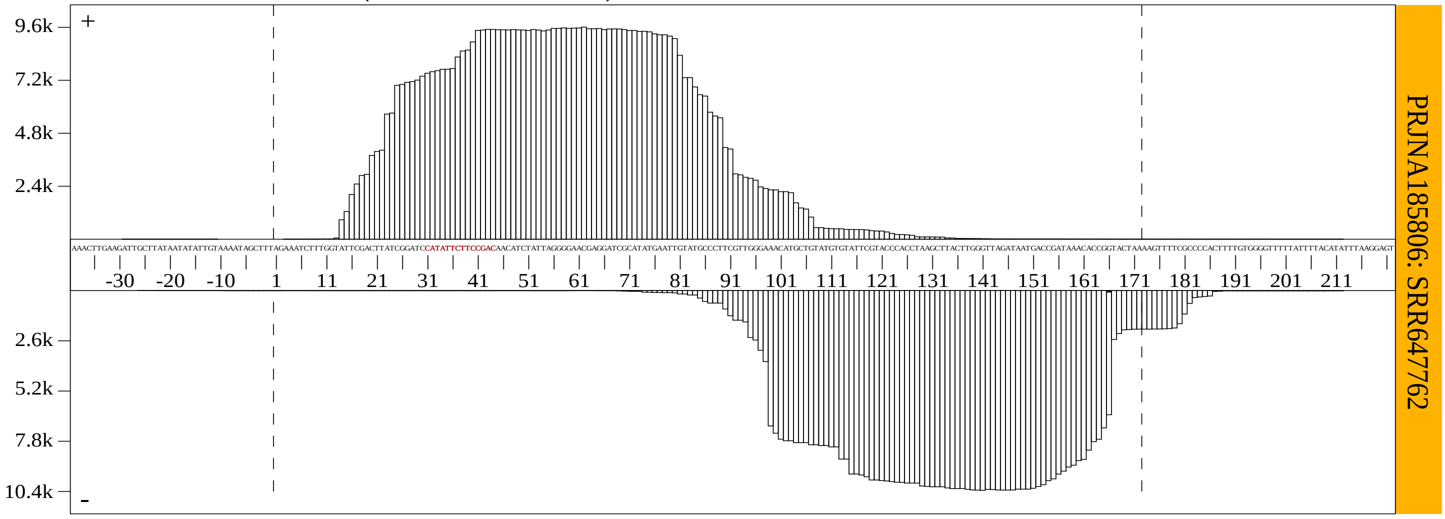


Lib size: 7,616k proposed pRNA: GUCGGAAGAA ...

Note: Looks like strand-independed sequencing

*Pediococcus claussenii (GCF_000237995.1)* NC_016605.1: 699,055 .. 698,774

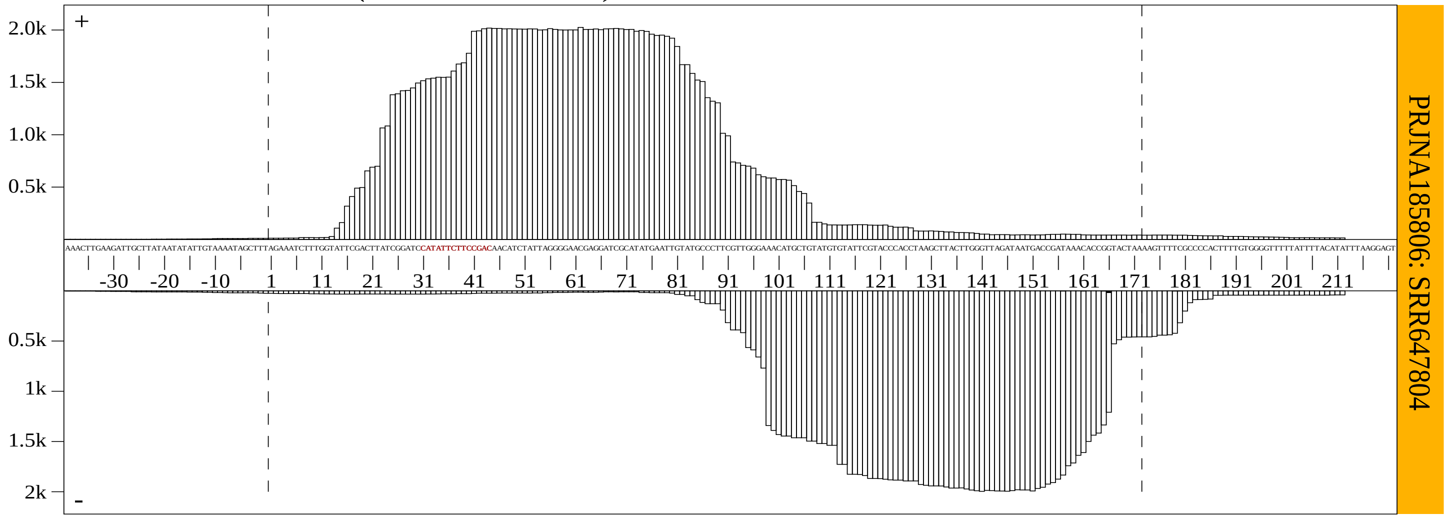


Lib size: 13,110k proposed pRNA: GUCGGAAGAA ...

Note: Looks like strand-independed sequencing

# Lactobacillaceae

## *Pediococcus pentosaceus (1)*

*Pediococcus pentosaceus (GCF_001411765.2)* NZ_CP015918.1: 779,877 .. 779,598

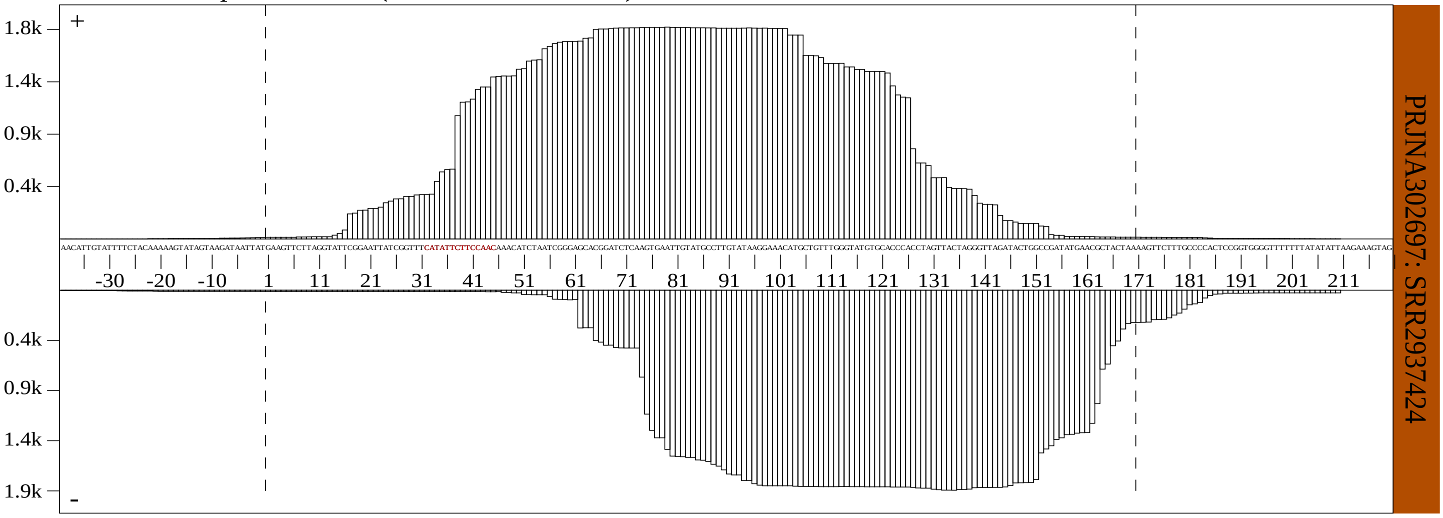


Lib size: 6,383k proposed pRNA: GUUGGAAGAA ...

Note: Looks like strand-independed sequencing

# Streptococcaceae

## *Streptococcus anginosus (4)*

*Streptococcus anginosus (GCF_000831165.1)* NZ_CP007573.1: 1,800,360 .. 1,800,653

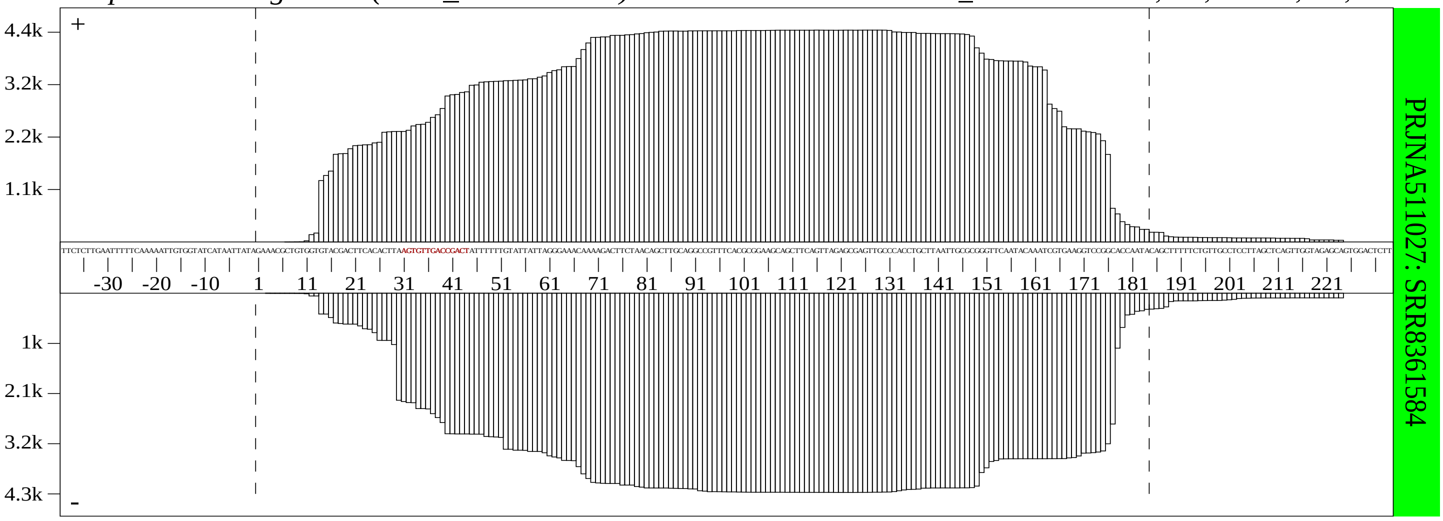


Lib size: 6,563k proposed pRNA: AGUCGGUCAA ...

Note: Looks like strand-independed sequencing

*Streptococcus anginosus (GCF_000831165.1)* NZ_CP007573.1: 1,800,360 .. 1,800,653

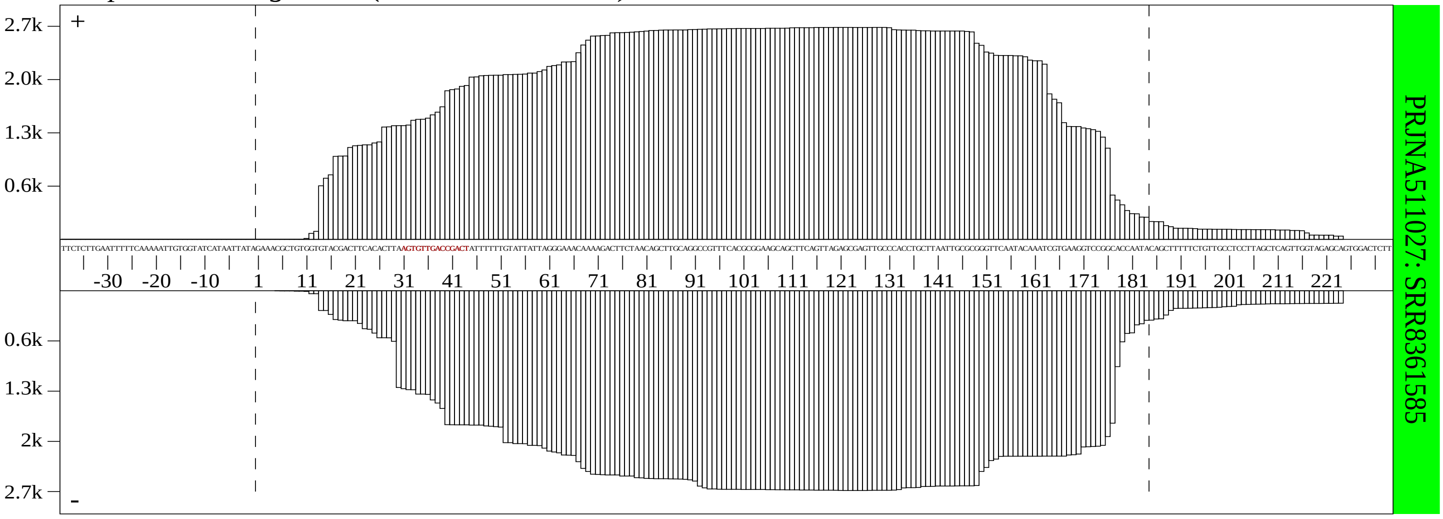


Lib size: 4,544k proposed pRNA: AGUCGGUCAA ...

Note: Looks like strand-independed sequencing

*Streptococcus anginosus (GCF_000831165.1)* NZ_CP007573.1: 1,800,360 .. 1,800,653

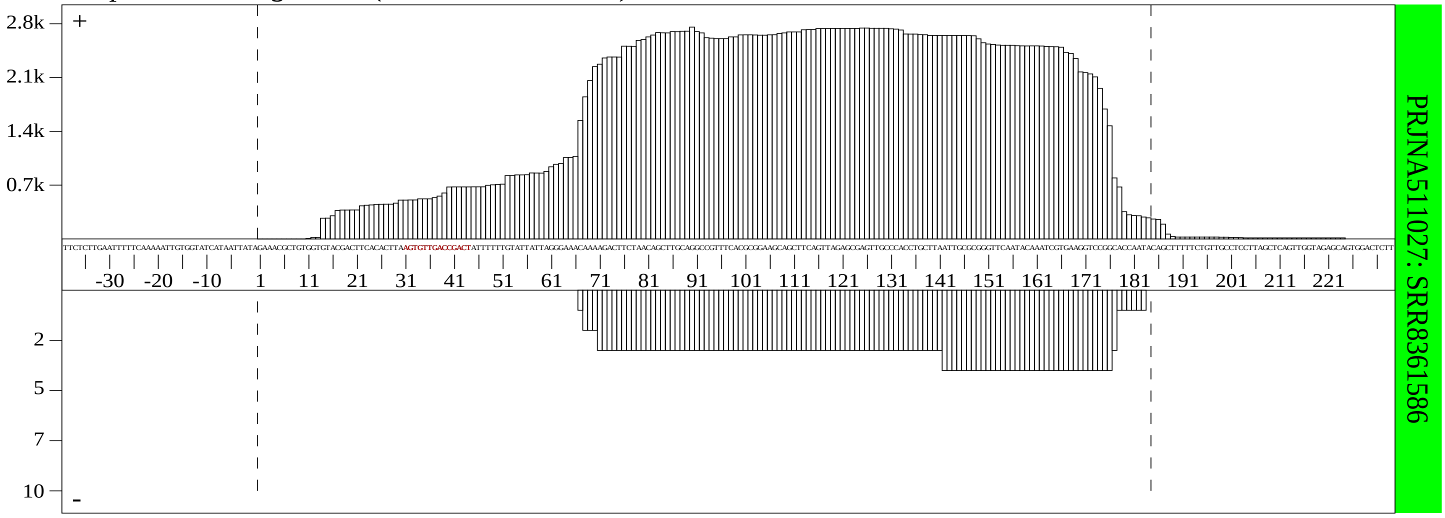


Lib size: 6,117k proposed pRNA: AGUCGGUCAA ...

Note: All reads reverse complemented

# Streptococcaceae

## *Streptococcus anginosus (4)*

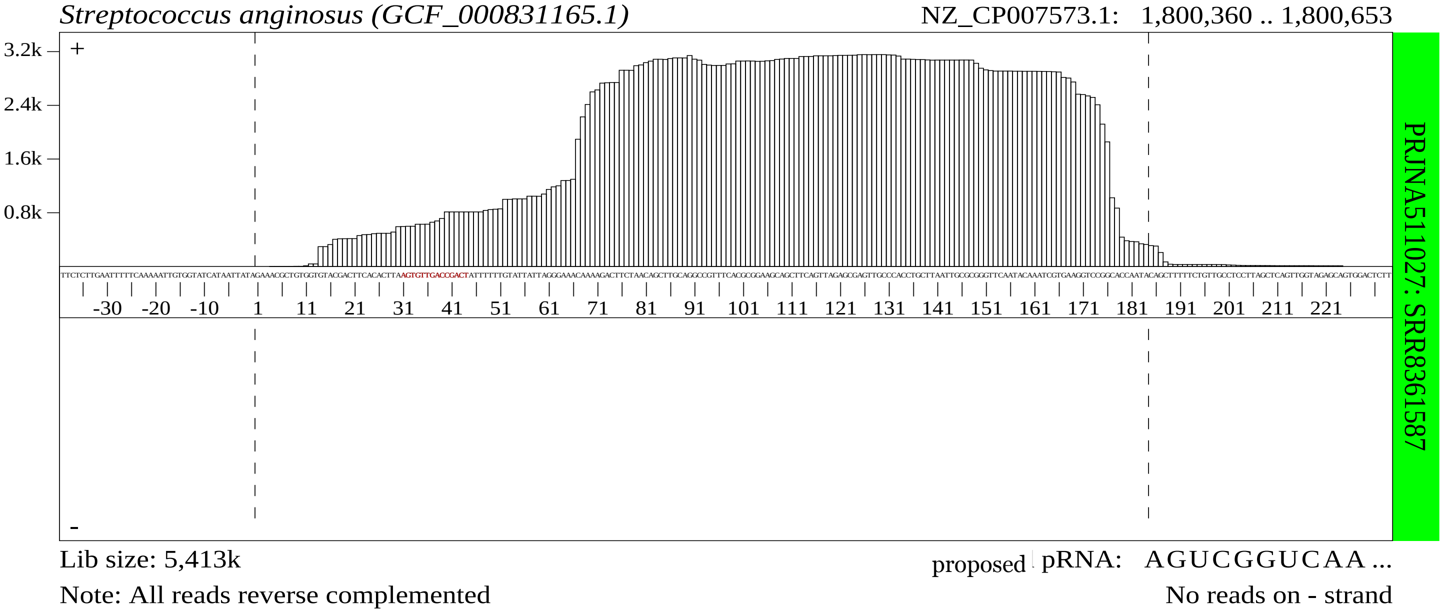

# Streptococcaceae

## *Streptococcus pneumoniae (6)*

*Streptococcus pneumoniae (GCF_000817005.1)* NZ_CP007593.1: 1,847,597 .. 1,847,890

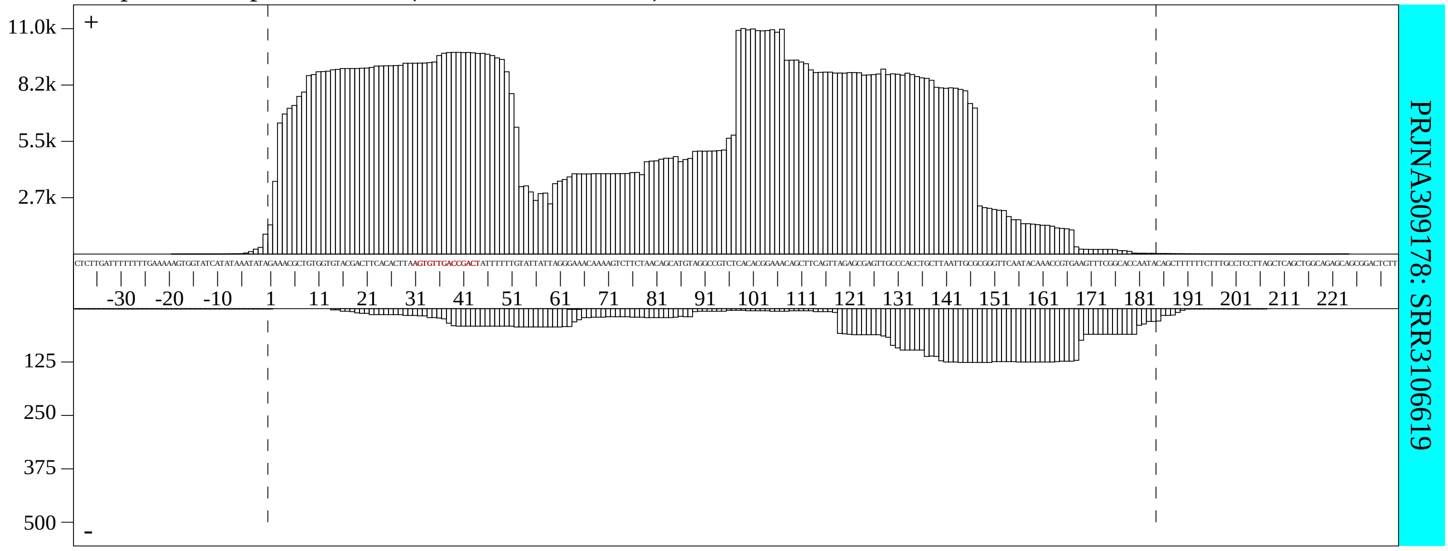


Lib size: 25,729k proposed pRNA: AGUCGGUCAA ...

*Streptococcus pneumoniae (GCF_000817005.1)* NZ_CP007593.1: 1,847,597 .. 1,847,890

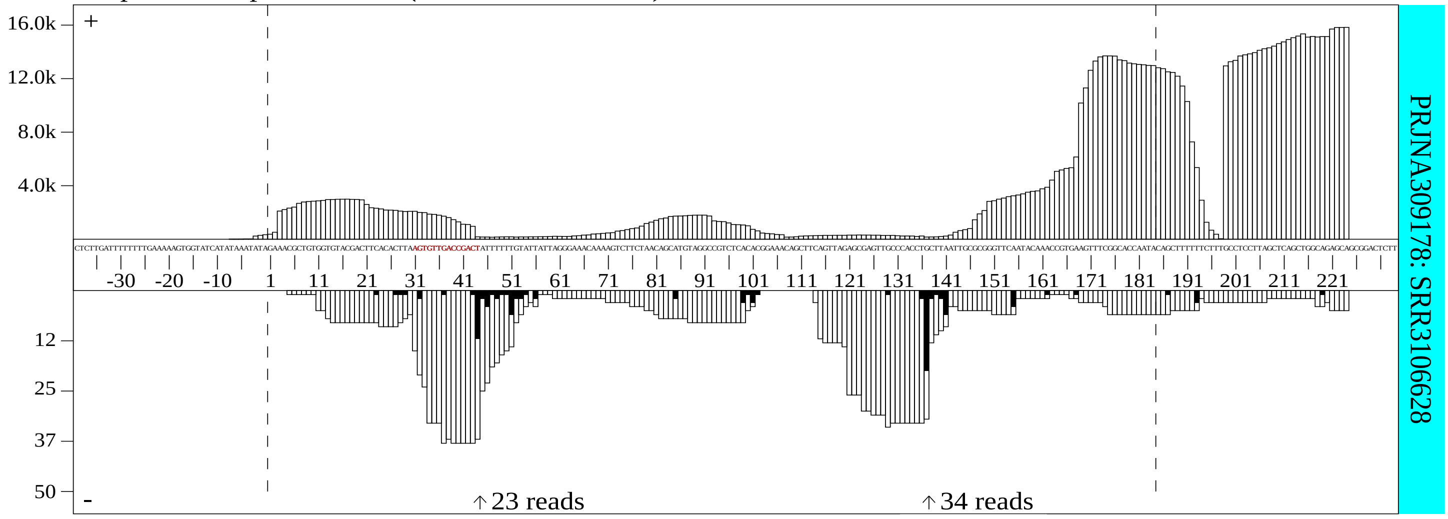


Lib size: 10,002k proposed pRNA: AGUCGGUCAA ...

Found pRNA: AGUCGGUCAA ...

# Streptococcaceae

## *Streptococcus pyogenes (9)*

*Streptococcus pyogenes (GCF_000743015.1)* NZ_CP008926.1: 815,811 .. 816,108

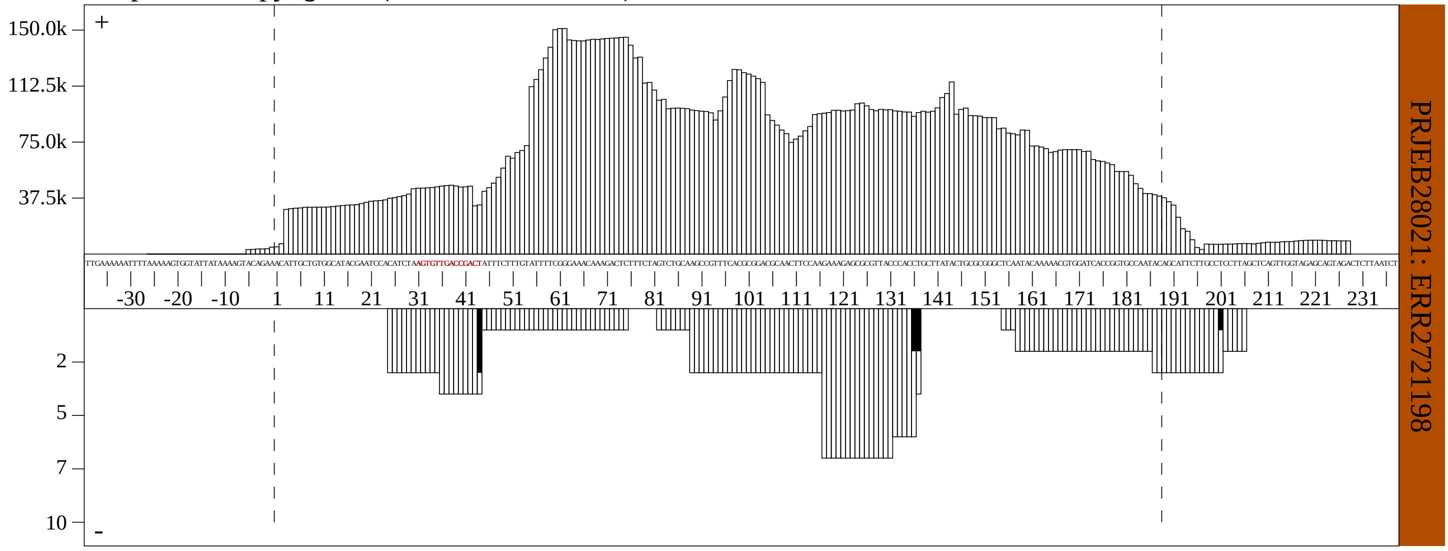


Lib size: 30,119k proposed pRNA: AGUCGGUCAA ...

*Streptococcus pyogenes (GCF_000743015.1)* NZ_CP008926.1: 815,811 .. 816,108

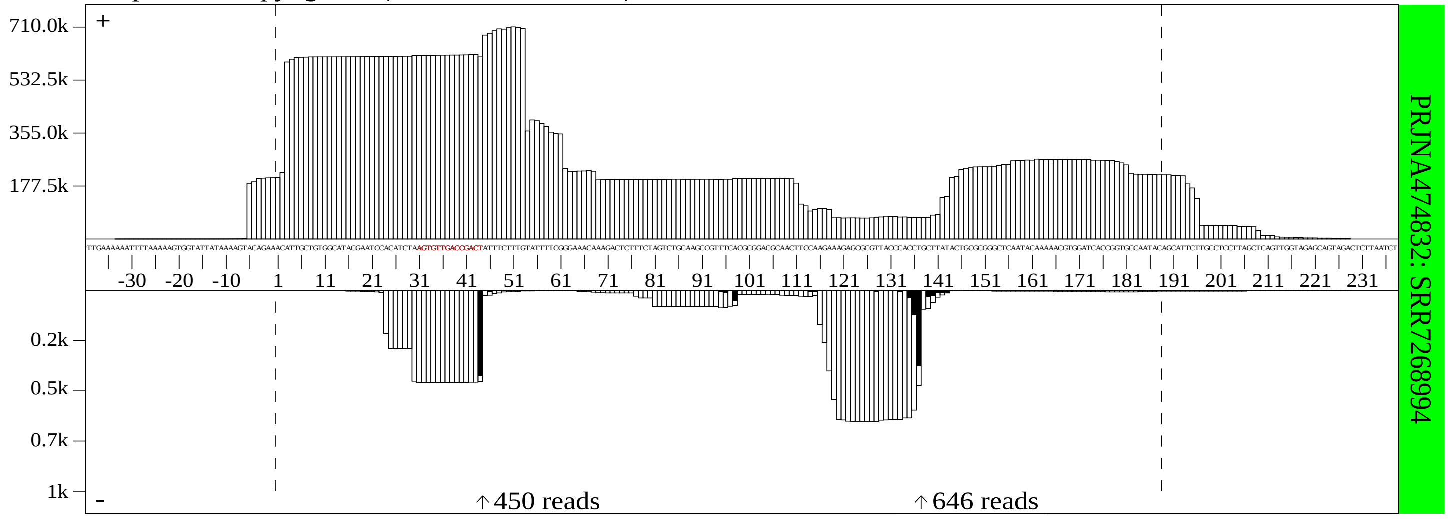


Lib size: 29,147k proposed pRNA: AGUCGGUCAA ...

Found pRNA: AGUCGGUCAA ...

*Streptococcus pyogenes (GCF_000743015.1)* NZ_CP008926.1: 815,811 .. 816,108

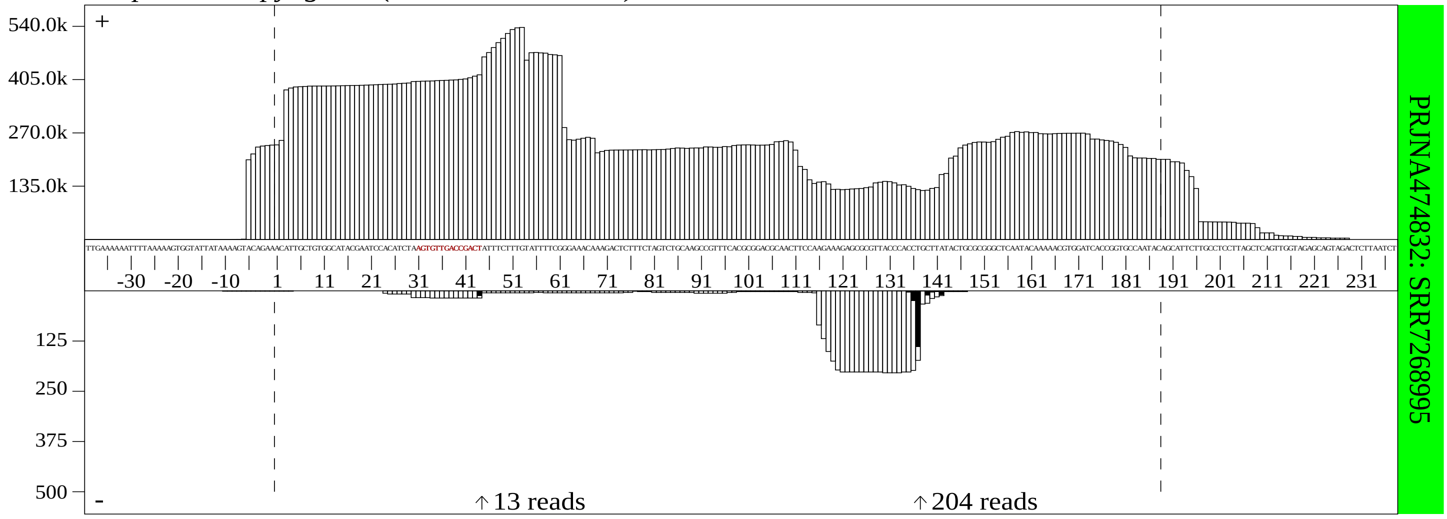


Lib size: 27,158k proposed pRNA: AGUCGGUCAA ...

Found pRNA: AGUCGGUCAA ...

# Streptococcaceae

## *Streptococcus pyogenes (9)*

*Streptococcus pyogenes (GCF_000743015.1)* NZ_CP008926.1: 815,811 .. 816,108

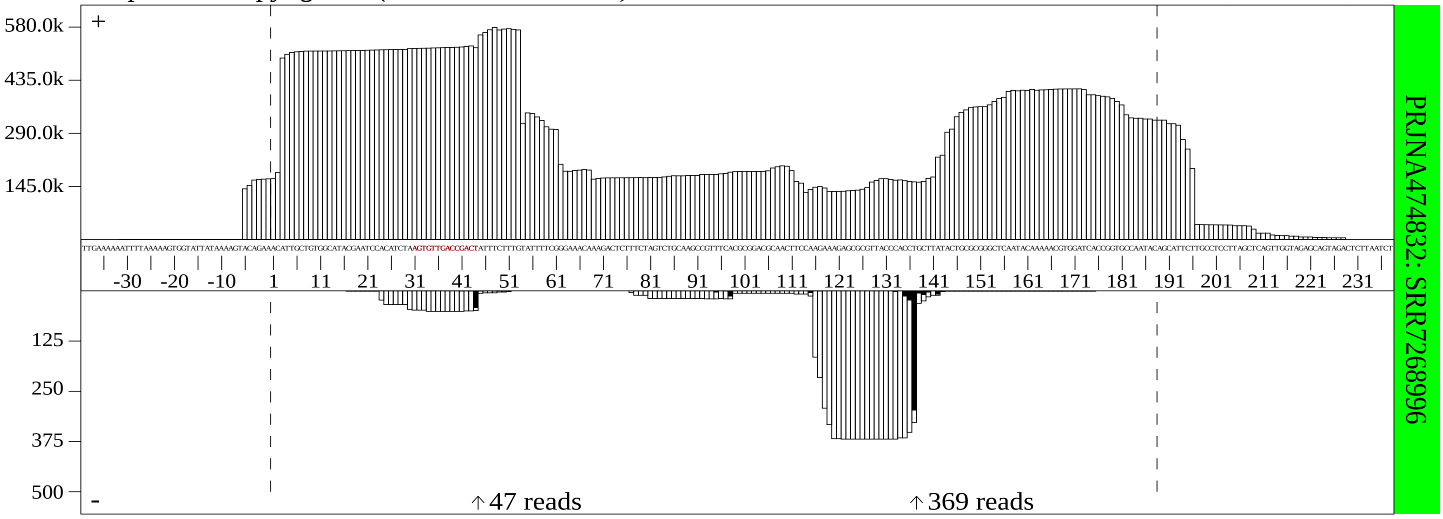


Lib size: 32,335k

proposed pRNA: AGUCGGUCAA ...
Found pRNA: AGUCGGUCAA ...

*Streptococcus pyogenes (GCF_000743015.1)* NZ_CP008926.1: 815,811 .. 816,108

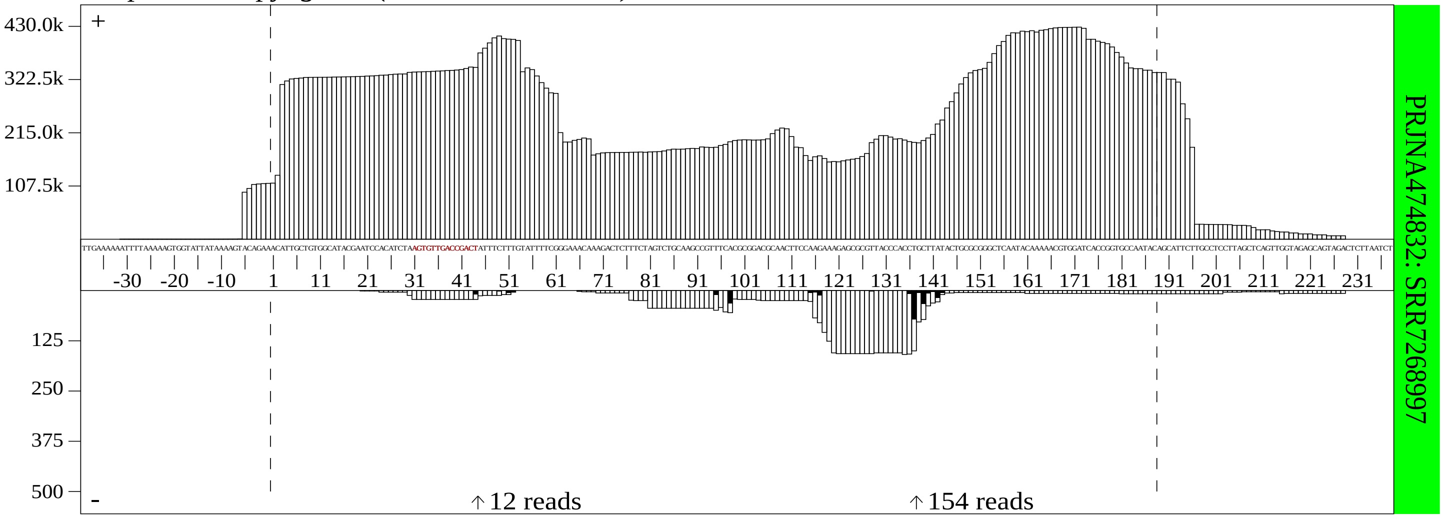


Lib size: 42,768k

proposed pRNA: AGUCGGUCAA ...
Found pRNA: AGUCGGUCAA ...

*Streptococcus pyogenes (GCF_000743015.1)* NZ_CP008926.1: 815,811 .. 816,108

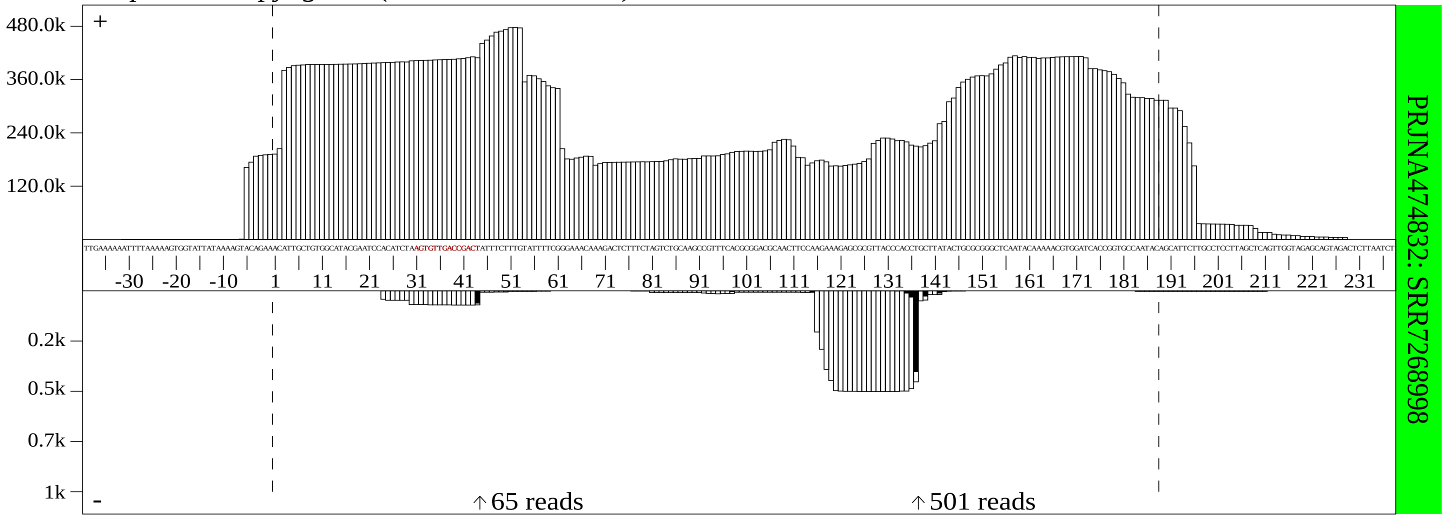


Lib size: 30,211k

proposed pRNA: AGUCGGUCAA ...
Found pRNA: AGUCGGUCAA ...

# Streptococcaceae

## *Streptococcus pyogenes (9)*

*Streptococcus pyogenes (GCF_000743015.1)* NZ_CP008926.1: 815,811 .. 816,108

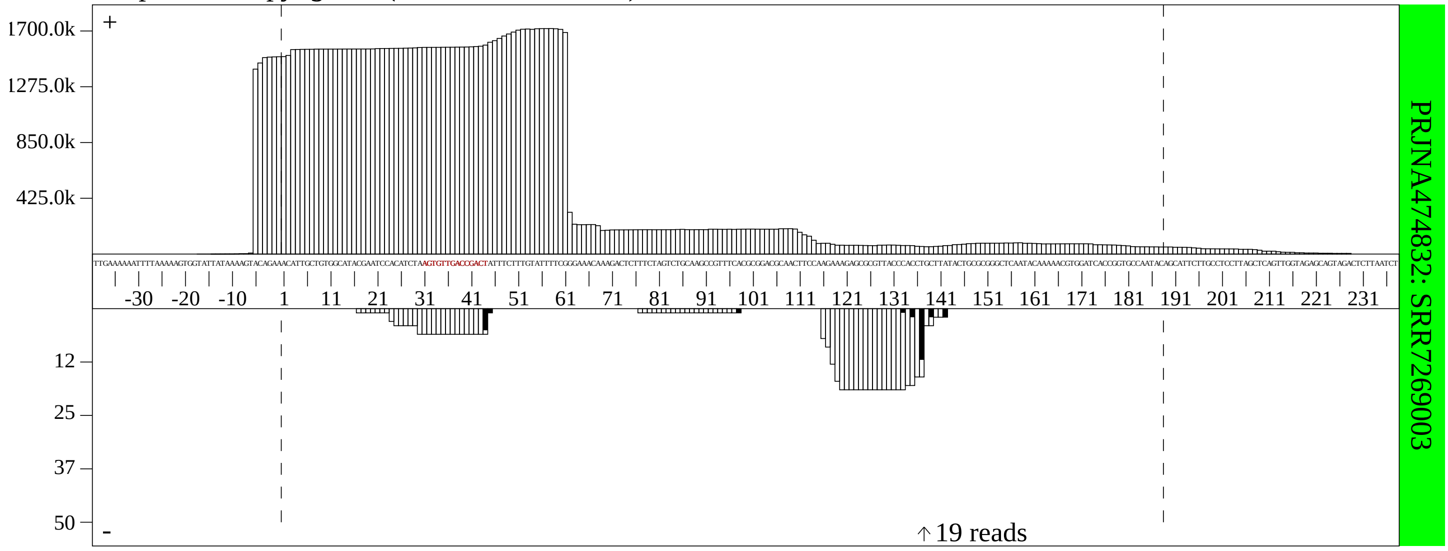


Lib size: 25,215k

proposed pRNA: AGUCGGUCAA ...

*Streptococcus pyogenes (GCF_000743015.1)* NZ_CP008926.1: 815,811 .. 816,108

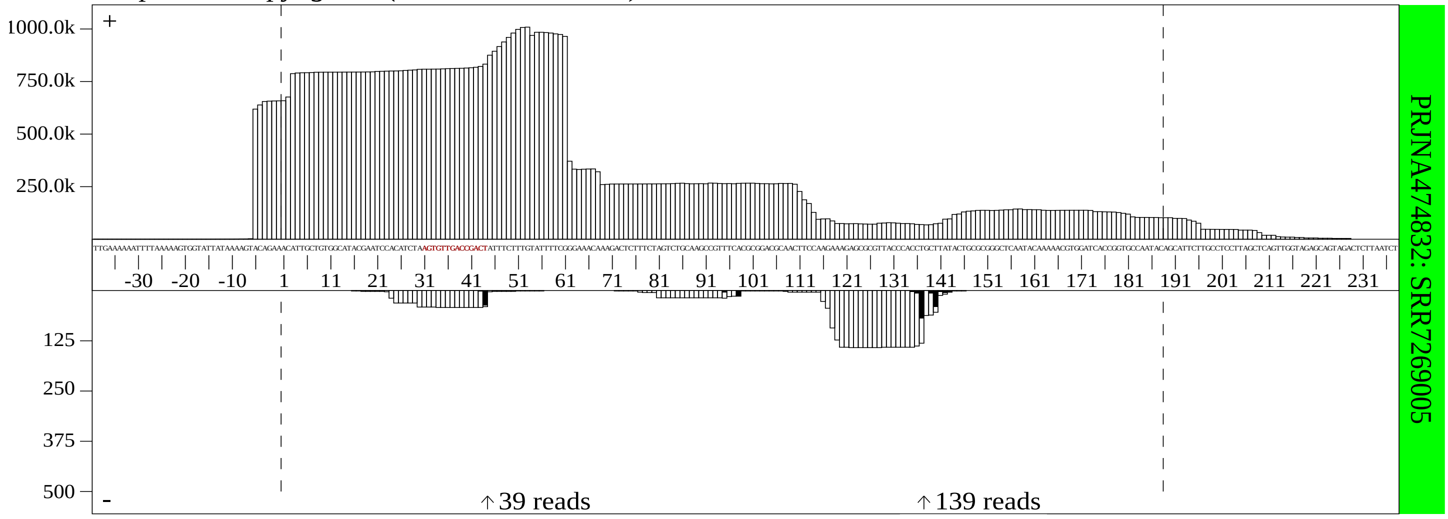


Lib size: 34,780k

proposed pRNA: AGUCGGUCAA ...
Found pRNA: AGUCGGUCAA ...

*Streptococcus pyogenes (GCF_000743015.1)* NZ_CP008926.1: 815,811 .. 816,108

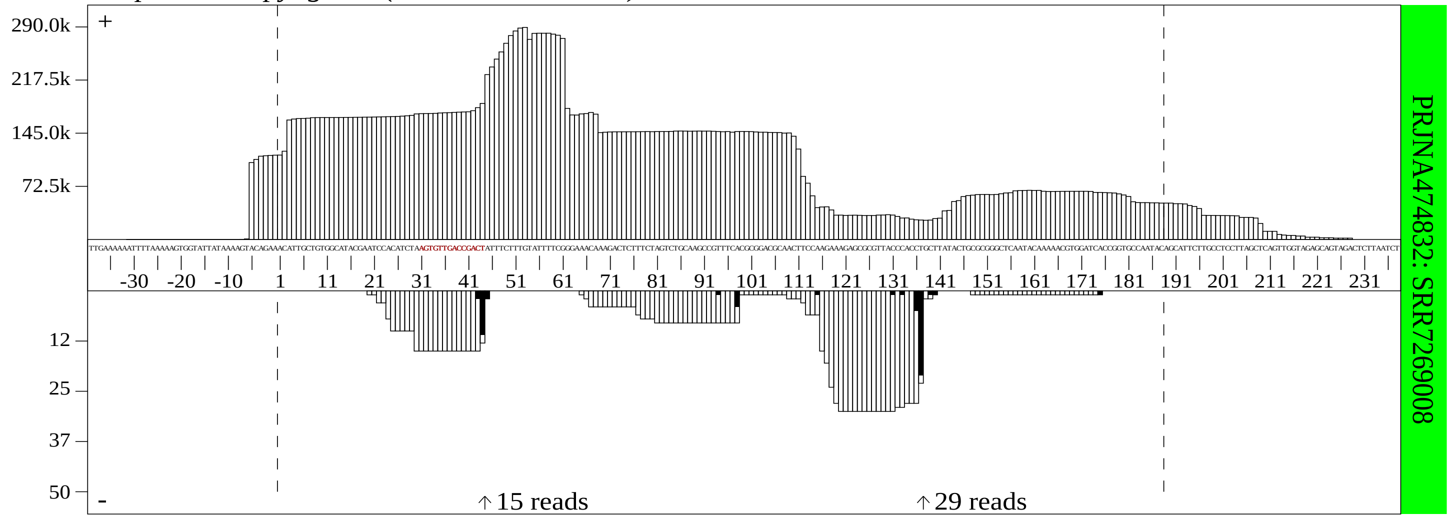


Lib size: 20,078k

proposed pRNA: AGUCGGUCAA ...
Found pRNA: AGUCGGUCAA ...

# Streptococcaceae

## *Streptococcus suis (2)*

*Streptococcus suis (GCF_000993745.1)* NZ_CP007497.1: 2,018,057 .. 2,018,350

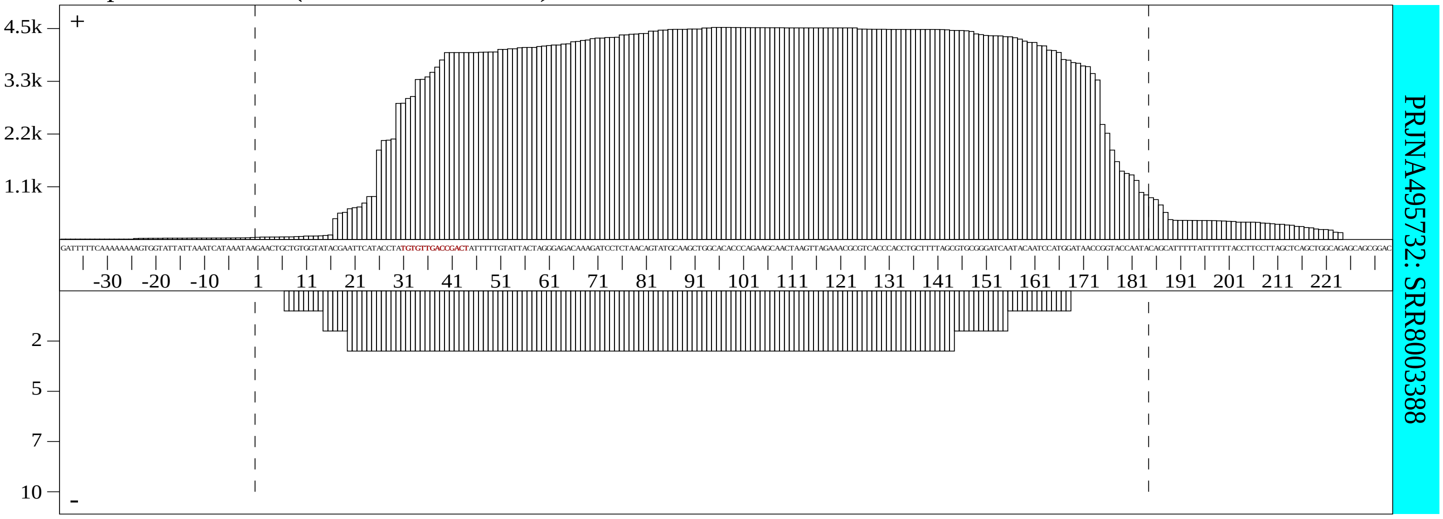


Lib size: 5,248k proposed pRNA: AGUCGGUCAA ...

Note: All reads reverse complemented

*Streptococcus suis (GCF_000993745.1)* NZ_CP007497.1: 2,018,057 .. 2,018,350

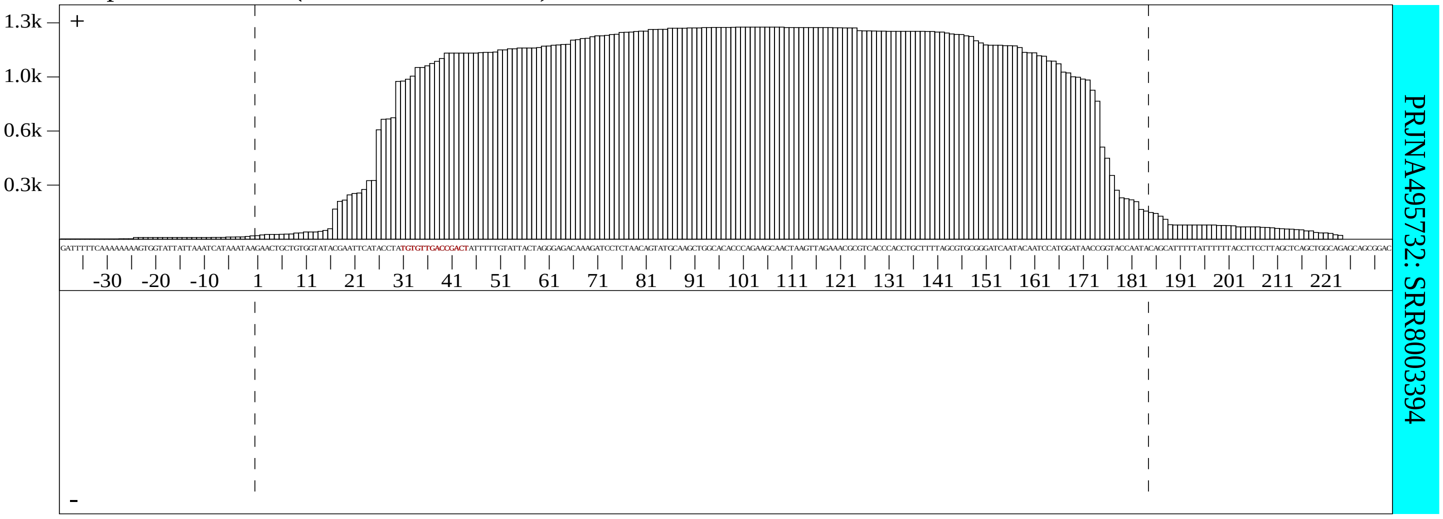


Lib size: 6,793k proposed pRNA: AGUCGGUCAA ...

Note: All reads reverse complemented No reads on - strand
