# Supplementary material for: Insights into 6S RNA in lactic acid bacteria (LAB)
Source: BMC Genom Data. 2021 Sep 3;22:29. doi: 10.1186/s12863-021-00983-2 (PMC8414754; doi:10.1186/s12863-021-00983-2)
Supplement: Supplementary file 8 — Additional file 8 6S RNA evidence in Weissella (pdf). RNA-Seq data, genomic context and sequences of putative 6S RNA loci in Weissella. [file 12863_2021_983_MOESM8_ESM.pdf]

Additional File 8 — 6S RNA evidence in *Weissella*

Supplemental Figure 1: Genomic context of *rarA* in *Weissella koreensis* and *Weissella confusa* mapped RNA-Seq data from bioprojects PRJNA306639 and PRJNA532838. The number of mapped reads is indicated on the right. Conditions are overlayed in different colors. As for main Figure 2, putative Rho-independent terminators are indicated by red hexagons. Genes in close proximity (<20 nt) are indicated by a semicircle connecting them. The data verifies active transcription of the predicted 6S RNA in *W. koreensis*. No prediction was found for *W. confusa*. However, similar transcriptional activity is observed for the expected locus immediately downstream of *rarA*.

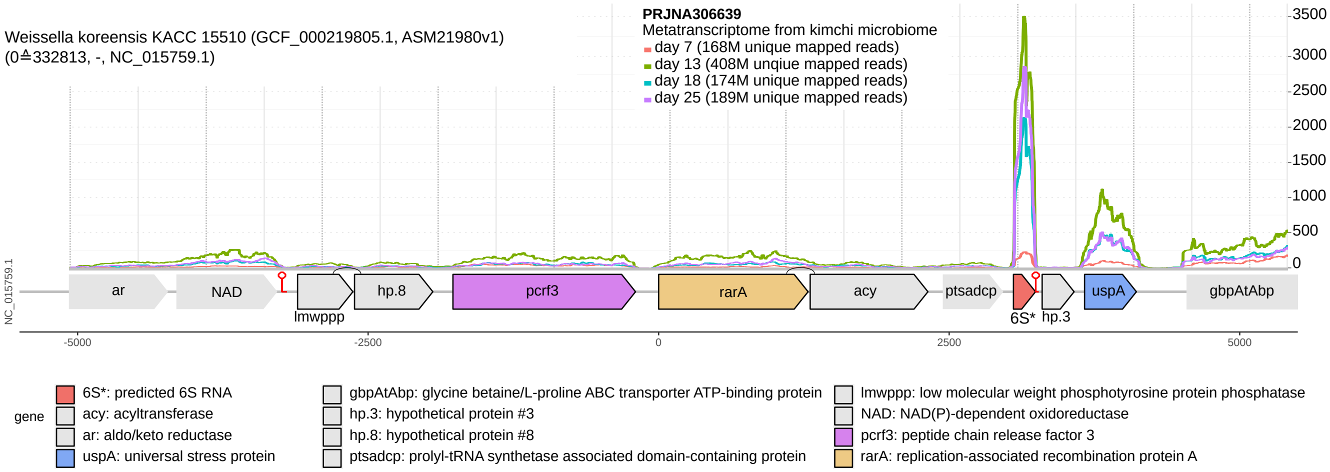

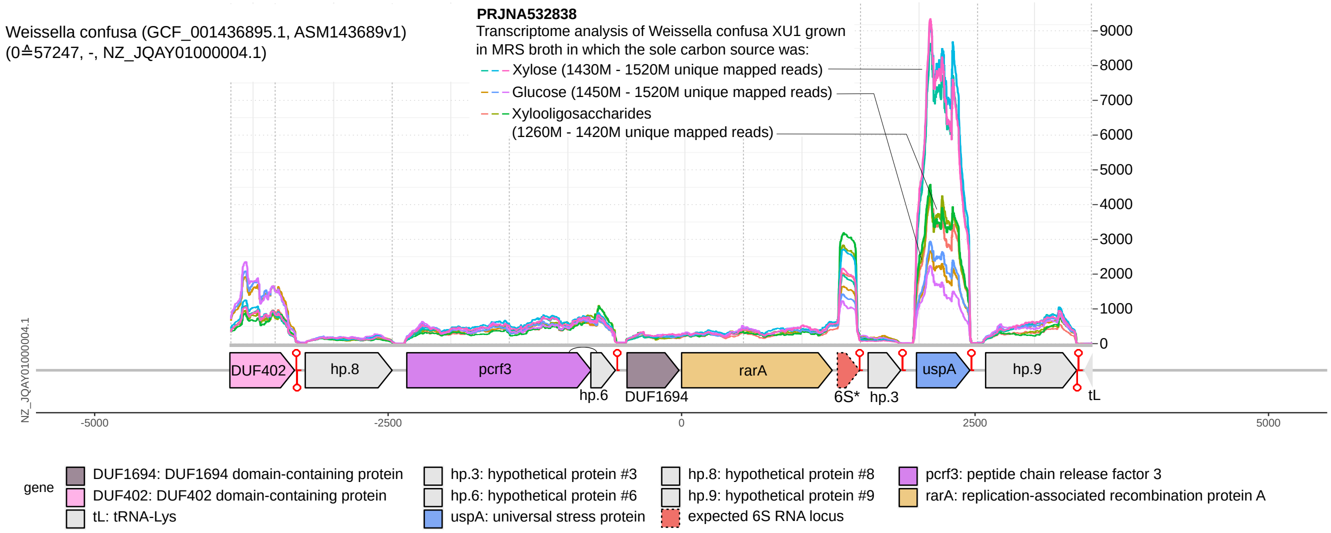

Supplemental Figure 2: Genomic context of *rarA* in further *Weissella* species. For each species, one representative strain is shown. Typically, *rarA* is followed by an intergenic region that is closed by a Rho-independent terminator. In three species, a low-scoring 6S RNA candidate was predicted in this locus (highlighted in red). We assume that a similar transcript is produced from the remaining intergenic regions of the other species.

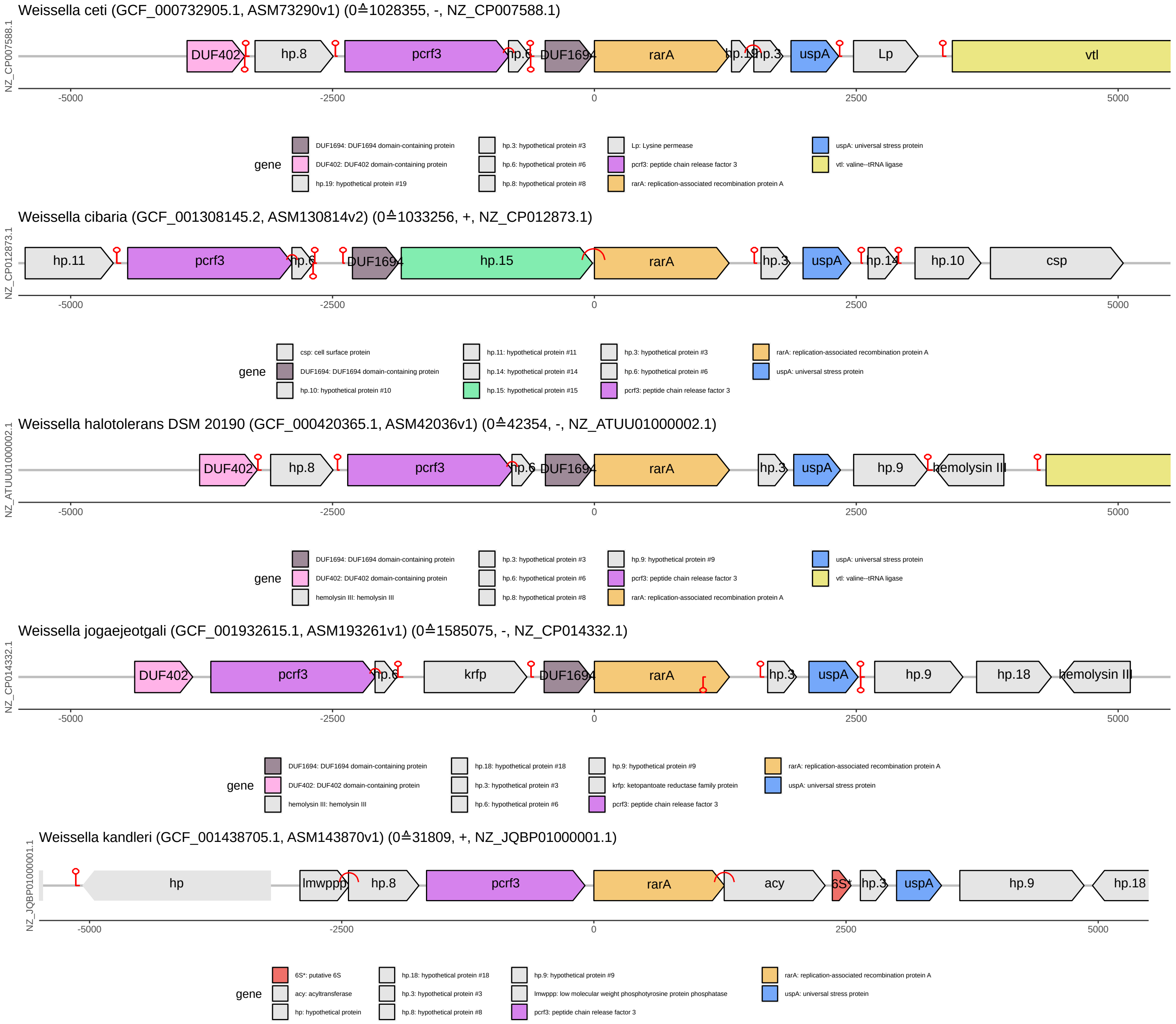

Weissella minor (GCF_001437425.1, ASM143742v1) (0≙85550, +, NZ_JQCD01000018.1)

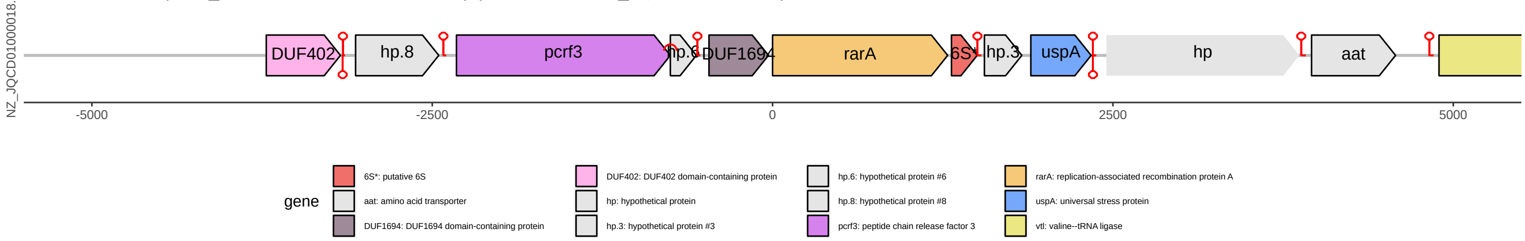

Weissella oryzae SG25 (GCF_000691805.2, ASM69180v2) (0≙139350, -, NZ_DF820486.1)

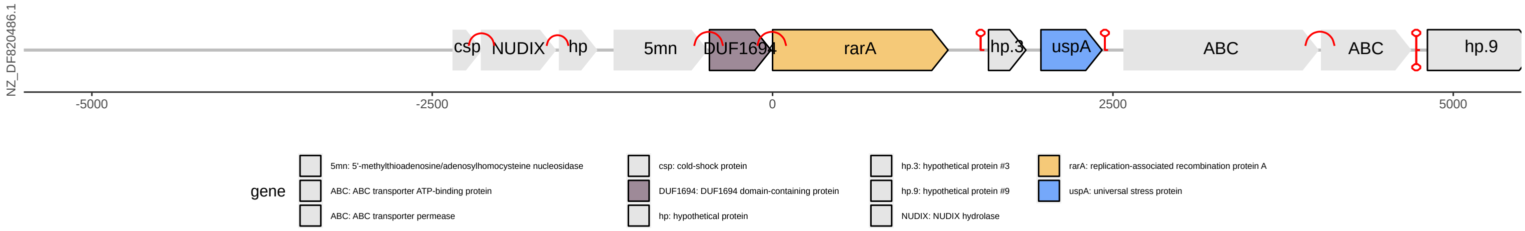

Weissella paramesenteroides (GCF_002386265.1, ASM238626v1) (0≙243024, +, NZ_CP023501.1)

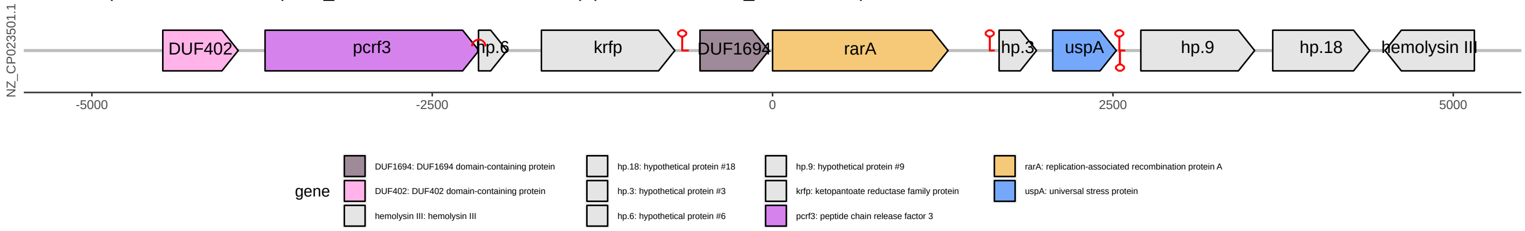

Weissella soli (GCF_003353445.1, ASM335344v1) (0≙42927, +, NZ_QRAS01000004.1)

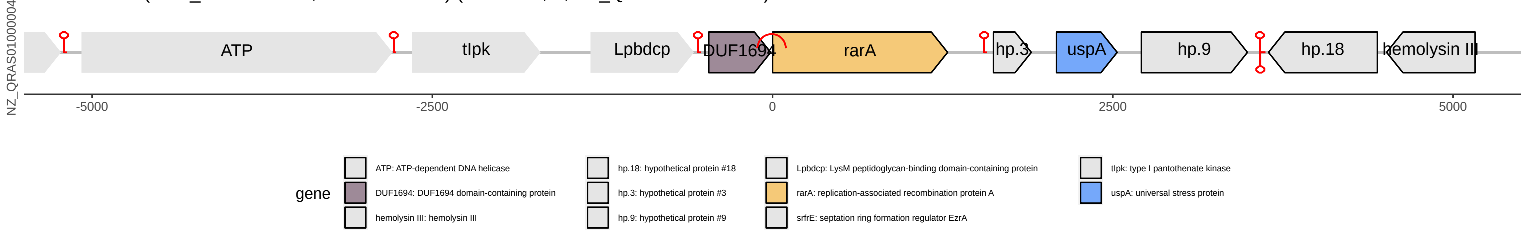

Weissella viridescens (GCF_900216215.1, MFPC16A2805a-v1) (0≙575554, -, NZ_LT907932.1)

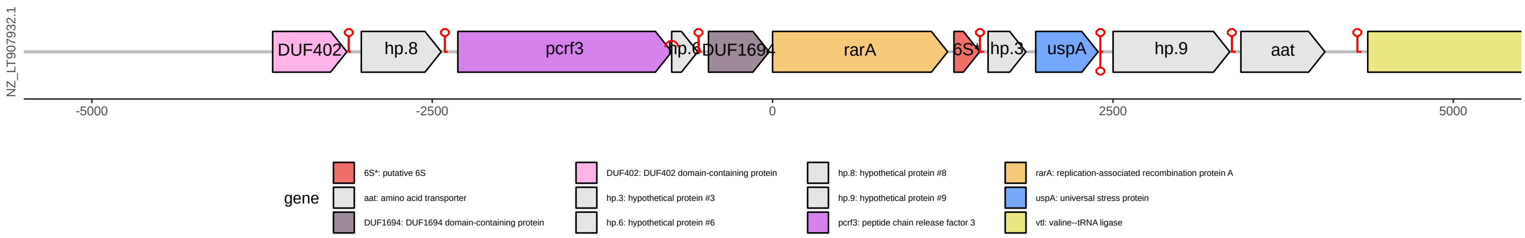

Supplemental Figure 3: Structural alignment of predicted 6S RNAs in *Weissella* species. *W. confusa* was predicted only based on RNA-Seq data (see Supplemental Figure 3 above).

```
                 ......((((((((.((.(((((((......((((..((((((((.............((
kandleri         A-AUUACCUGGUCGUACGCGUGAUUCAUAAUAUCAUAAUGUUGCUUUACAAC---UUUCU 56
koreensis        AAAUUUCUUGGACGUACGCGUGAUCC-UAUAUUCAUUAUGUUGCUUUACAACCUUUUUCU 59
koreensis_KACC   AAAUUUCUUGGACGUACGCGUGAUCC-UAUAUUCAUUAUGUUGCUUUACAACCUUUUUCU 59
confusa          ----UCCUUGGACGUACGCAUGAUUCCUACUA-UAUGUAGUUGCUUUACAACCAUAU--U 53
viridescens      AAAUUCCUUGAACAUACGCGUGAUUC-AAUUAUCUA-AAGUUGCUUUACAA--AUUUUCG 56
minor            GAAUUCCUUGAACAUACGCGUGGUUC-UAUUAUCUA-AAGUUGCUUUACACUAAUUUUCG 58
                 .........10........20........30........40........50.........

                 ((..(((((.((.((((((.(((((.(((.((((((.........))))))))).....)
kandleri         AAACGGGAAUGGCGGAAAGCCGGCGGAGCAUGCCAU-GAC---AUGUGGUAGCGACAAAC 112
koreensis        AAACGGGAAUGGCGGAAAGCCGGCGGAGCAUACCUU-AAC---ACGAGGUAGCUCCAAAU 115
koreensis_KACC   AAACGGGAAUGGCGGAAAGCCGGCGGAGCAUACCUU-AAC---ACGAGGUAGCUCCAAAU 115
confusa          GACCGGGGAUGUCGGGAAGCCGGCGAGGCAAGCUAU-UUC---ACUUAGUAGCCGCUAAA 109
viridescens      AA-UGGGGAUGUCGGGAAGCCGGCGGGGCAUGCCAACCAAUGCUGUUGGAAGUCGUUAAC 115
minor            AA-UGGGGAUGUCGGGAAGCCGGCGGGGCACGCCAACUACG--GGUUGGAAGCCGUCAAC 115
                 .........70........80........90........100.......110........

                 )))).))).....))))).)).)))....))))))).))))).)))).)))))).)...)
kandleri         GCUGUCUUAGU-GCCCACUUU-CUUAUA-CUAGGAGCCAACU-UUGACGAAUCAUCUAAC 168
koreensis        GCUGUCUUAGU-ACCCACUUU-CUUAUC-UUAGGAGCCAACA-AUGACGGAUCAACUAAC 171
koreensis_KACC   GCUGUCUUAGU-ACCCACUUU-CUUAUC-UUAGGAGCCAACA-AUGACGGAUCAACUAAC 171
confusa          GCUGACUUAUUCACCCACUUA-CCUAUAAUCUGGAGUCAACUCAUUGUGAAUCG-UUAAC 167
viridescens      GCUGACUUAUUCACCCACCUUUGCCA----UCGAAGCCAUCU-UAGACGAAUCAUCUAAC 170
minor            GCUGACUUAUUCACCCACCUUUGCCA----UCGAAGCCGACU-UAGACGAAUCAUCUAAC 170
                 .........130.......140.......150.......160.......170........

                 ))))))...)))).....................
kandleri         GGCGACAUACAAGUUUU---------------UU 187
koreensis        GGCGCCAAUCAAGUGUUU--------------AC 191
koreensis_KACC   GGCGCCAAUCAAGUGUUU--------------AC 191
confusa          GGCGCCAAUCAAGUGUUCGUGCGCUACCACGGUU 201
viridescens      GGUGUUAUACAAGUGUUU--------------AC 190
minor            GGUGUUAUACAAGUGUUC--------------GU 190
                 .........190.......200.......210..
```
